# Psychometric properties of the Swedish cardiac anxiety questionnaire: a Rasch analysis

**DOI:** 10.1038/s41598-025-28073-8

**Published:** 2025-11-24

**Authors:** Philip Leissner, Magnus Johansson, Katarina Mars, Claes Held, Robin Hofmann, Erik M.G. Olsson

**Affiliations:** 1 Department of Women’s and Children’s Health, Uppsala University, Uppsala, Sweden; 2 Division Safety and Transport, Measurement Science and Technology, RISE Research Institutes of Sweden, Stockholm, Sweden; 3 Centre for Psychiatry Research, Department of Clinical Neuroscience, Karolinska Institutet, Stockholm, Sweden; 4 Department of Clinical Science and Education, Division of Cardiology, Karolinska Institutet, Södersjukhuset, Stockholm, Sweden; 5 Department of Medical Sciences, Cardiology, Uppsala University, Uppsala, Sweden; 6 Uppsala Clinical Research Center, Uppsala University, Uppsala, Sweden; 7 Uppsala University, MTC-huset, Dag Hammarskjölds väg 14B, Akademiska sjukhuset, Uppsala, 751 85 Sweden

**Keywords:** CAQ, Rasch analysis, Confirmatory factor analysis, Myocardial infarction, Cardiology, Diseases, Health care, Medical research

## Abstract

Cardiac anxiety (CA) is a commonly occurring condition in populations with both cardiac and mental health conditions. It is assessed using the CA questionnaire (CAQ), which has been translated to several languages and its validity and reliability has been investigated using classical test theory. The factor structure of the CAQ remains uncertain, and no study has investigated the psychometric properties of the CAQ using modern test theory approaches. The aim of this study was to use Rasch analysis to test the validity and reliability of the original 18-item CAQ in a Swedish sample of patients with myocardial infarction, as well as other scales, based on previous findings. Participants were 754 patients after myocardial infarction with preserved cardiac function. Data were fitted to the Rasch model, investigating dimensionality, ordering of response categories, invariance, targeting and measurement uncertainty. Additionally, a confirmatory factor analysis (CFA) was conducted using a weighted least mean squares estimator and dynamic fit indices. All three subscales indicated problems with dimensionality and several items had disordered response categories, and with some of them minimal distance between steps. Many items displayed local dependency. There were also small problems with differential item functioning. Person locations were lower than the item threshold locations, but there were no significant floor or ceiling effects. The CFA also demonstrated a poor model fit of the three subscales in the 18-item version of the CAQ. An iterative removal of items resulted in a modified 10-item version still with questionable reliability. The original 18-item version of the CAQ did not fit the Rasch model. The major problems with the 18-item version were dimensionality, local dependency, and disordered response categories. No other version of the CAQ achieved acceptable fit to the Rasch model. These results indicate that the CAQ is lacking in terms of reliability and a revision of the scale is called for.

*Trial registration*: ClinicalTrials.gov. (2017, August 09). Evaluation of Decreased Usage of Betablockers After Myocardial Infarction in the SWEDEHEART Registry (REDUCESWEDEHEART). U.S. National Library of Medicine. https//clinicaltrials.gov/study/NCT03278509.

**Supplementary Information:**

The online version contains supplementary material available at 10.1038/s41598-025-28073-8.

## Introduction

Cardiac anxiety (CA) is a type of anxiety related to fear of heart malfunctioning^1^. It was primarily conceptualized as a subtype of panic disorder (PD), referring to patients seeking cardiac care but without cardiovascular disease (CVD)^2,3^. Later, it was recognized by the psychologist Georg Eifert who argued for what was then called “cardiophobia” to be a disorder in its own right^4,5^. He continued to broaden the definition to also include hypochondriasis or disease phobia (now best described as illness anxiety disorder (IAD) or somatic symptom disorder (SSD)), and obsessive-compulsive disorder (OCD)^6^. To measure cardiophobia, an initial pilot version of the cardiac anxiety questionnaire (CAQ) was developed, consisting of 16 items with the four factors (a) cardiac disease conviction/heart awareness, (b) cardioprotective behaviour, (c) medical help seeking, and (d) hyperarousal^7^. Both healthy patients with CA, as well as cardiac inpatients, were found to report high scores on this scale^8^. A second, 18-item version of the CAQ was later developed and tested in cardiac outpatients^1^. Principal component analysis (PCA) indicated that the questionnaire would be best described by a 3-factor structure consisting of the subscales (a) fear, (b) avoidance, and (c) attention. This 18-item version has been translated to several languages and its factor structure, reliability and validity has been tested in both cardiac and non-cardiac populations^9–17^.

In a previous paper we examined the psychometric properties and the various proposed factor structures of the Swedish translation of CAQ, in a sample of patients after myocardial infarction (MI)^17^. Internal consistency, temporal stability, and convergent validity all proved acceptable. The exploratory factor analysis (EFA) resulted in a model similar to the original 3-factor solution^1^, but with three of the original items removed (4, 10, and 13). This model, and models from previous publications^9–14^, were examined using confirmatory factor analysis (CFA). Both the 15-item, 3-factor, model generated by the EFA and a 10-item, 3-factor, model^14^ demonstrated relatively good model fit based on conventional rule-of-thumb cut-off criteria^18^. As a shorter questionnaire takes less time to administer and places a lesser burden on the respondent, it is preferrable over a longer version, if retaining high reliability^19^. Thus, considering the 10-item version as a reliable form of the CAQ could be beneficial.

However, a limitation of the previous study was that the CFA of CAQ was applying a maximum likelihood estimator, which assumes continuous data with a gaussian distribution. While this is not an uncommon practice, it might generate biased or incorrect results. The weighted least squares estimator for means and variances (WLSMV) is more appropriate when dealing with ordinal data and it does not assume normally distributed responses^20–22^. Similar to this point, it has long been known that using rule-of-thumb cut-off values for CFA model fit metrics is not a good practice and will lead to erroneous results^23,24^. Instead, it has been argued to using dynamic fit index cut-offs which have a substantially superior sensitivity to misspecification^24–26^.

Furthermore, while factor analysis is one of the classical test theory (CTT) methods that has been the traditional means for construction and refinement of patient-reported outcomes, it has some limitations^27,28^. The CAQ is, like most psychological instruments, based on ordinal scores, from which you can derive rank, rather than interval or ratio relationships^29^. These scores are in theory not designed to support change scores or parametric effect sizes^30–32^. However, by fitting data to the Rasch model, these issues can be addressed, and may allow the transformation of ordinal sum scores to estimated latent scores to be used for parametric operations^33^. Additionally, while CTT approaches helps answer questions regarding internal consistency and clustering of items, Rasch additionally helps answer how individual items function, in terms of difficulty, item fit, fairness across groups, and if response categories function as intended^34^.

There remains uncertainty regarding the factor structure of the CAQ, and the traditional psychometric methods that has been used does not appropriately address the ordinal nature of questionnaire data. Furthermore, the specificity to address certain psychometric issues is lacking. As such, the aim of the current study was to re-evaluate the factor structure of the CAQ and its validity and reliability using both CFA with the WLSMV estimator and Rasch analysis, in a sample of Swedish post-MI patients, using dynamic critical values for assessing model fit.

## Methods

### Study design and participants

The sample of the present study was part of the quality-of-life substudy of the REDUCE trial (RQoL), a multi-centre, clinical, registry-based randomized controlled trial, that evaluated the effects of beta-blocker treatment on various mental health and quality of life outcomes. Detailed description of study design, protocol and outcomes are previously reported^35–37^. In brief, eligible patients had to have had an MI in the last 1–7 days, a preserved cardiac function, and provided written informed consent. In addition, for inclusion in RQoL, participants had to have had a good understanding of the Swedish language.

### Measures

This study used the Swedish translation of the 18 item CAQ^1,17^. It is used to assess heart-related anxiety and asks the participant to answer questions how often they experience certain symptoms on a response scale using five ordered categories; “Never”, “Rarely”, “Sometimes”, “Often”, “Always”, that are scored 0–4. Subscale scores are typically divided by the number of items to make them comparable. In the development study of the CAQ, Cronbach’s alpha for the total scale was α = 0.83, for subscale fear α = 0.83, for avoidance α = 0.82; and for attention α = 0.69^1^.

### Statistical analysis

A total of 18 items were analysed, by the three separate subscales described in the original article^1^. Given that the sample size was sufficiently large, missing data was handled with case-wise deletion. All analyses were conducted using R version 4.4.2^38^, and compiled into reproducible reports using Quarto. The Rasch analyses were conducted using the package easy Rasch version 0.3.6.2^39^, which makes use of conditional maximum likelihood estimation with the partial credit model^40^ as implemented by the eRm package^41^.

#### Unidimensionality

Tests of dimensionality were (1) analysis of conditional item mean-square (MSQ) infit, where an item is considered overfit when infit is below the lower critical value threshold and underfit when infit is above the upper threshold^42^ with simulation-based cut-offs, using 200 bootstrap iterations to determine critical values for each item^43^; (2) investigation of patterns in model residuals, also with simulation-based cut-offs, using 250 bootstrap iterations and the 99th percentile as critical value^44^; (3) assessment of homogeneity with a conditional likelihood ratio test (CLRT)^45^, both on the full sample but also using a non-parametric bootstrap function, restraining the sample size to 400 and with 1000 iterations^39^; (4) visual inspection of loadings on the first residual contrast for identification of clusters in the data.

#### Ordering of response categories

Comparison of item category threshold locations were conducted by visual inspection of item probability curves and item threshold locations along the latent continuum. The probability of response in each item’s response category should increase consistently with the underlying latent trait and the thresholds between categories should be ordered and well-separated^46,47^.

#### Invariance

To assess whether item threshold locations were consistent across groups of sex (male vs. female), age (< 70 years vs. > 69 years of age), education, relationship status (single vs. in a relationship), immigration status (born in Sweden vs. not born in Sweden) a Differential Item Functioning (DIF) analysis was conducted. The analysis consisted of a likelihood ratio test for determining statistically significant DIF and a cut-off of 0.5 on the logit scale was used to determine a significant magnitude^46^.

#### Targeting

The distribution of person and item-threshold locations were inspected on a person-item map, also known as a Wright map^48^. Distributions and means of the person and item locations should be similar to each other, ideally without threshold location gaps and no visible floor or ceiling effects^49,50^. Floor or ceiling effects are deemed present when over 15% of respondents attain the minimum or maximum possible score, respectively^51^.

#### Reliability and measurement uncertainty

Reliability was reported using the person separation index^52^ and the relative measurement uncertainty^53^. The PSI excludes persons with min/max score, which can cause unexpected results in some sample/item combinations, whereas the RMU is more stable across conditions and also includes a 95% highest density continuous interval (HDCI). Reliability was only reported for item combinations shown to fulfil the measurement criteria.

#### Iterative revision

For the purpose of identifying the best fitting set of items, all subscales were subjected to an iterative analysis process. In this process, items were removed based on issues identified in the Rasch analysis. Removal of items was made based on a combined judgement considering all analyses, primarily prioritizing items with underfit and local dependencies.

#### Confirmatory factor analysis

CFA was conducted using a WLSMV estimator, with dynamic cut-off values to determine model fit^25^. Empirical fit indices of standardized root mean squared residual (SRMR), root mean square error of approximation (RMSEA), and comparative fit index (CFI) were estimated and compared to levels (0–2) of misspecification set by the `dynamic` R package. In a one-factor model, the levels of misspecification correspond to the number of items that have a residual correlation with another item. These levels were estimated and presented together with the model fit indices for each subscale.

## Results

### Participants

The sample consisted of 79% males and the mean age was 64.4 years (SD: 10.3). Demographic characteristics and mean (SD) scores on the CAQ subscales are presented in Table 1. Out of 806 participants, 52 (6%) had missing data on at least one variable and were excluded from all analyses.

**Table 1:** Descriptive statistics.

	Total (*n* = 754)
Age, mean (SD)	64.4 (10.3)
Male, % (n)	79% (592)
Highest level of Education, % (n)	
Secondary School	21% (162)
High School	42% (317)
University studies ≤ 3 years	19% (130)
University studies > 3 years	17% (145)
In a relationship, % (n)	81% (610)
Born in Sweden, % (n)	89% (669)
Cardiac Anxiety Questionnaire, Fear subscale, mean (SD)	1.24 (0.76)
Cardiac Anxiety Questionnaire, Avoidance subscale, mean (SD)	1.23 (0.80)
Cardiac Anxiety Questionnaire, Attention subscale, mean (SD)	0.85 (0.61)

CAQ: Cardiac Anxiety Questionnaire.

### Rasch analysis

All code and detailed analyses generated with Quarto (R) are provided in the Supplementary Material. Below we briefly summarize the initial findings from the original set of items with their three factors and later the item sets by Dragioti et al.^14^.

**Table:** 

Item	Description
1	I pay attention to my heart beat
2	I avoid physical exertion
3	My racing heart wakes me up at night
4	Chest pain/discomfort wakes me up at night
5	I take it easy as much as possible
6	I check my pulse
7	I avoid exercise or other physical work
8	I can feel my heart in my chest
9	I avoid activities that make my heart beat faster
10	If tests come out normal, I still worry about my heart
11	I feel safe being around a hospital, physician, or other medical facility
12	I avoid activities that make me sweat
13	I worry that doctors do not believe my chest pain/discomfort is real
14	When I have chest discomfort or I feel my heart is beating fast I worry that I may have a heart attack
15	When I have chest discomfort or I feel my heart is beating fast I have difficulty concentrating on anything else
16	When I have chest discomfort or I feel my heart is beating fast I get frightened
17	When I have chest discomfort or I feel my heart is beating fast I like to be checked out by a doctor
18	When I have chest discomfort or I feel my heart is beating fast I tell my family or friends

#### Distribution of response categories

**Fig. 1:**
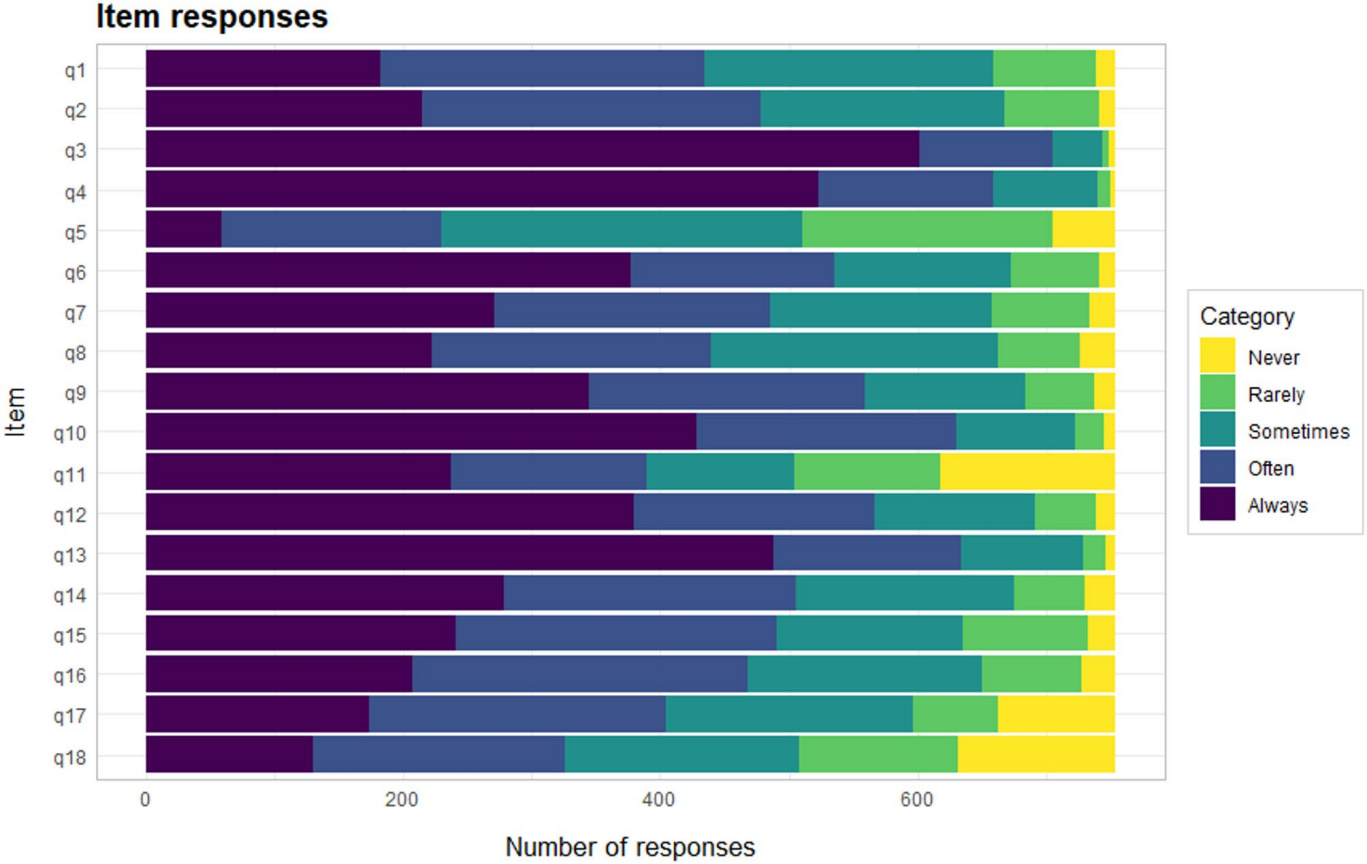
Distribution of response data in ordered categories.

The distribution across response categories (Fig. 1), shows that the highest category was rarely used by respondents, except for items 5, 11, 17 and 18. Items 3, 4 and 13 also stand out with more than half of participants endorsing the lowest category, and extremely few endorsing the highest categories, indicating high item difficulties relative to the respondents’ position of attitude.

#### Fear subscale

The 8-item version of this subscale showed several indications for more than one dimension. All items except item 10 showed problems with conditional item infit. Most notably, item 11 and 18 were strongly underfit (see Table 2). Correlations between standardized residuals were identified above the relative cut-off (*r* = 0.027) between item 10 and 13 (*r* = 0.08), 14 and 15 (*r* = 0.19), 14 and 16 (*r* = 0.21), 15 and 16 (*r* = 0.38), and 17 and 18 (*r* = 0.18). The CLRT for the full model showed statistically significant misfit (*p* < 0.001) and the bootstrap calculation, restraining the sample size to 400, showed statistically significant misfit for 100% of the iterations. By inspection of first residual contrast factor, it seems that items 11, 17 and 18 cluster together.

**Fig. 2:**
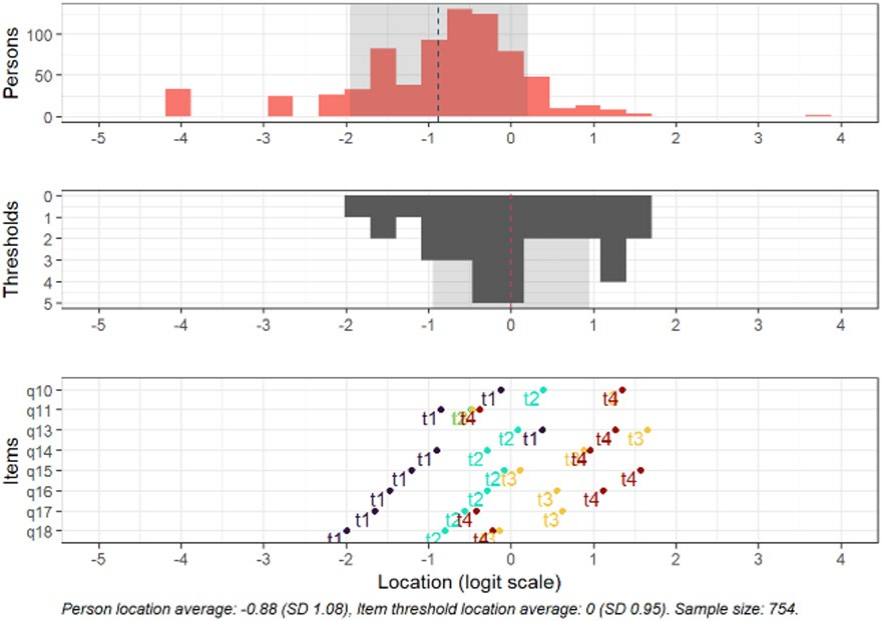
Targeting for the subscale fear. Separately shown for persons, thresholds and items. Person location average: −0.88, Item threshold location average: 0 (SD 0.95). Sample size: 754.

Regarding ceiling and floor effects, 34 (4.5%) respondents endorsed a minimum score on this subscale, and 1 (0.1%) endorsed a maximum score. Based on the logit scale, 11.3% had locations below the lowest item threshold (-1.99), and 0.1% of respondents had person locations above the highest item threshold (1.65).

All items except items 15 and 16 demonstrated disordered response category thresholds and item 11 also demonstrated minimal distance between response thresholds (see item locations, Fig. 2).

In this subscale, there was no significant DIF observed for the variables sex, age, relationship status, education or immigration status (all logit values < 0.5). However, some DIF was observed in the interaction between sex and relationship status for item 18 (logit value = 0.551), where male participants more often endorsed the higher categories if they live in a relationship (*n* = 501) compared to those who did not live in a relationship (*n* = 91).

**Table 2:** Fear subscale, conditional item infit.

Item		Infit MSQ	Infit thresholds
10	If tests come out normal, I still worry about my heart	0.951	[0.884, 1.124]
11	I feel safe being around a hospital, physician, or other medical facility	**1.386**	[0.879, 1.154]
13	I worry that doctors do not believe my chest pain/discomfort is real	**1.211**	[0.886, 1.159]
14	When I have chest discomfort or I feel my heart is beating fast I worry that I may have a heart attack	**0.754**	[0.894, 1.114]
15	When I have chest discomfort or I feel my heart is beating fast I have difficulty concentrating on anything else	**0.832**	[0.9, 1.096]
16	When I have chest discomfort or I feel my heart is beating fast I get frightened	**0.722**	[0.89, 1.137]
17	When I have chest discomfort or I feel my heart is beating fast I like to be checked out by a doctor	**0.806**	[0.844, 1.124]
18	When I have chest discomfort or I feel my heart is beating fast I tell my family or friends	**1.418**	[0.877, 1.095]

MSQ values based on conditional calculations (*n* = 754 complete cases). Simulation based thresholds from 200 simulated datasets.

Bold indicates item misfit.

#### Avoidance subscale

**Fig. 3:**
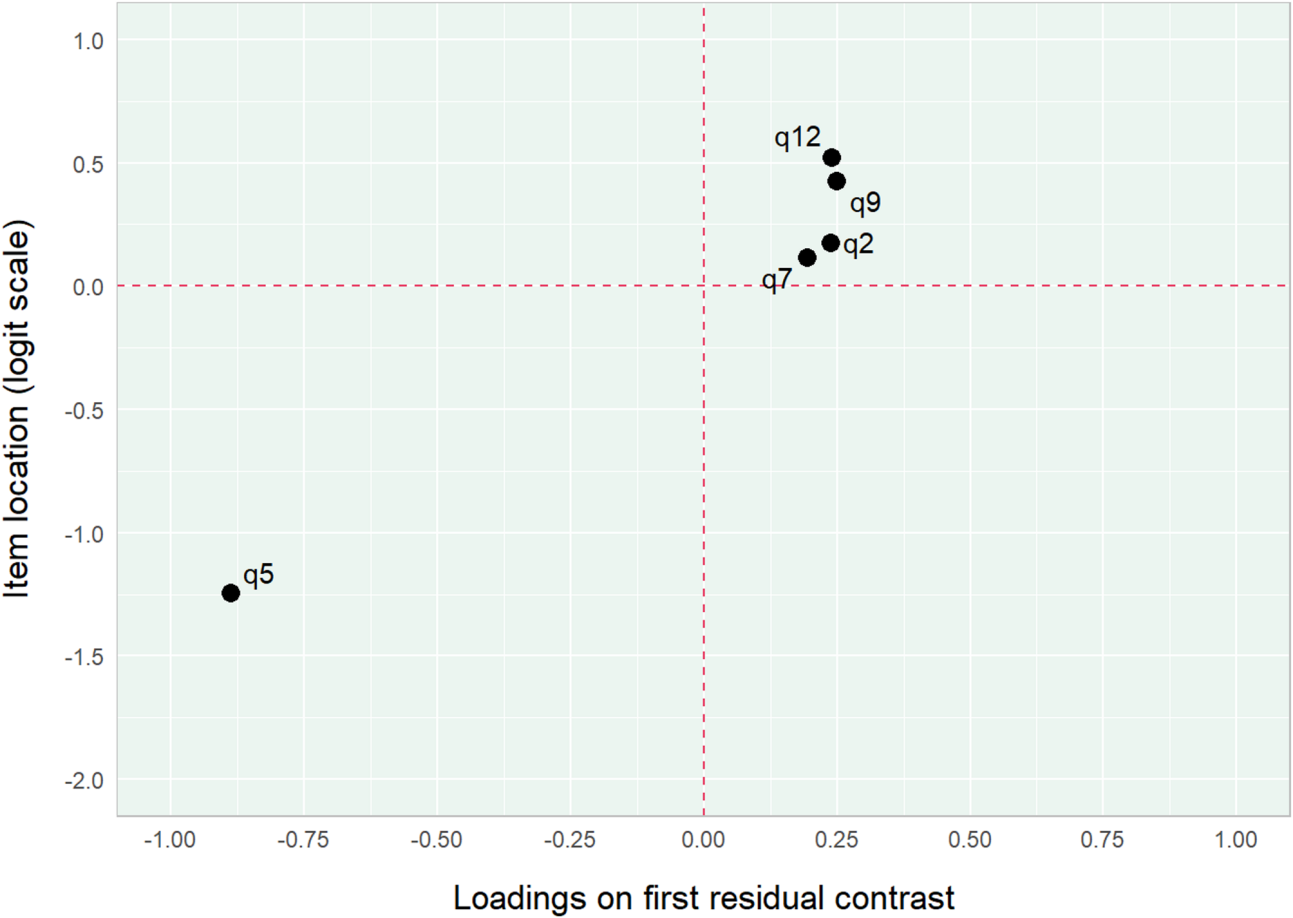
Item loadings and loadings on first residual contrast factor for the subscale Avoidance.

The 5-item avoidance subscale showed some issues with dimensionality. In terms of conditional item infit, three items (2, 7 and 12) were slightly overfit and item 5 was strongly underfit (see Table 3). Residual correlations were identified (relative cut-off *r* =-0.087) between item 2 and 7 (*r* = 0.05) and 9 and 12 (*r* = 0.04). The CLRT for the full model showed statistically significant misfit (*p* < 0.001) and the bootstrap calculation, restraining the sample size to 400, showed statistically significant misfit for 99.6% of the iterations. By inspection of residual contrast item 5 clearly stands out from the other items (see Fig. 3).

Regarding ceiling and floor effects, 36 (4.8%) respondents endorsed a minimum score on this subscale, and 2 (0.3%) endorsed a maximum score. Based on the logit scale, 4.8% had locations below the lowest item threshold (-3.92), and 1.1% of respondents had person locations above the highest item threshold (2.53).

**Table 3:** Avoidance subscale, conditional item infit.

Item		Infit MSQ	Infit thresholds
2	I avoid physical exertion	**0.812**	[0.873, 1.109]
5	I take it easy as much as possible	**1.577**	[0.891, 1.116]
7	I avoid exercise or other physical work	**0.833**	[0.916, 1.137]
9	I avoid activities that make my heart beat faster	0.925	[0.903, 1.117]
12	I avoid activities that make me sweat	**0.832**	[0.892, 1.116]

MSQ values based on conditional calculations (*n* = 754 complete cases).

Simulation based thresholds from 200 simulated datasets.

Bold indicates item misfit.

**Fig. 4:**
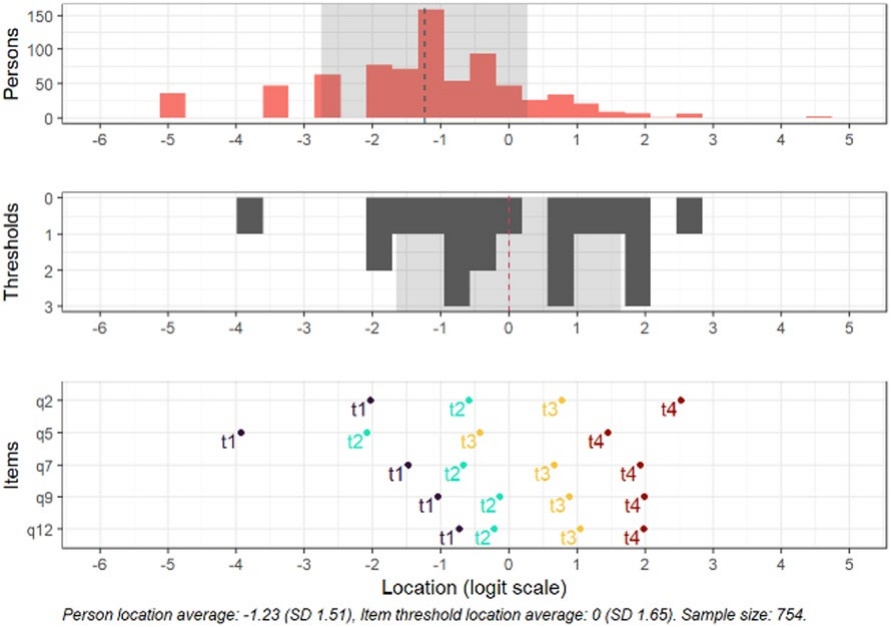
Targeting for the subscale avoidance. Separately shown for persons, thresholds and items. Person location average: −1.23 (SD 1.51), Item threshold location average: 0 (SD 1.65). Sample size: 754.

All items were congruent to the predefined scale steps, and showed decent distances between response category thresholds (see item locations, Fig. 4).

In this subscale, there was no significant DIF observed for the variables sex, age, relationship status, education or immigration status (all logit values < 0.5). There was also no significant DIF for the interaction between relationship status and sex.

#### Attention subscale

The 5-item attention subscale showed some issues with scale dimensionality. Two items showed problems with conditional item infit, with item 1 being slightly overfit and item 6 being underfit (see Table 4). Residual correlations were observed (relative cut-off r: 0.002) between item 3 and 4 (r: 0.3). The CLRT for the full model showed statistically significant misfit (*p* < 0.001) and the bootstrap calculation, restraining the sample size to 400, showed statistically significant misfit for 99.4% of the iterations. Based on loadings on first residual contrast, items 3 and 4 cluster together while items 1, 6 and 8 cluster together.

**Fig. 5:**
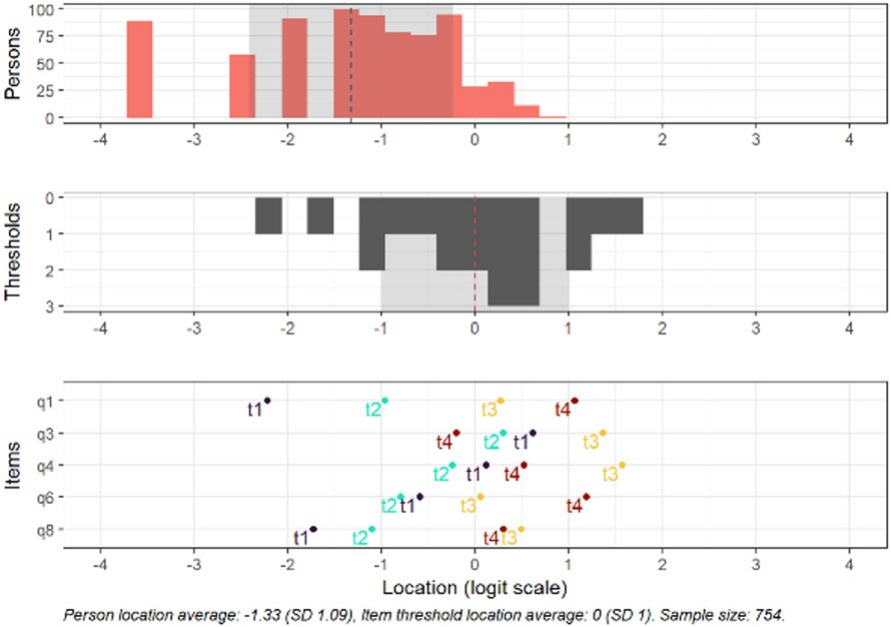
Targeting for the subscale attention. Separately shown for persons, thresholds and items. Person location average: -1.33 (SD 1.09), Item threshold location average: 0 (SD 1). Sample size: 754.

Regarding ceiling and floor effects, 89 (11.8%) respondents endorsed a minimum score on this subscale and 1 (0.1%) endorsed a maximum score. Based on the logit scale, 0% of respondents had person locations above the highest item threshold (1.57) and 19.5% had locations below the lowest item threshold (-2.22).

In the subscale attention, all items but item 1 demonstrated disordered response category thresholds (see item locations, Fig. 5).

In this subscale, there was no significant DIF observed for the variables sex, age, relationship status, education or immigration status (all logit values < 0.5). There was also no significant DIF for the interaction between relationship status and sex.

**Table 4:** Attention subscale, conditional item infit.

Item		InfitMSQ	Infit thresholds
1	I pay attention to my heart beat	**0.802**	[0.904, 1.103]
3	My racing heart wakes me up at night	0.904	[0.827, 1.15]
4	Chest pain/discomfort wakes me up at night	1.105	[0.893, 1.163]
6	I check my pulse	**1.306**	[0.877, 1.143]
8	I can feel my heart in my chest	0.967	[0.9, 1.112]

MSQ values based on conditional calculations (*n* = 754 complete cases). Simulation based thresholds from 200 simulated datasets.

Bold indicates item misfit.

#### Dragioti et al. (2011), 10-item version

Analysis of this fear subscale (consisting of the items 14, 15, 16, and 17), found item 17 to be underfit (MSQ: 1.488; thresholds: 0.901, 1.129), and residual correlations (relative cut-off r: -0.157) between items 15 and 16 (r: 0.03). Item 17 did not discriminate between the two highest response categories. No significant levels of DIF were observed.

The avoidance subscale (consisting of the items 2, 7, and 12), showed item 12 to be underfit (MSQ: 1.136; thresholds: 0.915, 1.198), and there were no residual correlations above the relative cut-off. Response categories were properly ordered and separated from eachother. DIF was observed for item 7 in terms of age (logits: 0.576), where older participants (> 74 years) did not discriminate between “never” and “rarely”, nor “often” and “always”.

This version of the subscale attention (consisting of the items 3, 4, and 8), found item 8 to be underfit (MSQ: 1.318; thresholds: 0.900, 1.092) and item 3 overfit (MSQ: 0.702; thresholds: 0.866, 1.148). Residual correlations (relative cut-off r: -0.104) were identified between item 3 and 4 (r: 0.11). Each item demonstrated disordered response categories. No significant levels DIF were found.

#### 4-factor version with reassurance seeking

The reassurance seeking scale (consisting of the items 11, 17 and 18 from the fear subscale), showed item misfit for item 11 (MSQ: 1.247; thresholds: 0.909, 1.144) and item 18 (MSQ: 0.765; thresholds: 0.914, 1.098), and a residual correlation (relative cut-off r: -0.211) between item 17 and 18 (r: -0.08). To achieve adequate ordering of response categories, the three middle categories (1,2,3) of item 11 was collapsed into one, the two highest categories for item 17 (3,4) and the middle and the second highest category for item 18 (2,3). No significant DIF was observed.

### Confirmatory factor analysis

The CFA, using WLSMV estimator and dynamic cut-off values, showed inadequate fit for all three subscales from the original 18-item version of the CAQ (see Table 5), when analysed as separate unidimensional scales.

**Table 5:** Confirmatory factor model fit indices.

	SRMR	RMSEA	CFI
**Fear**			
Level 0	0.025	0.026	0.998
Level 1	0.031	0.042	0.994
Level 2	0.042	0.067	0.986
Empirical fit indices	0.082	0.167	0.927
**Avoidance**			
Level 0	0.019	0.04	0.998
Level 1	0.026	0.065	0.996
Level 2	0.033	0.094	0.992
Empirical fit indices	0.03	0.136	0.984
**Attention**			
Level 0	0.028	0.04	0.994
Level 1	0.031	0.051	0.991
Level 2	0.045	0.088	0.98
Empirical fit indices	0.138	0.245	0.852

Empirical values are based on observed data. Level 0: 1/3 of items have a residual correlation with one other item, Level 1: 2/3 of items have a residual correlation with one other item, Level 2: Every item has a residual correlation with one other item in the data.

### Scale adjustments

Subsequent iterative Rasch analyses indicated that none of the subscales could demonstrate an item set larger than two items that had no substantive psychometric problems. In the section below, we report revised versions of each scale, with a minimum of 3 items, and briefly describe the remaining issues.

The revised fear subscale consisted of 3 items (14, 15 and 16) that showed problems with misfit for item 14 (MSQ: 1.158, thresholds: 0.898, 1.091) and item 16 (0.885, thresholds: 0.906, 1.102), and a residual correlation (relative cut-off: -0.331) between item 15 and 16 (r: -0.29). No significant levels of DIF were observed and the response categories were clearly separated and properly ordered.

After iterative analyses and adjustments, the revised avoidance subscale consisted of 4 items (2, 7, 9 and 12) that showed no problems with item misfit, but residual correlations (relative cut-off r: -0.171) between the two items pairs 2 and 7 (r: -0.1) and 9 and 12 (r: -0.13). There was no DIF for any of the items and response categories were clearly separated and properly ordered.

Adjustments to the attention subscale resulted in a revised version with 3 items (1, 3 and 8) that had no problems with item misfit, residual correlations or significant DIF. To achieve adequate ordering of response categories, the three middle categories (1, 2, 3) of item 3 was collapsed into one, and the two highest categories (3, 4) of item 8 was collapsed into one.

## Discussion

In this study we aimed to re-evaluate the psychometric properties of the CAQ in patients after an MI, using both a WLSMV estimator and simulation-based cut-offs for CFA, and Rasch analysis. In both cases, the questionnaire data achieved poor model fit. Additionally, the fourth factor, reassurance seeking, was tested, as well as the 10-item version of the CAQ proposed by Dragioti et al.^14^. Iterative analyses were conducted to identify modified item sets with adequate psychometric properties. However, no subscale with more than two items could be constructed that fit the Rasch model.

The original version of the CAQ demonstrated issues across all domains of the Rasch analysis. The results regarding item fit indicated that that neither of the scales were unidimensional. Although factor analysis also investigates dimensionality, Rasch analysis is stricter as it also considers patterns in the residuals^34^. Local dependency indicates that item responses covary in ways that are not related to the latent trait, and leads to an artificially elevated reliability^34,49^. For example, analyses of the fear subscale revealed residual correlations between items 14–16 and 10 and 13. The relation between items 14–16 likely reflect semantic overlap (all beginning with “When I have chest discomfort, or when my heart is beating fast…”), while correlations between items 10 (“If tests come out normal, I still worry about my heart”) and 13 (“I worry that doctors do not believe my chest pain/discomfort is real”) may indicate a distinct latent trait, possibly related to “cardiac disease conviction”, as conceptualized in the first 16-item version of the CAQ^7^. Additionally, in the avoidance subscale, item 5 (“I take it easy as much as possible”) was notably underfitting, meaning responses on this item was too unpredictable in relation to the latent trait. This item does not explicitly refer to anxiety and is arguably too vague, tapping into some other aspects related to physical activity.

Important for questionnaires with polytomous data, is that the different scale steps are ordered as intended. A high score on a certain item should correspond to a higher score on the latent trait that this item aims to capture, and vice versa. While some items demonstrated ordered response categories, such as the items in the avoidance scale, others clearly did not. Either they were disordered, or the distance between them was too small to effectively discriminate between scale steps. One example is item 11 (“I feel safe being around a hospital, physician, or other medical facility”). A problem with this item is that it does not explicitly assess anxiety. High endorsement may reflect general attitudes toward safety rather than anxiety-related reassurance seeking.

Items should also to a large extent be unbiased in relation to different groups. If an item is biased and an item has a different response pattern based on group differences, this is referred to as DIF^34^. The CAQ functioned well across different groups in this study, although some of the groups were small which makes results less reliable. The only observed significant DIF was in item 18 (“When I have chest discomfort, or when my heart is beating fast, I tell my family and friends”) for male participants based on relationship status. Why men tend to tell their family and friends to a greater extent when they are in a relationship may be due them relying more on their partners for social support, but more research would be needed to confirm this hypothesis.

In addition to testing the original model, the three factors in the 10-item version of the CAQ proposed by Dragioti et al.^14^, and the reassurance seeking subscale, as proposed by previous literature^9–11,15^, were tested. The factors proposed by Dragioti et al.^14^ were similar to the revised scales based on iterative analyses, with slight variations. However, these variations still showed problems with item misfit, local dependency, disordered response categories, and DIF, suggesting it is not a reliable scale. Considering the reassurance seeking subscale, there was also an indication for this subscale when analysing the fear subscale, as these items (11, 17, and 18) clustered together on the 1st contrast loadings. However, item 11 is clearly problematic, as it has very poor discriminative ability, and the semantic similarity between item 17 and 18 (both beginning with “When I have chest discomfort, or when my heart is beating fast…”), which is not shared with item 11, also likely creates unwanted local dependency.

Finally, a CFA based on an WLSMV estimator and using dynamic cut-off indices was conducted to provide results following similar principles as the Rasch analysis but that are familiar to readers that are used to CTT approaches. The empirical fit indices for the original model did not show acceptable fit, either by means of the traditional cut-off values or the simulated levels of misspecification, which is in line with the results provided by the Rasch analysis.

### Practical implications

The psychometric properties of the CAQ identified in this paper indicate that the subscales of the CAQ are not functioning as intended, capturing unintended noise, bias and measurement errors.

Despite the issues with the CAQ identified in this paper, these do not completely discredit the validity of the questionnaire. As the scale has been available for 25 years, it has been used both for assessing treatment outcomes^54–56^ and predicting cardiovascular risk^57–59^, in a range of CVDs. Furthermore, scales developed under CTT and MTT have in some cases been found to correlate above 0.85, indicating scores could be comparable to some extent^19^. This indicates that while there are many aspects of the CAQ that could be improved to increase construct specificity and psychometric robustness, the current version might still be useful as the best option.

Although our recommendations are to revise the questionnaire to improve its reliability, no other questionnaire of CA exists to date that has been validated using MTT. Another questionnaire, that perhaps taps into CA, is the cardiac distress inventory which has been developed using Rasch analysis^60^. However, there are some limitations with the methodology, for example as they are using rule-of-thumb cut-off values instead of the simulation-based approach. That questionnaire has also not yet been validated in Swedish. As such, the current CAQ might be the best option for anyone interested in assessing CA, for lack of better alternatives. However, we advise that the results be interpreted with caution and knowing that the scale is lacking in several aspects regarding its reliability.

### Potential improvements for the CAQ

Building on the point of estimation of treatment effects and risk prediction, a key limitation of the CAQ is that it is lacking valid cut-off values for clinical decision making. There are several diagnoses in the DSM-5 that correspond to CA (including PD, IAD, OCD, SSD, as well as anxiety disorder due to another medical condition), that could be used to validate a clinically relevant cut-off, and a revised CAQ with increased specificity and reliability might be able to distinguish between different symptom patterns relating to these disorders. And as previous publications have offered both relative and absolute risk estimates for scores on the CAQ, AUC-analyses could also be used to indicate optimal cut-off values for cardiovascular risk prediction.

When considering content validity, items in both the attention and avoidance subscales lack specificity to anxiety. For example, item 6 “I check my pulse” could be revised to “I check my pulse to make sure nothing is wrong” to better reflect anxiety-driven hypervigilance. Similarly, item 11 “I feel safe being around a hospital, physician, or other medical facility” could be rephrased to “Being far from a hospital, physician, or other medical facility makes me anxious”, to better capture the intended trait. Furthermore, item 5 “I take it easy as much as possible” could better reflect anxious avoidance by phrasing it like “If I don’t take it easy, I get anxious”. Another way of increasing specificity would be by focusing on fear-based beliefs, for example as seen in an a kinesiophobia scale, e.g., “I am afraid I might injure myself during physical activity/exercising”^61^.

Issues of local dependency in the fear subscale (and potential reassurance seeking subscale) could be solved by reconsidering the phrasing and combination of items 10, 11, and 13–18, to make sure they either all follow the same phrasing or that they only consist of items without apparent semantic similarities.

When considering the problems with disordered thresholds, the content and category labels of these items could be changed to better reflect an increasing level of the latent trait. It is also possible to combine the different scale steps, as we did in the revised versions of the scale. However, with fewer scale steps, reliability is typically lower and this might also have to be addressed. Finally, it is possible to consider whether these items at all belong to the CAQ.

### Limitations

The sample consists of post-MI patients with preserved cardiac function. This group is rather homogenous, with a large proportion of male participants, being generally healthier than the typical MI-patient, and on average having low levels of CA. This may have led to a narrower distribution of responses and affected the analyses regarding person separation reliability, targeting, and item difficulty. Although the psychometric properties of the CAQ have been evaluated in both cardiac and non-cardiac populations using CTT, more research is needed to see whether the scales are biased based on disease group using Rasch analysis.

Furthermore, these analyses are based on the Swedish translation of the, limiting the generalisability to other translations. However, this is currently the only analysis performed on the CAQ using Rasch analysis and there are no studies on the versions of the other translations proving that the questionnaire fits the Rasch model. Thus, more research is warranted, analysing the psychometric properties of the CAQ in other languages than Swedish, using Rasch analysis.

## Conclusion

The original version of the CAQ did not fit the Rasch model, disputing the reliability of the scale. Neither subscale proved to be unidimensional, and several items had disordered response categories. Iterative analyses of different combinations of items could not find a subscale with more than two items showed acceptable fit to the Rasch Model. The 10-item version by Dragioti et al. (2011) did also not fit the Rasch model, nor did the factor reassurance seeking. Based on these findings, it is advisable to revise the CAQ to improve its validity and reliability.

## Supplementary Information

Below is the link to the electronic supplementary material.

Supplementary Material 1

## Data Availability

The datasets generated and/or analysed during the current study are not publicly available due data protection regulation but are available from the corresponding author on reasonable request.
