# Supplementary material for: Psychometric properties of the Swedish cardiac anxiety questionnaire: a Rasch analysis
Source: Sci Rep. 2025 Nov 24;15:41834. doi: 10.1038/s41598-025-28073-8 (PMC12647126; doi:10.1038/s41598-025-28073-8)
Supplement: Supplementary file 1 — Supplementary Material 1 [file 41598_2025_28073_MOESM1_ESM.zip › Supplementary/analysis_att.html]

CAQ psychometric analysis, attention subscale


# CAQ psychometric analysis, attention subscale

 Code

- Show All Code
- Hide All Code
- ---
- View Source

Using Rasch Measurement Theory

Author

Affiliation

Magnus Johansson; Philip Leissner

RISE Research Institutes of Sweden; Department of women’s and children’s health, Uppsala University

## Table of contents

- 1 All items in the analysis
- 2 Descriptives - item level
- 3 Attention subscale
  - 3.1 Sumscore
  - 3.2 Rasch analysis 1
  - 3.3 Attention without items q3 and q4
  - 3.4 Test Information (Reliability)
  - 3.5 Attention subscale without item q6
  - 3.6 Attention without items q4 and q6
  - 3.7 Test Information (Reliability)

## Other Formats

- PDF

Code

```
# one package below requires that you use devtools to install them manually:
# first install devtools by
# install.packages('devtools')

library(easyRasch) # devtools::install_github("pgmj/easyRasch")
library(grateful)
library(ggrepel)
library(car)
library(kableExtra)
library(readxl)
library(tidyverse)
library(eRm)
library(iarm)
library(mirt)
library(psych)
library(psychotree)
library(matrixStats)
library(reshape)
library(knitr)
library(patchwork)
library(formattable) 
library(glue)
library(readxl) # for reading excel files

### optional libraries
#library(TAM)
#library(skimr)
#library(janitor)

### some commands exist in multiple packages, here we define preferred ones that are frequently used
select <- dplyr::select
count <- dplyr::count
recode <- car::recode
rename <- dplyr::rename
```

Code

```
### import data - this is just sample code, the files do not exist
df <- read_excel("data/CAQ_Rasch.xlsx") # replace with your datafile as needed

#library(haven) # for SPSS and other formats
#library(labelled) # for getting labels and metadata from SPSS files

### Load item information
# make sure that variable names in df match with itemlabels$itemnr
iteminfo <- read_excel("data/iteminfo.xlsx")

itemlabels <- iteminfo[,1:2]

### Make a backup of the dataframe, in case you need to revert changes at some point
d <- df
```

Code

```
##### Optionally: filter participants based on missing data

##### Before filtering out participants, you should check the missing data structure using RImissing() and RImissingP()

RImissing(d)
```

RImissingP() needs to be addressed, so we’ll do a manual check:

Code

```
d[,6:23] %>% 
  mutate(missing = rowSums(is.na(.))) %>% 
  count(missing)
```

```
# A tibble: 10 × 2
   missing     n
     <dbl> <int>
 1       0   756
 2       1    28
 3       2     8
 4       3     1
 5       4     3
 6       5     2
 7       9     1
 8      10     1
 9      16     1
10      18     5
```

We lose 50 respondents if we remove everyone with a missing value on any item. 756 is a good sample size, so we’ll go with that.

Code

```
d <- na.omit(d)
```

2 who were missing on some demographic variable were also removed.

Code

```
#---- Create DIF variables----
  
# DIF variables into vectors, recoded as factors since DIF functions need this
# these could also be stored in its own dataframe (not a tibble) instead of as vectors

d_dif <- d %>% 
  mutate(sex = factor(SEX),
         age = AGE_R,
         rel_status = factor(RELSTAT_bin),
         born_swe = factor(BORNSWE),
         edu = factor(EDUCATION)) %>% 
  select(sex,age,rel_status,born_swe,edu)

# remove DIF variables from item data
d <- d %>% 
  select(starts_with("Item")) %>% 
  set_names(itemlabels$itemnr)


### label sex variable as factor
# dif.sex <- factor(dif.sex,
#                       levels = c(1,2,3),
#                       labels = c("Female", "Male", "Other/missing response"))

# optionally, load RISE ggplot theme and color palettes and set the theme as default.
# just comment out the row below if you desire different theming
source("RISE_theme.R")
```

## 1 All items in the analysis

Code

```
RIlistitems(d)
```

| itemnr | item |
| --- | --- |
| q1 | I pay attention to my heart beat |
| q2 | I avoid physical exertion |
| q3 | My racing heart wakes me up at night |
| q4 | Chest pain/discomfort wakes me up at night |
| q5 | I take it easy as much as possible |
| q6 | I check my pulse |
| q7 | I avoid exercise or other physical work |
| q8 | I can feel my heart in my chest |
| q9 | I avoid activities that make my heart beat faster |
| q10 | If tests come out normal, I still worry about my heart |
| q11 | I feel safe being around a hospital, physician, or other medical facility |
| q12 | I avoid activities that make me sweat |
| q13 | I worry that doctors do not believe my chest pain/discomfort is real |
| q14 | When I have chest discomfort or I feel my heart is beating fast I worry that I may have a heart attack |
| q15 | When I have chest discomfort or I feel my heart is beating fast I have difficulty concentrating on anything else |
| q16 | When I have chest discomfort or I feel my heart is beating fast I get frightened |
| q17 | When I have chest discomfort or I feel my heart is beating fast I like to be checked out by a doctor |
| q18 | When I have chest discomfort or I feel my heart is beating fast I tell my family or friends |

Response distribution for all items are summarized below.

Code

```
RIallresp(d)
```

| Response category | Number of responses | Percent |
| --- | --- | --- |
| 0 | 5359 | 39.5 |
| 1 | 3580 | 26.4 |
| 2 | 2767 | 20.4 |
| 3 | 1244 | 9.2 |
| 4 | 622 | 4.6 |

## 2 Descriptives - item level

Code

```
RIlistItemsMargin(d, fontsize = 12)
```

| itemnr | item |
| --- | --- |
| q1 | I pay attention to my heart beat |
| q2 | I avoid physical exertion |
| q3 | My racing heart wakes me up at night |
| q4 | Chest pain/discomfort wakes me up at night |
| q5 | I take it easy as much as possible |
| q6 | I check my pulse |
| q7 | I avoid exercise or other physical work |
| q8 | I can feel my heart in my chest |
| q9 | I avoid activities that make my heart beat faster |
| q10 | If tests come out normal, I still worry about my heart |
| q11 | I feel safe being around a hospital, physician, or other medical facility |
| q12 | I avoid activities that make me sweat |
| q13 | I worry that doctors do not believe my chest pain/discomfort is real |
| q14 | When I have chest discomfort or I feel my heart is beating fast I worry that I may have a heart attack |
| q15 | When I have chest discomfort or I feel my heart is beating fast I have difficulty concentrating on anything else |
| q16 | When I have chest discomfort or I feel my heart is beating fast I get frightened |
| q17 | When I have chest discomfort or I feel my heart is beating fast I like to be checked out by a doctor |
| q18 | When I have chest discomfort or I feel my heart is beating fast I tell my family or friends |

- Tile plot
- Stacked bars
- Barplots

Code

```
RItileplot(d)
```

Code

```
RIbarstack(d) + scale_fill_viridis_d(labels = c("Always","Often","Sometimes","Rarely","Never"), direction = -1)
```

Code

```
RIbarplot(d)
```

Very few individuals endorse the highest categories of items q3, q4, q10, and q13. Over half of the participants endorse the lowest category for items q3, q4, and q13.

## 3 Attention subscale

Code

```
d_all <- d

items_att <- iteminfo %>% 
  filter(factor == "attention") %>% 
  pull(itemnr)

d <- d_all %>% 
  select(all_of(items_att))
```

### 3.1 Sumscore

Code

```
d_sum <- d %>%
  mutate(total = rowSums(select(., starts_with("q")), na.rm = TRUE))
d_sum <- d_sum["total"]
```

Code

```
total_freq <- as.data.frame(table(d_sum$total))
colnames(total_freq) <- c("Total", "N")
total_freq$Percent <- round(100 * total_freq$N / sum(total_freq$N), 1)
```

Code

```
RIallresp(d_sum)
```

| Response category | Number of responses | Percent |
| --- | --- | --- |
| 0 | 89 | 11.8 |
| 1 | 58 | 7.7 |
| 2 | 91 | 12.1 |
| 3 | 100 | 13.3 |
| 4 | 94 | 12.5 |
| 5 | 78 | 10.3 |
| 6 | 76 | 10.1 |
| 7 | 45 | 6.0 |
| 8 | 50 | 6.6 |
| 9 | 28 | 3.7 |
| 10 | 22 | 2.9 |
| 11 | 11 | 1.5 |
| 12 | 8 | 1.1 |
| 13 | 3 | 0.4 |
| 14 | 1 | 0.1 |

### 3.2 Rasch analysis 1

The eRm package, which uses Conditional Maximum Likelihood (CML) estimation, will be used primarily. For this analysis, the Partial Credit Model will be used.

| itemnr | item |
| --- | --- |
| q1 | I pay attention to my heart beat |
| q3 | My racing heart wakes me up at night |
| q4 | Chest pain/discomfort wakes me up at night |
| q6 | I check my pulse |
| q8 | I can feel my heart in my chest |

- Conditional item fit
- Item-restscore
- Conditional LRT
- Local dependency
- Residual correlations
- 1st contrast loadings
- Response categories
- Targeting
- Item hierarchy
- Score groups LR-test
- Score groups obs-exp
- Rasch-tree DIF immigration status
- Rasch-tree DIF sex
- Rasch-tree DIF age
- Rasch-tree DIF relationship and sex
- Person fit
- Floor and ceiling effects

Code

```
#RIitemfit(d, cutoff = "Smith98")

simfit1 <- RIgetfit(d, iterations = 200, cpu = 8) 

RIitemfit(d, simfit1)
```

| Item | InfitMSQ | Infit thresholds | OutfitMSQ | Outfit thresholds | Infit diff | Outfit diff | Relative location |
| --- | --- | --- | --- | --- | --- | --- | --- |
| q1 | 0.802 | [0.906, 1.099] | 0.818 | [0.914, 1.09] | 0.104 | 0.096 | 0.87 |
| q3 | 0.904 | [0.829, 1.147] | 0.708 | [0.757, 1.284] | no misfit | 0.049 | 1.85 |
| q4 | 1.105 | [0.88, 1.152] | 1.381 | [0.83, 1.205] | no misfit | 0.176 | 1.82 |
| q6 | 1.306 | [0.883, 1.157] | 1.309 | [0.888, 1.172] | 0.149 | 0.137 | 1.30 |
| q8 | 0.967 | [0.884, 1.136] | 0.977 | [0.89, 1.118] | no misfit | no misfit | 0.82 |
|  |
| --- |
| Note: |
| MSQ values based on conditional calculations (n = 754 complete cases).  Simulation based thresholds from 200 simulated datasets. |

Code

```
RIgetfitPlot(simfit1, d)
```

Code

```
RIrestscore(d)
```

| Item | Observed value | Model expected value | Absolute difference | Adjusted p-value (BH) | Statistical significance level | Location | Relative location |
| --- | --- | --- | --- | --- | --- | --- | --- |
| q1 | 0.60 | 0.47 | 0.13 | 0.000 | \*\*\* | -0.46 | 0.87 |
| q3 | 0.60 | 0.43 | 0.17 | 0.000 | \*\*\* | 0.52 | 1.85 |
| q4 | 0.38 | 0.44 | 0.06 | 0.214 |  | 0.49 | 1.82 |
| q6 | 0.34 | 0.47 | 0.13 | 0.000 | \*\*\* | -0.04 | 1.30 |
| q8 | 0.50 | 0.48 | 0.02 | 0.398 |  | -0.51 | 0.82 |

Code

```
clr_tests(d, model = "PCM")
```

```
Conditional Likelihood Ratio Tests:
```

```
        clr df pvalue  sig 
overall 101 19 3.5e-13  ***
```

Code

```
RIbootLRT(d, iterations = 1000, samplesize = 400, cpu = 8)
```

| Result | n | Percent |
| --- | --- | --- |
| Not statistically significant | 5 | 0.5 |
| Statistically significant | 995 | 99.5 |

Code

```
# using partial gamma LD from library(iarm)
RIpartgamLD(d)
```

| Item 1 | Item 2 | Partial gamma | SE | Lower CI | Upper CI | Adjusted p-value (BH) |
| --- | --- | --- | --- | --- | --- | --- |
| q3 | q4 | 0.766 | 0.040 | 0.687 | 0.844 | 0 |
| q4 | q3 | 0.689 | 0.046 | 0.598 | 0.779 | 0 |
| q1 | q6 | 0.399 | 0.058 | 0.286 | 0.512 | 0 |
| q1 | q8 | 0.323 | 0.061 | 0.203 | 0.442 | 0 |

Code

```
simcor1 <- RIgetResidCor(d, iterations = 250, cpu = 8)
RIresidcorr(d, cutoff = simcor1$p99)
```

|  | q1 | q3 | q4 | q6 | q8 |
| --- | --- | --- | --- | --- | --- |
| q1 |  |  |  |  |  |
| q3 | -0.14 |  |  |  |  |
| q4 | -0.21 | 0.3 |  |  |  |
| q6 | -0.04 | -0.37 | -0.43 |  |  |
| q8 | -0.03 | -0.11 | -0.23 | -0.26 |  |
|  |
| --- |
| Note: |
| Relative cut-off value is 0.003, which is 0.155 above the average correlation (-0.152).  Correlations above the cut-off are highlighted in red text. |

Code

```
RIloadLoc(d)
```

Code

```
mirt(d, model=1, itemtype='Rasch', verbose = FALSE) %>% 
  plot(type="trace", as.table = TRUE, 
       theta_lim = c(-6,6))
```

Code

```
# for fewer items or a more magnified figure, use:
#RIitemCats(d)
```

Code

```
# increase fig-height above as needed, if you have many items
RItargeting(d)
```

Code

```
RIitemHierarchy(d)
```

Code

```
iarm::score_groups(as.data.frame(d)) %>% 
  as.data.frame(nm = "score_group") %>% 
  dplyr::count(score_group)
```

```
  score_group   n
1           1 432
2           2 322
```

Code

```
dif_plots <- d %>% 
  add_column(dif = iarm::score_groups(.)) %>% 
  split(.$dif) %>% # split the data using the DIF variable
  map(~ RItileplot(.x %>% dplyr::select(!dif)) + labs(title = .x$dif))
dif_plots[[1]] + dif_plots[[2]]
```

Code

```
clr_tests(d, model = "PCM")
```

```
Conditional Likelihood Ratio Tests:
```

```
        clr df pvalue  sig 
overall 101 19 3.5e-13  ***
```

Code

```
item_obsexp(PCM(d))
```

```
Score group 1: 
   mean obs mean exp std.res sig
q1  0.9475   1.0432  -2.5794 -  
q3  0.0525   0.1060  -2.9864 -  
q4  0.2449   0.1890   2.3340 +  
q6  0.5190   0.4198   2.9182 +  
q8  0.9067   0.9119  -0.1351    

Score group 2: 
   mean obs mean exp std.res sig
q1  2.102    2.001    2.193  +  
q3  0.618    0.560    1.317     
q4  0.807    0.867   -1.244     
q6  1.590    1.696   -1.943     
q8  2.034    2.029    0.111
```

Code

```
RIdifTable(d, d_dif$born_swe)
```

```
[1] "No statistically significant DIF found."
```

Code

```
RIdifTable(d, d_dif$sex)
```

| Item | 2 | 3 | Mean location | StDev | MaxDiff |
| --- | --- | --- | --- | --- | --- |
| q1 | -0.542 | -0.455 | -0.499 | 0.061 | 0.087 |
| q3 | 0.451 | 0.547 | 0.499 | 0.068 | 0.096 |
| q4 | 0.328 | 0.542 | 0.435 | 0.152 | 0.214 |
| q6 | 0.363 | -0.135 | 0.114 | 0.352 | 0.498 |
| q8 | -0.600 | -0.500 | -0.550 | 0.071 | 0.100 |

Code

```
RIdifTable(d, d_dif$age)
```

```
[1] "No statistically significant DIF found."
```

Code

```
RIdifTable2(d, d_dif$rel_status, d_dif$sex)
```

| Item | 2 | 3 | Mean location | StDev | MaxDiff |
| --- | --- | --- | --- | --- | --- |
| q1 | -0.542 | -0.455 | -0.499 | 0.061 | 0.087 |
| q3 | 0.451 | 0.547 | 0.499 | 0.068 | 0.096 |
| q4 | 0.328 | 0.542 | 0.435 | 0.152 | 0.214 |
| q6 | 0.363 | -0.135 | 0.114 | 0.352 | 0.498 |
| q8 | -0.600 | -0.500 | -0.550 | 0.071 | 0.100 |

Code

```
RIpfit(d)
```

Code

```
RItif(d, samplePSI = T, cutoff = 1)
```

Item q6 is underfit, indicating it deviates from the dimension. Item q1 is overfit.

Strong residual correlation between items q3 and q4.

q3 Heart palpitations wake me up at night q4 Chest pain or discomfort wakes me up at night

Not so surprising when both are about being woken up at night, and besides, “heart palpitations” are a form of “chest discomfort”.

All items except q1 have disordered response thresholds.

No significant DIF is observed.

Based on inconsistencies of items q3 and q4 in previous studies and lacking face validity of these items reflecting the cognitive aspect of “attention”, we will start by removing these and see how the dimensionality problems with item q6 develop.

### 3.3 Attention without items q3 and q4

Code

```
d$q3 <- NULL
d$q4 <- NULL
```

- Conditional item fit
- Item-restscore
- Residual correlations
- Local dependency
- 1st contrast loadings
- Score groups obs-exp
- Partial gamma DIF sex

Code

```
simfit1 <- RIgetfit(d, iterations = 200, cpu = 8) 

RIitemfit(d, simfit1)
```

| Item | InfitMSQ | Infit thresholds | OutfitMSQ | Outfit thresholds | Infit diff | Outfit diff | Relative location |
| --- | --- | --- | --- | --- | --- | --- | --- |
| q1 | 0.818 | [0.907, 1.083] | 0.829 | [0.915, 1.08] | 0.089 | 0.086 | 0.93 |
| q6 | 1.146 | [0.903, 1.143] | 1.185 | [0.866, 1.114] | 0.003 | 0.071 | 1.40 |
| q8 | 1.069 | [0.893, 1.1] | 1.059 | [0.896, 1.102] | no misfit | no misfit | 0.88 |
|  |
| --- |
| Note: |
| MSQ values based on conditional calculations (n = 754 complete cases).  Simulation based thresholds from 200 simulated datasets. |

Code

```
RIgetfitPlot(simfit1, d)
```

Code

```
RIrestscore(d)
```

| Item | Observed value | Model expected value | Absolute difference | Adjusted p-value (BH) | Statistical significance level | Location | Relative location |
| --- | --- | --- | --- | --- | --- | --- | --- |
| q1 | 0.61 | 0.50 | 0.11 | 0.000 | \*\*\* | -0.14 | 0.93 |
| q6 | 0.43 | 0.49 | 0.06 | 0.124 |  | 0.33 | 1.40 |
| q8 | 0.48 | 0.50 | 0.02 | 0.675 |  | -0.19 | 0.88 |

Code

```
simcor1 <- RIgetResidCor(d, iterations = 250, cpu = 8)
RIresidcorr(d, cutoff = simcor1$p99)
```

|  | q1 | q6 | q8 |
| --- | --- | --- | --- |
| q1 |  |  |  |
| q6 | -0.26 |  |  |
| q8 | -0.16 | -0.52 |  |
|  |
| --- |
| Note: |
| Relative cut-off value is -0.21, which is 0.106 above the average correlation (-0.315).  Correlations above the cut-off are highlighted in red text. |

Code

```
# using partial gamma LD from library(iarm)
RIpartgamLD(d)
```

| Item 1 | Item 2 | Partial gamma | SE | Lower CI | Upper CI | Adjusted p-value (BH) |
| --- | --- | --- | --- | --- | --- | --- |
| q1 | q8 | 0.412 | 0.062 | 0.290 | 0.534 | 0 |
| q1 | q6 | 0.288 | 0.069 | 0.152 | 0.423 | 0 |

Code

```
RIloadLoc(d)
```

Code

```
item_obsexp(PCM(d))
```

```
Score group 1: 
   mean obs mean exp std.res sig
q1  0.915    0.995   -2.158  -  
q6  0.422    0.336    2.626  +  
q8  0.840    0.845   -0.134     

Score group 2: 
   mean obs mean exp std.res sig
q1  2.054    1.987    1.654     
q6  1.583    1.654   -1.562     
q8  2.014    2.010    0.101
```

Code

```
# using partial gamma from library(iarm)
RIpartgamDIF(d, d_dif$sex)
```

| Item | Partial gamma | SE | Lower CI | Upper CI | Adjusted p-value (BH) |
| --- | --- | --- | --- | --- | --- |
| q6 | 0.349 | 0.088 | 0.177 | 0.520 | 0.000 |
| q8 | -0.211 | 0.084 | -0.375 | -0.047 | 0.035 |

Item q6 is still underfit but not to the same extent. Item q1 is slightly overfit, similar to before.

Residual correlations are now identified between items q1 and q8. A lesser extent of local dependency can also be observed between items q1 and q6.

q1 I pay attention to my heart beat q6 I check my pulse q8 I can feel my heart in my chest

Items q1 and q6 may share a similarity in that they describe some kind of action, “paying attention” and “checking”. Items q1 and q8 both describe feeling and paying attention to the heart, which item q6 does not, and may explain the excessive correlation between these two items.

The response categories also need to be reviewed.

#### 3.3.1 Response categories

Code

```
mirt(d, model=1, itemtype='Rasch', verbose = FALSE) %>% 
  plot(type="trace", as.table = TRUE, 
       theta_lim = c(-6,6))
```

Code

```
RIitemHierarchy(d)
```

Item q1 displays well ordeded thresholds, but both items q6 and q8 have disordered response category thresholds.

As item q6 has problems with the two bottom categories we will combine these, and item q8 has problems with the two higher categories and so we will combine these.

Code

```
d %>% 
  mutate(q6 = car::recode(q6,"2=1;3=2;4=3"),
         q8 = car::recode(q8,"4=3")) %>% 
  RItileplot()
```

Code

```
d %>% 
  mutate(q6 = car::recode(q6,"2=1;3=2;4=3"),
         q8 = car::recode(q8,"4=3")) %>% 
  mirt(model=1, itemtype='Rasch', verbose = FALSE) %>% 
  plot(type="trace", as.table = TRUE, 
       theta_lim = c(-6,6))
```

Now all response categories look fine.

Code

```
d2 <- d %>% 
  mutate(q6 = car::recode(q6,"2=1;3=2;4=3"),
         q8 = car::recode(q8,"4=3"))
```

We can’t remove more items from the scale and the items that remain still have problems with q6 being underfit, and items q6 and q8 showing some local dependency with item q1.

Let’s still inspect the test information.

### 3.4 Test Information (Reliability)

Code

```
RItif(d2, samplePSI = T)
```

While the scale seems to have the highest amount of information around 0-1 on the latent scale, the test information is not above 3.33 on any part of the scale.

Remembering that q6 was strongly underfit in the first 5-item version and that we removed items q3 and q4 first, we could try testing the 4-item version, with items q3 and q4, but excluding item q6.

### 3.5 Attention subscale without item q6

Code

```
items_att <- iteminfo %>% 
  filter(factor == "attention") %>% 
  pull(itemnr)

d <- d_all %>% 
  select(all_of(items_att))
  
d$q6 <- NULL
```

- Conditional item fit
- Item-restscore
- Residual correlations
- Local dependency
- 1st contrast loadings
- Score groups obs-exp

Code

```
simfit1 <- RIgetfit(d, iterations = 200, cpu = 8) 

RIitemfit(d, simfit1)
```

| Item | InfitMSQ | Infit thresholds | OutfitMSQ | Outfit thresholds | Infit diff | Outfit diff | Relative location |
| --- | --- | --- | --- | --- | --- | --- | --- |
| q1 | 1.029 | [0.887, 1.118] | 1.029 | [0.903, 1.113] | no misfit | no misfit | 0.95 |
| q3 | 0.845 | [0.83, 1.179] | 0.622 | [0.75, 1.395] | no misfit | 0.128 | 2.11 |
| q4 | 1.071 | [0.852, 1.105] | 1.39 | [0.766, 1.208] | no misfit | 0.182 | 2.04 |
| q8 | 1.061 | [0.915, 1.115] | 1.046 | [0.926, 1.097] | no misfit | no misfit | 0.90 |
|  |
| --- |
| Note: |
| MSQ values based on conditional calculations (n = 754 complete cases).  Simulation based thresholds from 200 simulated datasets. |

Code

```
RIgetfitPlot(simfit1, d)
```

Code

```
RIrestscore(d)
```

| Item | Observed value | Model expected value | Absolute difference | Adjusted p-value (BH) | Statistical significance level | Location | Relative location |
| --- | --- | --- | --- | --- | --- | --- | --- |
| q1 | 0.52 | 0.53 | 0.01 | 0.782 |  | -0.55 | 0.95 |
| q3 | 0.70 | 0.50 | 0.20 | 0.000 | \*\*\* | 0.61 | 2.11 |
| q4 | 0.47 | 0.51 | 0.04 | 0.576 |  | 0.54 | 2.04 |
| q8 | 0.53 | 0.54 | 0.01 | 0.782 |  | -0.60 | 0.90 |

Code

```
simcor1 <- RIgetResidCor(d, iterations = 250, cpu = 8)
RIresidcorr(d, cutoff = simcor1$p99)
```

|  | q1 | q3 | q4 | q8 |
| --- | --- | --- | --- | --- |
| q1 |  |  |  |  |
| q3 | -0.25 |  |  |  |
| q4 | -0.34 | 0.16 |  |  |
| q8 | -0.12 | -0.26 | -0.42 |  |
|  |
| --- |
| Note: |
| Relative cut-off value is -0.068, which is 0.135 above the average correlation (-0.203).  Correlations above the cut-off are highlighted in red text. |

Code

```
# using partial gamma LD from library(iarm)
RIpartgamLD(d)
```

| Item 1 | Item 2 | Partial gamma | SE | Lower CI | Upper CI | Adjusted p-value (BH) |
| --- | --- | --- | --- | --- | --- | --- |
| q3 | q4 | 0.726 | 0.049 | 0.630 | 0.823 | 0 |
| q4 | q3 | 0.601 | 0.060 | 0.483 | 0.719 | 0 |
| q1 | q8 | 0.337 | 0.073 | 0.193 | 0.480 | 0 |
| q8 | q1 | 0.336 | 0.074 | 0.191 | 0.480 | 0 |

Code

```
RIloadLoc(d)
```

Code

```
item_obsexp(PCM(d))
```

```
Score group 1: 
   mean obs mean exp std.res sig
q1  0.9969   0.9807   0.4811    
q3  0.0343   0.0758  -2.7061 -  
q4  0.1776   0.1364   2.0091 +  
q8  0.8224   0.8381  -0.4576    

Score group 2: 
   mean obs mean exp std.res sig
q1  2.060    2.076   -0.379     
q3  0.622    0.582    1.007     
q4  0.867    0.907   -0.912     
q8  2.121    2.106    0.349
```

This version of the Attention subscale does not demonstrate any problems with item misfit. However, as expected, the problem with residual correlations between items q3 and q4 remains.

Let’s try a version without item q4.

### 3.6 Attention without items q4 and q6

Code

```
d$q4 <- NULL
```

- Conditional item fit
- Item-restscore
- Residual correlations
- Local dependency
- 1st contrast loadings
- Score groups obs-exp

Code

```
simfit1 <- RIgetfit(d, iterations = 200, cpu = 8) 

RIitemfit(d, simfit1)
```

| Item | InfitMSQ | Infit thresholds | OutfitMSQ | Outfit thresholds | Infit diff | Outfit diff | Relative location |
| --- | --- | --- | --- | --- | --- | --- | --- |
| q1 | 1.012 | [0.902, 1.072] | 1.024 | [0.911, 1.061] | no misfit | no misfit | 0.99 |
| q3 | 1.122 | [0.845, 1.166] | 1.047 | [0.766, 1.264] | no misfit | no misfit | 2.18 |
| q8 | 0.995 | [0.905, 1.076] | 1.024 | [0.912, 1.078] | no misfit | no misfit | 0.96 |
|  |
| --- |
| Note: |
| MSQ values based on conditional calculations (n = 754 complete cases).  Simulation based thresholds from 200 simulated datasets. |

Code

```
RIgetfitPlot(simfit1, d)
```

Code

```
RIrestscore(d)
```

| Item | Observed value | Model expected value | Absolute difference | Adjusted p-value (BH) | Statistical significance level | Location | Relative location |
| --- | --- | --- | --- | --- | --- | --- | --- |
| q1 | 0.57 | 0.57 | 0.00 | 0.949 |  | -0.39 | 0.99 |
| q3 | 0.56 | 0.52 | 0.04 | 0.949 |  | 0.80 | 2.18 |
| q8 | 0.58 | 0.57 | 0.01 | 0.949 |  | -0.42 | 0.96 |

Code

```
simcor1 <- RIgetResidCor(d, iterations = 250, cpu = 8)
RIresidcorr(d, cutoff = simcor1$p99)
```

|  | q1 | q3 | q8 |
| --- | --- | --- | --- |
| q1 |  |  |  |
| q3 | -0.26 |  |  |
| q8 | -0.34 | -0.29 |  |
|  |
| --- |
| Note: |
| Relative cut-off value is -0.169, which is 0.13 above the average correlation (-0.299).  Correlations above the cut-off are highlighted in red text. |

Code

```
# using partial gamma LD from library(iarm)
RIpartgamLD(d)
```

```
[1] "No statistically significant local dependency found."
```

Code

```
RIloadLoc(d)
```

Code

```
item_obsexp(PCM(d))
```

```
Score group 1: 
   mean obs mean exp std.res sig
q1  1.0771   1.0611   0.5303    
q3  0.0800   0.0762   0.2581    
q8  0.9057   0.9255  -0.6478    

Score group 2: 
   mean obs mean exp std.res sig
q1  2.193    2.213   -0.469     
q3  0.663    0.668   -0.108     
q8  2.277    2.253    0.556
```

Looks good in terms of dimensionality, let’s review the response thresholds again.

#### 3.6.1 Response categories

Code

```
mirt(d, model=1, itemtype='Rasch', verbose = FALSE) %>% 
  plot(type="trace", as.table = TRUE, 
       theta_lim = c(-6,6))
```

Code

```
RIitemHierarchy(d)
```

Like before, item q1 demonstrates well ordered thresholds, and item q8 is problematic in terms of the two higher thresholds. Item q3 also has very disordered thresholds.

Recoding of response categories.

Code

```
d %>% 
  mutate(q3 = car::recode(q3,"2=1;3=2;4=2"),
         q8 = car::recode(q8,"4=3")) %>% 
  RItileplot()
```

Code

```
d %>% 
  mutate(q3 = car::recode(q3,"2=1;3=2;4=2"),
         q8 = car::recode(q8,"4=3")) %>% 
  mirt(model=1, itemtype='Rasch', verbose = FALSE) %>% 
  plot(type="trace", as.table = TRUE, 
       theta_lim = c(-6,6))
```

Now, looks better again.

Code

```
d2 <- d %>% 
  mutate(q3 = car::recode(q3,"2=1;3=2;4=2"),
         q8 = car::recode(q8,"4=3"))
```

Now all that remains is to inspect the test information.

### 3.7 Test Information (Reliability)

Code

```
RItif(d2, samplePSI = T)
```

Similar to the other version of the scale, in spite of solving item misfit, residual correlations, and disordered response categories, the test information is still very low, indicating a low reliability of the scale.

## Reuse

CC BY 4.0

##### Source Code

```
---
title: "CAQ psychometric analysis, attention subscale"
subtitle: "Using Rasch Measurement Theory"
title-block-banner: "#009ca6"
title-block-banner-color: "#FFFFFF"
author: 
  name: Magnus Johansson; Philip Leissner
  affiliation: RISE Research Institutes of Sweden; Department of women's and children's health, Uppsala University
  affiliation-url: https://www.ri.se/en/kbm; https://www.uu.se/en/department/womens-and-childrens-health
  orcid: 0000-0003-1669-592X; 0000-0003-0787-9102
date-format: iso
always_allow_html: true
format: 
  html:
    toc: true
    toc-depth: 3
    toc-title: "Table of contents"
    embed-resources: true
    standalone: true
    page-layout: full
    mainfont: 'Lato'
    monofont: 'Roboto Mono'
    code-overflow: wrap
    code-fold: true
    code-tools: true
    code-link: true
    number-sections: true
    fig-dpi: 96
    layout-align: left
    linestretch: 1.6
    theme:
      - materia
      - custom.scss
    css: styles.css
    license: CC BY
  pdf:
    papersize: a4
    documentclass: report 
execute:
  echo: true
  warning: false
  message: false
  cache: true
editor_options: 
  markdown: 
    wrap: 72
  chunk_output_type: console
---

```{r}
#| label: setup

# one package below requires that you use devtools to install them manually:
# first install devtools by
# install.packages('devtools')

library(easyRasch) # devtools::install_github("pgmj/easyRasch")
library(grateful)
library(ggrepel)
library(car)
library(kableExtra)
library(readxl)
library(tidyverse)
library(eRm)
library(iarm)
library(mirt)
library(psych)
library(psychotree)
library(matrixStats)
library(reshape)
library(knitr)
library(patchwork)
library(formattable) 
library(glue)
library(readxl) # for reading excel files

### optional libraries
#library(TAM)
#library(skimr)
#library(janitor)

### some commands exist in multiple packages, here we define preferred ones that are frequently used
select <- dplyr::select
count <- dplyr::count
recode <- car::recode
rename <- dplyr::rename
```


```{r}
### import data - this is just sample code, the files do not exist
df <- read_excel("data/CAQ_Rasch.xlsx") # replace with your datafile as needed

#library(haven) # for SPSS and other formats
#library(labelled) # for getting labels and metadata from SPSS files

### Load item information
# make sure that variable names in df match with itemlabels$itemnr
iteminfo <- read_excel("data/iteminfo.xlsx")

itemlabels <- iteminfo[,1:2]

### Make a backup of the dataframe, in case you need to revert changes at some point
d <- df
```

```{r}
##### Optionally: filter participants based on missing data

##### Before filtering out participants, you should check the missing data structure using RImissing() and RImissingP()

RImissing(d)
```

RImissingP() needs to be addressed, so we'll do a manual check:

```{r}
d[,6:23] %>% 
  mutate(missing = rowSums(is.na(.))) %>% 
  count(missing)
```

We lose 50 respondents if we remove everyone with a missing value on any item. 756 is a good sample size, so we'll go with that.

```{r}
d <- na.omit(d)
```

2 who were missing on some demographic variable were also removed.

```{r}
#---- Create DIF variables----
  
# DIF variables into vectors, recoded as factors since DIF functions need this
# these could also be stored in its own dataframe (not a tibble) instead of as vectors

d_dif <- d %>% 
  mutate(sex = factor(SEX),
         age = AGE_R,
         rel_status = factor(RELSTAT_bin),
         born_swe = factor(BORNSWE),
         edu = factor(EDUCATION)) %>% 
  select(sex,age,rel_status,born_swe,edu)

# remove DIF variables from item data
d <- d %>% 
  select(starts_with("Item")) %>% 
  set_names(itemlabels$itemnr)


### label sex variable as factor
# dif.sex <- factor(dif.sex,
#                       levels = c(1,2,3),
#                       labels = c("Female", "Male", "Other/missing response"))

# optionally, load RISE ggplot theme and color palettes and set the theme as default.
# just comment out the row below if you desire different theming
source("RISE_theme.R")
```

## All items in the analysis
```{r}
RIlistitems(d)
```

Response distribution for all items are summarized below.

```{r}
#| tbl-cap: "Total number of responses for all items"
RIallresp(d)
```

## Descriptives - item level

```{r}
#| column: margin
RIlistItemsMargin(d, fontsize = 12)
```

::: panel-tabset
### Tile plot
```{r}
RItileplot(d)
```
### Stacked bars
```{r}
RIbarstack(d) + scale_fill_viridis_d(labels = c("Always","Often","Sometimes","Rarely","Never"), direction = -1)
```
### Barplots
```{r}
#| layout-ncol: 2
RIbarplot(d)
```
:::

Very few individuals endorse the highest categories of items q3, q4, q10, and q13. Over half of the participants endorse the lowest category for items q3, q4, and q13.


## Attention subscale

```{r}
d_all <- d

items_att <- iteminfo %>% 
  filter(factor == "attention") %>% 
  pull(itemnr)

d <- d_all %>% 
  select(all_of(items_att))
  
```

### Sumscore
```{r}
d_sum <- d %>%
  mutate(total = rowSums(select(., starts_with("q")), na.rm = TRUE))
d_sum <- d_sum["total"]
```

```{r}
total_freq <- as.data.frame(table(d_sum$total))
colnames(total_freq) <- c("Total", "N")
total_freq$Percent <- round(100 * total_freq$N / sum(total_freq$N), 1)
```

```{r}
RIallresp(d_sum)
```

### Rasch analysis 1

The eRm package, which uses Conditional Maximum Likelihood (CML)
estimation, will be used primarily. For this analysis, the Partial
Credit Model will be used.

```{r}
#| column: margin
#| echo: false
RIlistItemsMargin(d, fontsize = 13)
```

::: panel-tabset
#### Conditional item fit
```{r}
#RIitemfit(d, cutoff = "Smith98")

simfit1 <- RIgetfit(d, iterations = 200, cpu = 8) 

RIitemfit(d, simfit1)
RIgetfitPlot(simfit1, d)
```
#### Item-restscore
```{r}
RIrestscore(d)
```
#### Conditional LRT
```{r}
clr_tests(d, model = "PCM")

RIbootLRT(d, iterations = 1000, samplesize = 400, cpu = 8)
```
#### Local dependency
```{r}
# using partial gamma LD from library(iarm)
RIpartgamLD(d)
```

#### Residual correlations
```{r}
simcor1 <- RIgetResidCor(d, iterations = 250, cpu = 8)
RIresidcorr(d, cutoff = simcor1$p99)
```
#### 1st contrast loadings
```{r}
RIloadLoc(d)
```
#### Response categories
```{r}
mirt(d, model=1, itemtype='Rasch', verbose = FALSE) %>% 
  plot(type="trace", as.table = TRUE, 
       theta_lim = c(-6,6))
# for fewer items or a more magnified figure, use:
#RIitemCats(d)
```
#### Targeting
```{r}
#| fig-height: 5
# increase fig-height above as needed, if you have many items
RItargeting(d)
```
#### Item hierarchy
```{r}
#| fig-height: 5
RIitemHierarchy(d)
```
#### Score groups LR-test
```{r}
iarm::score_groups(as.data.frame(d)) %>% 
  as.data.frame(nm = "score_group") %>% 
  dplyr::count(score_group)

dif_plots <- d %>% 
  add_column(dif = iarm::score_groups(.)) %>% 
  split(.$dif) %>% # split the data using the DIF variable
  map(~ RItileplot(.x %>% dplyr::select(!dif)) + labs(title = .x$dif))
dif_plots[[1]] + dif_plots[[2]]

clr_tests(d, model = "PCM")
```
#### Score groups obs-exp
```{r}
item_obsexp(PCM(d))
```
#### Rasch-tree DIF immigration status
```{r}
RIdifTable(d, d_dif$born_swe)
```
#### Rasch-tree DIF sex
```{r}
RIdifTable(d, d_dif$sex)
```
#### Rasch-tree DIF age
```{r}
RIdifTable(d, d_dif$age)
```
#### Rasch-tree DIF relationship and sex
```{r}
RIdifTable2(d, d_dif$rel_status, d_dif$sex)
```
#### Person fit
```{r}
RIpfit(d)
```
#### Floor and ceiling effects
```{r}
RItif(d, samplePSI = T, cutoff = 1)
```
:::

Item q6 is underfit, indicating it deviates from the dimension. Item q1 is overfit.

Strong residual correlation between items q3 and q4.

q3 Heart palpitations wake me up at night 
q4 Chest pain or discomfort wakes me up at night 

Not so surprising when both are about being woken up at night, and besides, "heart palpitations" are a form of "chest discomfort".

All items except q1 have disordered response thresholds.

No significant DIF is observed.

Based on inconsistencies of items q3 and q4 in previous studies and lacking face validity of these items reflecting the cognitive aspect of "attention", we will start by removing these and see how the dimensionality problems with item q6 develop.

### Attention without items q3 and q4

```{r}
d$q3 <- NULL
d$q4 <- NULL
```


::: panel-tabset
#### Conditional item fit
```{r}
simfit1 <- RIgetfit(d, iterations = 200, cpu = 8) 

RIitemfit(d, simfit1)
RIgetfitPlot(simfit1, d)
```
#### Item-restscore
```{r}
RIrestscore(d)
```
#### Residual correlations
```{r}
simcor1 <- RIgetResidCor(d, iterations = 250, cpu = 8)
RIresidcorr(d, cutoff = simcor1$p99)
```
#### Local dependency
```{r}
# using partial gamma LD from library(iarm)
RIpartgamLD(d)
```
#### 1st contrast loadings
```{r}
RIloadLoc(d)
```
#### Score groups obs-exp
```{r}
item_obsexp(PCM(d))
```
#### Partial gamma DIF sex
```{r}
# using partial gamma from library(iarm)
RIpartgamDIF(d, d_dif$sex)
```
:::

Item q6 is still underfit but not to the same extent. Item q1 is slightly overfit, similar to before. 

Residual correlations are now identified between items q1 and q8. A lesser extent of local dependency can also be observed between items q1 and q6.

q1 I pay attention to my heart beat
q6 I check my pulse
q8 I can feel my heart in my chest

Items q1 and q6 may share a similarity in that they describe some kind of action, "paying attention" and "checking". Items q1 and q8 both describe feeling and paying attention to the heart, which item q6 does not, and may explain the excessive correlation between these two items.

The response categories also need to be reviewed.

#### Response categories
```{r}
mirt(d, model=1, itemtype='Rasch', verbose = FALSE) %>% 
  plot(type="trace", as.table = TRUE, 
       theta_lim = c(-6,6))

RIitemHierarchy(d)
```

Item q1 displays well ordeded thresholds, but both items q6 and q8 have disordered response category thresholds.

As item q6 has problems with the two bottom categories we will combine these, and item q8 has problems with the two higher categories and so we will combine these.

```{r}
d %>% 
  mutate(q6 = car::recode(q6,"2=1;3=2;4=3"),
         q8 = car::recode(q8,"4=3")) %>% 
  RItileplot()

d %>% 
  mutate(q6 = car::recode(q6,"2=1;3=2;4=3"),
         q8 = car::recode(q8,"4=3")) %>% 
  mirt(model=1, itemtype='Rasch', verbose = FALSE) %>% 
  plot(type="trace", as.table = TRUE, 
       theta_lim = c(-6,6))
```

Now all response categories look fine.

```{r}
d2 <- d %>% 
  mutate(q6 = car::recode(q6,"2=1;3=2;4=3"),
         q8 = car::recode(q8,"4=3")) 
```

We can't remove more items from the scale and the items that remain still have problems with q6 being underfit, and items q6 and q8 showing some local dependency with item q1.

Let's still inspect the test information.

### Test Information (Reliability)
```{r}
RItif(d2, samplePSI = T)
```

While the scale seems to have the highest amount of information around 0-1 on the latent scale, the test information is not above 3.33 on any part of the scale.

Remembering that q6 was strongly underfit in the first 5-item version and that we removed items q3 and q4 first, we could try testing the 4-item version, with items q3 and q4, but excluding item q6.

### Attention subscale without item q6

```{r}
items_att <- iteminfo %>% 
  filter(factor == "attention") %>% 
  pull(itemnr)

d <- d_all %>% 
  select(all_of(items_att))
  
d$q6 <- NULL
```

::: panel-tabset
#### Conditional item fit
```{r}
simfit1 <- RIgetfit(d, iterations = 200, cpu = 8) 

RIitemfit(d, simfit1)
RIgetfitPlot(simfit1, d)
```
#### Item-restscore
```{r}
RIrestscore(d)
```
#### Residual correlations
```{r}
simcor1 <- RIgetResidCor(d, iterations = 250, cpu = 8)
RIresidcorr(d, cutoff = simcor1$p99)
```
#### Local dependency
```{r}
# using partial gamma LD from library(iarm)
RIpartgamLD(d)
```
#### 1st contrast loadings
```{r}
RIloadLoc(d)
```
#### Score groups obs-exp
```{r}
item_obsexp(PCM(d))
```
:::

This version of the Attention subscale does not demonstrate any problems with item misfit. However, as expected, the problem with residual correlations between items q3 and q4 remains.

Let's try a version without item q4.

### Attention without items q4 and q6
```{r}
d$q4 <- NULL
```

::: panel-tabset
#### Conditional item fit
```{r}
simfit1 <- RIgetfit(d, iterations = 200, cpu = 8) 

RIitemfit(d, simfit1)
RIgetfitPlot(simfit1, d)
```
#### Item-restscore
```{r}
RIrestscore(d)
```
#### Residual correlations
```{r}
simcor1 <- RIgetResidCor(d, iterations = 250, cpu = 8)
RIresidcorr(d, cutoff = simcor1$p99)
```
#### Local dependency
```{r}
# using partial gamma LD from library(iarm)
RIpartgamLD(d)
```
#### 1st contrast loadings
```{r}
RIloadLoc(d)
```
#### Score groups obs-exp
```{r}
item_obsexp(PCM(d))
```
:::

Looks good in terms of dimensionality, let's review the response thresholds again.

#### Response categories
```{r}
mirt(d, model=1, itemtype='Rasch', verbose = FALSE) %>% 
  plot(type="trace", as.table = TRUE, 
       theta_lim = c(-6,6))

RIitemHierarchy(d)
```

Like before, item q1 demonstrates well ordered thresholds, and item q8 is problematic in terms of the two higher thresholds. Item q3 also has very disordered thresholds.

Recoding of response categories.

```{r}
d %>% 
  mutate(q3 = car::recode(q3,"2=1;3=2;4=2"),
         q8 = car::recode(q8,"4=3")) %>% 
  RItileplot()
```

```{r}
d %>% 
  mutate(q3 = car::recode(q3,"2=1;3=2;4=2"),
         q8 = car::recode(q8,"4=3")) %>% 
  mirt(model=1, itemtype='Rasch', verbose = FALSE) %>% 
  plot(type="trace", as.table = TRUE, 
       theta_lim = c(-6,6))
```

Now, looks better again.

```{r}
d2 <- d %>% 
  mutate(q3 = car::recode(q3,"2=1;3=2;4=2"),
         q8 = car::recode(q8,"4=3"))
```

Now all that remains is to inspect the test information.

### Test Information (Reliability)
```{r}
RItif(d2, samplePSI = T)
```

Similar to the other version of the scale, in spite of solving item misfit, residual correlations, and disordered response categories, the test information is still very low, indicating a low reliability of the scale.
```
