# Supplementary material for: Psychometric properties of the Swedish cardiac anxiety questionnaire: a Rasch analysis
Source: Sci Rep. 2025 Nov 24;15:41834. doi: 10.1038/s41598-025-28073-8 (PMC12647126; doi:10.1038/s41598-025-28073-8)
Supplement: Supplementary file 1 — Supplementary Material 1 [file 41598_2025_28073_MOESM1_ESM.zip › Supplementary/analysis_av.html]

CAQ psychometric analysis, avoidance subscale


# CAQ psychometric analysis, avoidance subscale

 Code

- Show All Code
- Hide All Code
- ---
- View Source

Using Rasch Measurement Theory

Author

Affiliation

Magnus Johansson; Philip Leissner

RISE Research Institutes of Sweden; Department of women’s and children’s health, Uppsala University

## Table of contents

- 1 All items in the analysis
- 2 Descriptives - item level
- 3 Avoidance subscale
  - 3.1 Sumscore
  - 3.2 Rasch analysis
  - 3.3 Avoidance without q5
  - 3.4 Test Information (Reliability)
  - 3.5 Avoidance without 5 and 12
  - 3.6 Test Information (Reliability)
- 4 Forming testlets
  - 4.1 Test Information (Reliability)

## Other Formats

- PDF

Code

```
# one package below requires that you use devtools to install them manually:
# first install devtools by
# install.packages('devtools')

library(easyRasch) # devtools::install_github("pgmj/easyRasch")
library(grateful)
library(ggrepel)
library(car)
library(kableExtra)
library(readxl)
library(tidyverse)
library(eRm)
library(iarm)
library(mirt)
library(psych)
library(psychotree)
library(matrixStats)
library(reshape)
library(knitr)
library(patchwork)
library(formattable) 
library(glue)
library(readxl) # for reading excel files

### optional libraries
#library(TAM)
#library(skimr)
#library(janitor)

### some commands exist in multiple packages, here we define preferred ones that are frequently used
select <- dplyr::select
count <- dplyr::count
recode <- car::recode
rename <- dplyr::rename
```

Code

```
### import data - this is just sample code, the files do not exist
df <- read_excel("data/CAQ_Rasch.xlsx") # replace with your datafile as needed

#library(haven) # for SPSS and other formats
#library(labelled) # for getting labels and metadata from SPSS files

### Load item information
# make sure that variable names in df match with itemlabels$itemnr
iteminfo <- read_excel("data/iteminfo.xlsx")

itemlabels <- iteminfo[,1:2]

### Make a backup of the dataframe, in case you need to revert changes at some point
d <- df
```

Code

```
##### Optionally: filter participants based on missing data

##### Before filtering out participants, you should check the missing data structure using RImissing() and RImissingP()

RImissing(d)
```

RImissingP() needs to be addressed, so we’ll do a manual check:

Code

```
d[,6:23] %>% 
  mutate(missing = rowSums(is.na(.))) %>% 
  count(missing)
```

```
# A tibble: 10 × 2
   missing     n
     <dbl> <int>
 1       0   756
 2       1    28
 3       2     8
 4       3     1
 5       4     3
 6       5     2
 7       9     1
 8      10     1
 9      16     1
10      18     5
```

We lose 50 respondents if we remove everyone with a missing value on any item. 756 is a good sample size, so we’ll go with that.

Code

```
d <- na.omit(d)
```

2 who were missing on some demographic variable were also removed.

Code

```
#---- Create DIF variables----
  
# DIF variables into vectors, recoded as factors since DIF functions need this
# these could also be stored in its own dataframe (not a tibble) instead of as vectors

d_dif <- d %>% 
  mutate(sex = factor(SEX),
         age = AGE_R,
         rel_status = factor(RELSTAT_bin),
         born_swe = factor(BORNSWE),
         edu = factor(EDUCATION)) %>% 
  select(sex,age,rel_status,born_swe,edu)

# remove DIF variables from item data
d <- d %>% 
  select(starts_with("Item")) %>% 
  set_names(itemlabels$itemnr)


### label sex variable as factor
# dif.sex <- factor(dif.sex,
#                       levels = c(1,2,3),
#                       labels = c("Female", "Male", "Other/missing response"))

# optionally, load RISE ggplot theme and color palettes and set the theme as default.
# just comment out the row below if you desire different theming
source("RISE_theme.R")
```

## 1 All items in the analysis

Code

```
RIlistitems(d)
```

| itemnr | item |
| --- | --- |
| q1 | I pay attention to my heart beat |
| q2 | I avoid physical exertion |
| q3 | My racing heart wakes me up at night |
| q4 | Chest pain/discomfort wakes me up at night |
| q5 | I take it easy as much as possible |
| q6 | I check my pulse |
| q7 | I avoid exercise or other physical work |
| q8 | I can feel my heart in my chest |
| q9 | I avoid activities that make my heart beat faster |
| q10 | If tests come out normal, I still worry about my heart |
| q11 | I feel safe being around a hospital, physician, or other medical facility |
| q12 | I avoid activities that make me sweat |
| q13 | I worry that doctors do not believe my chest pain/discomfort is real |
| q14 | When I have chest discomfort or I feel my heart is beating fast I worry that I may have a heart attack |
| q15 | When I have chest discomfort or I feel my heart is beating fast I have difficulty concentrating on anything else |
| q16 | When I have chest discomfort or I feel my heart is beating fast I get frightened |
| q17 | When I have chest discomfort or I feel my heart is beating fast I like to be checked out by a doctor |
| q18 | When I have chest discomfort or I feel my heart is beating fast I tell my family or friends |

Response distribution for all items are summarized below.

Code

```
RIallresp(d)
```

| Response category | Number of responses | Percent |
| --- | --- | --- |
| 0 | 5359 | 39.5 |
| 1 | 3580 | 26.4 |
| 2 | 2767 | 20.4 |
| 3 | 1244 | 9.2 |
| 4 | 622 | 4.6 |

## 2 Descriptives - item level

Code

```
RIlistItemsMargin(d, fontsize = 12)
```

| itemnr | item |
| --- | --- |
| q1 | I pay attention to my heart beat |
| q2 | I avoid physical exertion |
| q3 | My racing heart wakes me up at night |
| q4 | Chest pain/discomfort wakes me up at night |
| q5 | I take it easy as much as possible |
| q6 | I check my pulse |
| q7 | I avoid exercise or other physical work |
| q8 | I can feel my heart in my chest |
| q9 | I avoid activities that make my heart beat faster |
| q10 | If tests come out normal, I still worry about my heart |
| q11 | I feel safe being around a hospital, physician, or other medical facility |
| q12 | I avoid activities that make me sweat |
| q13 | I worry that doctors do not believe my chest pain/discomfort is real |
| q14 | When I have chest discomfort or I feel my heart is beating fast I worry that I may have a heart attack |
| q15 | When I have chest discomfort or I feel my heart is beating fast I have difficulty concentrating on anything else |
| q16 | When I have chest discomfort or I feel my heart is beating fast I get frightened |
| q17 | When I have chest discomfort or I feel my heart is beating fast I like to be checked out by a doctor |
| q18 | When I have chest discomfort or I feel my heart is beating fast I tell my family or friends |

- Tile plot
- Stacked bars
- Barplots

Code

```
RItileplot(d)
```

Code

```
RIbarstack(d) + scale_fill_viridis_d(labels = c("Always","Often","Sometimes","Rarely","Never"), direction = -1)
```

Code

```
RIbarplot(d)
```

Very few individuals endorse the highest categories of items q3, q4, q10, and q13. Over half of the participants endorse the lowest category for items q3, q4, and q13.

## 3 Avoidance subscale

Code

```
d_all <- d

items_av <- iteminfo %>% 
  filter(factor == "avoidance") %>% 
  pull(itemnr)

d <- d_all %>% 
  select(all_of(items_av))
```

### 3.1 Sumscore

Code

```
d_sum <- d %>%
  mutate(total = rowSums(select(., starts_with("q")), na.rm = TRUE))
d_sum <- d_sum["total"]
```

Code

```
total_freq <- as.data.frame(table(d_sum$total))
colnames(total_freq) <- c("Total", "N")
total_freq$Percent <- round(100 * total_freq$N / sum(total_freq$N), 1)
```

Code

```
RIallresp(d_sum)
```

| Response category | Number of responses | Percent |
| --- | --- | --- |
| 0 | 36 | 4.8 |
| 1 | 47 | 6.2 |
| 2 | 63 | 8.4 |
| 3 | 77 | 10.2 |
| 4 | 71 | 9.4 |
| 5 | 77 | 10.2 |
| 6 | 82 | 10.9 |
| 7 | 54 | 7.2 |
| 8 | 47 | 6.2 |
| 9 | 47 | 6.2 |
| 10 | 47 | 6.2 |
| 11 | 26 | 3.4 |
| 12 | 19 | 2.5 |
| 13 | 15 | 2.0 |
| 14 | 21 | 2.8 |
| 15 | 9 | 1.2 |
| 16 | 7 | 0.9 |
| 17 | 1 | 0.1 |
| 18 | 6 | 0.8 |
| 20 | 2 | 0.3 |

### 3.2 Rasch analysis

The eRm package, which uses Conditional Maximum Likelihood (CML) estimation, will be used primarily. For this analysis, the Partial Credit Model will be used.

| itemnr | item |
| --- | --- |
| q2 | I avoid physical exertion |
| q5 | I take it easy as much as possible |
| q7 | I avoid exercise or other physical work |
| q9 | I avoid activities that make my heart beat faster |
| q12 | I avoid activities that make me sweat |

- Conditional item fit
- Item-restscore
- Conditional LRT
- Local dependency
- Residual correlations
- 1st contrast loadings
- Response categories
- Targeting
- Item hierarchy
- Score groups LR-test
- Score groups obs-exp
- Rasch-tree DIF immigration status
- Rasch-tree DIF sex
- Rasch-tree DIF rel
- Rasch-tree DIF age
- Rasch-tree DIF rel\*age
- Person fit
- Floor and ceiling effects

Code

```
#RIitemfit(d, cutoff = "Smith98")

simfit1 <- RIgetfit(d, iterations = 200, cpu = 8) 

RIitemfit(d, simfit1)
```

| Item | InfitMSQ | Infit thresholds | OutfitMSQ | Outfit thresholds | Infit diff | Outfit diff | Relative location |
| --- | --- | --- | --- | --- | --- | --- | --- |
| q2 | 0.812 | [0.892, 1.12] | 0.824 | [0.829, 1.213] | 0.08 | 0.005 | 1.41 |
| q5 | 1.577 | [0.893, 1.115] | 1.548 | [0.912, 1.119] | 0.462 | 0.429 | -0.01 |
| q7 | 0.833 | [0.907, 1.099] | 0.802 | [0.911, 1.097] | 0.074 | 0.109 | 1.35 |
| q9 | 0.925 | [0.872, 1.143] | 0.9 | [0.846, 1.185] | no misfit | no misfit | 1.66 |
| q12 | 0.832 | [0.911, 1.124] | 0.694 | [0.846, 1.156] | 0.079 | 0.152 | 1.75 |
|  |
| --- |
| Note: |
| MSQ values based on conditional calculations (n = 754 complete cases).  Simulation based thresholds from 200 simulated datasets. |

Code

```
RIgetfitPlot(simfit1, d)
```

Code

```
RIrestscore(d)
```

| Item | Observed value | Model expected value | Absolute difference | Adjusted p-value (BH) | Statistical significance level | Location | Relative location |
| --- | --- | --- | --- | --- | --- | --- | --- |
| q2 | 0.70 | 0.60 | 0.10 | 0.000 | \*\*\* | 0.17 | 1.41 |
| q5 | 0.37 | 0.60 | 0.23 | 0.000 | \*\*\* | -1.24 | -0.01 |
| q7 | 0.69 | 0.61 | 0.08 | 0.000 | \*\*\* | 0.12 | 1.35 |
| q9 | 0.66 | 0.60 | 0.06 | 0.032 | \* | 0.43 | 1.66 |
| q12 | 0.72 | 0.61 | 0.11 | 0.000 | \*\*\* | 0.52 | 1.75 |

Code

```
clr_tests(d, model = "PCM")
```

```
Conditional Likelihood Ratio Tests:
```

```
        clr df pvalue  sig 
overall 117 19 3.3e-16  ***
```

Code

```
RIbootLRT(d, iterations = 1000, samplesize = 400, cpu = 8)
```

| Result | n | Percent |
| --- | --- | --- |
| Not statistically significant | 3 | 0.3 |
| Statistically significant | 997 | 99.7 |

Code

```
# using partial gamma LD from library(iarm)
RIpartgamLD(d)
```

| Item 1 | Item 2 | Partial gamma | SE | Lower CI | Upper CI | Adjusted p-value (BH) |
| --- | --- | --- | --- | --- | --- | --- |
| q12 | q9 | 0.474 | 0.059 | 0.358 | 0.590 | 0 |
| q7 | q2 | 0.43 | 0.063 | 0.306 | 0.553 | 0 |
| q2 | q7 | 0.41 | 0.066 | 0.282 | 0.539 | 0 |
| q9 | q12 | 0.407 | 0.063 | 0.282 | 0.531 | 0 |

Code

```
simcor1 <- RIgetResidCor(d, iterations = 250, cpu = 8)
RIresidcorr(d, cutoff = simcor1$p99)
```

|  | q2 | q5 | q7 | q9 | q12 |
| --- | --- | --- | --- | --- | --- |
| q2 |  |  |  |  |  |
| q5 | -0.32 |  |  |  |  |
| q7 | 0.05 | -0.3 |  |  |  |
| q9 | -0.19 | -0.32 | -0.28 |  |  |
| q12 | -0.17 | -0.35 | -0.17 | 0.04 |  |
|  |
| --- |
| Note: |
| Relative cut-off value is -0.081, which is 0.119 above the average correlation (-0.201).  Correlations above the cut-off are highlighted in red text. |

Code

```
RIloadLoc(d)
```

Code

```
mirt(d, model=1, itemtype='Rasch', verbose = FALSE) %>% 
  plot(type="trace", as.table = TRUE, 
       theta_lim = c(-6,6))
```

Code

```
# for fewer items or a more magnified figure, use:
#RIitemCats(d)
```

Code

```
# increase fig-height above as needed, if you have many items
RItargeting(d)
```

Code

```
RIitemHierarchy(d)
```

Code

```
iarm::score_groups(as.data.frame(d)) %>% 
  as.data.frame(nm = "score_group") %>% 
  dplyr::count(score_group)
```

```
  score_group   n
1           1 371
2           2 383
```

Code

```
dif_plots <- d %>% 
  add_column(dif = iarm::score_groups(.)) %>% 
  split(.$dif) %>% # split the data using the DIF variable
  map(~ RItileplot(.x %>% dplyr::select(!dif)) + labs(title = .x$dif))
dif_plots[[1]] + dif_plots[[2]]
```

Code

```
clr_tests(d, model = "PCM")
```

```
Conditional Likelihood Ratio Tests:
```

```
        clr df pvalue  sig 
overall 117 19 3.3e-16  ***
```

Code

```
item_obsexp(PCM(d))
```

```
Score group 1: 
    mean obs mean exp std.res sig
q2   0.591    0.635   -1.376     
q5   1.684    1.511    5.047  ++ 
q7   0.433    0.483   -1.585     
q9   0.304    0.314   -0.359     
q12  0.191    0.253   -2.413  -  

Score group 2: 
    mean obs mean exp std.res sig
q2   1.861    1.822    1.034     
q5   2.465    2.616   -4.375  -- 
q7   1.879    1.835    1.094     
q9   1.528    1.516    0.283     
q12  1.491    1.435    1.360
```

Code

```
RIdifTable(d, d_dif$born_swe)
```

| Item | 2 | 3 | Mean location | StDev | MaxDiff |
| --- | --- | --- | --- | --- | --- |
| q2 | 0.203 | 0.166 | 0.184 | 0.026 | 0.037 |
| q5 | -0.990 | -1.287 | -1.138 | 0.210 | 0.297 |
| q7 | -0.046 | 0.151 | 0.052 | 0.139 | 0.197 |
| q9 | 0.287 | 0.453 | 0.370 | 0.118 | 0.166 |
| q12 | 0.547 | 0.517 | 0.532 | 0.021 | 0.029 |

Code

```
RIdifTable(d, d_dif$sex)
```

```
[1] "No statistically significant DIF found."
```

Code

```
RIdifTable(d, d_dif$rel_status)
```

| Item | 2 | 3 | Mean location | StDev | MaxDiff |
| --- | --- | --- | --- | --- | --- |
| q2 | 0.264 | 0.070 | 0.167 | 0.137 | 0.194 |
| q5 | -1.323 | -1.125 | -1.224 | 0.140 | 0.197 |
| q7 | 0.041 | 0.293 | 0.167 | 0.178 | 0.252 |
| q9 | 0.406 | 0.429 | 0.417 | 0.016 | 0.022 |
| q12 | 0.612 | 0.334 | 0.473 | 0.197 | 0.278 |

Code

```
RIdifTable(d, d_dif$age)
```

| Item | 2 | 3 | Mean location | StDev | MaxDiff |
| --- | --- | --- | --- | --- | --- |
| q2 | 0.167 | 0.189 | 0.178 | 0.015 | 0.022 |
| q5 | -1.246 | -1.234 | -1.240 | 0.008 | 0.011 |
| q7 | 0.009 | 0.297 | 0.153 | 0.204 | 0.288 |
| q9 | 0.466 | 0.362 | 0.414 | 0.074 | 0.104 |
| q12 | 0.604 | 0.386 | 0.495 | 0.154 | 0.217 |

Code

```
RIdifTable2(d, d_dif$rel_status, d_dif$sex)
```

```
[1] "No statistically significant DIF found."
```

Code

```
RIpfit(d)
```

Code

```
RItif(d, samplePSI = T, cutoff = 1)
```

Item q5 is highly underfit, while the other items are slightly overfit.

There is a residual correlation between items q2 and q7, as well as between items q9 and q12.

All response thresholds look fine.

No significant level of DIF is observed.

Since item q5 has dimensionality issues, we will remove it. We may also need to remove items q2 or q12 because of residual correlations, but if we follow this logic, we are left with only two items, which likely won’t form a stable subscale. In any case, we’ll start by removing item 5.

### 3.3 Avoidance without q5

Code

```
d$q5 <- NULL
```

- Conditional item fit
- Item-restscore
- Residual correlations
- Response categories
- Local dependency
- 1st contrast loadings
- Score groups obs-exp

Code

```
simfit1 <- RIgetfit(d, iterations = 200, cpu = 8) 

RIitemfit(d, simfit1)
```

| Item | InfitMSQ | Infit thresholds | OutfitMSQ | Outfit thresholds | Infit diff | Outfit diff | Relative location |
| --- | --- | --- | --- | --- | --- | --- | --- |
| q2 | 0.975 | [0.819, 1.143] | 0.979 | [0.84, 1.154] | no misfit | no misfit | 1.57 |
| q7 | 1.028 | [0.875, 1.1] | 1.029 | [0.888, 1.087] | no misfit | no misfit | 1.56 |
| q9 | 1.082 | [0.867, 1.103] | 1.096 | [0.889, 1.1] | no misfit | no misfit | 1.96 |
| q12 | 0.93 | [0.865, 1.142] | 0.859 | [0.874, 1.144] | no misfit | 0.015 | 2.07 |
|  |
| --- |
| Note: |
| MSQ values based on conditional calculations (n = 754 complete cases).  Simulation based thresholds from 200 simulated datasets. |

Code

```
RIgetfitPlot(simfit1, d)
```

Code

```
RIrestscore(d)
```

| Item | Observed value | Model expected value | Absolute difference | Adjusted p-value (BH) | Statistical significance level | Location | Relative location |
| --- | --- | --- | --- | --- | --- | --- | --- |
| q2 | 0.73 | 0.71 | 0.02 | 0.561 |  | -0.22 | 1.57 |
| q7 | 0.71 | 0.71 | 0.00 | 0.919 |  | -0.23 | 1.56 |
| q9 | 0.68 | 0.70 | 0.02 | 0.561 |  | 0.16 | 1.96 |
| q12 | 0.75 | 0.70 | 0.05 | 0.127 |  | 0.28 | 2.07 |

Code

```
simcor1 <- RIgetResidCor(d, iterations = 250, cpu = 8)
RIresidcorr(d, cutoff = simcor1$p99)
```

|  | q2 | q7 | q9 | q12 |
| --- | --- | --- | --- | --- |
| q2 |  |  |  |  |
| q7 | -0.1 |  |  |  |
| q9 | -0.36 | -0.46 |  |  |
| q12 | -0.35 | -0.36 | -0.13 |  |
|  |
| --- |
| Note: |
| Relative cut-off value is -0.171, which is 0.122 above the average correlation (-0.292).  Correlations above the cut-off are highlighted in red text. |

Code

```
mirt(d, model=1, itemtype='Rasch', verbose = FALSE) %>% 
  plot(type="trace", as.table = TRUE, 
       theta_lim = c(-6,6))
```

Code

```
# for fewer items or a more magnified figure, use:
#RIitemCats(d)
```

Code

```
# using partial gamma LD from library(iarm)
RIpartgamLD(d)
```

| Item 1 | Item 2 | Partial gamma | SE | Lower CI | Upper CI | Adjusted p-value (BH) |
| --- | --- | --- | --- | --- | --- | --- |
| q12 | q9 | 0.412 | 0.070 | 0.274 | 0.550 | 0.000 |
| q2 | q7 | 0.37 | 0.076 | 0.221 | 0.519 | 0.000 |
| q7 | q2 | 0.351 | 0.074 | 0.206 | 0.497 | 0.000 |
| q9 | q12 | 0.262 | 0.073 | 0.118 | 0.406 | 0.004 |

Code

```
RIloadLoc(d)
```

Code

```
item_obsexp(PCM(d))
```

```
Score group 1: 
    mean obs mean exp std.res sig
q2   0.942    0.964   -0.695     
q7   0.777    0.774    0.111     
q9   0.527    0.497    1.004     
q12  0.387    0.396   -0.308     

Score group 2: 
    mean obs mean exp std.res sig
q2   2.098    2.073    0.650     
q7   2.126    2.131   -0.102     
q9   1.793    1.828   -0.814     
q12  1.772    1.758    0.318
```

There is still residual correlation between items q12 and q9, as well as between items q7 and q2.

Let’s inspect the test information before we move on by removing q12.

### 3.4 Test Information (Reliability)

Code

```
RItif(d, samplePSI = T)
```

The test information curve is somewhat close to the cut-off but still below, indicating a low reliability of the scale. Let’s try the 3-item version, also removing item q12.

### 3.5 Avoidance without 5 and 12

Code

```
d$q12 <- NULL
```

- Conditional item fit
- Item-restscore
- Response categories
- Residual correlations
- Local dependency
- Score groups obs-exp

Code

```
simfit1 <- RIgetfit(d, iterations = 200, cpu = 8) 

RIitemfit(d, simfit1)
```

| Item | InfitMSQ | Infit thresholds | OutfitMSQ | Outfit thresholds | Infit diff | Outfit diff | Relative location |
| --- | --- | --- | --- | --- | --- | --- | --- |
| q2 | 0.885 | [0.904, 1.09] | 0.885 | [0.914, 1.085] | 0.019 | 0.029 | 1.52 |
| q7 | 0.942 | [0.9, 1.102] | 0.939 | [0.909, 1.101] | no misfit | no misfit | 1.50 |
| q9 | 1.193 | [0.902, 1.124] | 1.178 | [0.898, 1.124] | 0.069 | 0.054 | 1.88 |
|  |
| --- |
| Note: |
| MSQ values based on conditional calculations (n = 754 complete cases).  Simulation based thresholds from 200 simulated datasets. |

Code

```
RIgetfitPlot(simfit1, d)
```

Code

```
RIrestscore(d)
```

| Item | Observed value | Model expected value | Absolute difference | Adjusted p-value (BH) | Statistical significance level | Location | Relative location |
| --- | --- | --- | --- | --- | --- | --- | --- |
| q2 | 0.75 | 0.69 | 0.06 | 0.035 | \* | -0.11 | 1.52 |
| q7 | 0.72 | 0.68 | 0.04 | 0.136 |  | -0.13 | 1.50 |
| q9 | 0.62 | 0.68 | 0.06 | 0.104 |  | 0.24 | 1.88 |

Code

```
mirt(d, model=1, itemtype='Rasch', verbose = FALSE) %>% 
  plot(type="trace", as.table = TRUE, 
       theta_lim = c(-6,6))
```

Code

```
# for fewer items or a more magnified figure, use:
#RIitemCats(d)
```

Code

```
simcor1 <- RIgetResidCor(d, iterations = 250, cpu = 8)
RIresidcorr(d, cutoff = simcor1$p99)
```

|  | q2 | q7 | q9 |
| --- | --- | --- | --- |
| q2 |  |  |  |
| q7 | -0.24 |  |  |
| q9 | -0.44 | -0.53 |  |
|  |
| --- |
| Note: |
| Relative cut-off value is -0.304, which is 0.1 above the average correlation (-0.404).  Correlations above the cut-off are highlighted in red text. |

Code

```
# using partial gamma LD from library(iarm)
RIpartgamLD(d)
```

| Item 1 | Item 2 | Partial gamma | SE | Lower CI | Upper CI | Adjusted p-value (BH) |
| --- | --- | --- | --- | --- | --- | --- |
| q2 | q7 | 0.436 | 0.075 | 0.289 | 0.582 | 0 |
| q7 | q2 | 0.359 | 0.074 | 0.214 | 0.504 | 0 |

Code

```
item_obsexp(PCM(d))
```

```
Score group 1: 
   mean obs mean exp std.res sig
q2  0.898    0.935   -1.144     
q7  0.694    0.726   -0.957     
q9  0.526    0.456    2.315  +  

Score group 2: 
   mean obs mean exp std.res sig
q2  2.072    2.035    1.011     
q7  2.125    2.093    0.837     
q9  1.711    1.779   -1.757
```

The item fit for the remaining items seems to have worsened, and the residual correlation between items q2 and q7 has become slightly stronger.

Response thresholds seem to be working quite well.

### 3.6 Test Information (Reliability)

Code

```
RItif(d, samplePSI = T)
```

TIF shows that this version has lower reliability than the previous 4-item version. The most acceptable version of the scale seems to be also including item q12, despite residual correlations.

## 4 Forming testlets

We can also try combining the two pairs of items that have residual correlations and see if these thresholds work.

Code

```
items_av <- iteminfo %>% 
  filter(factor == "avoidance") %>% 
  pull(itemnr)

d <- d_all %>% 
  select(all_of(items_av))
```

Code

```
# Combine item 2 and 7 into one "superitem" by averaging
d$q19 <- d$q2 + d$q7


# Combine item 9 and 12 into one "superitem" by averaging
d$q20 <- d$q9 + d$q12

d$q12 <- NULL
d$q7 <- NULL
d$q9 <- NULL
d$q2 <- NULL

print(d)
```

```
# A tibble: 754 × 3
      q5   q19   q20
   <dbl> <dbl> <dbl>
 1     3     2     0
 2     1     4     0
 3     1     1     0
 4     2     0     1
 5     1     2     0
 6     2     4     4
 7     3     2     2
 8     4     6     6
 9     2     4     4
10     1     1     3
# ℹ 744 more rows
```

Code

```
# drop single items
```

#### 4.0.1 Response categories

Code

```
mirt(d, model=1, itemtype='Rasch', verbose = FALSE) %>% 
  plot(type="trace", as.table = TRUE, 
       theta_lim = c(-6,6))
```

Code

```
# for fewer items or a more magnified figure, use:
#RIitemCats(d)
```

Recoding response categories

Code

```
d %>% 
  mutate(q20 = car::recode(q20,"2=1;3=2;4=2;5=3;6=3;7=4;8=4"),
         q19 = car::recode(q19,"2=1;3=2;4=2;5=3;6=3;7=4;8=4")) %>% 
  RItileplot()
```

Code

```
d %>% 
  mutate(q20 = car::recode(q20,"2=1;3=2;4=2;5=3;6=3;7=4;8=4"),
         q19 = car::recode(q19,"2=1;3=2;4=2;5=3;6=3;7=4;8=4")) %>% 
  mirt(model=1, itemtype='Rasch', verbose = FALSE) %>% 
  plot(type="trace", as.table = TRUE, 
       theta_lim = c(-6,6))
```

Code

```
d2 <- d %>% 
  mutate(q20 = car::recode(q20,"2=1;3=2;4=2;5=3;6=3;7=4;8=4"),
         q19 = car::recode(q19,"2=1;3=2;4=2;5=3;6=3;7=4;8=4"))
```

Response categories looks nice when adding together these two items and only having 4 categories.

Let’s see how they work together with item 5, although including it will likely lead to item misfit again.

#### 4.0.2 Conditional item fit

Code

```
simfit1 <- RIgetfit(d2, iterations = 200, cpu = 8) 

RIitemfit(d2, simfit1)
```

| Item | InfitMSQ | Infit thresholds | OutfitMSQ | Outfit thresholds | Infit diff | Outfit diff | Relative location |
| --- | --- | --- | --- | --- | --- | --- | --- |
| q5 | 1.35 | [0.899, 1.083] | 1.328 | [0.886, 1.088] | 0.267 | 0.240 | 0.00 |
| q19 | 0.787 | [0.898, 1.109] | 0.795 | [0.876, 1.146] | 0.111 | 0.081 | 1.06 |
| q20 | 0.845 | [0.903, 1.101] | 0.814 | [0.909, 1.094] | 0.058 | 0.095 | 1.47 |
|  |
| --- |
| Note: |
| MSQ values based on conditional calculations (n = 754 complete cases).  Simulation based thresholds from 200 simulated datasets. |

Code

```
RIgetfitPlot(simfit1, d2)
```

Although less underfit, item q5 is still an issue. Removing it will leave us with a scale that only has two items which will be to unstable.

Code

```
# Drop item 5
d2$q5 <- NULL
```

#### 4.0.3 Conditional item fit

Code

```
simfit1 <- RIgetfit(d2, iterations = 200, cpu = 8) 

RIitemfit(d2, simfit1)
```

| Item | InfitMSQ | Infit thresholds | OutfitMSQ | Outfit thresholds | Infit diff | Outfit diff | Relative location |
| --- | --- | --- | --- | --- | --- | --- | --- |
| q19 | 0.995 | [0.985, 1.014] | 0.991 | [0.981, 1.019] | no misfit | no misfit | 1.42 |
| q20 | 0.995 | [0.985, 1.014] | 0.991 | [0.981, 1.019] | no misfit | no misfit | 2.13 |
|  |
| --- |
| Note: |
| MSQ values based on conditional calculations (n = 754 complete cases).  Simulation based thresholds from 200 simulated datasets. |

Code

```
RIgetfitPlot(simfit1, d2)
```

#### 4.0.4 Targeting

Code

```
# increase fig-height above as needed, if you have many items
RItargeting(d2)
```

### 4.1 Test Information (Reliability)

Code

```
RItif(d2, samplePSI = T)
```

Although there is no longer item misfit, the test information is too low with only these two items.

## Reuse

CC BY 4.0

##### Source Code

```
---
title: "CAQ psychometric analysis, avoidance subscale"
subtitle: "Using Rasch Measurement Theory"
title-block-banner: "#009ca6"
title-block-banner-color: "#FFFFFF"
author: 
  name: Magnus Johansson; Philip Leissner
  affiliation: RISE Research Institutes of Sweden; Department of women's and children's health, Uppsala University
  affiliation-url: https://www.ri.se/en/kbm; https://www.uu.se/en/department/womens-and-childrens-health
  orcid: 0000-0003-1669-592X; 0000-0003-0787-9102
date-format: iso
always_allow_html: true
format: 
  html:
    toc: true
    toc-depth: 3
    toc-title: "Table of contents"
    embed-resources: true
    standalone: true
    page-layout: full
    mainfont: 'Lato'
    monofont: 'Roboto Mono'
    code-overflow: wrap
    code-fold: true
    code-tools: true
    code-link: true
    number-sections: true
    fig-dpi: 96
    layout-align: left
    linestretch: 1.6
    theme:
      - materia
      - custom.scss
    css: styles.css
    license: CC BY
  pdf:
    papersize: a4
    documentclass: report 
execute:
  echo: true
  warning: false
  message: false
  cache: true
editor_options: 
  markdown: 
    wrap: 72
  chunk_output_type: console
---

```{r}
#| label: setup

# one package below requires that you use devtools to install them manually:
# first install devtools by
# install.packages('devtools')

library(easyRasch) # devtools::install_github("pgmj/easyRasch")
library(grateful)
library(ggrepel)
library(car)
library(kableExtra)
library(readxl)
library(tidyverse)
library(eRm)
library(iarm)
library(mirt)
library(psych)
library(psychotree)
library(matrixStats)
library(reshape)
library(knitr)
library(patchwork)
library(formattable) 
library(glue)
library(readxl) # for reading excel files

### optional libraries
#library(TAM)
#library(skimr)
#library(janitor)

### some commands exist in multiple packages, here we define preferred ones that are frequently used
select <- dplyr::select
count <- dplyr::count
recode <- car::recode
rename <- dplyr::rename
```


```{r}
### import data - this is just sample code, the files do not exist
df <- read_excel("data/CAQ_Rasch.xlsx") # replace with your datafile as needed

#library(haven) # for SPSS and other formats
#library(labelled) # for getting labels and metadata from SPSS files

### Load item information
# make sure that variable names in df match with itemlabels$itemnr
iteminfo <- read_excel("data/iteminfo.xlsx")

itemlabels <- iteminfo[,1:2]

### Make a backup of the dataframe, in case you need to revert changes at some point
d <- df
```

```{r}
##### Optionally: filter participants based on missing data

##### Before filtering out participants, you should check the missing data structure using RImissing() and RImissingP()

RImissing(d)
```

RImissingP() needs to be addressed, so we'll do a manual check:

```{r}
d[,6:23] %>% 
  mutate(missing = rowSums(is.na(.))) %>% 
  count(missing)
```

We lose 50 respondents if we remove everyone with a missing value on any item. 756 is a good sample size, so we'll go with that.

```{r}
d <- na.omit(d)
```

2 who were missing on some demographic variable were also removed.

```{r}
#---- Create DIF variables----
  
# DIF variables into vectors, recoded as factors since DIF functions need this
# these could also be stored in its own dataframe (not a tibble) instead of as vectors

d_dif <- d %>% 
  mutate(sex = factor(SEX),
         age = AGE_R,
         rel_status = factor(RELSTAT_bin),
         born_swe = factor(BORNSWE),
         edu = factor(EDUCATION)) %>% 
  select(sex,age,rel_status,born_swe,edu)

# remove DIF variables from item data
d <- d %>% 
  select(starts_with("Item")) %>% 
  set_names(itemlabels$itemnr)


### label sex variable as factor
# dif.sex <- factor(dif.sex,
#                       levels = c(1,2,3),
#                       labels = c("Female", "Male", "Other/missing response"))

# optionally, load RISE ggplot theme and color palettes and set the theme as default.
# just comment out the row below if you desire different theming
source("RISE_theme.R")
```

## All items in the analysis
```{r}
RIlistitems(d)
```

Response distribution for all items are summarized below.

```{r}
#| tbl-cap: "Total number of responses for all items"
RIallresp(d)
```

## Descriptives - item level

```{r}
#| column: margin
RIlistItemsMargin(d, fontsize = 12)
```

::: panel-tabset
### Tile plot
```{r}
RItileplot(d)
```
### Stacked bars
```{r}
RIbarstack(d) + scale_fill_viridis_d(labels = c("Always","Often","Sometimes","Rarely","Never"), direction = -1)
```
### Barplots
```{r}
#| layout-ncol: 2
RIbarplot(d)
```
:::

Very few individuals endorse the highest categories of items q3, q4, q10, and q13. Over half of the participants endorse the lowest category for items q3, q4, and q13.

## Avoidance subscale

```{r}
d_all <- d

items_av <- iteminfo %>% 
  filter(factor == "avoidance") %>% 
  pull(itemnr)

d <- d_all %>% 
  select(all_of(items_av))
  
```

### Sumscore
```{r}
d_sum <- d %>%
  mutate(total = rowSums(select(., starts_with("q")), na.rm = TRUE))
d_sum <- d_sum["total"]
```

```{r}
total_freq <- as.data.frame(table(d_sum$total))
colnames(total_freq) <- c("Total", "N")
total_freq$Percent <- round(100 * total_freq$N / sum(total_freq$N), 1)
```

```{r}
RIallresp(d_sum)
```

### Rasch analysis

The eRm package, which uses Conditional Maximum Likelihood (CML)
estimation, will be used primarily. For this analysis, the Partial
Credit Model will be used.

```{r}
#| column: margin
#| echo: false
RIlistItemsMargin(d, fontsize = 13)
```

::: panel-tabset
#### Conditional item fit
```{r}
#RIitemfit(d, cutoff = "Smith98")

simfit1 <- RIgetfit(d, iterations = 200, cpu = 8) 

RIitemfit(d, simfit1)
RIgetfitPlot(simfit1, d)
```
#### Item-restscore
```{r}
RIrestscore(d)
```
#### Conditional LRT
```{r}
clr_tests(d, model = "PCM")

RIbootLRT(d, iterations = 1000, samplesize = 400, cpu = 8)
```
#### Local dependency
```{r}
# using partial gamma LD from library(iarm)
RIpartgamLD(d)
```
#### Residual correlations
```{r}
simcor1 <- RIgetResidCor(d, iterations = 250, cpu = 8)
RIresidcorr(d, cutoff = simcor1$p99)
```
#### 1st contrast loadings
```{r}
RIloadLoc(d)
```
#### Response categories
```{r}
mirt(d, model=1, itemtype='Rasch', verbose = FALSE) %>% 
  plot(type="trace", as.table = TRUE, 
       theta_lim = c(-6,6))
# for fewer items or a more magnified figure, use:
#RIitemCats(d)
```
#### Targeting
```{r}
#| fig-height: 5
# increase fig-height above as needed, if you have many items
RItargeting(d)
```
#### Item hierarchy
```{r}
#| fig-height: 5
RIitemHierarchy(d)
```
#### Score groups LR-test
```{r}
iarm::score_groups(as.data.frame(d)) %>% 
  as.data.frame(nm = "score_group") %>% 
  dplyr::count(score_group)

dif_plots <- d %>% 
  add_column(dif = iarm::score_groups(.)) %>% 
  split(.$dif) %>% # split the data using the DIF variable
  map(~ RItileplot(.x %>% dplyr::select(!dif)) + labs(title = .x$dif))
dif_plots[[1]] + dif_plots[[2]]

clr_tests(d, model = "PCM")
```
#### Score groups obs-exp
```{r}
item_obsexp(PCM(d))
```
#### Rasch-tree DIF immigration status
```{r}
RIdifTable(d, d_dif$born_swe)
```
#### Rasch-tree DIF sex
```{r}
RIdifTable(d, d_dif$sex)
```
#### Rasch-tree DIF rel
```{r}
RIdifTable(d, d_dif$rel_status)
```
#### Rasch-tree DIF age
```{r}
RIdifTable(d, d_dif$age)
```
#### Rasch-tree DIF rel*age
```{r}
RIdifTable2(d, d_dif$rel_status, d_dif$sex)
```
#### Person fit
```{r}
RIpfit(d)
```
#### Floor and ceiling effects
```{r}
RItif(d, samplePSI = T, cutoff = 1)
```
:::


Item q5 is highly underfit, while the other items are slightly overfit.

There is a residual correlation between items q2 and q7, as well as between items q9 and q12.

All response thresholds look fine.

No significant level of DIF is observed.

Since item q5 has dimensionality issues, we will remove it. We may also need to remove items q2 or q12 because of residual correlations, but if we follow this logic, we are left with only two items, which likely won’t form a stable subscale. In any case, we’ll start by removing item 5.

### Avoidance without q5

```{r}
d$q5 <- NULL
```

::: panel-tabset
#### Conditional item fit
```{r}
simfit1 <- RIgetfit(d, iterations = 200, cpu = 8) 

RIitemfit(d, simfit1)
RIgetfitPlot(simfit1, d)
```
#### Item-restscore
```{r}
RIrestscore(d)
```
#### Residual correlations
```{r}
simcor1 <- RIgetResidCor(d, iterations = 250, cpu = 8)
RIresidcorr(d, cutoff = simcor1$p99)
```
#### Response categories
```{r}
mirt(d, model=1, itemtype='Rasch', verbose = FALSE) %>% 
  plot(type="trace", as.table = TRUE, 
       theta_lim = c(-6,6))
# for fewer items or a more magnified figure, use:
#RIitemCats(d)
```
#### Local dependency
```{r}
# using partial gamma LD from library(iarm)
RIpartgamLD(d)
```
#### 1st contrast loadings
```{r}
RIloadLoc(d)
```
#### Score groups obs-exp
```{r}
item_obsexp(PCM(d))
```
:::

There is still residual correlation between items q12 and q9, as well as between items q7 and q2. 

Let's inspect the test information before we move on by removing q12.

### Test Information (Reliability)
```{r}
RItif(d, samplePSI = T)
```

The test information curve is somewhat close to the cut-off but still below, indicating a low reliability of the scale. Let's try the 3-item version, also removing item q12.

### Avoidance without 5 and 12

```{r}
d$q12 <- NULL
```

::: panel-tabset
#### Conditional item fit
```{r}
simfit1 <- RIgetfit(d, iterations = 200, cpu = 8) 

RIitemfit(d, simfit1)
RIgetfitPlot(simfit1, d)
```
#### Item-restscore
```{r}
RIrestscore(d)
```
#### Response categories
```{r}
mirt(d, model=1, itemtype='Rasch', verbose = FALSE) %>% 
  plot(type="trace", as.table = TRUE, 
       theta_lim = c(-6,6))
# for fewer items or a more magnified figure, use:
#RIitemCats(d)
```
#### Residual correlations
```{r}
simcor1 <- RIgetResidCor(d, iterations = 250, cpu = 8)
RIresidcorr(d, cutoff = simcor1$p99)
```
#### Local dependency
```{r}
# using partial gamma LD from library(iarm)
RIpartgamLD(d)
```
#### Score groups obs-exp
```{r}
item_obsexp(PCM(d))
```
:::

The item fit for the remaining items seems to have worsened, and the residual correlation between items q2 and q7 has become slightly stronger.

Response thresholds seem to be working quite well.

### Test Information (Reliability)
```{r}
RItif(d, samplePSI = T)
```

TIF shows that this version has lower reliability than the previous 4-item version. The most acceptable version of the scale seems to be also including item q12, despite residual correlations.

## Forming testlets
We can also try combining the two pairs of items that have residual correlations and see if these thresholds work.

```{r}
items_av <- iteminfo %>% 
  filter(factor == "avoidance") %>% 
  pull(itemnr)

d <- d_all %>% 
  select(all_of(items_av))
  
```

```{r}
# Combine item 2 and 7 into one "superitem" by averaging
d$q19 <- d$q2 + d$q7


# Combine item 9 and 12 into one "superitem" by averaging
d$q20 <- d$q9 + d$q12

d$q12 <- NULL
d$q7 <- NULL
d$q9 <- NULL
d$q2 <- NULL

print(d)
# drop single items
```

#### Response categories
```{r}
mirt(d, model=1, itemtype='Rasch', verbose = FALSE) %>% 
  plot(type="trace", as.table = TRUE, 
       theta_lim = c(-6,6))
# for fewer items or a more magnified figure, use:
#RIitemCats(d)
```

Recoding response categories

```{r}
d %>% 
  mutate(q20 = car::recode(q20,"2=1;3=2;4=2;5=3;6=3;7=4;8=4"),
         q19 = car::recode(q19,"2=1;3=2;4=2;5=3;6=3;7=4;8=4")) %>% 
  RItileplot()

d %>% 
  mutate(q20 = car::recode(q20,"2=1;3=2;4=2;5=3;6=3;7=4;8=4"),
         q19 = car::recode(q19,"2=1;3=2;4=2;5=3;6=3;7=4;8=4")) %>% 
  mirt(model=1, itemtype='Rasch', verbose = FALSE) %>% 
  plot(type="trace", as.table = TRUE, 
       theta_lim = c(-6,6))
```

```{r}
d2 <- d %>% 
  mutate(q20 = car::recode(q20,"2=1;3=2;4=2;5=3;6=3;7=4;8=4"),
         q19 = car::recode(q19,"2=1;3=2;4=2;5=3;6=3;7=4;8=4"))
```

Response categories looks nice when adding together these two items and only having 4 categories.

Let's see how they work together with item 5, although including it will likely lead to item misfit again.

#### Conditional item fit
```{r}
simfit1 <- RIgetfit(d2, iterations = 200, cpu = 8) 

RIitemfit(d2, simfit1)
RIgetfitPlot(simfit1, d2)
```

Although less underfit, item q5 is still an issue. Removing it will leave us with a scale that only has two items which will be to unstable.

```{r}
# Drop item 5
d2$q5 <- NULL
```

#### Conditional item fit
```{r}
simfit1 <- RIgetfit(d2, iterations = 200, cpu = 8) 

RIitemfit(d2, simfit1)
RIgetfitPlot(simfit1, d2)
```
#### Targeting
```{r}
#| fig-height: 5
# increase fig-height above as needed, if you have many items
RItargeting(d2)
```
### Test Information (Reliability)
```{r}
RItif(d2, samplePSI = T)
```

Although there is no longer item misfit, the test information is too low with only these two items.
```
