# Supplementary material for: Psychometric properties of the Swedish cardiac anxiety questionnaire: a Rasch analysis
Source: Sci Rep. 2025 Nov 24;15:41834. doi: 10.1038/s41598-025-28073-8 (PMC12647126; doi:10.1038/s41598-025-28073-8)
Supplement: Supplementary file 1 — Supplementary Material 1 [file 41598_2025_28073_MOESM1_ESM.zip › Supplementary/analysis_drag.html]

CAQ psychometric analysis, model by Dragioti et al. (2011)


# CAQ psychometric analysis, model by Dragioti et al. (2011)

 Code

- Show All Code
- Hide All Code
- ---
- View Source

Using Rasch Measurement Theory

Author

Affiliation

Magnus Johansson; Philip Leissner

RISE Research Institutes of Sweden; Department of women’s and children’s health, Uppsala University

Published

2025-08-28

## Table of contents

- 1 All items in the analysis
- 2 Descriptives - item level
- 3 Dragioti fear version
  - 3.1 Rasch analysis
  - 3.2 Test Information (Reliability)
- 4 Dragioti avoidance version
  - 4.1 Rasch analysis
  - 4.2 Recoding of response categories
  - 4.3 Re-evaluation of DIF
  - 4.4 Test Information (Reliability)
- 5 Dragioti attention version
  - 5.1 Rasch analysis
  - 5.2 Test Information (Reliability)

## Other Formats

- PDF

Code

```
# one package below requires that you use devtools to install them manually:
# first install devtools by
# install.packages('devtools')

library(easyRasch) # devtools::install_github("pgmj/easyRasch")
library(grateful)
library(ggrepel)
library(car)
library(kableExtra)
library(readxl)
library(tidyverse)
library(eRm)
library(iarm)
library(mirt)
library(psych)
library(psychotree)
library(matrixStats)
library(reshape)
library(knitr)
library(patchwork)
library(formattable) 
library(glue)
library(readxl) # for reading excel files

### optional libraries
#library(TAM)
#library(skimr)
#library(janitor)

### some commands exist in multiple packages, here we define preferred ones that are frequently used
select <- dplyr::select
count <- dplyr::count
recode <- car::recode
rename <- dplyr::rename
```

Code

```
### import data - this is just sample code, the files do not exist
df <- read_excel("data/CAQ_Rasch.xlsx") # replace with your datafile as needed

#library(haven) # for SPSS and other formats
#library(labelled) # for getting labels and metadata from SPSS files

### Load item information
# make sure that variable names in df match with itemlabels$itemnr
iteminfo <- read_excel("data/iteminfo.xlsx")

itemlabels <- iteminfo[,1:2]

### Make a backup of the dataframe, in case you need to revert changes at some point
d <- df
```

Code

```
##### Optionally: filter participants based on missing data

##### Before filtering out participants, you should check the missing data structure using RImissing() and RImissingP()

RImissing(d)
```

RImissingP() behöver åtgärdas, så vi gör en manuell koll:

Code

```
d[,6:23] %>% 
  mutate(missing = rowSums(is.na(.))) %>% 
  count(missing)
```

```
# A tibble: 10 × 2
   missing     n
     <dbl> <int>
 1       0   756
 2       1    28
 3       2     8
 4       3     1
 5       4     3
 6       5     2
 7       9     1
 8      10     1
 9      16     1
10      18     5
```

Vi tappar 50 respondenter om vi tar bort alla med missing på något item. 756 är en bra sampelstorlek, så vi kör på detta

Code

```
d <- na.omit(d)
```

2 som hade missing på någon demografisk variabel togs också bort.

Code

```
#---- Create DIF variables----
  
# DIF variables into vectors, recoded as factors since DIF functions need this
# these could also be stored in its own dataframe (not a tibble) instead of as vectors

d$age_cat <- ifelse(d$AGE_R > 74, "older", "younger")

d_dif <- d %>% 
  mutate(sex = factor(SEX),
         age = AGE_R,
         age_cat = factor(age_cat),
         rel_status = factor(RELSTAT_bin),
         born_swe = factor(BORNSWE),
         edu = factor(EDUCATION)) %>% 
  select(sex,age,age_cat, rel_status,born_swe,edu)

# remove DIF variables from item data
d <- d %>% 
  select(starts_with("Item")) %>% 
  set_names(itemlabels$itemnr)


### label gender variable as factor
# dif.gender <- factor(dif.gender,
#                       levels = c(1,2,3),
#                       labels = c("Female", "Male", "Other/missing response"))

# optionally, load RISE ggplot theme and color palettes and set the theme as default.
# just comment out the row below if you desire different theming
source("RISE_theme.R")
```

## 1 All items in the analysis

Code

```
RIlistitems(d)
```

| itemnr | item |
| --- | --- |
| q1 | I pay attention to my heart beat |
| q2 | I avoid physical exertion |
| q3 | My racing heart wakes me up at night |
| q4 | Chest pain/discomfort wakes me up at night |
| q5 | I take it easy as much as possible |
| q6 | I check my pulse |
| q7 | I avoid exercise or other physical work |
| q8 | I can feel my heart in my chest |
| q9 | I avoid activities that make my heart beat faster |
| q10 | If tests come out normal, I still worry about my heart |
| q11 | I feel safe being around a hospital, physician, or other medical facility |
| q12 | I avoid activities that make me sweat |
| q13 | I worry that doctors do not believe my chest pain/discomfort is real |
| q14 | When I have chest discomfort or I feel my heart is beating fast I worry that I may have a heart attack |
| q15 | When I have chest discomfort or I feel my heart is beating fast I have difficulty concentrating on anything else |
| q16 | When I have chest discomfort or I feel my heart is beating fast I get frightened |
| q17 | When I have chest discomfort or I feel my heart is beating fast I like to be checked out by a doctor |
| q18 | When I have chest discomfort or I feel my heart is beating fast I tell my family or friends |

Response distribution for all items are summarized below.

Code

```
RIallresp(d)
```

| Response category | Number of responses | Percent |
| --- | --- | --- |
| 0 | 5359 | 39.5 |
| 1 | 3580 | 26.4 |
| 2 | 2767 | 20.4 |
| 3 | 1244 | 9.2 |
| 4 | 622 | 4.6 |

## 2 Descriptives - item level

Code

```
RIlistItemsMargin(d, fontsize = 12)
```

| itemnr | item |
| --- | --- |
| q1 | I pay attention to my heart beat |
| q2 | I avoid physical exertion |
| q3 | My racing heart wakes me up at night |
| q4 | Chest pain/discomfort wakes me up at night |
| q5 | I take it easy as much as possible |
| q6 | I check my pulse |
| q7 | I avoid exercise or other physical work |
| q8 | I can feel my heart in my chest |
| q9 | I avoid activities that make my heart beat faster |
| q10 | If tests come out normal, I still worry about my heart |
| q11 | I feel safe being around a hospital, physician, or other medical facility |
| q12 | I avoid activities that make me sweat |
| q13 | I worry that doctors do not believe my chest pain/discomfort is real |
| q14 | When I have chest discomfort or I feel my heart is beating fast I worry that I may have a heart attack |
| q15 | When I have chest discomfort or I feel my heart is beating fast I have difficulty concentrating on anything else |
| q16 | When I have chest discomfort or I feel my heart is beating fast I get frightened |
| q17 | When I have chest discomfort or I feel my heart is beating fast I like to be checked out by a doctor |
| q18 | When I have chest discomfort or I feel my heart is beating fast I tell my family or friends |

- Tile plot
- Stacked bars
- Barplots

Code

```
RItileplot(d)
```

Code

```
RIbarstack(d)
```

Code

```
RIbarplot(d)
```

## 3 Dragioti fear version

Code

```
d_all <- d

items_df <- iteminfo %>% 
  filter(dragioti == "yes", factor == "fear") %>% 
  pull(itemnr)

d <- d_all %>% 
  select(all_of(items_df))
```

### 3.1 Rasch analysis

The eRm package, which uses Conditional Maximum Likelihood (CML) estimation, will be used primarily. For this analysis, the Partial Credit Model will be used.

| itemnr | item |
| --- | --- |
| q14 | When I have chest discomfort or I feel my heart is beating fast I worry that I may have a heart attack |
| q15 | When I have chest discomfort or I feel my heart is beating fast I have difficulty concentrating on anything else |
| q16 | When I have chest discomfort or I feel my heart is beating fast I get frightened |
| q17 | When I have chest discomfort or I feel my heart is beating fast I like to be checked out by a doctor |

- Conditional item fit
- Item-restscore
- PCA
- Local dependency
- Residual correlations
- 1st contrast loadings
- Response categories
- Targeting
- Item hierarchy
- Score groups LR-test
- Score groups obs-exp
- Rasch-tree DIF sex
- Rasch-tree DIF age
- Rasch-tree DIF relationship status
- Rasch-tree DIF edu
- Partial gamma DIF immigration status
- Rasch-tree DIF rel\*age
- Person fit
- Floor and ceiling effects

Code

```
#RIitemfit(d, cutoff = "Smith98")

simfit1 <- RIgetfit(d, iterations = 200, cpu = 8) 

RIitemfit(d, simfit1)
```

| Item | InfitMSQ | Infit thresholds | OutfitMSQ | Outfit thresholds | Infit diff | Outfit diff | Relative location |
| --- | --- | --- | --- | --- | --- | --- | --- |
| q14 | 0.91 | [0.917, 1.092] | 0.901 | [0.901, 1.107] | 0.007 | 0 | 1.46 |
| q15 | 0.903 | [0.884, 1.148] | 0.909 | [0.886, 1.174] | no misfit | no misfit | 1.33 |
| q16 | 0.749 | [0.886, 1.109] | 0.769 | [0.889, 1.109] | 0.137 | 0.12 | 1.18 |
| q17 | 1.488 | [0.899, 1.108] | 1.596 | [0.893, 1.122] | 0.38 | 0.474 | 0.52 |
|  |
| --- |
| Note: |
| MSQ values based on conditional calculations (n = 754 complete cases).  Simulation based thresholds from 200 simulated datasets. |

Code

```
RIgetfitPlot(simfit1, d)
```

Code

```
RIrestscore(d)
```

| Item | Observed value | Model expected value | Absolute difference | Adjusted p-value (BH) | Statistical significance level | Location | Relative location |
| --- | --- | --- | --- | --- | --- | --- | --- |
| q14 | 0.70 | 0.65 | 0.05 | 0.045 | \* | 0.34 | 1.46 |
| q15 | 0.70 | 0.66 | 0.04 | 0.052 | . | 0.21 | 1.33 |
| q16 | 0.76 | 0.66 | 0.10 | 0.000 | \*\*\* | 0.05 | 1.18 |
| q17 | 0.55 | 0.67 | 0.12 | 0.000 | \*\*\* | -0.60 | 0.52 |

Code

```
RIpcmPCA(d)
```

PCA of Rasch model residuals

| Eigenvalues | Proportion of variance |
| --- | --- |
| 1.69 | 50.5% |
| 1.26 | 28% |
| 1.04 | 21.4% |
| 0.01 | 0.2% |

Code

```
# using partial gamma LD from library(iarm)
RIpartgamLD(d)
```

| Item 1 | Item 2 | Partial gamma | SE | Lower CI | Upper CI | Adjusted p-value (BH) |
| --- | --- | --- | --- | --- | --- | --- |
| q16 | q15 | 0.491 | 0.061 | 0.372 | 0.610 | 0.000 |
| q15 | q16 | 0.375 | 0.065 | 0.247 | 0.503 | 0.000 |
| q15 | q14 | 0.212 | 0.071 | 0.073 | 0.350 | 0.033 |
| q16 | q14 | 0.212 | 0.074 | 0.067 | 0.357 | 0.050 |
| q14 | q15 | 0.208 | 0.071 | 0.069 | 0.348 | 0.042 |

Code

```
simcor1 <- RIgetResidCor(d, iterations = 250, cpu = 8)
RIresidcorr(d, cutoff = simcor1$p99)
```

|  | q14 | q15 | q16 | q17 |
| --- | --- | --- | --- | --- |
| q14 |  |  |  |  |
| q15 | -0.18 |  |  |  |
| q16 | -0.18 | 0.03 |  |  |
| q17 | -0.37 | -0.52 | -0.41 |  |
|  |
| --- |
| Note: |
| Relative cut-off value is -0.151, which is 0.123 above the average correlation (-0.273).  Correlations above the cut-off are highlighted in red text. |

Code

```
RIloadLoc(d)
```

Code

```
mirt(d, model=1, itemtype='Rasch', verbose = FALSE) %>% 
  plot(type="trace", as.table = TRUE, 
       theta_lim = c(-6,6))
```

Code

```
# for fewer items or a more magnified figure, use:
#RIitemCats(d)
```

Code

```
# increase fig-height above as needed, if you have many items
RItargeting(d)
```

Code

```
RIitemHierarchy(d)
```

Code

```
iarm::score_groups(as.data.frame(d)) %>% 
  as.data.frame(nm = "score_group") %>% 
  dplyr::count(score_group)
```

```
  score_group   n
1           1 436
2           2 318
```

Code

```
dif_plots <- d %>% 
  add_column(dif = iarm::score_groups(.)) %>% 
  split(.$dif) %>% # split the data using the DIF variable
  map(~ RItileplot(.x %>% dplyr::select(!dif)) + labs(title = .x$dif))
dif_plots[[1]] + dif_plots[[2]]
```

Code

```
clr_tests(d, model = "PCM")
```

```
Conditional Likelihood Ratio Tests:
```

```
        clr df pvalue  sig 
overall 111 15 1.1e-16  ***
```

Code

```
item_obsexp(PCM(d))
```

```
Score group 1: 
    mean obs mean exp std.res sig
q14  0.608    0.619   -0.343     
q15  0.708    0.725   -0.543     
q16  0.789    0.827   -1.183     
q17  1.129    1.032    2.859  +  

Score group 2: 
    mean obs mean exp std.res sig
q14  1.924    1.911    0.287     
q15  2.102    2.082    0.446     
q16  2.159    2.086    1.730     
q17  2.471    2.577   -2.271  -
```

Code

```
RIdifTable(d, d_dif$sex)
```

| Item | 2 | 3 | Mean location | StDev | MaxDiff |
| --- | --- | --- | --- | --- | --- |
| q14 | 0.366 | 0.306 | 0.336 | 0.043 | 0.060 |
| q15 | 0.211 | 0.201 | 0.206 | 0.007 | 0.009 |
| q16 | -0.183 | 0.161 | -0.011 | 0.243 | 0.343 |
| q17 | -0.394 | -0.668 | -0.531 | 0.194 | 0.274 |

Code

```
RIdifTable(d, d_dif$age)
```

| Item | 2 | 3 | Mean location | StDev | MaxDiff |
| --- | --- | --- | --- | --- | --- |
| q14 | 0.386 | 0.251 | 0.318 | 0.095 | 0.134 |
| q15 | 0.102 | 0.434 | 0.268 | 0.235 | 0.332 |
| q16 | -0.049 | 0.216 | 0.083 | 0.187 | 0.265 |
| q17 | -0.438 | -0.901 | -0.670 | 0.327 | 0.463 |

Code

```
RIdifTable(d, d_dif$rel_status)
```

| Item | 2 | 3 | Mean location | StDev | MaxDiff |
| --- | --- | --- | --- | --- | --- |
| q14 | 0.379 | 0.175 | 0.277 | 0.145 | 0.204 |
| q15 | 0.227 | 0.132 | 0.180 | 0.067 | 0.095 |
| q16 | 0.080 | -0.055 | 0.013 | 0.095 | 0.135 |
| q17 | -0.687 | -0.252 | -0.470 | 0.307 | 0.434 |

Code

```
RIdifTable(d, d_dif$edu)
```

| Item | 2 | 3 | Mean location | StDev | MaxDiff |
| --- | --- | --- | --- | --- | --- |
| q14 | 0.276 | 0.469 | 0.372 | 0.136 | 0.193 |
| q15 | 0.242 | 0.139 | 0.191 | 0.072 | 0.103 |
| q16 | 0.113 | -0.062 | 0.026 | 0.124 | 0.175 |
| q17 | -0.631 | -0.546 | -0.589 | 0.060 | 0.085 |

Code

```
# using partial gamma from library(iarm)
RIpartgamDIF(d, d_dif$born_swe)
```

```
[1] "No statistically significant DIF found."
```

Code

```
RIdifTable2(d, d_dif$rel_status, d_dif$sex)
```

| Item | 2 | 4 | 5 | Mean location | StDev | MaxDiff |
| --- | --- | --- | --- | --- | --- | --- |
| q14 | 0.366 | 0.379 | -0.060 | 0.228 | 0.250 | 0.439 |
| q15 | 0.211 | 0.195 | 0.240 | 0.215 | 0.023 | 0.045 |
| q16 | -0.183 | 0.161 | 0.146 | 0.041 | 0.194 | 0.343 |
| q17 | -0.394 | -0.734 | -0.326 | -0.485 | 0.218 | 0.408 |

Code

```
RIpfit(d)
```

Code

```
RItif(d, samplePSI = T, cutoff = 1)
```

Item q17 is clearly misfit and some residual correlations between item q15 and q16.

There is statistically significant DIF for age, sex, education and relationship status but nothing exceeding 0.5.

Category thresholds works for all items, except for the highest categories of item q17.

This analysis suggest that the fear subscale from the 10-item version by Dragioti would be better without item q17. That woudl result in exactly the same version of the subscale that was derived from the Rasch analysis based on the original, 18-item, version.

### 3.2 Test Information (Reliability)

Code

```
RItif(d, samplePSI = T)
```

However, test information 3.33 (PSI = 0.7) is reached between 0.5 and 1.25 logits, where 9.5% of the participants are located. This is better than any other version of the fear scale.

## 4 Dragioti avoidance version

Code

```
items_dav <- iteminfo %>% 
  filter(dragioti == "yes", factor == "avoidance") %>% 
  pull(itemnr)

d <- d_all %>% 
  select(all_of(items_dav))
```

### 4.1 Rasch analysis

The eRm package, which uses Conditional Maximum Likelihood (CML) estimation, will be used primarily. For this analysis, the Partial Credit Model will be used.

| itemnr | item |
| --- | --- |
| q2 | I avoid physical exertion |
| q7 | I avoid exercise or other physical work |
| q12 | I avoid activities that make me sweat |

- Conditional item fit
- Item-restscore
- PCA
- Local dependency
- Residual correlations
- 1st contrast loadings
- Response categories
- Targeting
- Item hierarchy
- Score groups LR-test
- Score groups obs-exp
- Rasch-tree DIF sex
- Rasch-tree DIF age
- Rasch-tree DIF relationship status
- Rasch-tree DIF edu
- Partial gamma DIF immigration status
- Person fit
- Floor and ceiling effects

Code

```
#RIitemfit(d, cutoff = "Smith98")

simfit1 <- RIgetfit(d, iterations = 200, cpu = 8) 

RIitemfit(d, simfit1)
```

| Item | InfitMSQ | Infit thresholds | OutfitMSQ | Outfit thresholds | Infit diff | Outfit diff | Relative location |
| --- | --- | --- | --- | --- | --- | --- | --- |
| q2 | 0.952 | [0.857, 1.093] | 0.948 | [0.851, 1.12] | no misfit | no misfit | 1.61 |
| q7 | 0.952 | [0.919, 1.095] | 0.95 | [0.923, 1.083] | no misfit | no misfit | 1.60 |
| q12 | 1.136 | [0.917, 1.1] | 1.072 | [0.923, 1.093] | 0.036 | no misfit | 2.14 |
|  |
| --- |
| Note: |
| MSQ values based on conditional calculations (n = 754 complete cases).  Simulation based thresholds from 200 simulated datasets. |

Code

```
RIgetfitPlot(simfit1, d)
```

Code

```
RIrestscore(d)
```

| Item | Observed value | Model expected value | Absolute difference | Adjusted p-value (BH) | Statistical significance level | Location | Relative location |
| --- | --- | --- | --- | --- | --- | --- | --- |
| q2 | 0.75 | 0.72 | 0.03 | 0.469 |  | -0.17 | 1.61 |
| q7 | 0.74 | 0.72 | 0.02 | 0.469 |  | -0.18 | 1.60 |
| q12 | 0.70 | 0.71 | 0.01 | 0.568 |  | 0.36 | 2.14 |

Code

```
RIpcmPCA(d)
```

PCA of Rasch model residuals

| Eigenvalues | Proportion of variance |
| --- | --- |
| 1.54 | 52.8% |
| 1.45 | 46.8% |
| 0.01 | 0.3% |

Code

```
# using partial gamma LD from library(iarm)
RIpartgamLD(d)
```

| Item 1 | Item 2 | Partial gamma | SE | Lower CI | Upper CI | Adjusted p-value (BH) |
| --- | --- | --- | --- | --- | --- | --- |
| q7 | q2 | 0.284 | 0.085 | 0.118 | 0.450 | 0.005 |
| q2 | q7 | 0.242 | 0.088 | 0.070 | 0.415 | 0.035 |

Code

```
simcor1 <- RIgetResidCor(d, iterations = 250, cpu = 8)
RIresidcorr(d, cutoff = simcor1$p99)
```

|  | q2 | q7 | q12 |
| --- | --- | --- | --- |
| q2 |  |  |  |
| q7 | -0.31 |  |  |
| q12 | -0.44 | -0.49 |  |
|  |
| --- |
| Note: |
| Relative cut-off value is -0.308, which is 0.105 above the average correlation (-0.414).  Correlations above the cut-off are highlighted in red text. |

Code

```
RIloadLoc(d)
```

Code

```
mirt(d, model=1, itemtype='Rasch', verbose = FALSE) %>% 
  plot(type="trace", as.table = TRUE, 
       theta_lim = c(-6,6))
```

Code

```
# for fewer items or a more magnified figure, use:
#RIitemCats(d)
```

Code

```
# increase fig-height above as needed, if you have many items
RItargeting(d)
```

Code

```
RIitemHierarchy(d)
```

Code

```
iarm::score_groups(as.data.frame(d)) %>% 
  as.data.frame(nm = "score_group") %>% 
  dplyr::count(score_group)
```

```
  score_group   n
1           1 450
2           2 304
```

Code

```
dif_plots <- d %>% 
  add_column(dif = iarm::score_groups(.)) %>% 
  split(.$dif) %>% # split the data using the DIF variable
  map(~ RItileplot(.x %>% dplyr::select(!dif)) + labs(title = .x$dif))
dif_plots[[1]] + dif_plots[[2]]
```

Code

```
clr_tests(d, model = "PCM")
```

```
Conditional Likelihood Ratio Tests:
```

```
        clr  df pvalue sig 
overall 58.2 11 2e-08   ***
```

Code

```
item_obsexp(PCM(d))
```

```
Score group 1: 
    mean obs mean exp std.res sig
q2   0.932    0.962   -0.955     
q7   0.725    0.748   -0.687     
q12  0.410    0.357    1.836     

Score group 2: 
    mean obs mean exp std.res sig
q2   2.086    2.057    0.813     
q7   2.136    2.114    0.583     
q12  1.684    1.735   -1.309
```

Code

```
RIdifTable(d, d_dif$sex)
```

```
[1] "No statistically significant DIF found."
```

Code

```
RIdifTable(d, d_dif$age)
```

| Item | 2 | 3 | Mean location | StDev | MaxDiff |
| --- | --- | --- | --- | --- | --- |
| q2 | -0.152 | -0.232 | -0.192 | 0.057 | 0.080 |
| q7 | -0.332 | 0.244 | -0.044 | 0.408 | 0.576 |
| q12 | 0.484 | -0.013 | 0.236 | 0.351 | 0.496 |

Code

```
RIdifTable(d, d_dif$rel_status)
```

| Item | 2 | 3 | Mean location | StDev | MaxDiff |
| --- | --- | --- | --- | --- | --- |
| q2 | -0.106 | -0.212 | -0.159 | 0.075 | 0.106 |
| q7 | -0.326 | 0.059 | -0.134 | 0.272 | 0.385 |
| q12 | 0.431 | 0.153 | 0.292 | 0.197 | 0.278 |

Code

```
RIdifTable(d, d_dif$edu)
```

```
[1] "No statistically significant DIF found."
```

Code

```
# using partial gamma from library(iarm)
RIpartgamDIF(d, d_dif$born_swe)
```

```
[1] "No statistically significant DIF found."
```

Code

```
RIpfit(d)
```

Code

```
RItif(d, samplePSI = T, cutoff = 1)
```

This version of the avoidance subscale only shows a slight underfitting on item q12 and no residual correlations above the relative cut-off.

Response categories all are working well.

Statistically significant DIF was found for age and relationship status, and the DIF for age was above the cut-off of 0.5.

Let’s inspect the category thresholds for DIF.

- LR DIF Table
- LR DIF Figure
- LR Test

Code

```
RIdifThreshTblLR(d, d_dif$age_cat)
```

|  | Threshold locations | | | | Standard errors | | |
| --- | --- | --- | --- | --- | --- | --- | --- |
| Item threshold | older | younger | MaxDiff | All | SE\_older | SE\_younger | SE\_All |
| **q2** | | | | | | | |
| c1 | -2.184 | -2.586 | 0.402 | -2.440 | 0.317 | 0.171 | 0.148 |
| c2 | -0.188 | -0.158 | 0.03 | -0.153 | 0.253 | 0.130 | 0.114 |
| c3 | 1.936 | 1.446 | 0.49 | 1.504 | 0.426 | 0.182 | 0.164 |
| c4 | 2.128 | 4.17 | 2.042 | 3.568 | 0.701 | 0.492 | 0.385 |
| **q7** | | | | | | | |
| c1 | -0.896 | -1.948 | 1.052 | -1.666 | 0.272 | 0.156 | 0.132 |
| c2 | -0.438 | -0.159 | 0.279 | -0.194 | 0.280 | 0.137 | 0.122 |
| c3 | 2.061 | 1.244 | 0.817 | 1.371 | 0.432 | 0.181 | 0.165 |
| c4 | 2.869 | 3.013 | 0.144 | 2.915 | 0.856 | 0.347 | 0.315 |
| **q12** | | | | | | | |
| c1 | -0.865 | -0.618 | 0.247 | -0.646 | 0.261 | 0.127 | 0.113 |
| c2 | 0.031 | 0.44 | 0.409 | 0.369 | 0.305 | 0.154 | 0.136 |
| c3 | 1.176 | 2.085 | 0.909 | 1.849 | 0.406 | 0.239 | 0.203 |
| c4 | 2.225 | 3.507 | 1.282 | 3.010 | 0.618 | 0.498 | 0.376 |
|  |
| --- |
| Note: |
| Values highlighted in red are above the chosen cutoff 0.5 logits. Background color brown and blue indicate the lowest and highest values among the DIF groups. |

Code

```
RIdifThreshFigLR(d, d_dif$age_cat)
```

Code

```
LRtest(PCM(d), d_dif$age_cat)
```

```
Andersen LR-test: 
LR-value: 36.032 
Chi-square df: 11 
p-value:  0
```

Looks like older participants (>74 years) barely discriminate between the two highest categories on item q2, and the two lower categories on item q7. Item q12 seems to, on average, have lower thresholds for older participants.

### 4.2 Recoding of response categories

Code

```
d %>% 
  mutate(q7 = car::recode(q7,"1=0;2=1;3=2;4=2")) %>% 
  RItileplot()
```

Code

```
d2 <- d %>% 
  mutate(q7 = car::recode(q7,"1=0;2=1;3=2;4=2"))
```

### 4.3 Re-evaluation of DIF

- Rasch-tree DIF age
- LR DIF Table
- LR DIF Figure
- LR Test

Code

```
RIdifTable(d2, d_dif$age)
```

| Item | 2 | 3 | Mean location | StDev | MaxDiff |
| --- | --- | --- | --- | --- | --- |
| q2 | -0.191 | -0.257 | -0.224 | 0.047 | 0.066 |
| q7 | -0.297 | 0.238 | -0.029 | 0.378 | 0.535 |
| q12 | 0.487 | 0.019 | 0.253 | 0.331 | 0.468 |

Code

```
RIdifThreshTblLR(d2, d_dif$age_cat)
```

|  | Threshold locations | | | | Standard errors | | |
| --- | --- | --- | --- | --- | --- | --- | --- |
| Item threshold | older | younger | MaxDiff | All | SE\_older | SE\_younger | SE\_All |
| **q2** | | | | | | | |
| c1 | -2.347 | -2.911 | 0.564 | -2.743 | 0.341 | 0.201 | 0.172 |
| c2 | -0.196 | -0.359 | 0.163 | -0.294 | 0.262 | 0.136 | 0.119 |
| c3 | 2.097 | 1.384 | 0.713 | 1.500 | 0.431 | 0.185 | 0.168 |
| c4 | 2.246 | 4.08 | 1.834 | 3.521 | 0.701 | 0.484 | 0.380 |
| **q7** | | | | | | | |
| c1 | 0.162 | -0.1 | 0.262 | -0.016 | 0.242 | 0.131 | 0.114 |
| c2 | 1.934 | 0.997 | 0.937 | 1.160 | 0.425 | 0.181 | 0.164 |
| **q12** | | | | | | | |
| c1 | -0.996 | -0.862 | 0.134 | -0.850 | 0.272 | 0.132 | 0.117 |
| c2 | 0.082 | 0.282 | 0.2 | 0.274 | 0.317 | 0.158 | 0.140 |
| c3 | 1.349 | 2.113 | 0.764 | 1.884 | 0.411 | 0.249 | 0.207 |
| c4 | 2.319 | 3.449 | 1.13 | 3.015 | 0.617 | 0.494 | 0.377 |
|  |
| --- |
| Note: |
| Values highlighted in red are above the chosen cutoff 0.5 logits. Background color brown and blue indicate the lowest and highest values among the DIF groups. |

Code

```
RIdifThreshFigLR(d2, d_dif$age_cat)
```

Code

```
LRtest(PCM(d2), d_dif$age_cat)
```

```
Andersen LR-test: 
LR-value: 25.331 
Chi-square df: 9 
p-value:  0.003
```

Recoding of response categories did not solve the problem of DIF.

### 4.4 Test Information (Reliability)

Code

```
RItif(d, samplePSI = T)
```

Test information is not above 3.33 on any part of the scale. Reliability is low.

## 5 Dragioti attention version

Code

```
items_dat <- iteminfo %>% 
  filter(dragioti == "yes", factor == "attention") %>% 
  pull(itemnr)

d <- d_all %>% 
  select(all_of(items_dat))
```

### 5.1 Rasch analysis

The eRm package, which uses Conditional Maximum Likelihood (CML) estimation, will be used primarily. For this analysis, the Partial Credit Model will be used.

| itemnr | item |
| --- | --- |
| q3 | My racing heart wakes me up at night |
| q4 | Chest pain/discomfort wakes me up at night |
| q8 | I can feel my heart in my chest |

- Conditional item fit
- Item-restscore
- PCA
- Local dependency
- Residual correlations
- 1st contrast loadings
- Response categories
- Targeting
- Item hierarchy
- Score groups LR-test
- Score groups obs-exp
- Rasch-tree DIF sex
- Rasch-tree DIF age
- Rasch-tree DIF relationship status
- Rasch-tree DIF edu
- Partial gamma DIF immigration status
- Person fit
- Floor and ceiling effects

Code

```
#RIitemfit(d, cutoff = "Smith98")

simfit1 <- RIgetfit(d, iterations = 200, cpu = 8) 

RIitemfit(d, simfit1)
```

| Item | InfitMSQ | Infit thresholds | OutfitMSQ | Outfit thresholds | Infit diff | Outfit diff | Relative location |
| --- | --- | --- | --- | --- | --- | --- | --- |
| q3 | 0.702 | [0.844, 1.14] | 0.547 | [0.803, 1.185] | 0.142 | 0.256 | 2.00 |
| q4 | 1.007 | [0.891, 1.098] | 1.039 | [0.867, 1.117] | no misfit | no misfit | 1.95 |
| q8 | 1.318 | [0.906, 1.093] | 1.166 | [0.95, 1.057] | 0.225 | 0.109 | 0.80 |
|  |
| --- |
| Note: |
| MSQ values based on conditional calculations (n = 754 complete cases).  Simulation based thresholds from 200 simulated datasets. |

Code

```
RIgetfitPlot(simfit1, d)
```

Code

```
RIrestscore(d)
```

| Item | Observed value | Model expected value | Absolute difference | Adjusted p-value (BH) | Statistical significance level | Location | Relative location |
| --- | --- | --- | --- | --- | --- | --- | --- |
| q3 | 0.74 | 0.50 | 0.24 | 0.000 | \*\*\* | 0.42 | 2.00 |
| q4 | 0.51 | 0.51 | 0.00 | 0.934 |  | 0.36 | 1.95 |
| q8 | 0.40 | 0.52 | 0.12 | 0.007 | \*\* | -0.78 | 0.80 |

Code

```
RIpcmPCA(d)
```

PCA of Rasch model residuals

| Eigenvalues | Proportion of variance |
| --- | --- |
| 1.91 | 72.6% |
| 1.05 | 26% |
| 0.04 | 1.4% |

Code

```
# using partial gamma LD from library(iarm)
RIpartgamLD(d)
```

| Item 1 | Item 2 | Partial gamma | SE | Lower CI | Upper CI | Adjusted p-value (BH) |
| --- | --- | --- | --- | --- | --- | --- |
| q3 | q4 | 0.731 | 0.052 | 0.630 | 0.832 | 0 |
| q4 | q3 | 0.499 | 0.072 | 0.357 | 0.641 | 0 |
| q3 | q8 | 0.432 | 0.096 | 0.244 | 0.620 | 0 |

Code

```
simcor1 <- RIgetResidCor(d, iterations = 250, cpu = 8)
RIresidcorr(d, cutoff = simcor1$p99)
```

|  | q3 | q4 | q8 |
| --- | --- | --- | --- |
| q3 |  |  |  |
| q4 | 0.11 |  |  |
| q8 | -0.28 | -0.48 |  |
|  |
| --- |
| Note: |
| Relative cut-off value is -0.096, which is 0.118 above the average correlation (-0.214).  Correlations above the cut-off are highlighted in red text. |

Code

```
RIloadLoc(d)
```

Code

```
mirt(d, model=1, itemtype='Rasch', verbose = FALSE) %>% 
  plot(type="trace", as.table = TRUE, 
       theta_lim = c(-6,6))
```

Code

```
# for fewer items or a more magnified figure, use:
#RIitemCats(d)
```

Code

```
# increase fig-height above as needed, if you have many items
RItargeting(d)
```

Code

```
RIitemHierarchy(d)
```

Code

```
iarm::score_groups(as.data.frame(d)) %>% 
  as.data.frame(nm = "score_group") %>% 
  dplyr::count(score_group)
```

```
  score_group   n
1           1 507
2           2 247
```

Code

```
dif_plots <- d %>% 
  add_column(dif = iarm::score_groups(.)) %>% 
  split(.$dif) %>% # split the data using the DIF variable
  map(~ RItileplot(.x %>% dplyr::select(!dif)) + labs(title = .x$dif))
dif_plots[[1]] + dif_plots[[2]]
```

Code

```
clr_tests(d, model = "PCM")
```

```
Conditional Likelihood Ratio Tests:
```

```
        clr  df pvalue sig
overall 28.6 11 0.0026  **
```

Code

```
item_obsexp(PCM(d))
```

```
Score group 1: 
   mean obs mean exp std.res sig
q3  0.0469   0.1004  -3.0746 -- 
q4  0.2000   0.1829   0.7418    
q8  1.2500   1.2135   1.3418    

Score group 2: 
   mean obs mean exp std.res sig
q3  0.818    0.748    1.385     
q4  1.134    1.156   -0.423     
q8  2.291    2.339   -0.942
```

Code

```
RIdifTable(d, d_dif$sex)
```

```
[1] "No statistically significant DIF found."
```

Code

```
RIdifTable(d, d_dif$age)
```

| Item | 2 | 3 | Mean location | StDev | MaxDiff |
| --- | --- | --- | --- | --- | --- |
| q3 | 0.323 | 0.761 | 0.542 | 0.310 | 0.438 |
| q4 | 0.375 | 0.264 | 0.320 | 0.078 | 0.111 |
| q8 | -0.698 | -1.025 | -0.861 | 0.231 | 0.327 |

Code

```
RIdifTable(d, d_dif$rel_status)
```

```
[1] "No statistically significant DIF found."
```

Code

```
RIdifTable(d, d_dif$edu)
```

```
[1] "No statistically significant DIF found."
```

Code

```
# using partial gamma from library(iarm)
RIpartgamDIF(d, d_dif$born_swe)
```

```
[1] "No statistically significant DIF found."
```

Code

```
RIpfit(d)
```

Code

```
RItif(d, samplePSI = T, cutoff = 1)
```

It is quite clearly problematic to include both items q3 and q4 in a scale with other items. The item mistfit and residual correlations indicate this.

The items themselves are also problematic with poor ordering of category thresholds.

If any of these items should be included in the attention subscale, only one of them is enough.

### 5.2 Test Information (Reliability)

Code

```
RItif(d, samplePSI = T)
```

Test information 3.33 (PSI = 0.7) is reached between 0.15 and 1.2 logits, where 5.6% of the participants are located.

## Reuse

CC BY 4.0

##### Source Code

```
---
title: "CAQ psychometric analysis, model by Dragioti et al. (2011)"
subtitle: "Using Rasch Measurement Theory"
title-block-banner: "#009ca6"
title-block-banner-color: "#FFFFFF"
author: 
  name: Magnus Johansson; Philip Leissner
  affiliation: RISE Research Institutes of Sweden; Department of women's and children's health, Uppsala University
  affiliation-url: https://www.ri.se/en/kbm; https://www.uu.se/en/department/womens-and-childrens-health
  orcid: 0000-0003-1669-592X; 0000-0003-0787-9102
date: last-modified
date-format: iso
always_allow_html: true
format: 
  html:
    toc: true
    toc-depth: 3
    toc-title: "Table of contents"
    embed-resources: true
    standalone: true
    page-layout: full
    mainfont: 'Lato'
    monofont: 'Roboto Mono'
    code-overflow: wrap
    code-fold: true
    code-tools: true
    code-link: true
    number-sections: true
    fig-dpi: 96
    layout-align: left
    linestretch: 1.6
    theme:
      - materia
      - custom.scss
    css: styles.css
    license: CC BY
  pdf:
    papersize: a4
    documentclass: report 
execute:
  echo: true
  warning: false
  message: false
  cache: true
editor_options: 
  markdown: 
    wrap: 72
  chunk_output_type: console
---

```{r}
#| label: setup

# one package below requires that you use devtools to install them manually:
# first install devtools by
# install.packages('devtools')

library(easyRasch) # devtools::install_github("pgmj/easyRasch")
library(grateful)
library(ggrepel)
library(car)
library(kableExtra)
library(readxl)
library(tidyverse)
library(eRm)
library(iarm)
library(mirt)
library(psych)
library(psychotree)
library(matrixStats)
library(reshape)
library(knitr)
library(patchwork)
library(formattable) 
library(glue)
library(readxl) # for reading excel files

### optional libraries
#library(TAM)
#library(skimr)
#library(janitor)

### some commands exist in multiple packages, here we define preferred ones that are frequently used
select <- dplyr::select
count <- dplyr::count
recode <- car::recode
rename <- dplyr::rename
```


```{r}
### import data - this is just sample code, the files do not exist
df <- read_excel("data/CAQ_Rasch.xlsx") # replace with your datafile as needed

#library(haven) # for SPSS and other formats
#library(labelled) # for getting labels and metadata from SPSS files

### Load item information
# make sure that variable names in df match with itemlabels$itemnr
iteminfo <- read_excel("data/iteminfo.xlsx")

itemlabels <- iteminfo[,1:2]

### Make a backup of the dataframe, in case you need to revert changes at some point
d <- df
```

```{r}
##### Optionally: filter participants based on missing data

##### Before filtering out participants, you should check the missing data structure using RImissing() and RImissingP()

RImissing(d)
```

RImissingP() behöver åtgärdas, så vi gör en manuell koll:

```{r}
d[,6:23] %>% 
  mutate(missing = rowSums(is.na(.))) %>% 
  count(missing)
```

Vi tappar 50 respondenter om vi tar bort alla med missing på något item. 756 är en bra sampelstorlek, så vi kör på detta

```{r}
d <- na.omit(d)
```

2 som hade missing på någon demografisk variabel togs också bort.

```{r}
#---- Create DIF variables----
  
# DIF variables into vectors, recoded as factors since DIF functions need this
# these could also be stored in its own dataframe (not a tibble) instead of as vectors

d$age_cat <- ifelse(d$AGE_R > 74, "older", "younger")

d_dif <- d %>% 
  mutate(sex = factor(SEX),
         age = AGE_R,
         age_cat = factor(age_cat),
         rel_status = factor(RELSTAT_bin),
         born_swe = factor(BORNSWE),
         edu = factor(EDUCATION)) %>% 
  select(sex,age,age_cat, rel_status,born_swe,edu)

# remove DIF variables from item data
d <- d %>% 
  select(starts_with("Item")) %>% 
  set_names(itemlabels$itemnr)


### label gender variable as factor
# dif.gender <- factor(dif.gender,
#                       levels = c(1,2,3),
#                       labels = c("Female", "Male", "Other/missing response"))

# optionally, load RISE ggplot theme and color palettes and set the theme as default.
# just comment out the row below if you desire different theming
source("RISE_theme.R")
```

## All items in the analysis
```{r}
RIlistitems(d)
```

Response distribution for all items are summarized below.

```{r}
#| tbl-cap: "Total number of responses for all items"
RIallresp(d)
```

## Descriptives - item level

```{r}
#| column: margin
RIlistItemsMargin(d, fontsize = 12)
```

::: panel-tabset
### Tile plot
```{r}
RItileplot(d)
```
### Stacked bars
```{r}
RIbarstack(d)
```
### Barplots
```{r}
#| layout-ncol: 2
RIbarplot(d)
```
:::

## Dragioti fear version
```{r}
d_all <- d

items_df <- iteminfo %>% 
  filter(dragioti == "yes", factor == "fear") %>% 
  pull(itemnr)

d <- d_all %>% 
  select(all_of(items_df))
```


### Rasch analysis

The eRm package, which uses Conditional Maximum Likelihood (CML)
estimation, will be used primarily. For this analysis, the Partial
Credit Model will be used.

```{r}
#| column: margin
#| echo: false
RIlistItemsMargin(d, fontsize = 13)
```

::: panel-tabset
#### Conditional item fit
```{r}
#RIitemfit(d, cutoff = "Smith98")

simfit1 <- RIgetfit(d, iterations = 200, cpu = 8) 

RIitemfit(d, simfit1)
RIgetfitPlot(simfit1, d)
```
#### Item-restscore
```{r}
RIrestscore(d)
```
#### PCA
```{r}
#| tbl-cap: "PCA of Rasch model residuals"
RIpcmPCA(d)
```
#### Local dependency
```{r}
# using partial gamma LD from library(iarm)
RIpartgamLD(d)
```

#### Residual correlations
```{r}
simcor1 <- RIgetResidCor(d, iterations = 250, cpu = 8)
RIresidcorr(d, cutoff = simcor1$p99)
```
#### 1st contrast loadings
```{r}
RIloadLoc(d)
```
#### Response categories
```{r}
mirt(d, model=1, itemtype='Rasch', verbose = FALSE) %>% 
  plot(type="trace", as.table = TRUE, 
       theta_lim = c(-6,6))
# for fewer items or a more magnified figure, use:
#RIitemCats(d)
```
#### Targeting
```{r}
#| fig-height: 5
# increase fig-height above as needed, if you have many items
RItargeting(d)
```
#### Item hierarchy
```{r}
#| fig-height: 5
RIitemHierarchy(d)
```
#### Score groups LR-test
```{r}
iarm::score_groups(as.data.frame(d)) %>% 
  as.data.frame(nm = "score_group") %>% 
  dplyr::count(score_group)

dif_plots <- d %>% 
  add_column(dif = iarm::score_groups(.)) %>% 
  split(.$dif) %>% # split the data using the DIF variable
  map(~ RItileplot(.x %>% dplyr::select(!dif)) + labs(title = .x$dif))
dif_plots[[1]] + dif_plots[[2]]

clr_tests(d, model = "PCM")
```
#### Score groups obs-exp
```{r}
item_obsexp(PCM(d))
```
#### Rasch-tree DIF sex
```{r}
RIdifTable(d, d_dif$sex)
```
#### Rasch-tree DIF age
```{r}
RIdifTable(d, d_dif$age)
```
#### Rasch-tree DIF relationship status
```{r}
RIdifTable(d, d_dif$rel_status)
```
#### Rasch-tree DIF edu
```{r}
RIdifTable(d, d_dif$edu)
```
#### Partial gamma DIF immigration status
```{r}
# using partial gamma from library(iarm)
RIpartgamDIF(d, d_dif$born_swe)
```
#### Rasch-tree DIF rel*age
```{r}
RIdifTable2(d, d_dif$rel_status, d_dif$sex)
```
#### Person fit
```{r}
RIpfit(d)
```
#### Floor and ceiling effects
```{r}
RItif(d, samplePSI = T, cutoff = 1)
```
:::

Item q17 is clearly misfit and some residual correlations between item q15 and q16.    

There is statistically significant DIF for age, sex, education and relationship status but nothing exceeding 0.5.

Category thresholds works for all items, except for the highest categories of item q17.

This analysis suggest that the fear subscale from the 10-item version by Dragioti would be better without item q17. That woudl result in exactly the same version of the subscale that was derived from the Rasch analysis based on the original, 18-item, version.

### Test Information (Reliability)
```{r}
RItif(d, samplePSI = T)
```

However, test information 3.33 (PSI = 0.7) is reached between 0.5 and 1.25 logits, where 9.5% of the participants are located. This is better than any other version of the fear scale.

## Dragioti avoidance version
```{r}
items_dav <- iteminfo %>% 
  filter(dragioti == "yes", factor == "avoidance") %>% 
  pull(itemnr)

d <- d_all %>% 
  select(all_of(items_dav))
```


### Rasch analysis

The eRm package, which uses Conditional Maximum Likelihood (CML)
estimation, will be used primarily. For this analysis, the Partial
Credit Model will be used.

```{r}
#| column: margin
#| echo: false
RIlistItemsMargin(d, fontsize = 13)
```

::: panel-tabset
#### Conditional item fit
```{r}
#RIitemfit(d, cutoff = "Smith98")

simfit1 <- RIgetfit(d, iterations = 200, cpu = 8) 

RIitemfit(d, simfit1)
RIgetfitPlot(simfit1, d)
```
#### Item-restscore
```{r}
RIrestscore(d)
```
#### PCA
```{r}
#| tbl-cap: "PCA of Rasch model residuals"
RIpcmPCA(d)
```
#### Local dependency
```{r}
# using partial gamma LD from library(iarm)
RIpartgamLD(d)
```

#### Residual correlations
```{r}
simcor1 <- RIgetResidCor(d, iterations = 250, cpu = 8)
RIresidcorr(d, cutoff = simcor1$p99)
```
#### 1st contrast loadings
```{r}
RIloadLoc(d)
```
#### Response categories
```{r}
mirt(d, model=1, itemtype='Rasch', verbose = FALSE) %>% 
  plot(type="trace", as.table = TRUE, 
       theta_lim = c(-6,6))
# for fewer items or a more magnified figure, use:
#RIitemCats(d)
```
#### Targeting
```{r}
#| fig-height: 5
# increase fig-height above as needed, if you have many items
RItargeting(d)
```
#### Item hierarchy
```{r}
#| fig-height: 5
RIitemHierarchy(d)
```
#### Score groups LR-test
```{r}
iarm::score_groups(as.data.frame(d)) %>% 
  as.data.frame(nm = "score_group") %>% 
  dplyr::count(score_group)

dif_plots <- d %>% 
  add_column(dif = iarm::score_groups(.)) %>% 
  split(.$dif) %>% # split the data using the DIF variable
  map(~ RItileplot(.x %>% dplyr::select(!dif)) + labs(title = .x$dif))
dif_plots[[1]] + dif_plots[[2]]

clr_tests(d, model = "PCM")
```
#### Score groups obs-exp
```{r}
item_obsexp(PCM(d))
```
#### Rasch-tree DIF sex
```{r}
RIdifTable(d, d_dif$sex)
```
#### Rasch-tree DIF age
```{r}
RIdifTable(d, d_dif$age)
```
#### Rasch-tree DIF relationship status
```{r}
RIdifTable(d, d_dif$rel_status)
```
#### Rasch-tree DIF edu
```{r}
RIdifTable(d, d_dif$edu)
```
#### Partial gamma DIF immigration status
```{r}
# using partial gamma from library(iarm)
RIpartgamDIF(d, d_dif$born_swe)
```
#### Person fit
```{r}
RIpfit(d)
```
#### Floor and ceiling effects
```{r}
RItif(d, samplePSI = T, cutoff = 1)
```
:::

This version of the avoidance subscale only shows a slight underfitting on item q12 and no residual correlations above the relative cut-off. 

Response categories all are working well.

Statistically significant DIF was found for age and relationship status, and the DIF for age was above the cut-off of 0.5.

Let's inspect the category thresholds for DIF.

::: panel-tabset
#### LR DIF Table
```{r}
RIdifThreshTblLR(d, d_dif$age_cat)
```
#### LR DIF Figure
```{r}
RIdifThreshFigLR(d, d_dif$age_cat)
```
#### LR Test
```{r}
LRtest(PCM(d), d_dif$age_cat)
```
:::

Looks like older participants (>74 years) barely discriminate between the two highest categories on item q2, and the two lower categories on item q7. Item q12 seems to, on average, have lower thresholds for older participants.

### Recoding of response categories
```{r}
d %>% 
  mutate(q7 = car::recode(q7,"1=0;2=1;3=2;4=2")) %>% 
  RItileplot()
```
```{r}
d2 <- d %>% 
  mutate(q7 = car::recode(q7,"1=0;2=1;3=2;4=2"))
```

### Re-evaluation of DIF
::: panel-tabset
#### Rasch-tree DIF age
```{r}
RIdifTable(d2, d_dif$age)
```
#### LR DIF Table
```{r}
RIdifThreshTblLR(d2, d_dif$age_cat)
```
#### LR DIF Figure
```{r}
RIdifThreshFigLR(d2, d_dif$age_cat)
```
#### LR Test
```{r}
LRtest(PCM(d2), d_dif$age_cat)
```
:::

Recoding of response categories did not solve the problem of DIF.

### Test Information (Reliability)
```{r}
RItif(d, samplePSI = T)
```

Test information is not above 3.33 on any part of the scale. Reliability is low.

## Dragioti attention version
```{r}
items_dat <- iteminfo %>% 
  filter(dragioti == "yes", factor == "attention") %>% 
  pull(itemnr)

d <- d_all %>% 
  select(all_of(items_dat))
```


### Rasch analysis

The eRm package, which uses Conditional Maximum Likelihood (CML)
estimation, will be used primarily. For this analysis, the Partial
Credit Model will be used.

```{r}
#| column: margin
#| echo: false
RIlistItemsMargin(d, fontsize = 13)
```

::: panel-tabset
#### Conditional item fit
```{r}
#RIitemfit(d, cutoff = "Smith98")

simfit1 <- RIgetfit(d, iterations = 200, cpu = 8) 

RIitemfit(d, simfit1)
RIgetfitPlot(simfit1, d)
```
#### Item-restscore
```{r}
RIrestscore(d)
```
#### PCA
```{r}
#| tbl-cap: "PCA of Rasch model residuals"
RIpcmPCA(d)
```
#### Local dependency
```{r}
# using partial gamma LD from library(iarm)
RIpartgamLD(d)
```

#### Residual correlations
```{r}
simcor1 <- RIgetResidCor(d, iterations = 250, cpu = 8)
RIresidcorr(d, cutoff = simcor1$p99)
```
#### 1st contrast loadings
```{r}
RIloadLoc(d)
```
#### Response categories
```{r}
mirt(d, model=1, itemtype='Rasch', verbose = FALSE) %>% 
  plot(type="trace", as.table = TRUE, 
       theta_lim = c(-6,6))
# for fewer items or a more magnified figure, use:
#RIitemCats(d)
```
#### Targeting
```{r}
#| fig-height: 5
# increase fig-height above as needed, if you have many items
RItargeting(d)
```
#### Item hierarchy
```{r}
#| fig-height: 5
RIitemHierarchy(d)
```
#### Score groups LR-test
```{r}
iarm::score_groups(as.data.frame(d)) %>% 
  as.data.frame(nm = "score_group") %>% 
  dplyr::count(score_group)

dif_plots <- d %>% 
  add_column(dif = iarm::score_groups(.)) %>% 
  split(.$dif) %>% # split the data using the DIF variable
  map(~ RItileplot(.x %>% dplyr::select(!dif)) + labs(title = .x$dif))
dif_plots[[1]] + dif_plots[[2]]

clr_tests(d, model = "PCM")
```
#### Score groups obs-exp
```{r}
item_obsexp(PCM(d))
```
#### Rasch-tree DIF sex
```{r}
RIdifTable(d, d_dif$sex)
```
#### Rasch-tree DIF age
```{r}
RIdifTable(d, d_dif$age)
```
#### Rasch-tree DIF relationship status
```{r}
RIdifTable(d, d_dif$rel_status)
```
#### Rasch-tree DIF edu
```{r}
RIdifTable(d, d_dif$edu)
```
#### Partial gamma DIF immigration status
```{r}
# using partial gamma from library(iarm)
RIpartgamDIF(d, d_dif$born_swe)
```
#### Person fit
```{r}
RIpfit(d)
```
#### Floor and ceiling effects
```{r}
RItif(d, samplePSI = T, cutoff = 1)
```
:::

It is quite clearly problematic to include both items q3 and q4 in a scale with other items. The item mistfit and residual correlations indicate this.

The items themselves are also problematic with poor ordering of category thresholds.

If any of these items should be included in the attention subscale, only one of them is enough.

### Test Information (Reliability)
```{r}
RItif(d, samplePSI = T)
```

Test information 3.33 (PSI = 0.7) is reached between 0.15 and 1.2 logits, where 5.6% of the participants are located.
```
