# Supplementary figures and images for: Psychometric properties of the Swedish cardiac anxiety questionnaire: a Rasch analysis (part 2 of 2)
Source: Sci Rep. 2025 Nov 24;15:41834. doi: 10.1038/s41598-025-28073-8 (PMC12647126; doi:10.1038/s41598-025-28073-8)

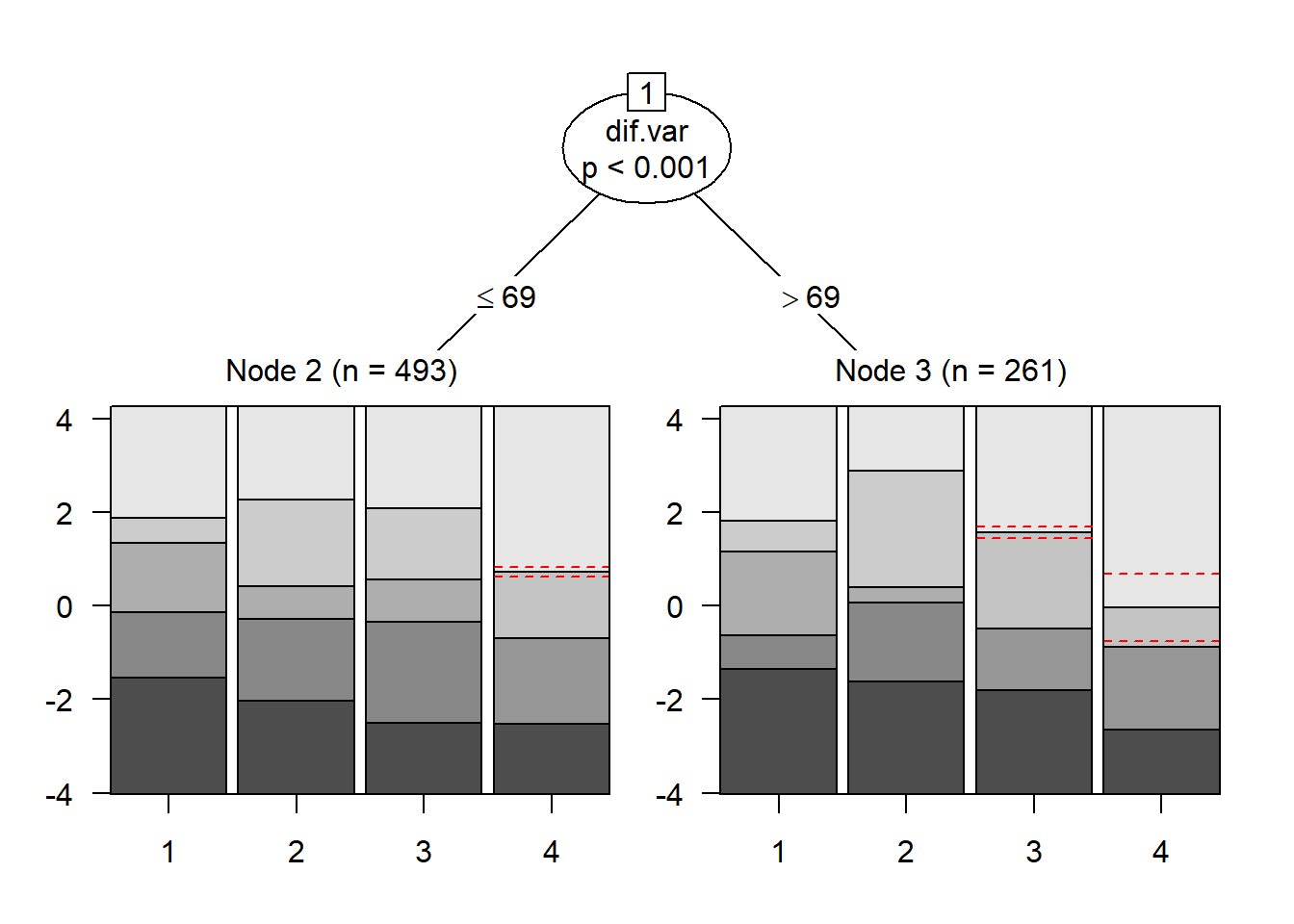

Supplement: Supplementary file 1 — Supplementary Material 1 [file 41598_2025_28073_MOESM1_ESM.zip › Supplementary/analysis_drag_files/figure-html/unnamed-chunk-27-1.png]

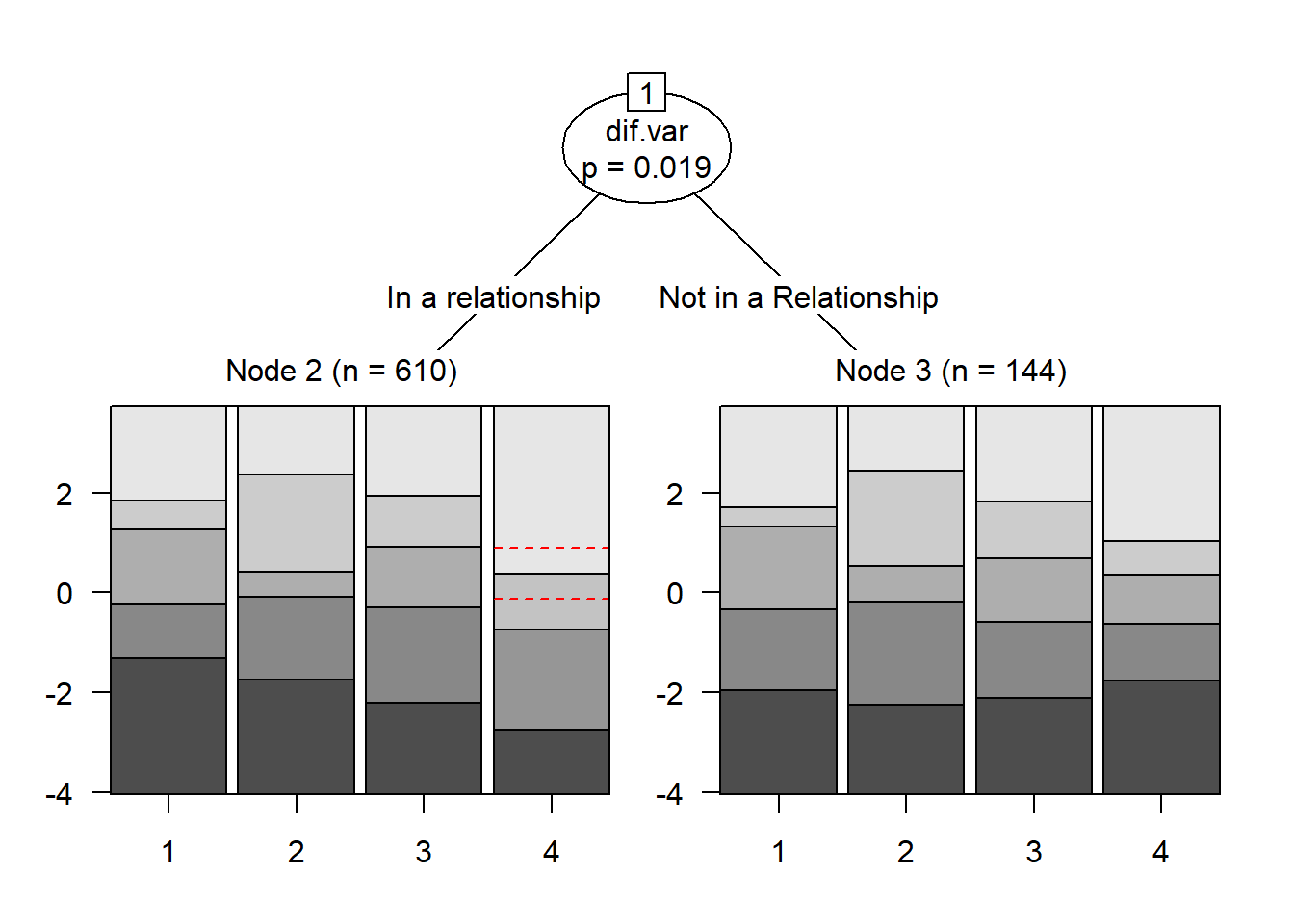

Supplement: Supplementary file 1 — Supplementary Material 1 [file 41598_2025_28073_MOESM1_ESM.zip › Supplementary/analysis_drag_files/figure-html/unnamed-chunk-28-1.png]

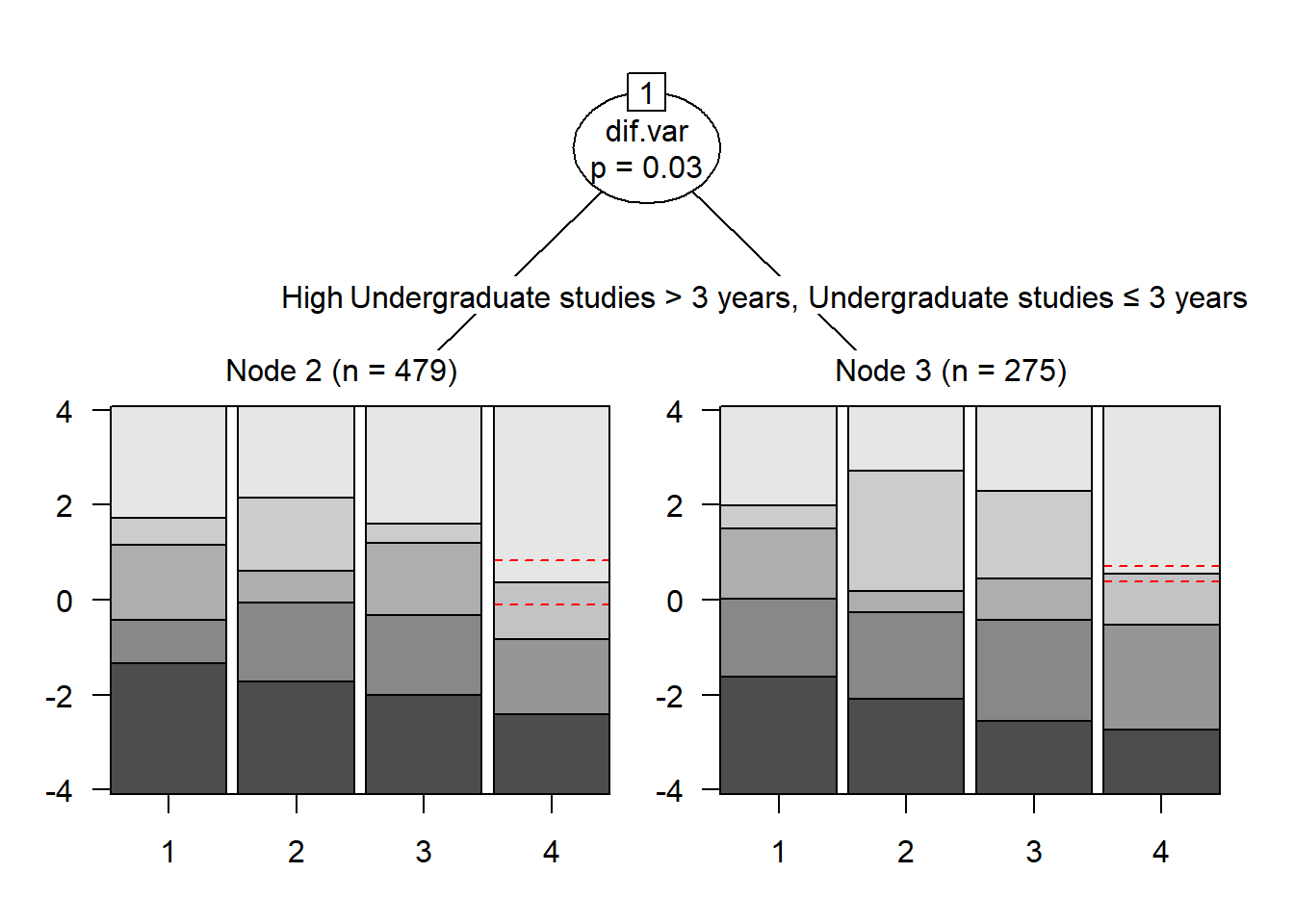

Supplement: Supplementary file 1 — Supplementary Material 1 [file 41598_2025_28073_MOESM1_ESM.zip › Supplementary/analysis_drag_files/figure-html/unnamed-chunk-29-1.png]

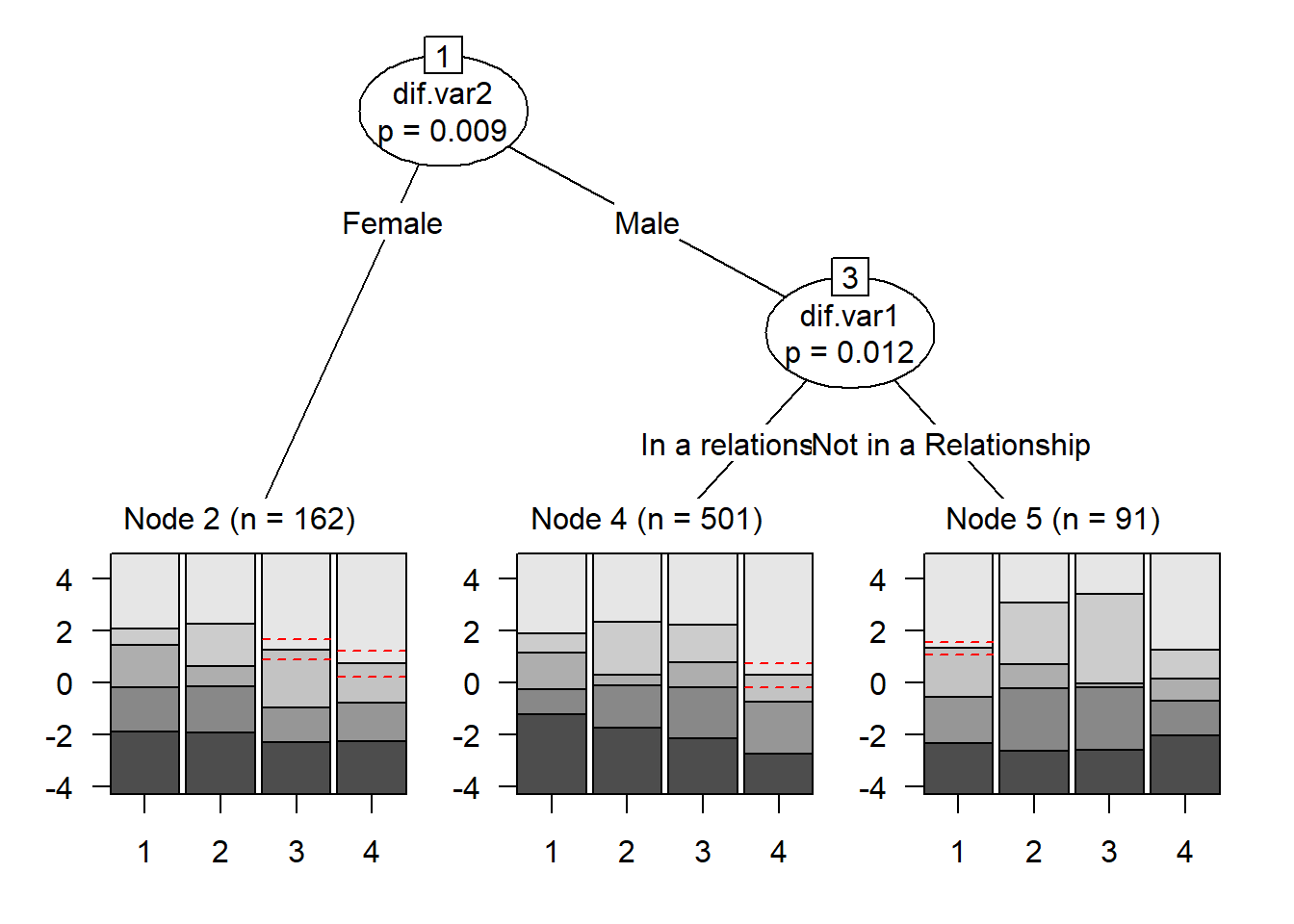

Supplement: Supplementary file 1 — Supplementary Material 1 [file 41598_2025_28073_MOESM1_ESM.zip › Supplementary/analysis_drag_files/figure-html/unnamed-chunk-31-1.png]

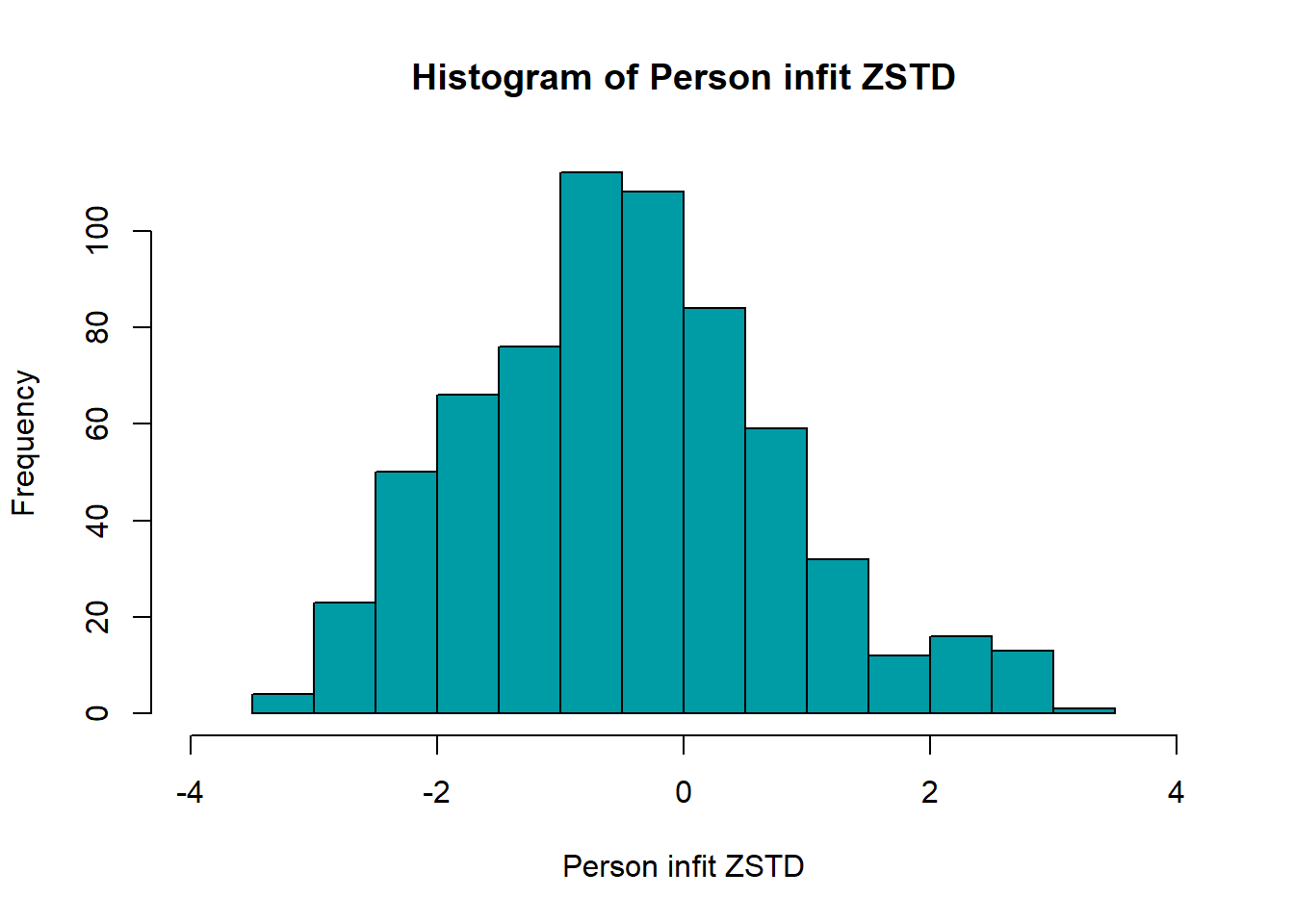

Supplement: Supplementary file 1 — Supplementary Material 1 [file 41598_2025_28073_MOESM1_ESM.zip › Supplementary/analysis_drag_files/figure-html/unnamed-chunk-32-1.png]

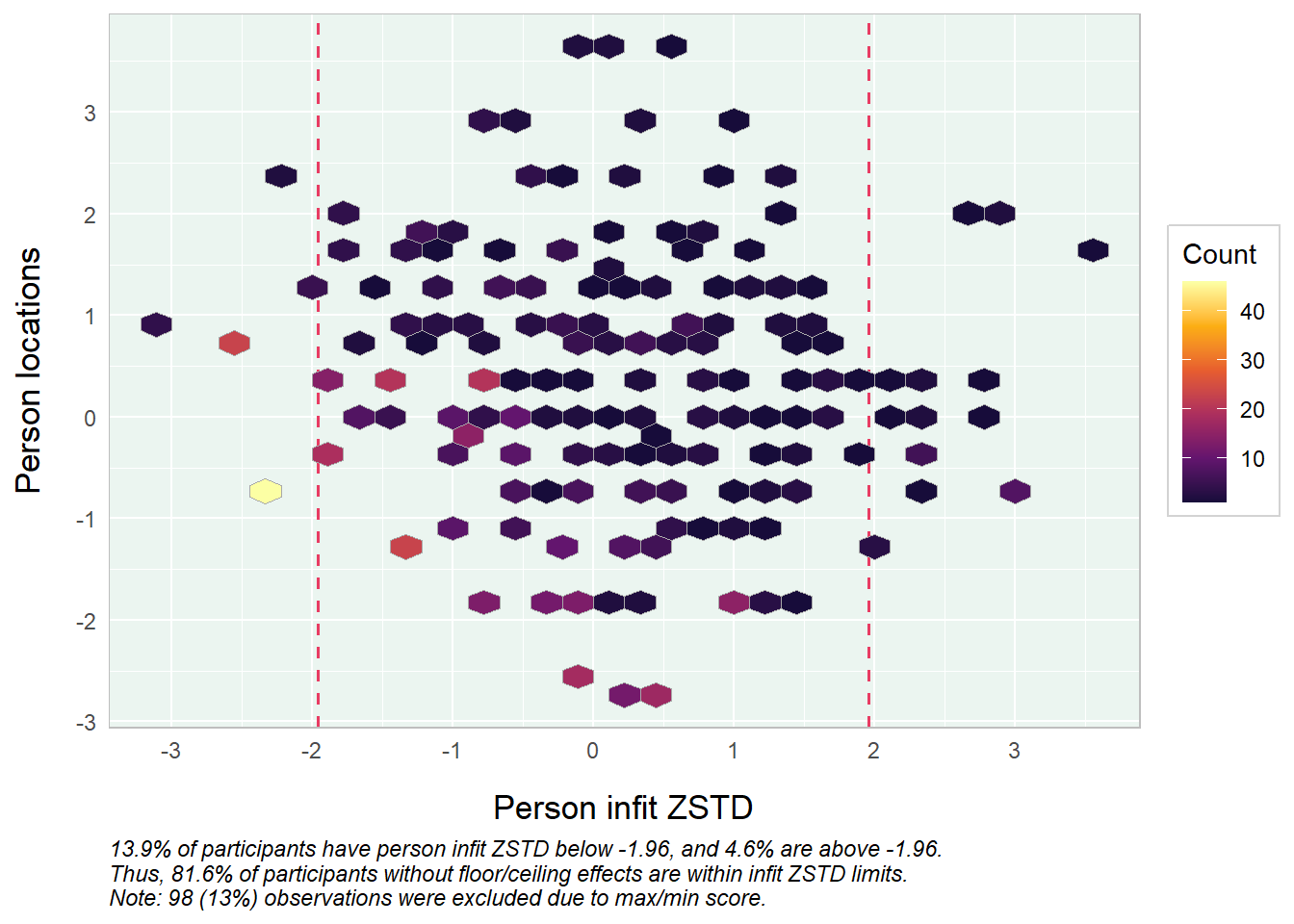

Supplement: Supplementary file 1 — Supplementary Material 1 [file 41598_2025_28073_MOESM1_ESM.zip › Supplementary/analysis_drag_files/figure-html/unnamed-chunk-32-2.png]

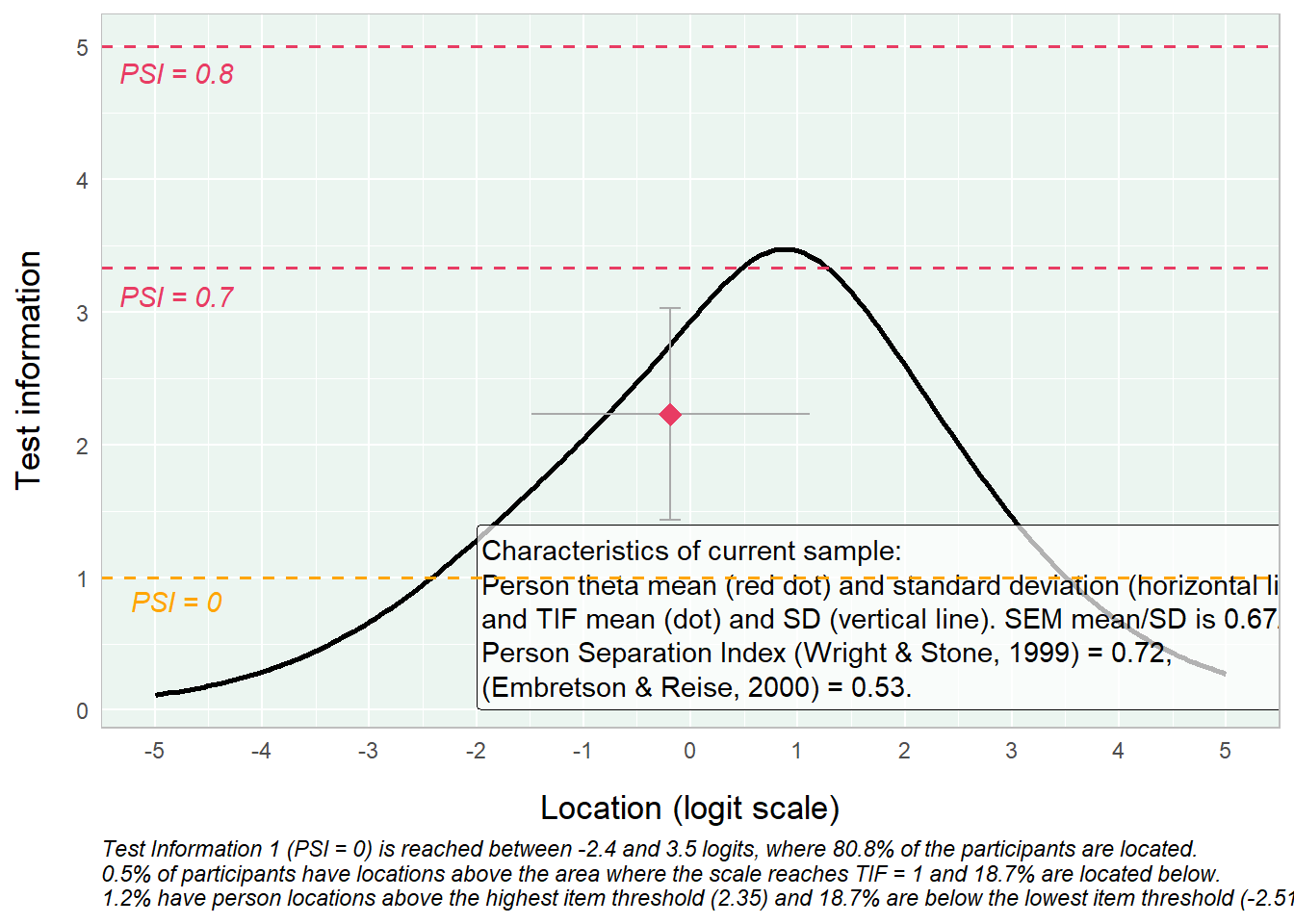

Supplement: Supplementary file 1 — Supplementary Material 1 [file 41598_2025_28073_MOESM1_ESM.zip › Supplementary/analysis_drag_files/figure-html/unnamed-chunk-33-1.png]

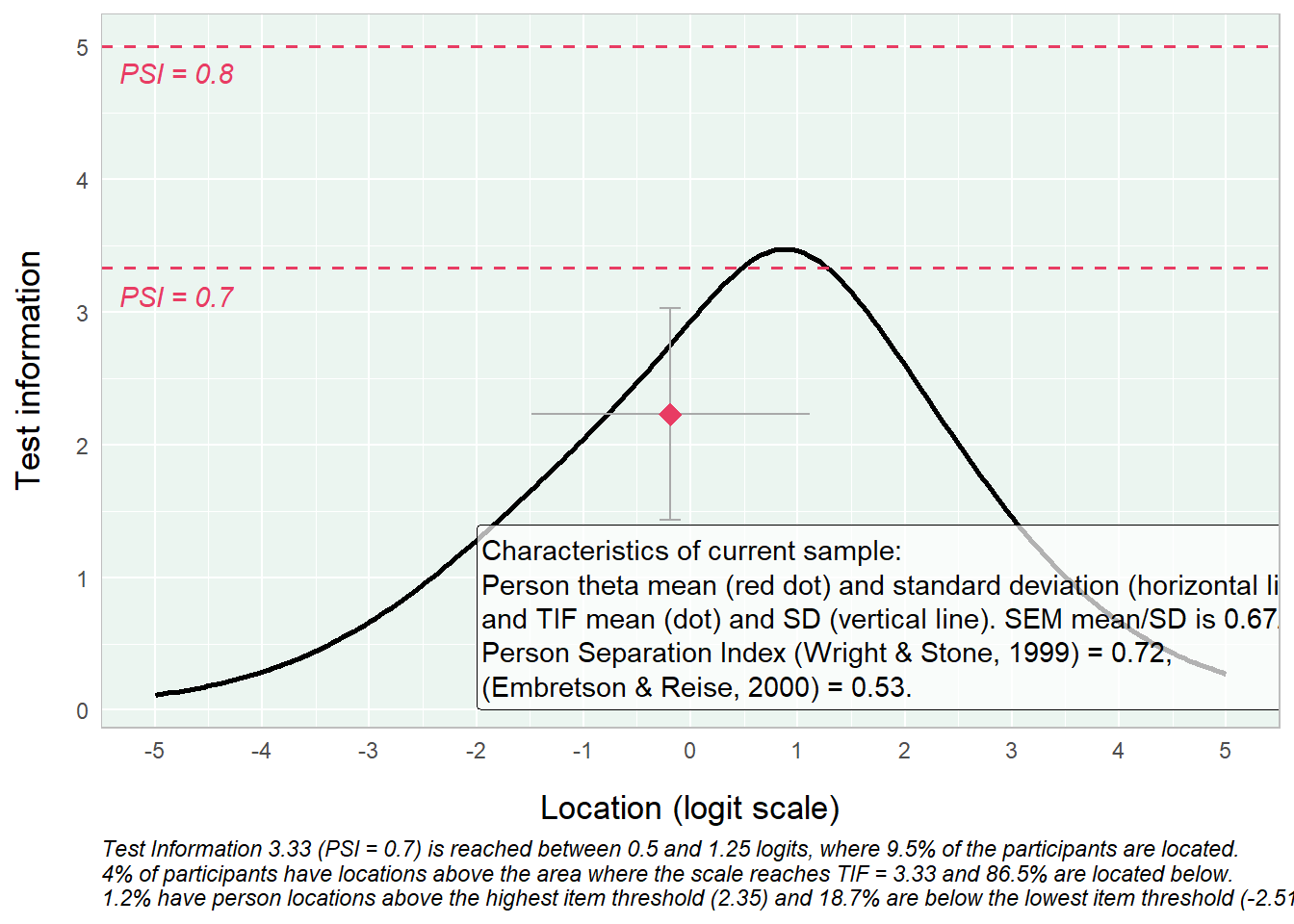

Supplement: Supplementary file 1 — Supplementary Material 1 [file 41598_2025_28073_MOESM1_ESM.zip › Supplementary/analysis_drag_files/figure-html/unnamed-chunk-34-1.png]

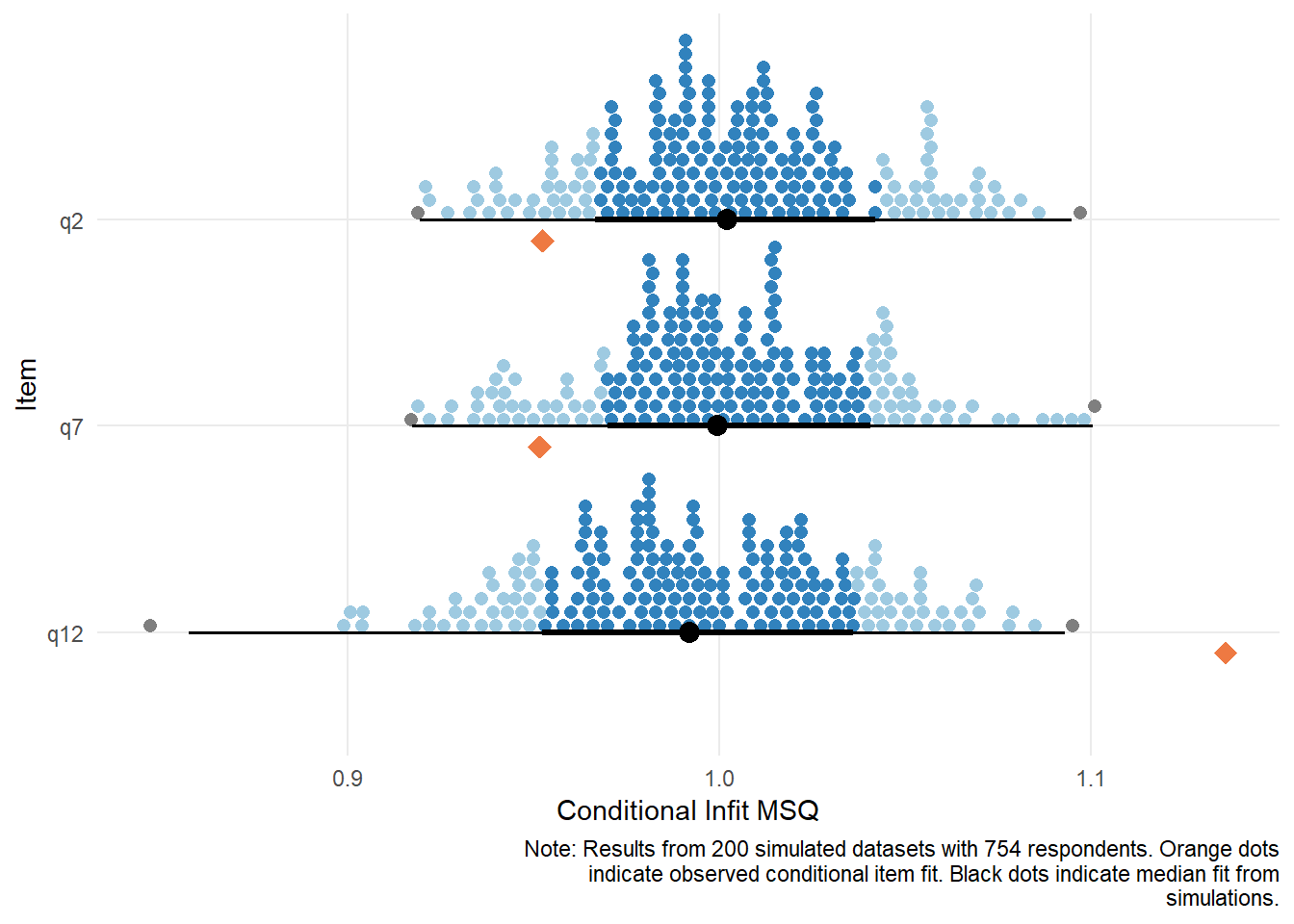

Supplement: Supplementary file 1 — Supplementary Material 1 [file 41598_2025_28073_MOESM1_ESM.zip › Supplementary/analysis_drag_files/figure-html/unnamed-chunk-37-1.png]

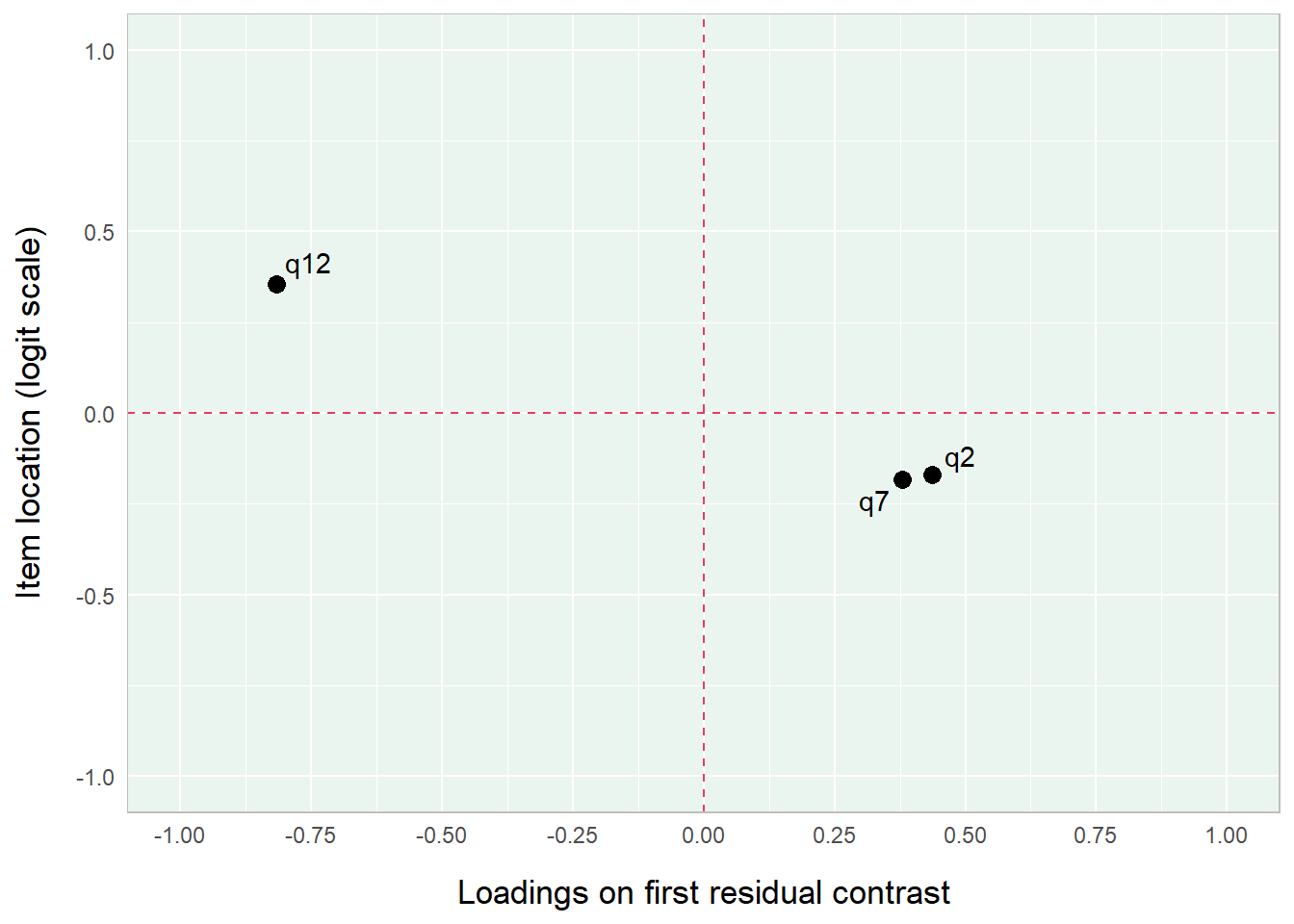

Supplement: Supplementary file 1 — Supplementary Material 1 [file 41598_2025_28073_MOESM1_ESM.zip › Supplementary/analysis_drag_files/figure-html/unnamed-chunk-42-1.png]

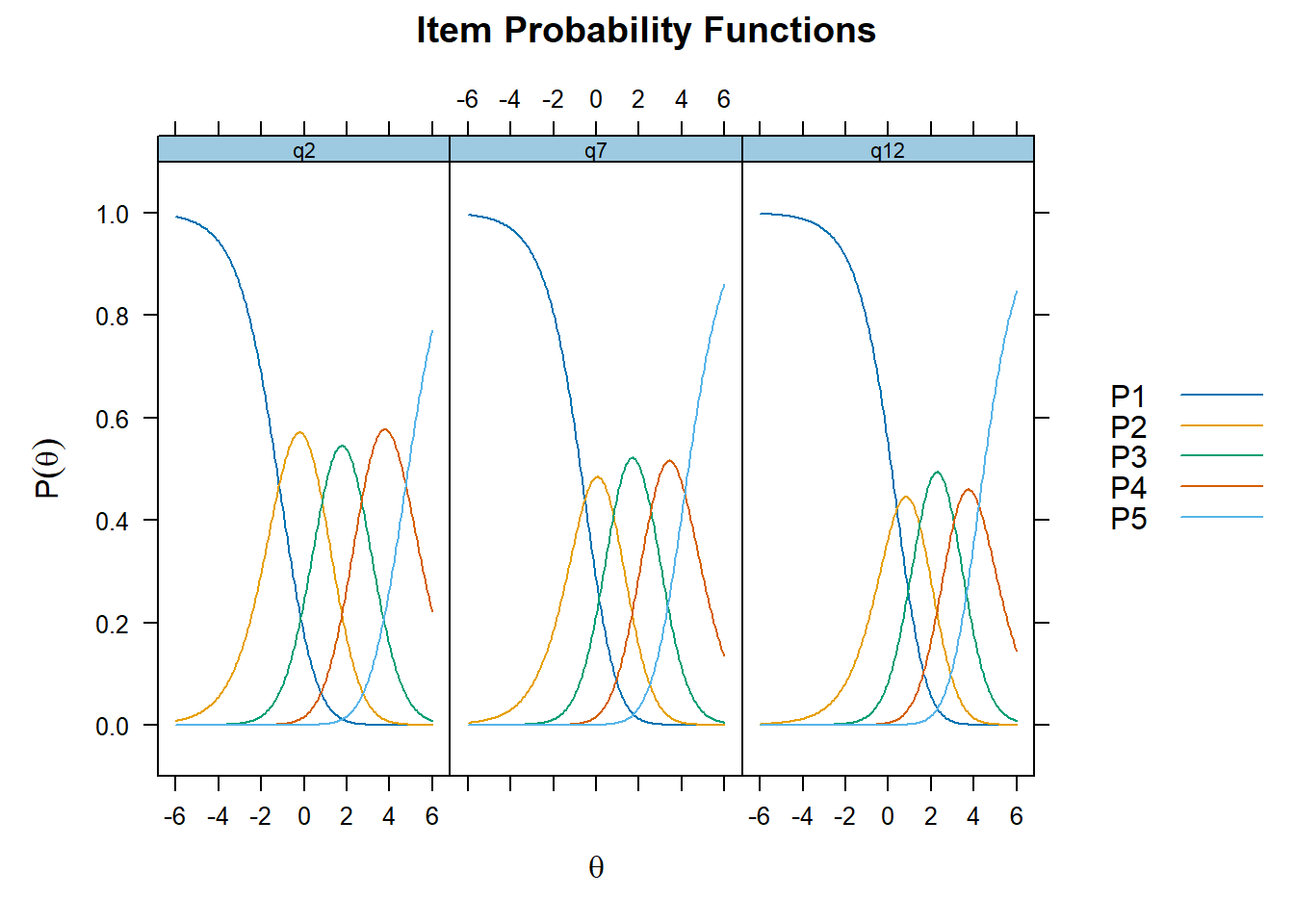

Supplement: Supplementary file 1 — Supplementary Material 1 [file 41598_2025_28073_MOESM1_ESM.zip › Supplementary/analysis_drag_files/figure-html/unnamed-chunk-43-1.png]

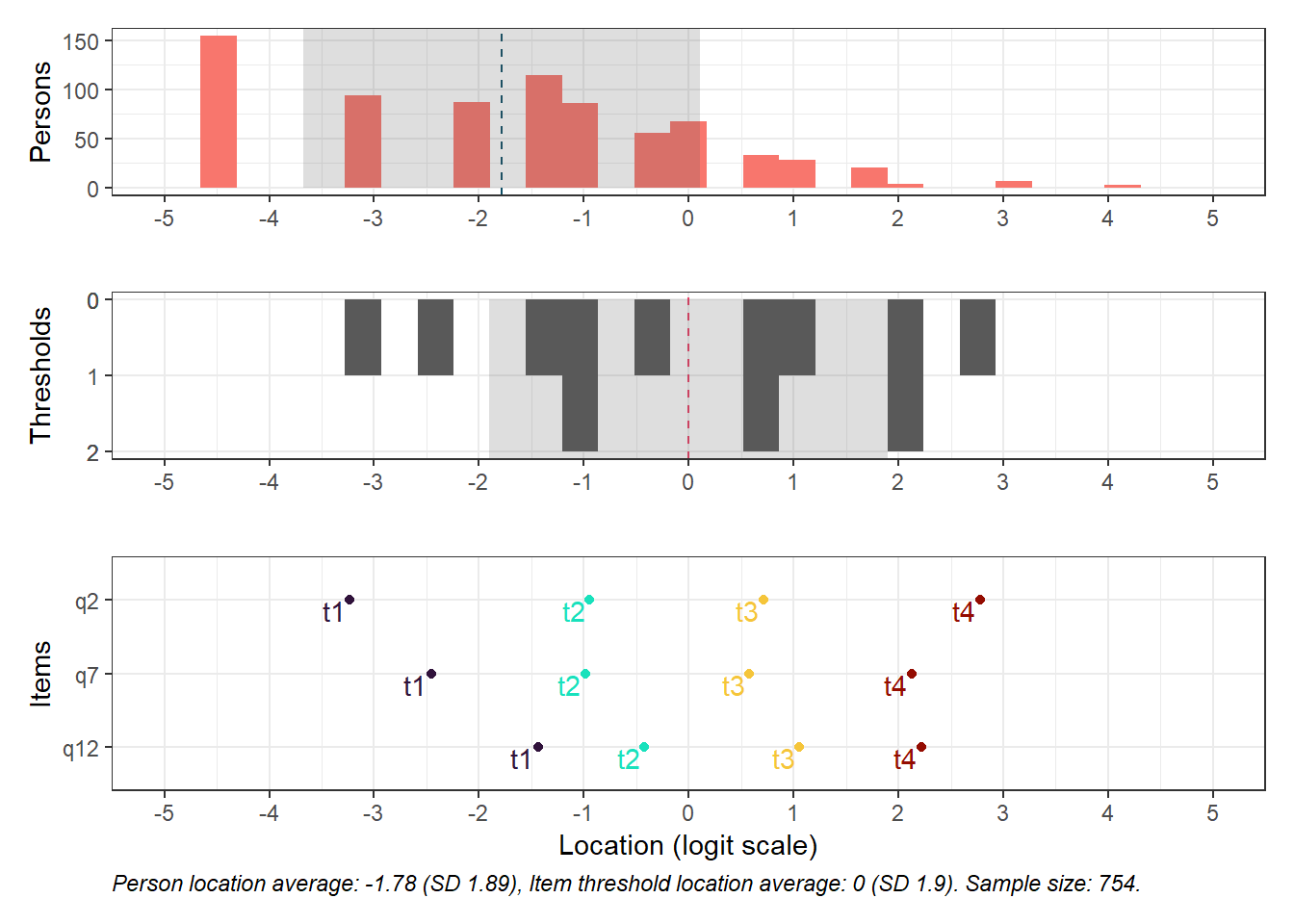

Supplement: Supplementary file 1 — Supplementary Material 1 [file 41598_2025_28073_MOESM1_ESM.zip › Supplementary/analysis_drag_files/figure-html/unnamed-chunk-44-1.png]

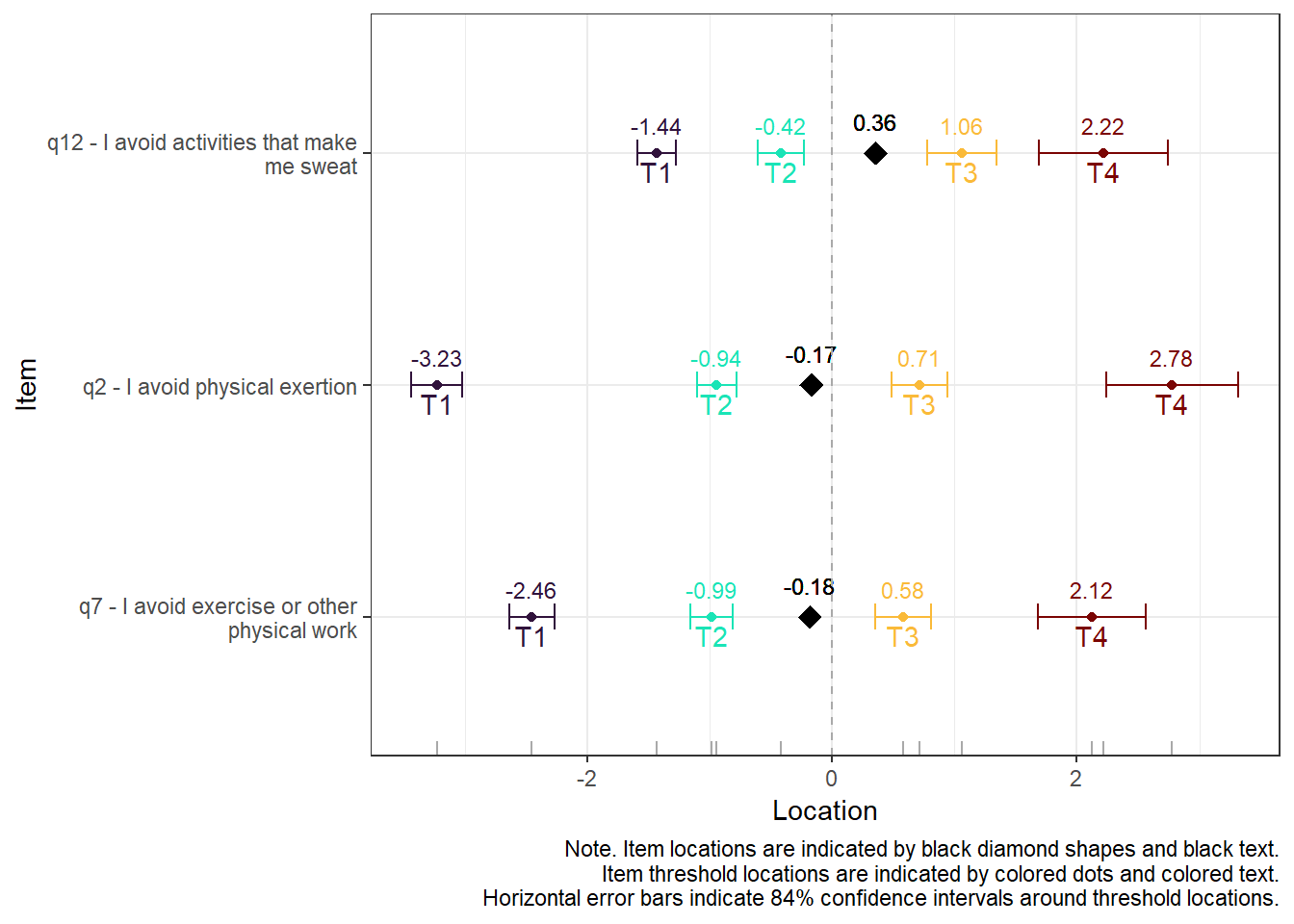

Supplement: Supplementary file 1 — Supplementary Material 1 [file 41598_2025_28073_MOESM1_ESM.zip › Supplementary/analysis_drag_files/figure-html/unnamed-chunk-45-1.png]

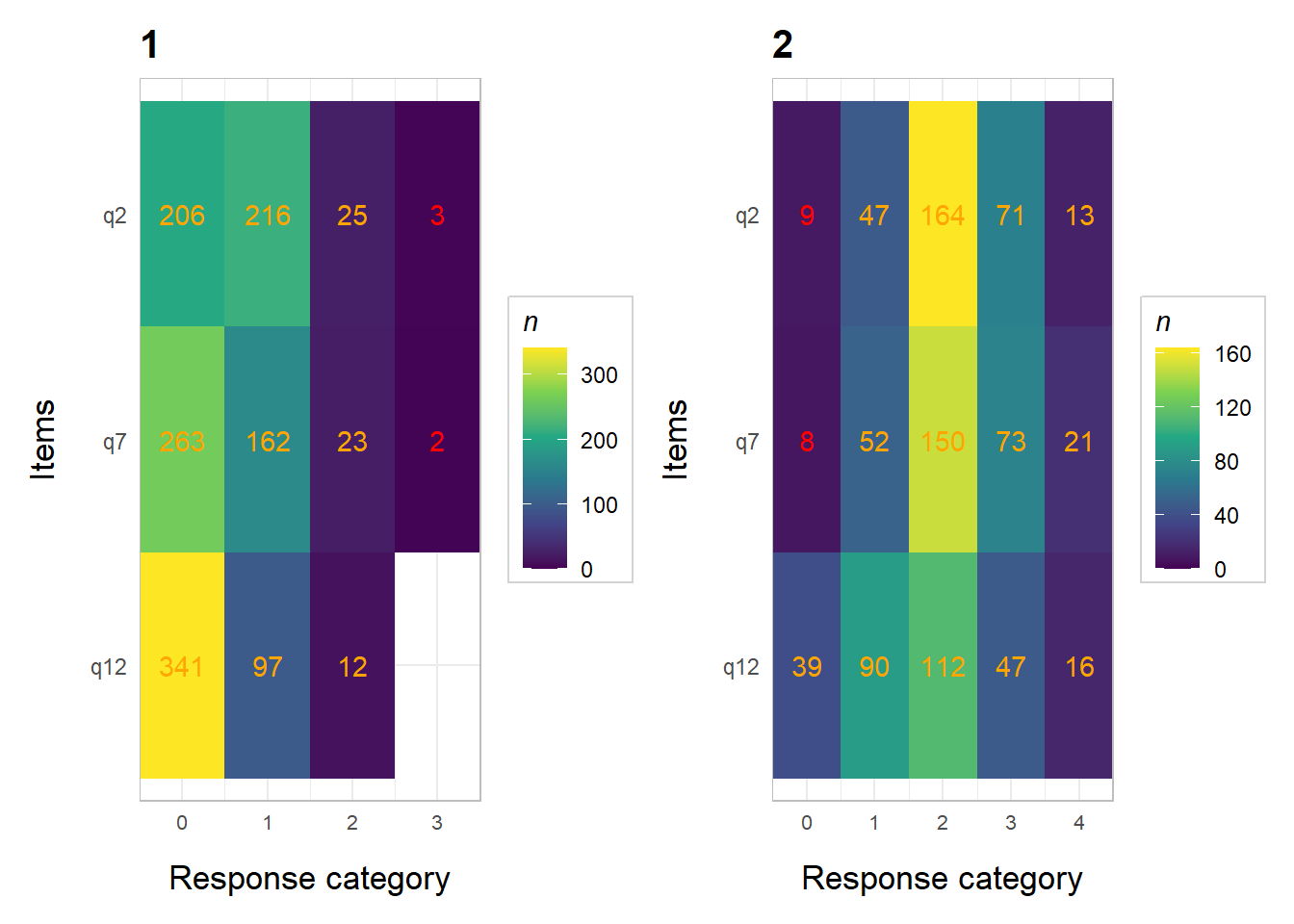

Supplement: Supplementary file 1 — Supplementary Material 1 [file 41598_2025_28073_MOESM1_ESM.zip › Supplementary/analysis_drag_files/figure-html/unnamed-chunk-46-1.png]

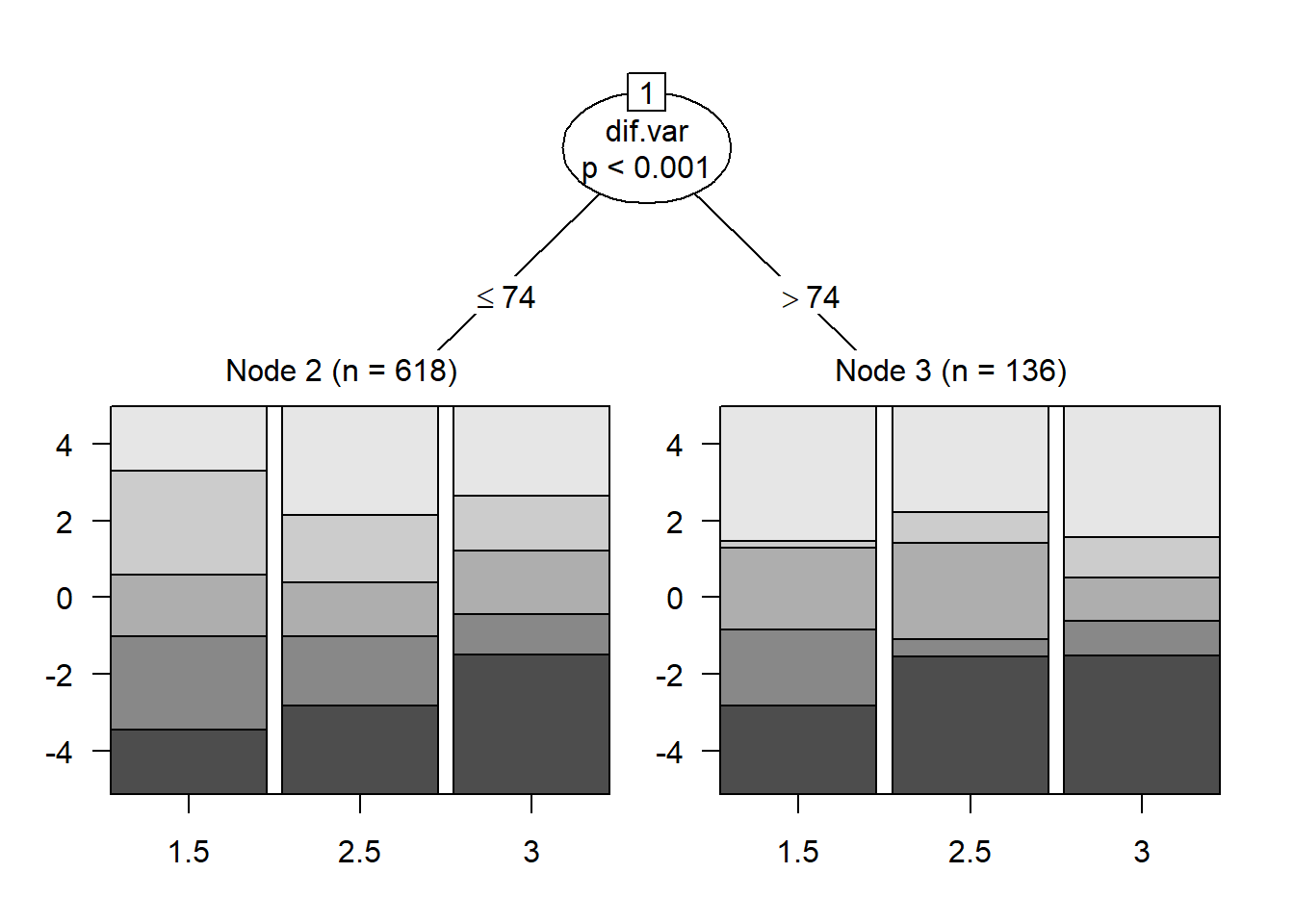

Supplement: Supplementary file 1 — Supplementary Material 1 [file 41598_2025_28073_MOESM1_ESM.zip › Supplementary/analysis_drag_files/figure-html/unnamed-chunk-49-1.png]

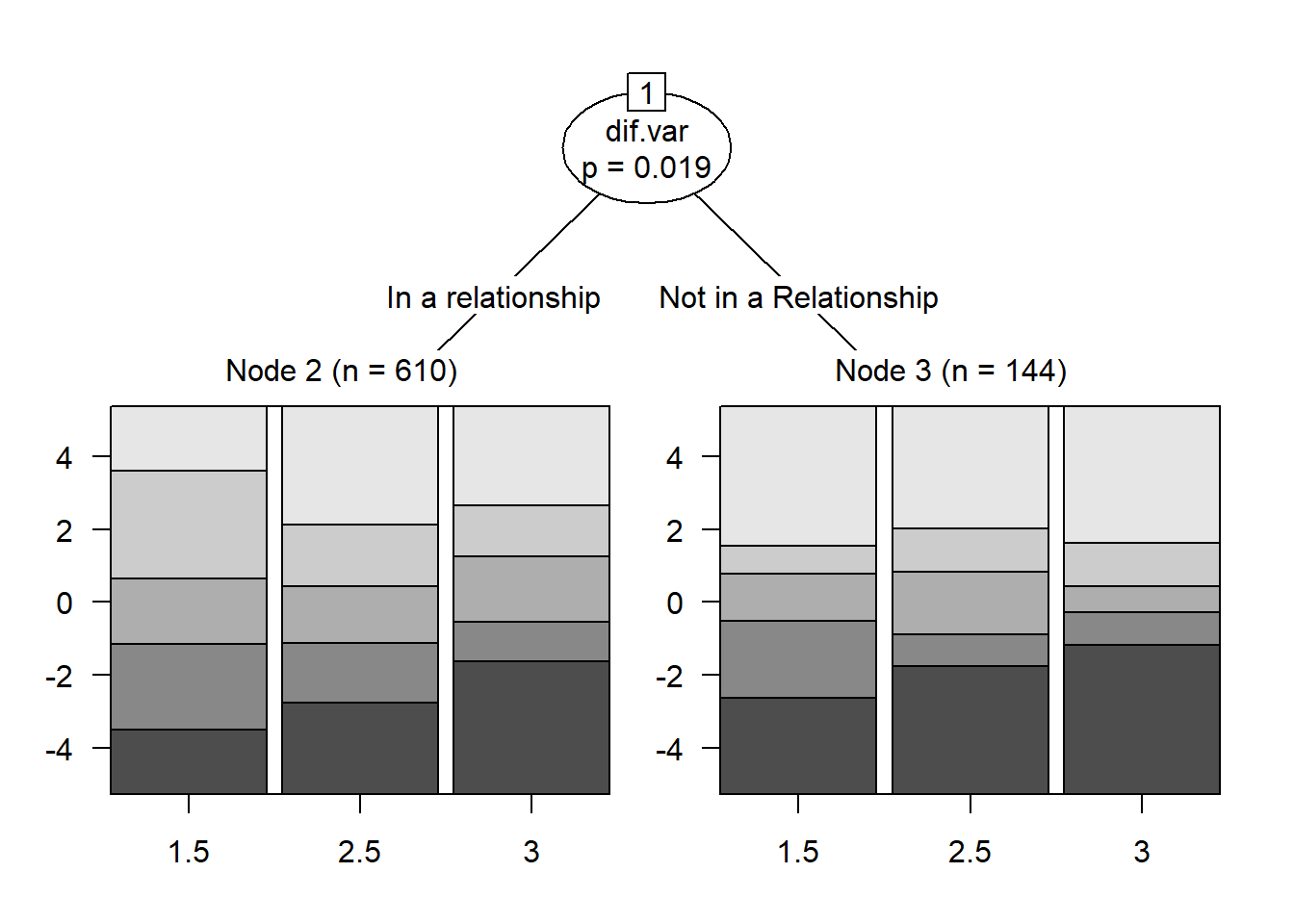

Supplement: Supplementary file 1 — Supplementary Material 1 [file 41598_2025_28073_MOESM1_ESM.zip › Supplementary/analysis_drag_files/figure-html/unnamed-chunk-50-1.png]

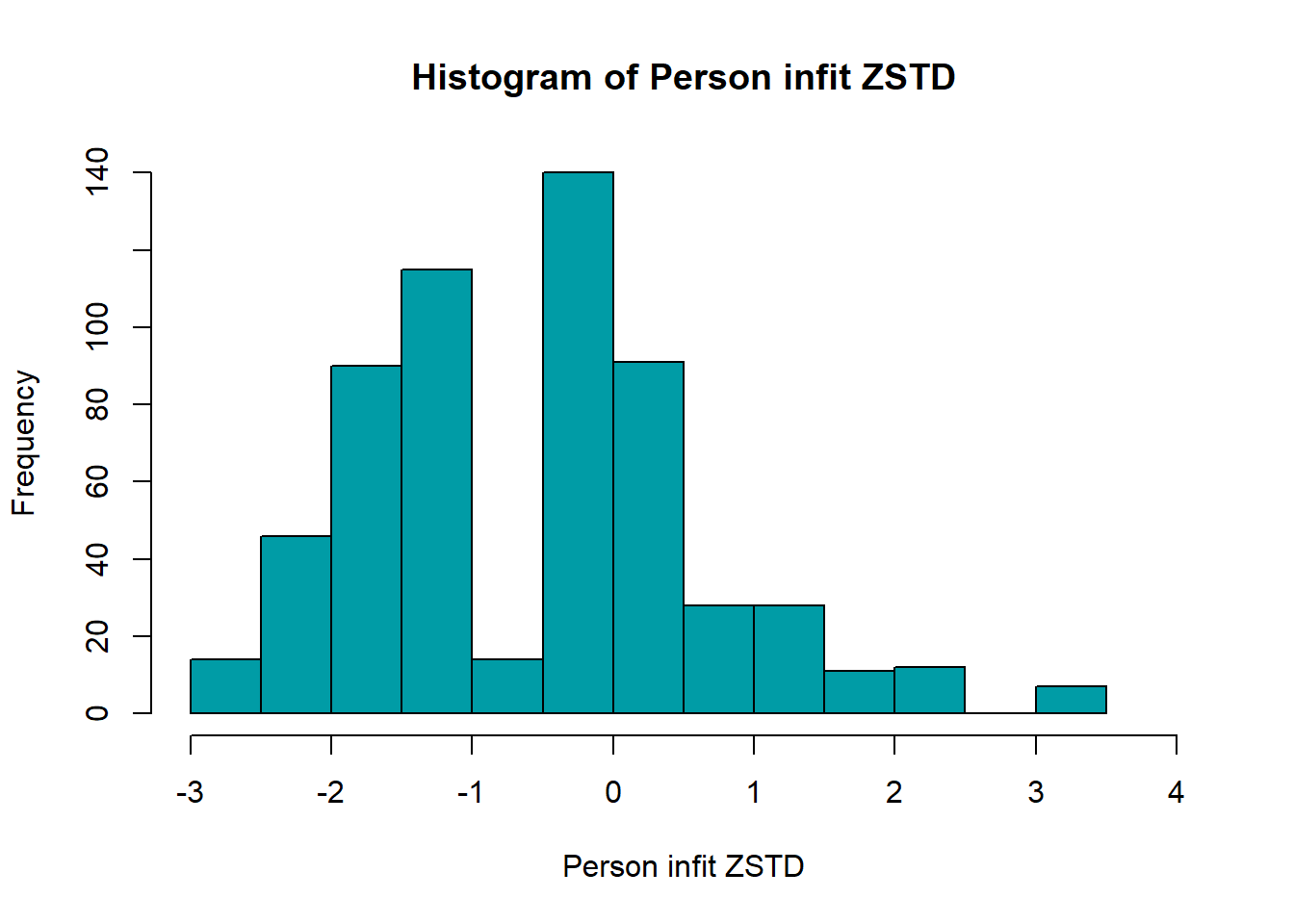

Supplement: Supplementary file 1 — Supplementary Material 1 [file 41598_2025_28073_MOESM1_ESM.zip › Supplementary/analysis_drag_files/figure-html/unnamed-chunk-53-1.png]

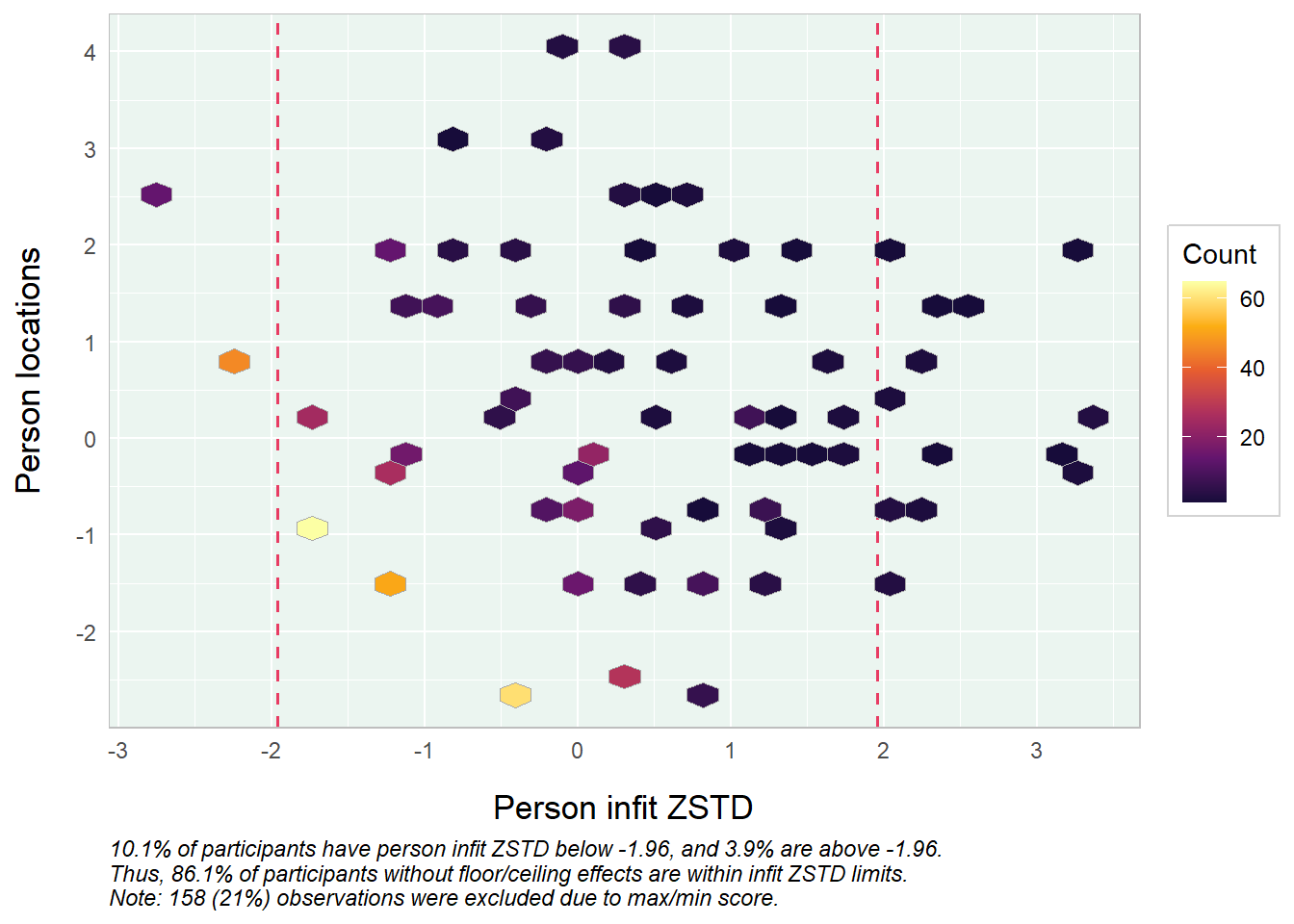

Supplement: Supplementary file 1 — Supplementary Material 1 [file 41598_2025_28073_MOESM1_ESM.zip › Supplementary/analysis_drag_files/figure-html/unnamed-chunk-53-2.png]

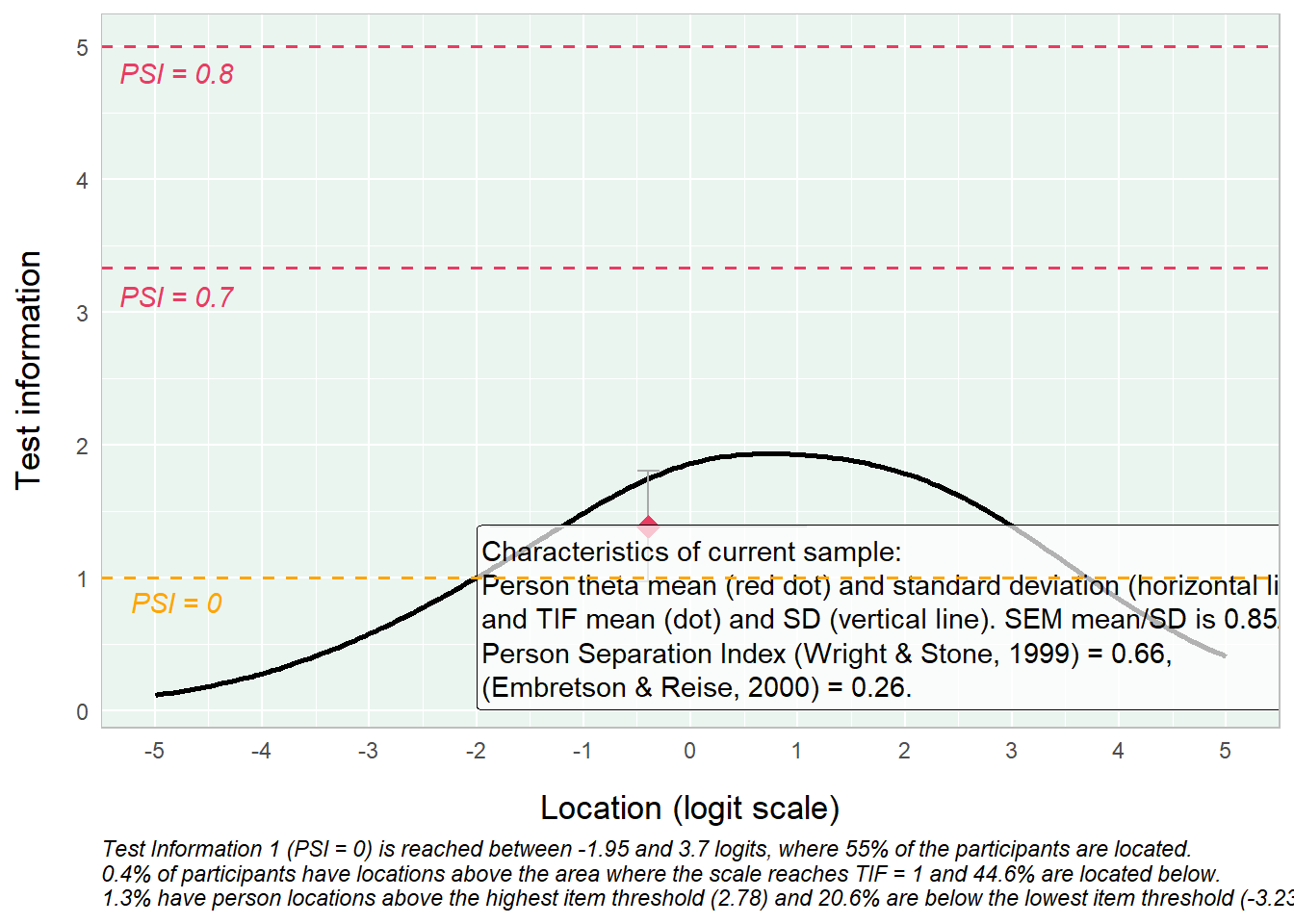

Supplement: Supplementary file 1 — Supplementary Material 1 [file 41598_2025_28073_MOESM1_ESM.zip › Supplementary/analysis_drag_files/figure-html/unnamed-chunk-54-1.png]

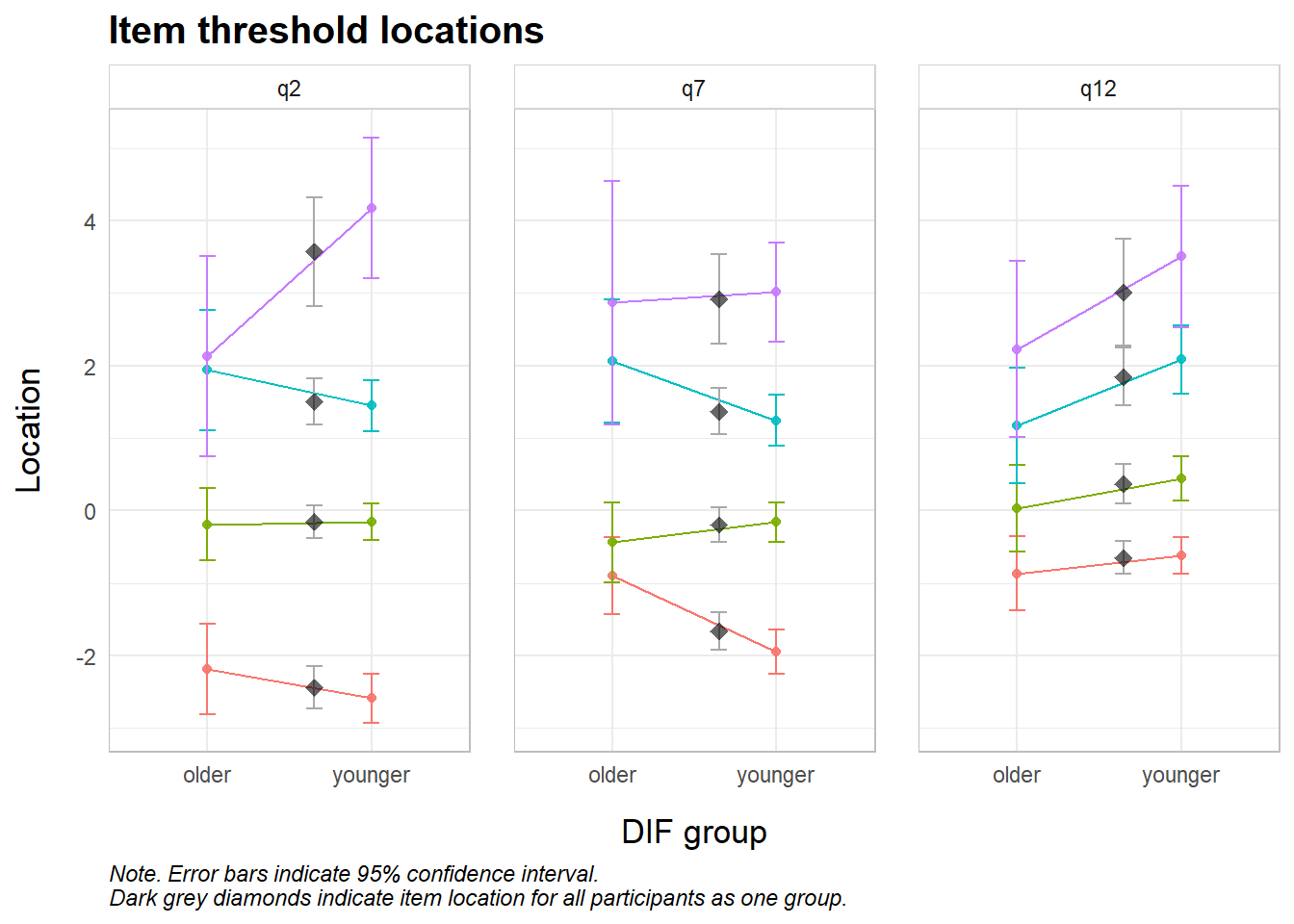

Supplement: Supplementary file 1 — Supplementary Material 1 [file 41598_2025_28073_MOESM1_ESM.zip › Supplementary/analysis_drag_files/figure-html/unnamed-chunk-56-1.png]

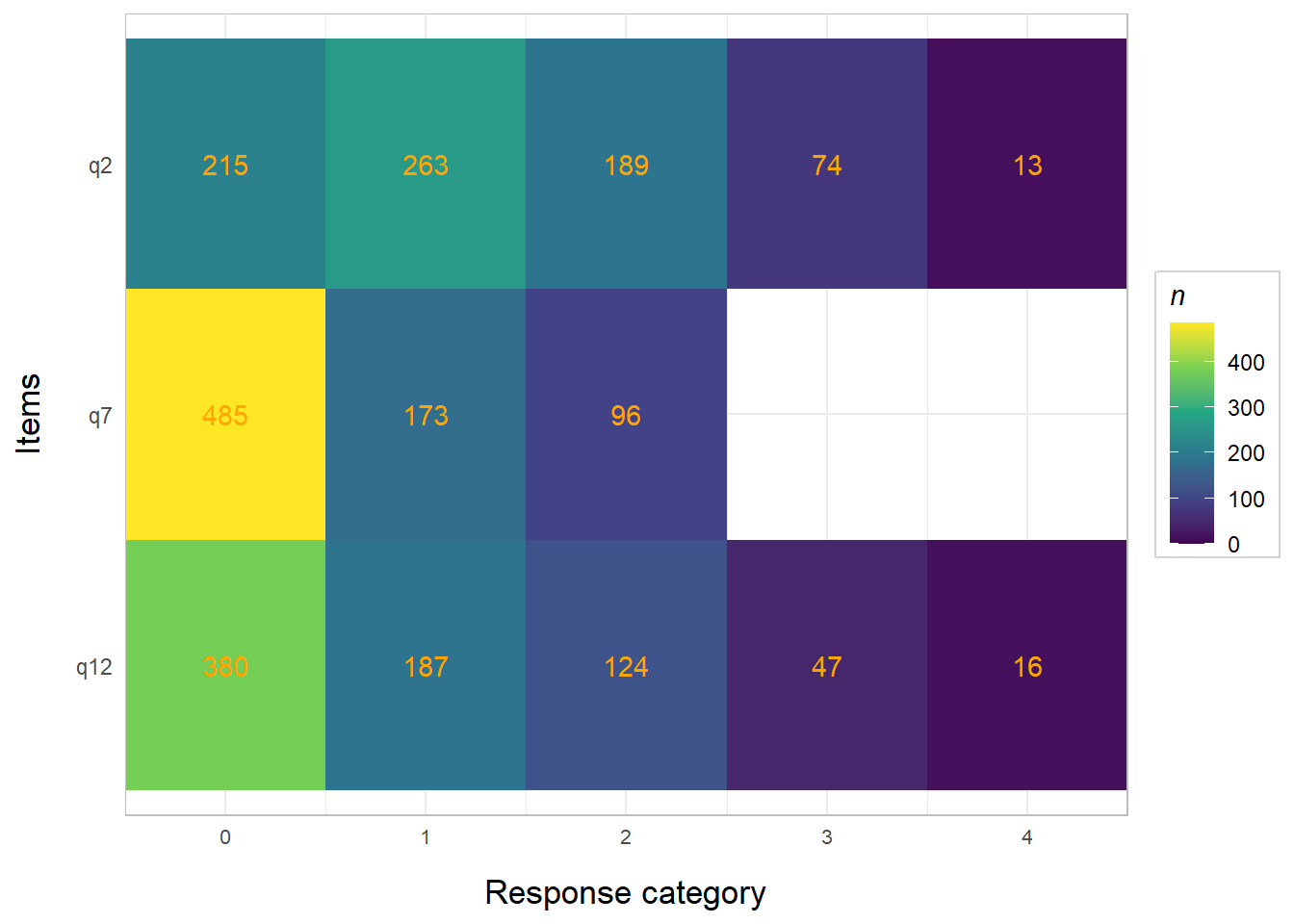

Supplement: Supplementary file 1 — Supplementary Material 1 [file 41598_2025_28073_MOESM1_ESM.zip › Supplementary/analysis_drag_files/figure-html/unnamed-chunk-58-1.png]

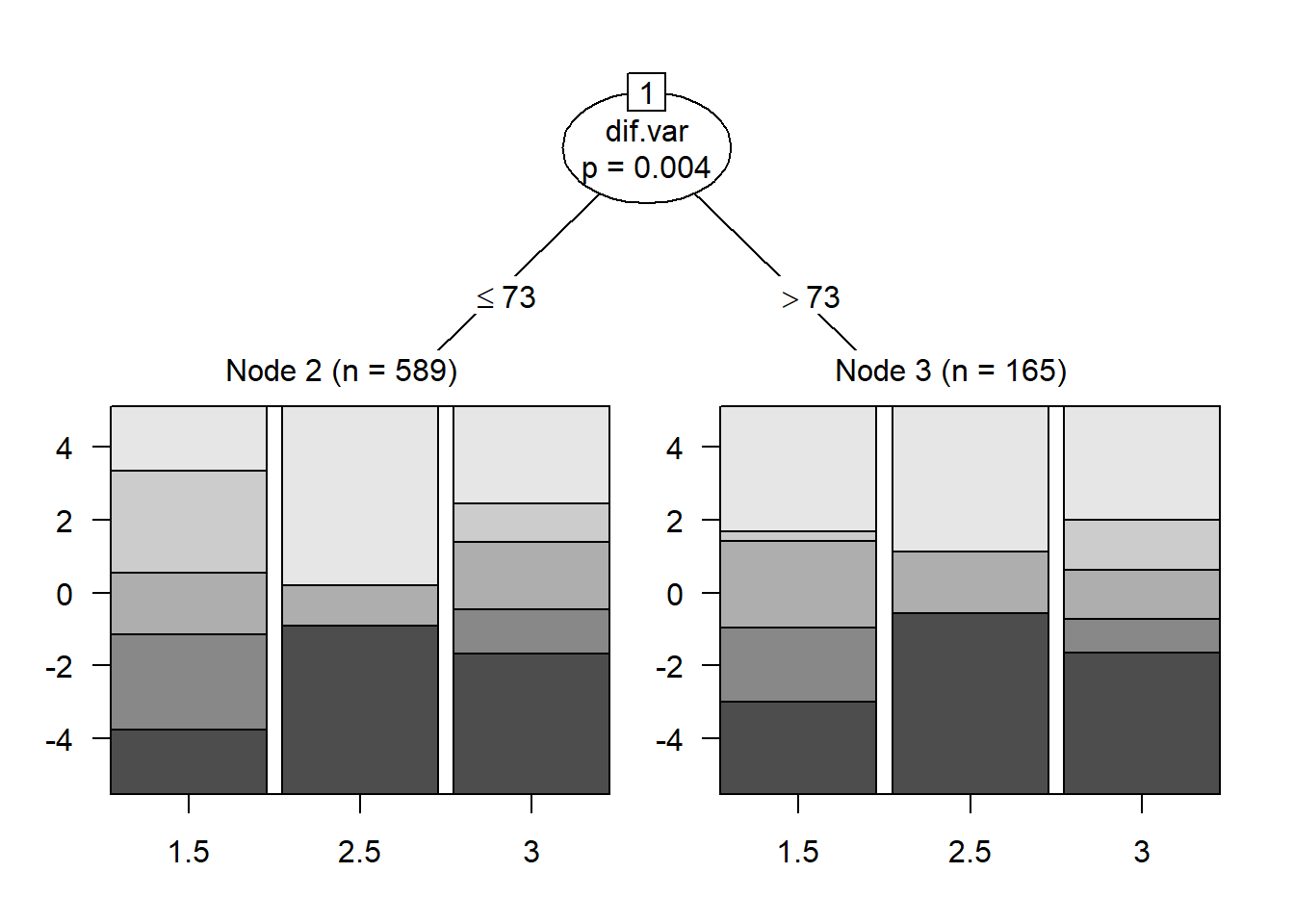

Supplement: Supplementary file 1 — Supplementary Material 1 [file 41598_2025_28073_MOESM1_ESM.zip › Supplementary/analysis_drag_files/figure-html/unnamed-chunk-60-1.png]

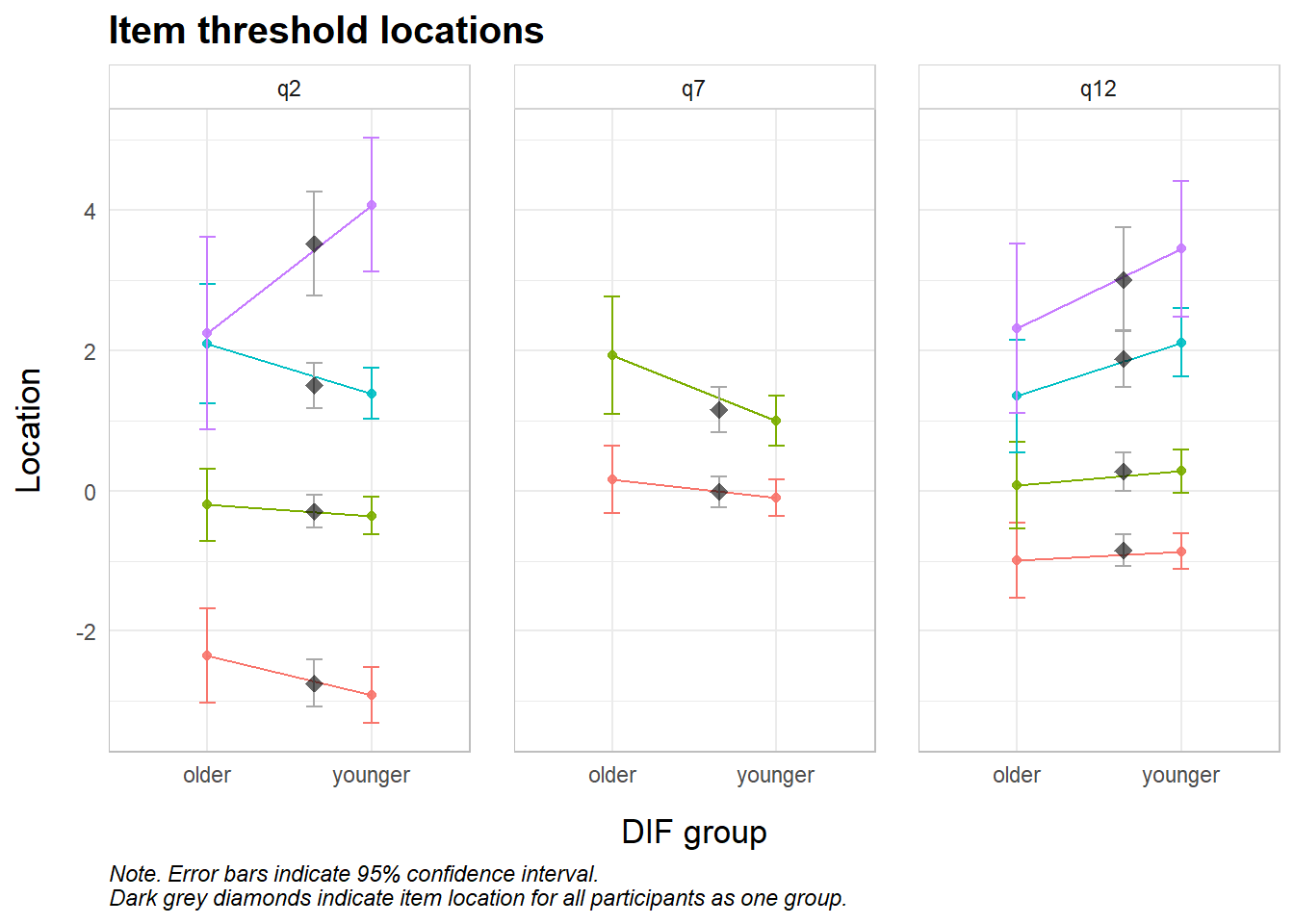

Supplement: Supplementary file 1 — Supplementary Material 1 [file 41598_2025_28073_MOESM1_ESM.zip › Supplementary/analysis_drag_files/figure-html/unnamed-chunk-62-1.png]

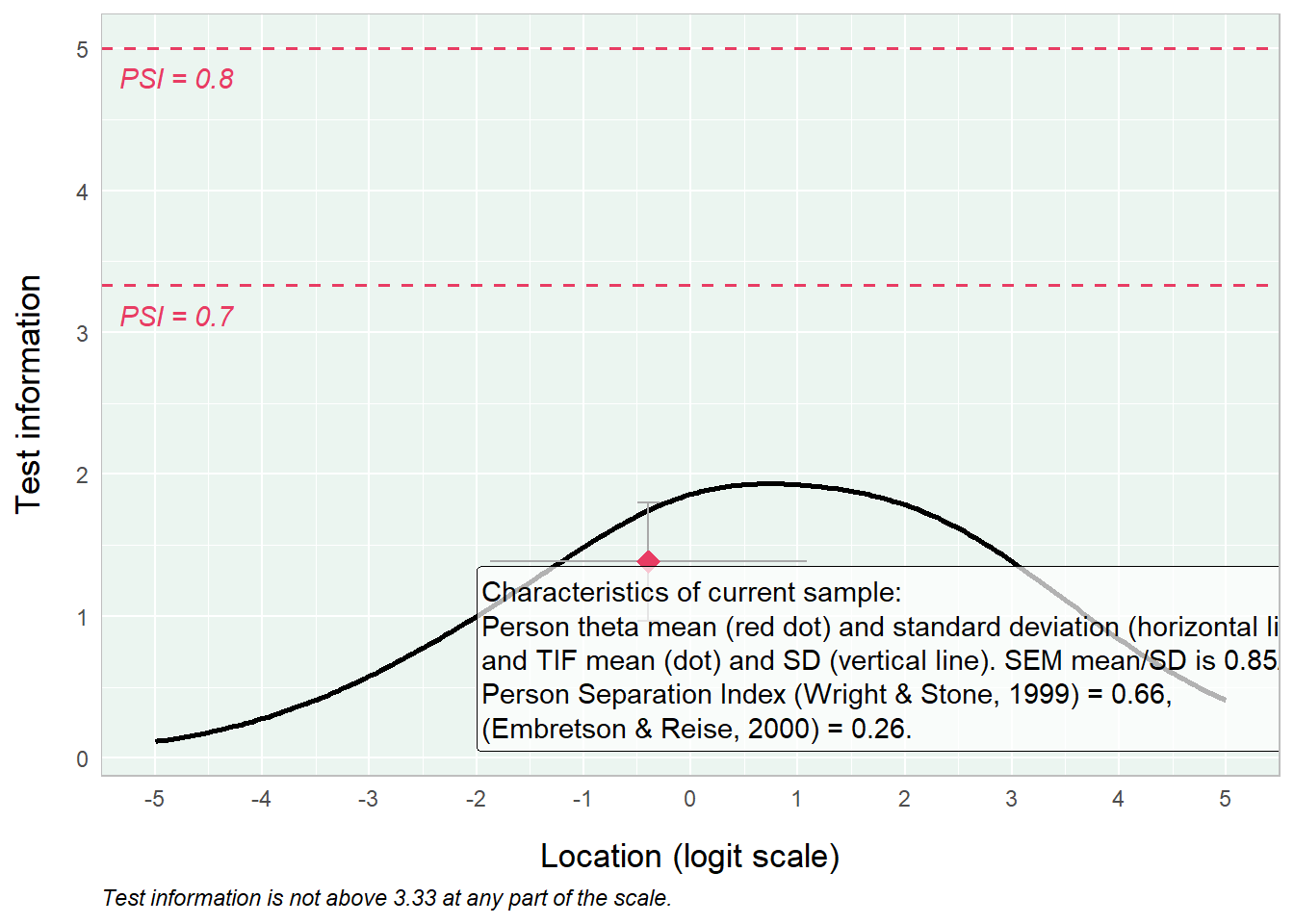

Supplement: Supplementary file 1 — Supplementary Material 1 [file 41598_2025_28073_MOESM1_ESM.zip › Supplementary/analysis_drag_files/figure-html/unnamed-chunk-64-1.png]

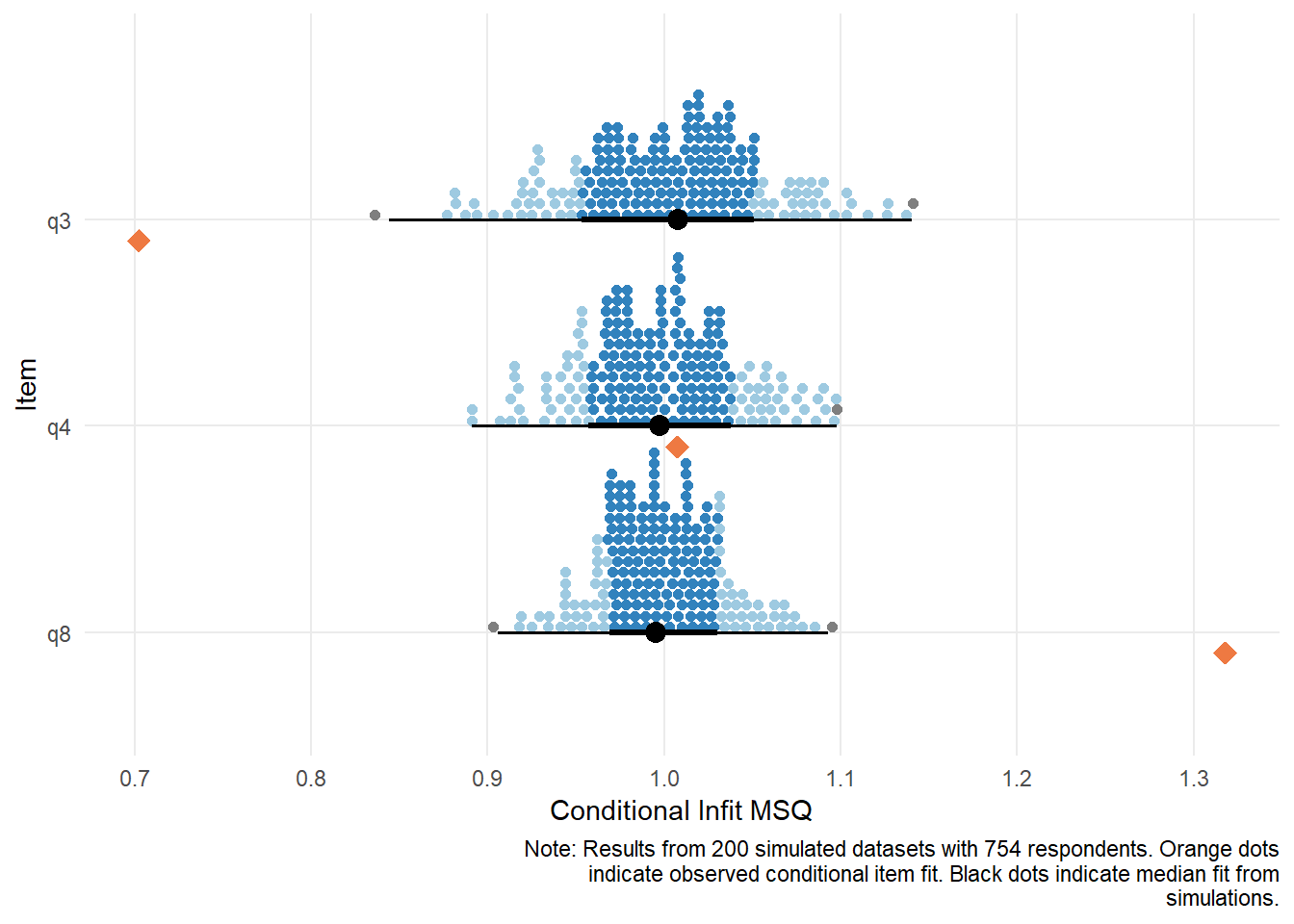

Supplement: Supplementary file 1 — Supplementary Material 1 [file 41598_2025_28073_MOESM1_ESM.zip › Supplementary/analysis_drag_files/figure-html/unnamed-chunk-67-1.png]
