# Supplementary figures and images for: Psychometric properties of the Swedish cardiac anxiety questionnaire: a Rasch analysis (part 1 of 2)
Source: Sci Rep. 2025 Nov 24;15:41834. doi: 10.1038/s41598-025-28073-8 (PMC12647126; doi:10.1038/s41598-025-28073-8)

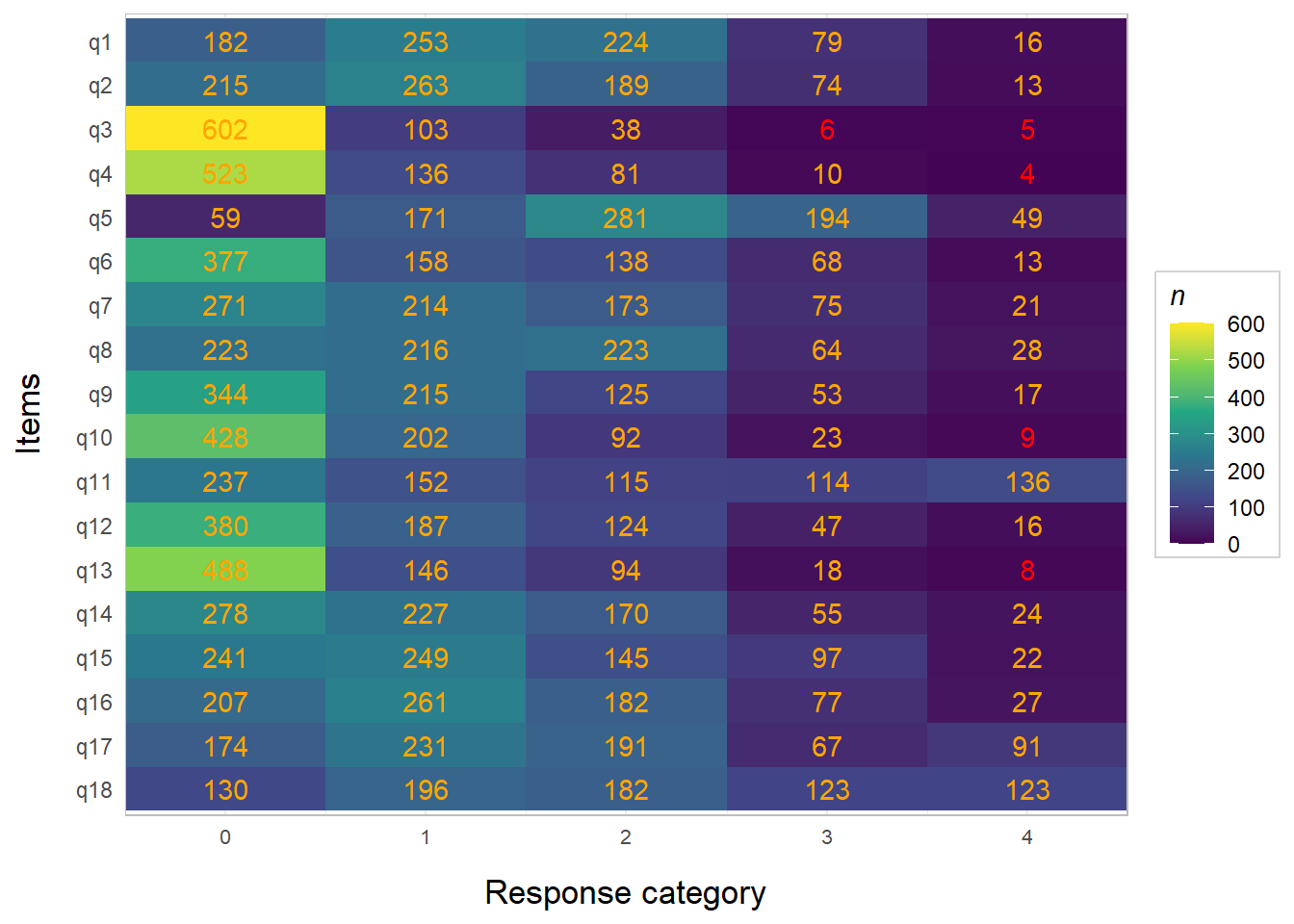

Supplement: Supplementary file 1 — Supplementary Material 1 [file 41598_2025_28073_MOESM1_ESM.zip › Supplementary/analysis_att_files/figure-html/unnamed-chunk-10-1.png]

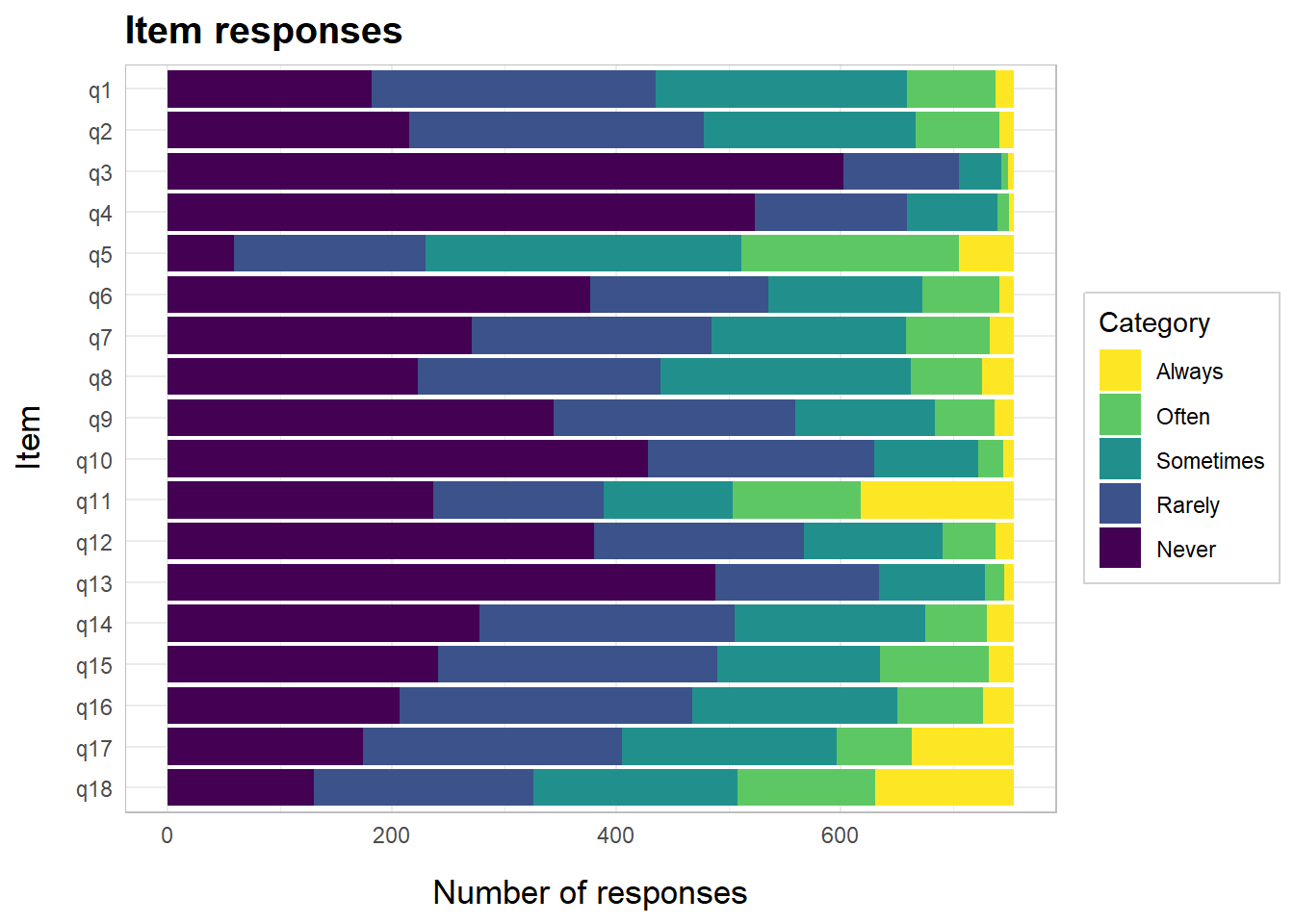

Supplement: Supplementary file 1 — Supplementary Material 1 [file 41598_2025_28073_MOESM1_ESM.zip › Supplementary/analysis_att_files/figure-html/unnamed-chunk-11-1.png]

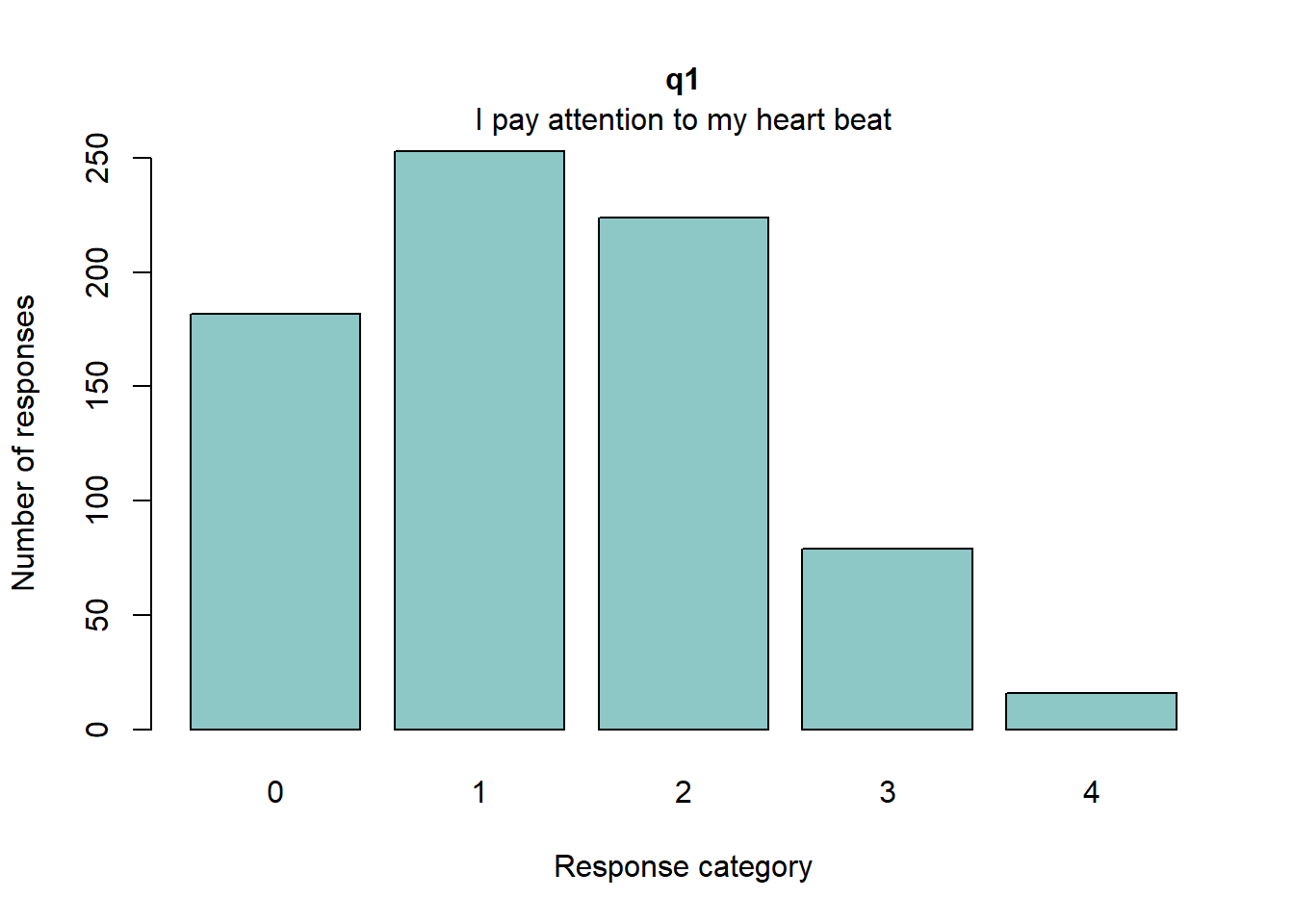

Supplement: Supplementary file 1 — Supplementary Material 1 [file 41598_2025_28073_MOESM1_ESM.zip › Supplementary/analysis_att_files/figure-html/unnamed-chunk-12-1.png]

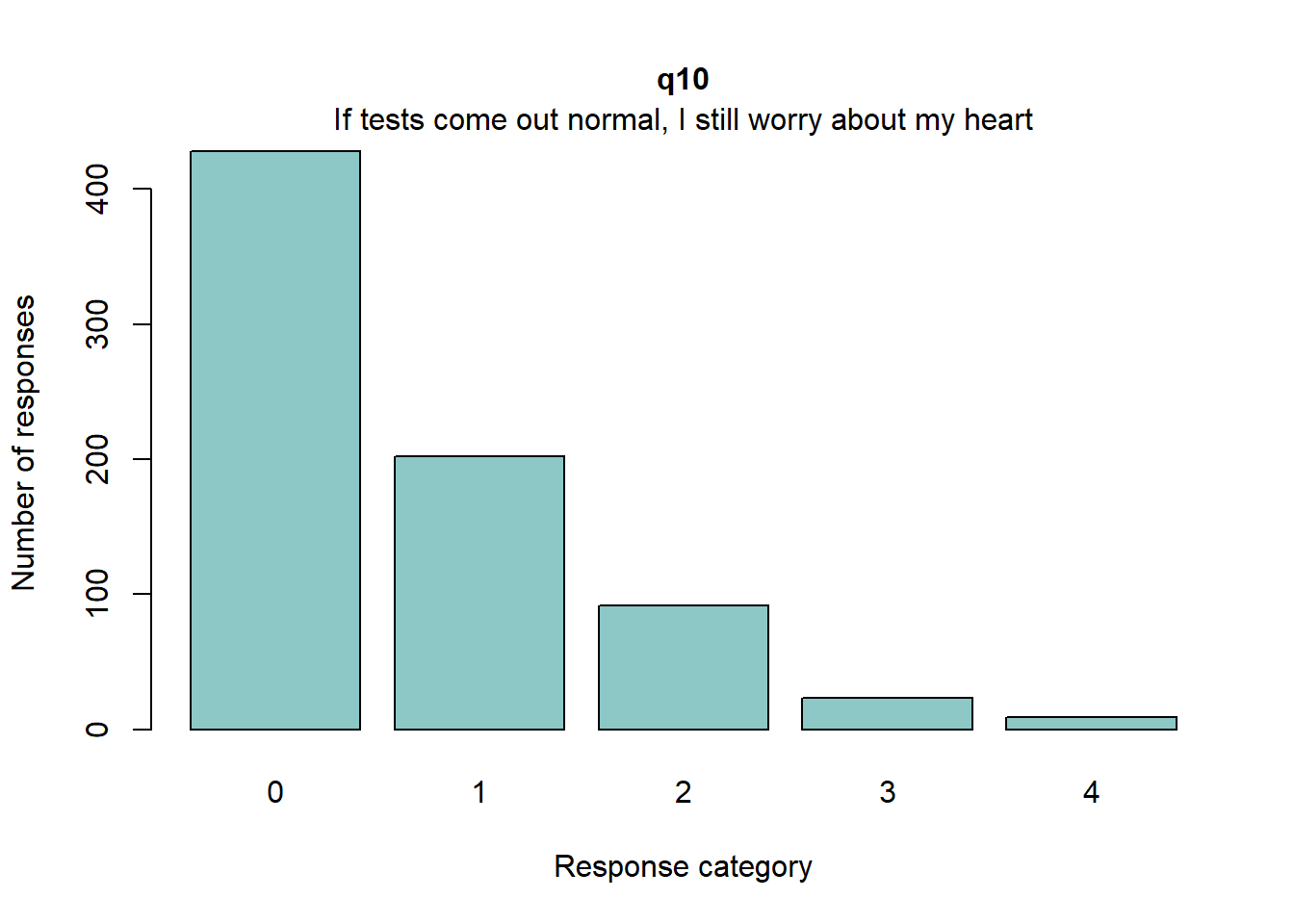

Supplement: Supplementary file 1 — Supplementary Material 1 [file 41598_2025_28073_MOESM1_ESM.zip › Supplementary/analysis_att_files/figure-html/unnamed-chunk-12-10.png]

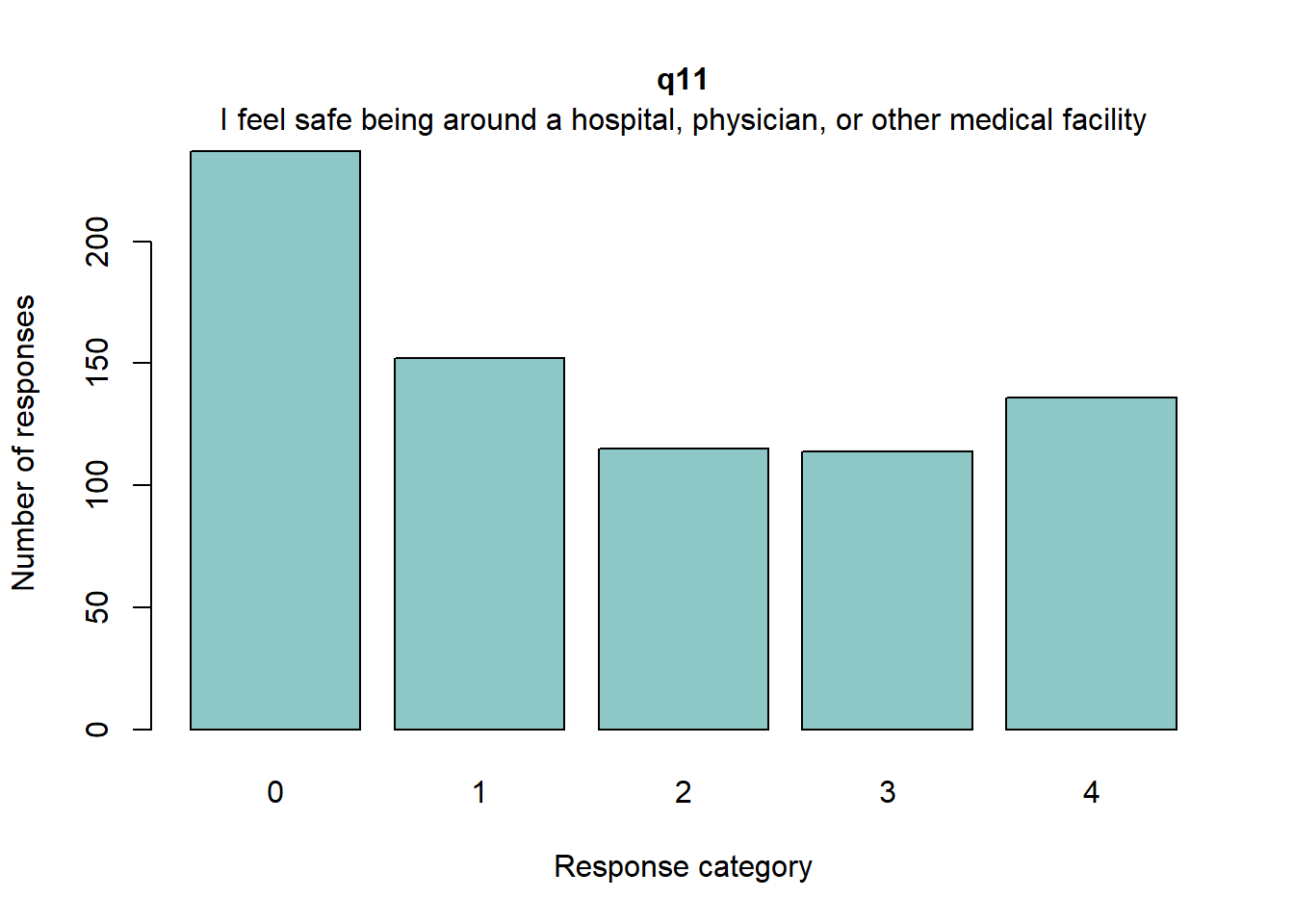

Supplement: Supplementary file 1 — Supplementary Material 1 [file 41598_2025_28073_MOESM1_ESM.zip › Supplementary/analysis_att_files/figure-html/unnamed-chunk-12-11.png]

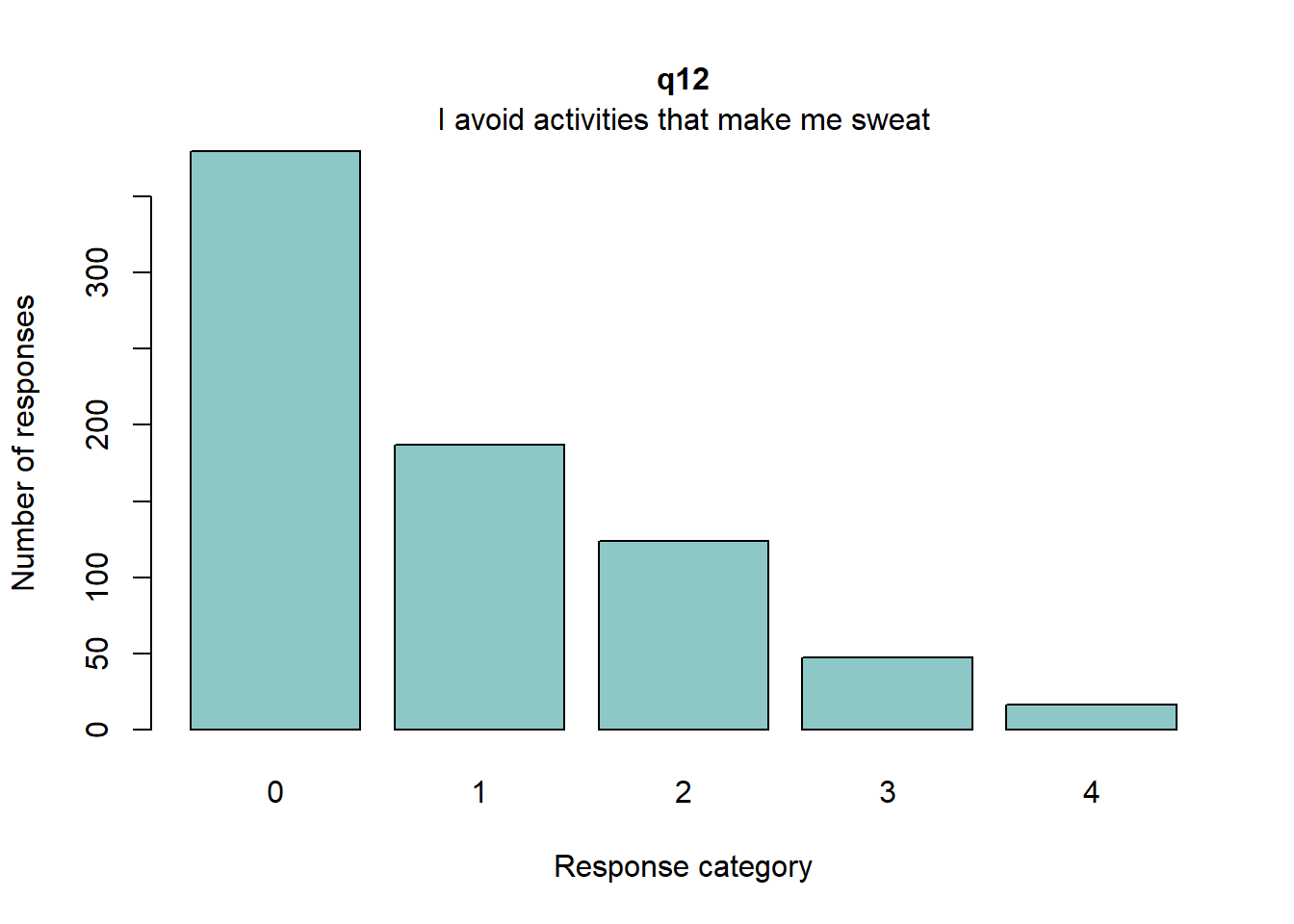

Supplement: Supplementary file 1 — Supplementary Material 1 [file 41598_2025_28073_MOESM1_ESM.zip › Supplementary/analysis_att_files/figure-html/unnamed-chunk-12-12.png]

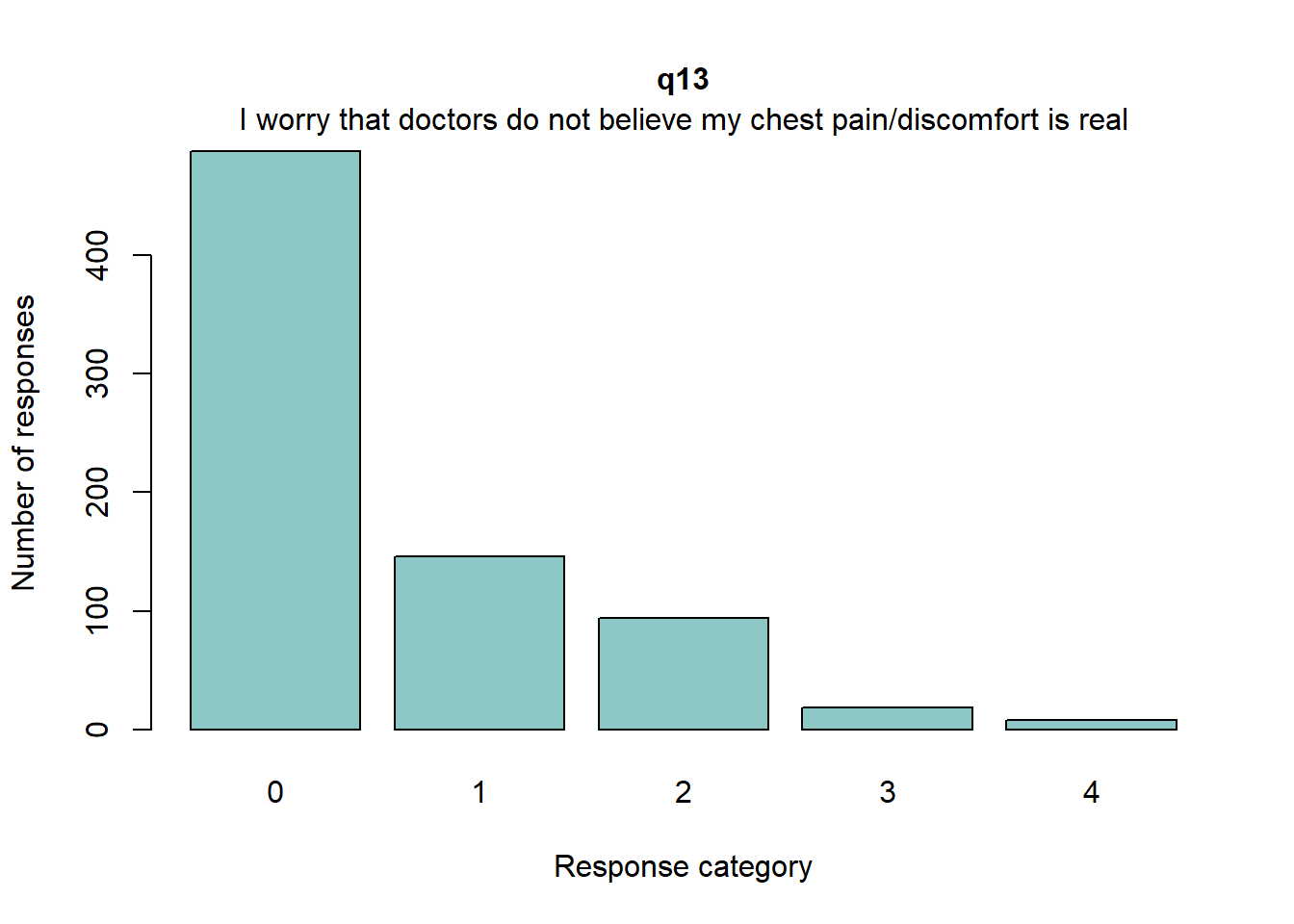

Supplement: Supplementary file 1 — Supplementary Material 1 [file 41598_2025_28073_MOESM1_ESM.zip › Supplementary/analysis_att_files/figure-html/unnamed-chunk-12-13.png]

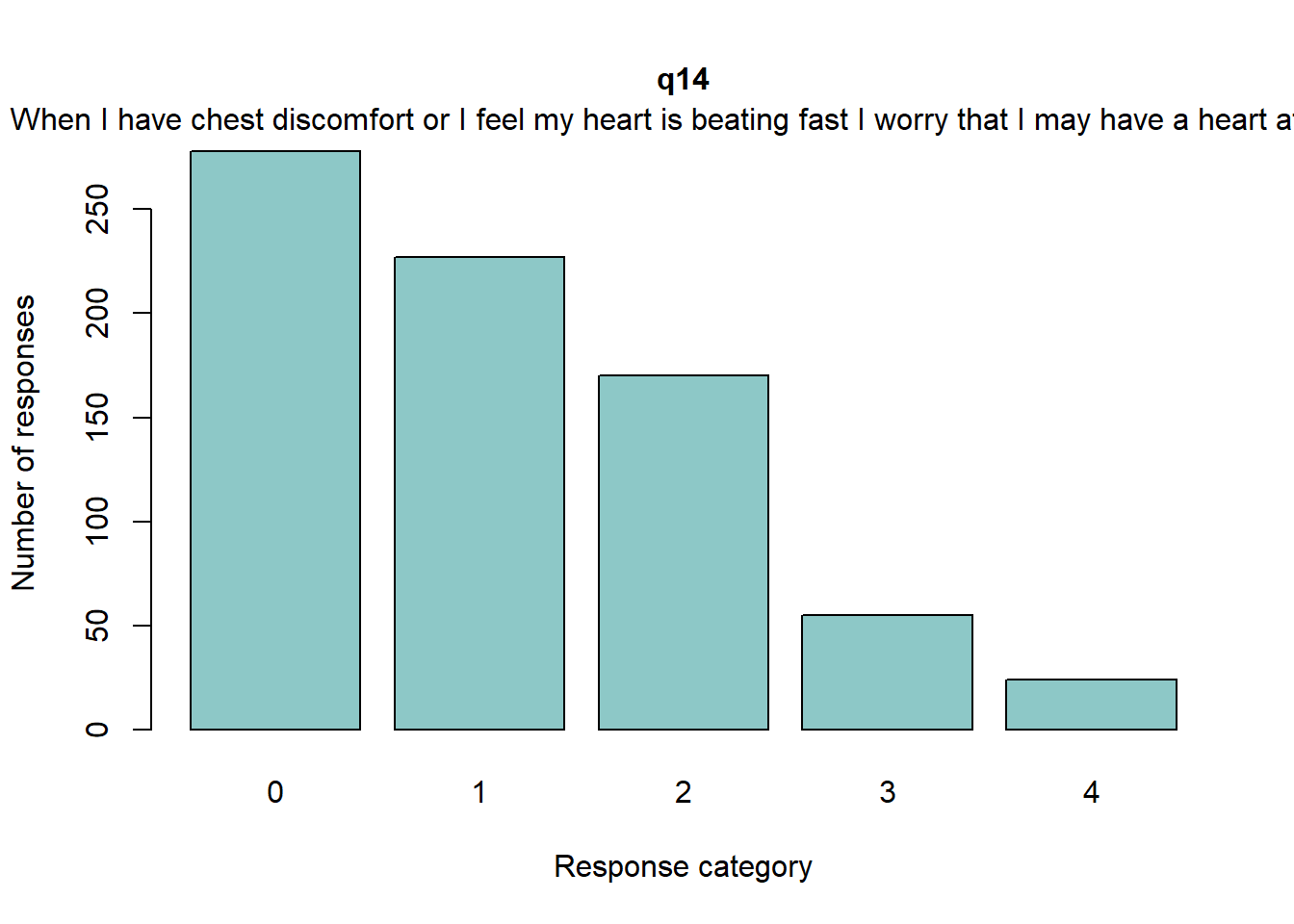

Supplement: Supplementary file 1 — Supplementary Material 1 [file 41598_2025_28073_MOESM1_ESM.zip › Supplementary/analysis_att_files/figure-html/unnamed-chunk-12-14.png]

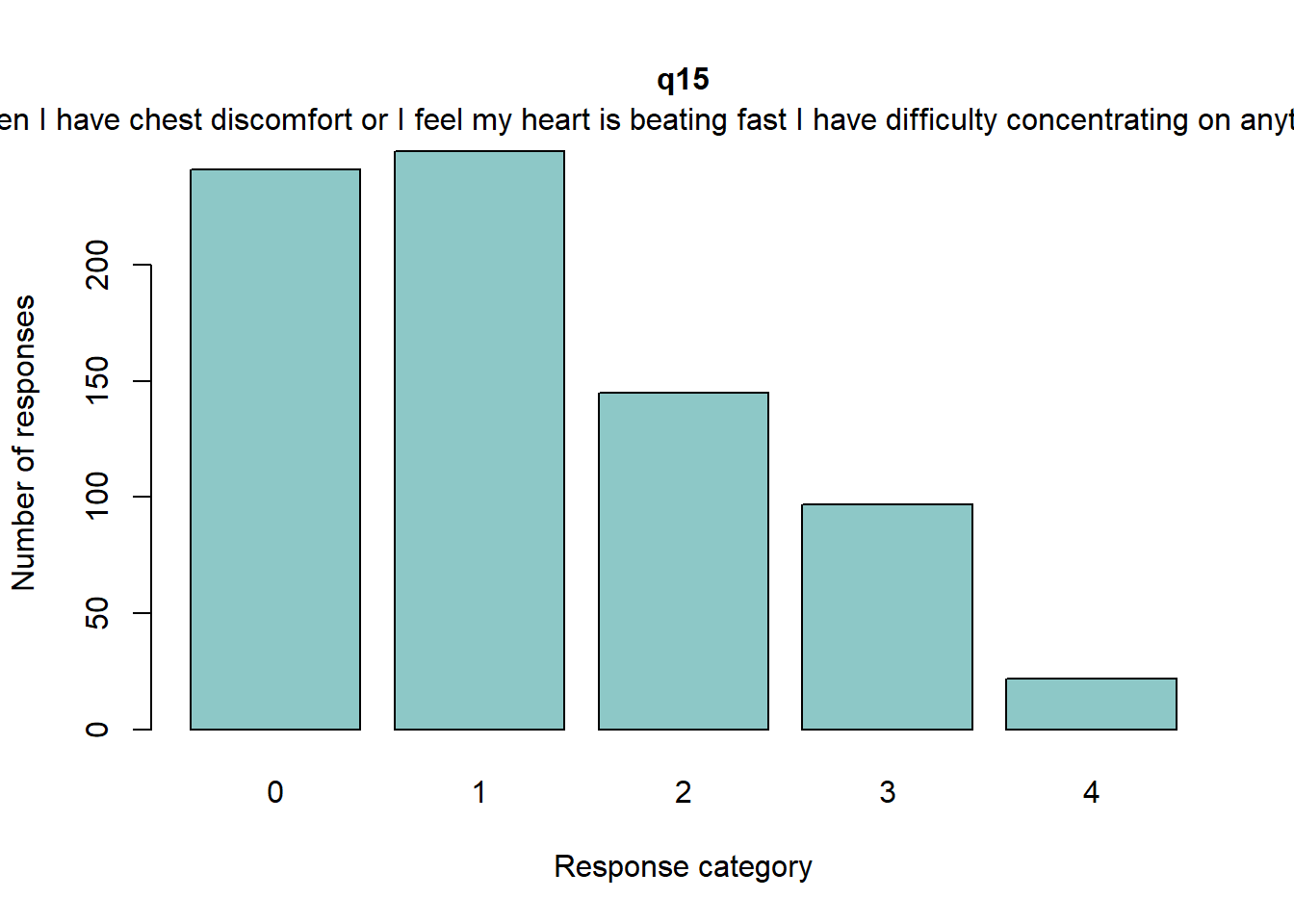

Supplement: Supplementary file 1 — Supplementary Material 1 [file 41598_2025_28073_MOESM1_ESM.zip › Supplementary/analysis_att_files/figure-html/unnamed-chunk-12-15.png]

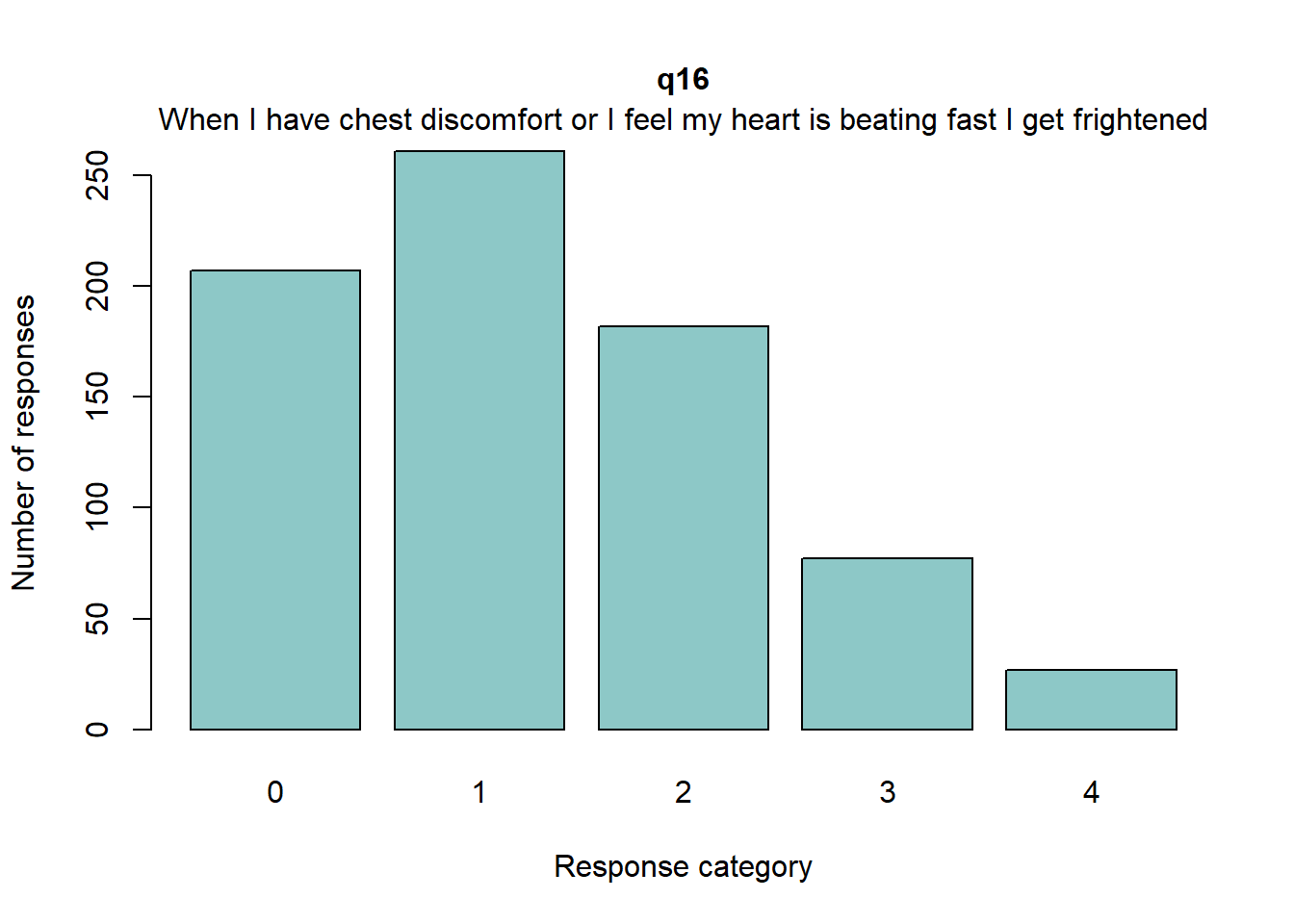

Supplement: Supplementary file 1 — Supplementary Material 1 [file 41598_2025_28073_MOESM1_ESM.zip › Supplementary/analysis_att_files/figure-html/unnamed-chunk-12-16.png]

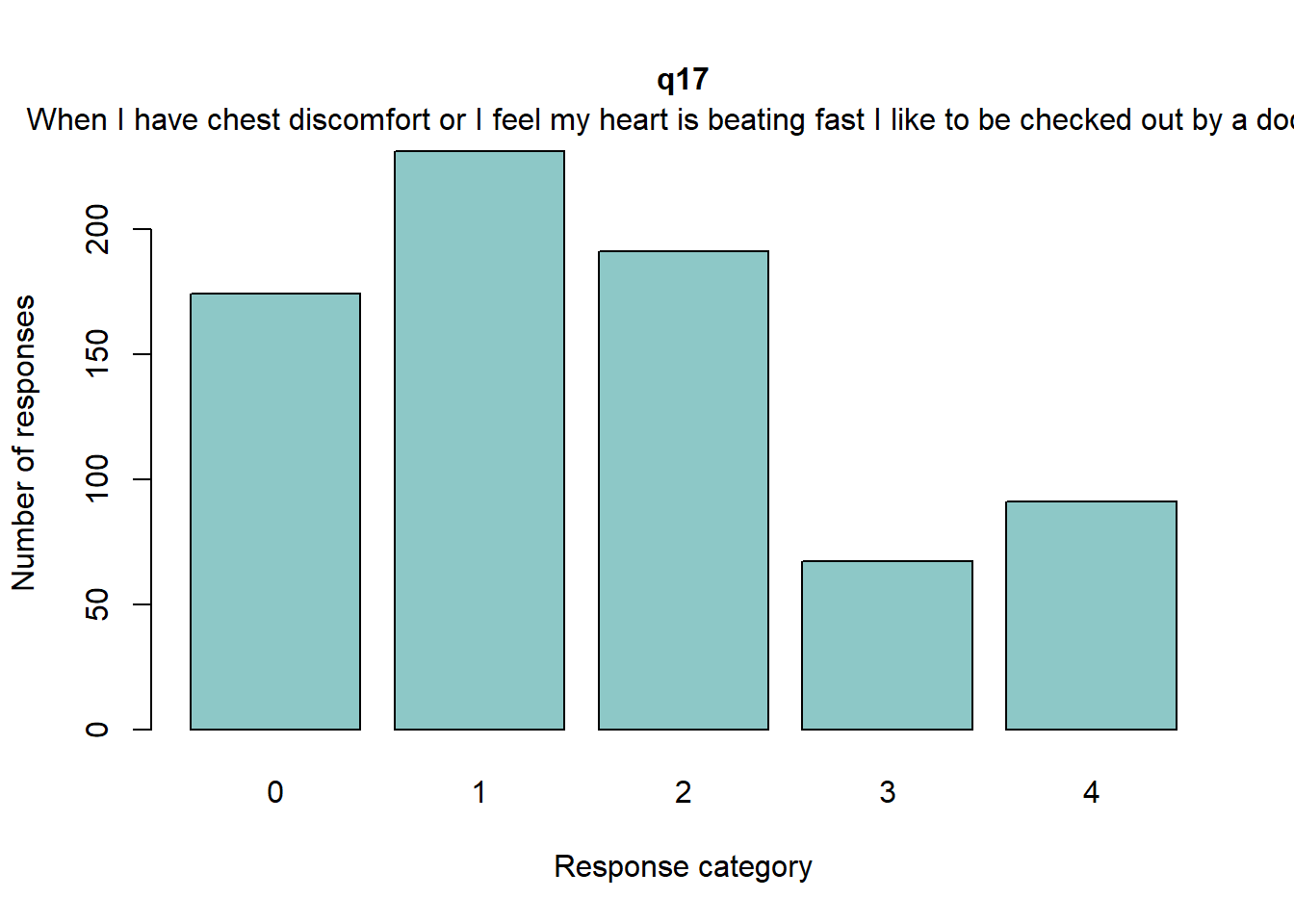

Supplement: Supplementary file 1 — Supplementary Material 1 [file 41598_2025_28073_MOESM1_ESM.zip › Supplementary/analysis_att_files/figure-html/unnamed-chunk-12-17.png]

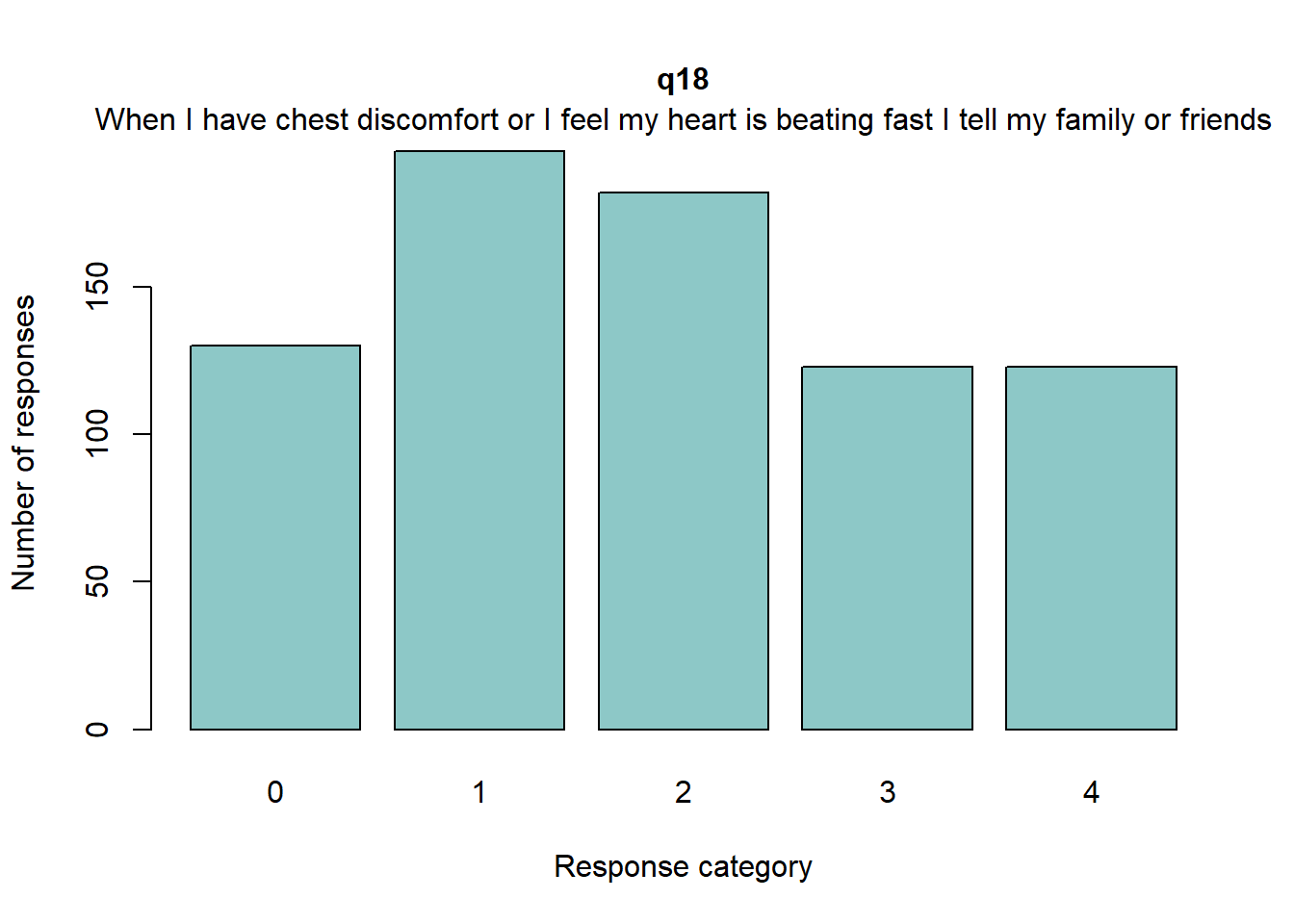

Supplement: Supplementary file 1 — Supplementary Material 1 [file 41598_2025_28073_MOESM1_ESM.zip › Supplementary/analysis_att_files/figure-html/unnamed-chunk-12-18.png]

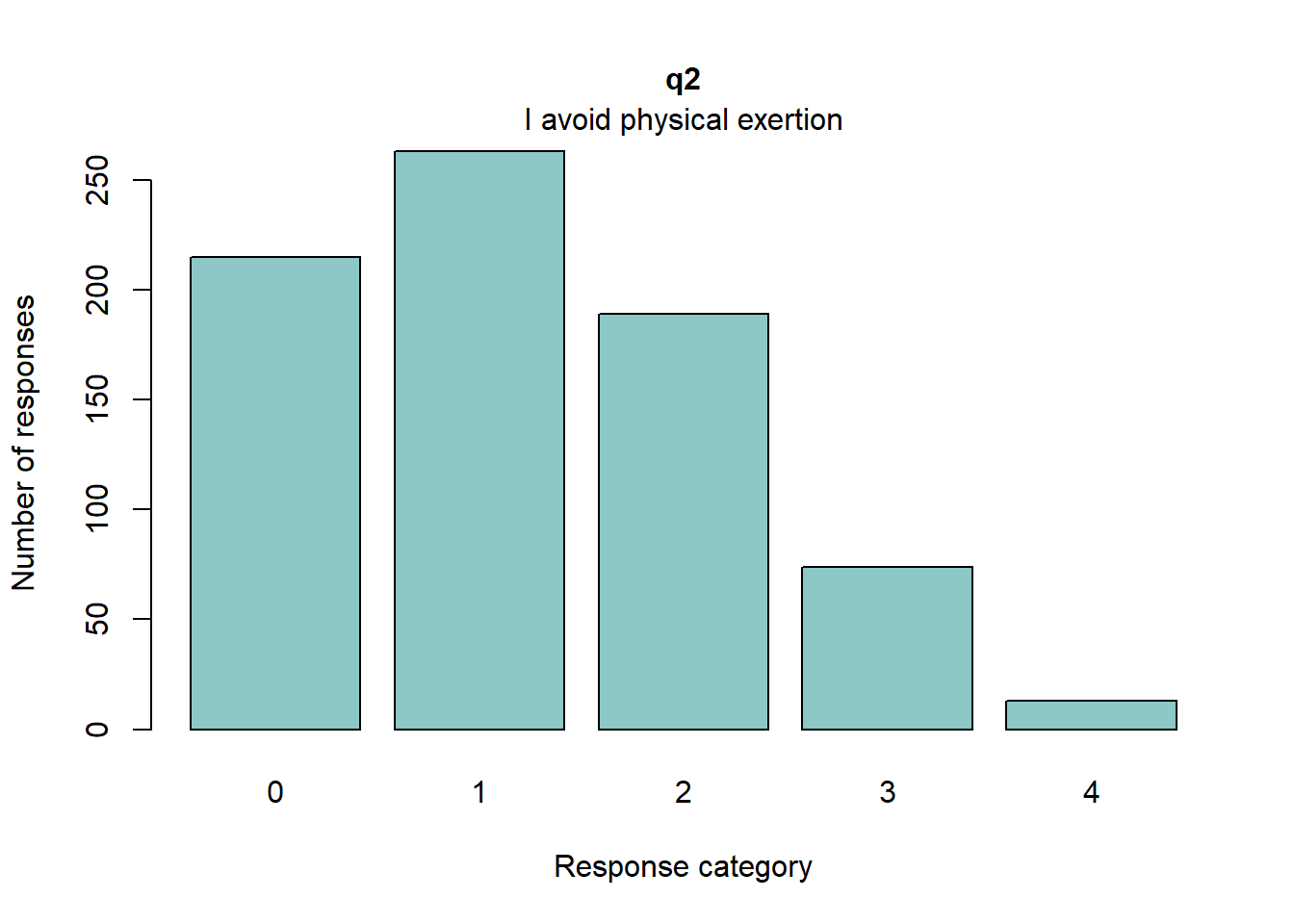

Supplement: Supplementary file 1 — Supplementary Material 1 [file 41598_2025_28073_MOESM1_ESM.zip › Supplementary/analysis_att_files/figure-html/unnamed-chunk-12-2.png]

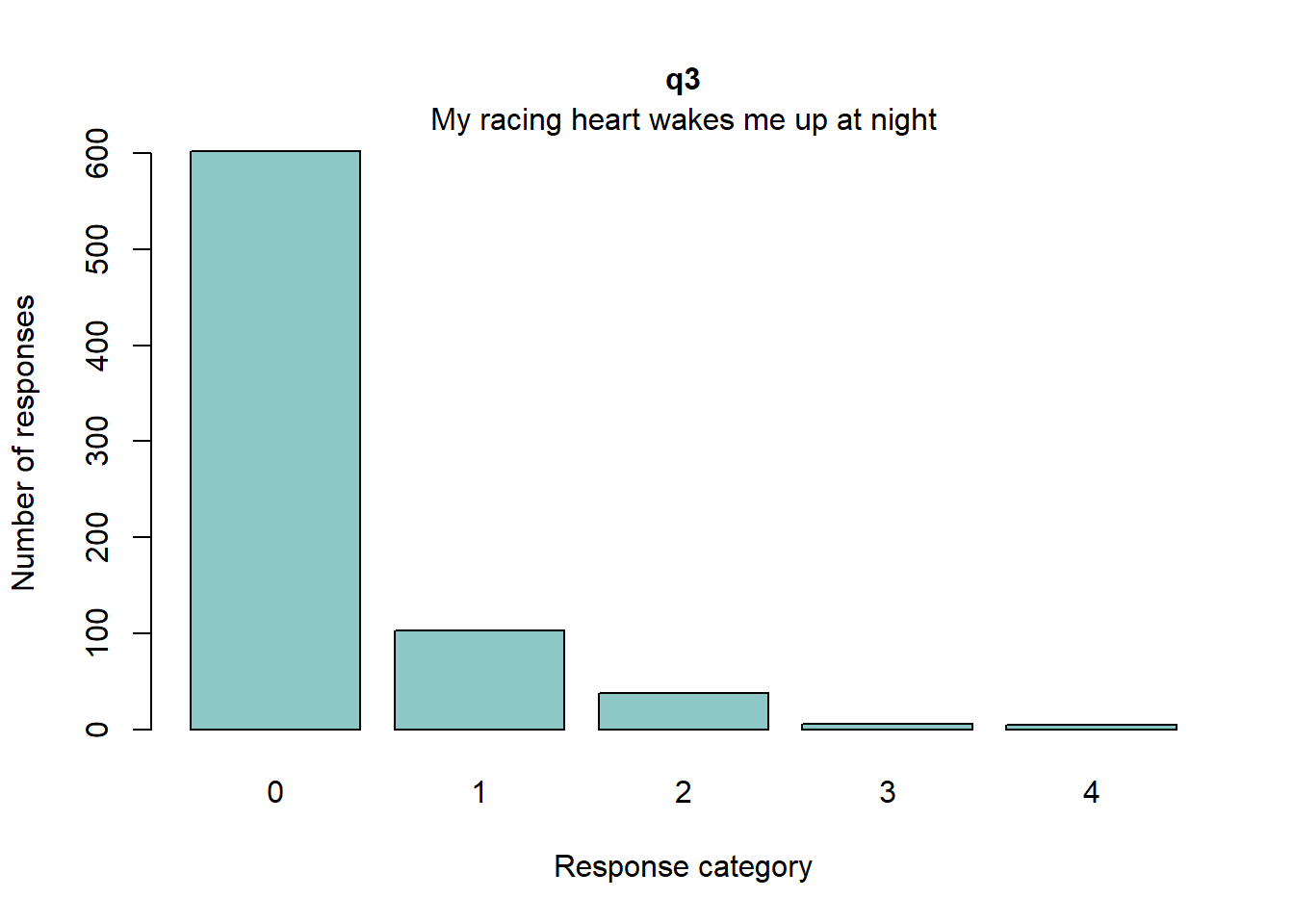

Supplement: Supplementary file 1 — Supplementary Material 1 [file 41598_2025_28073_MOESM1_ESM.zip › Supplementary/analysis_att_files/figure-html/unnamed-chunk-12-3.png]

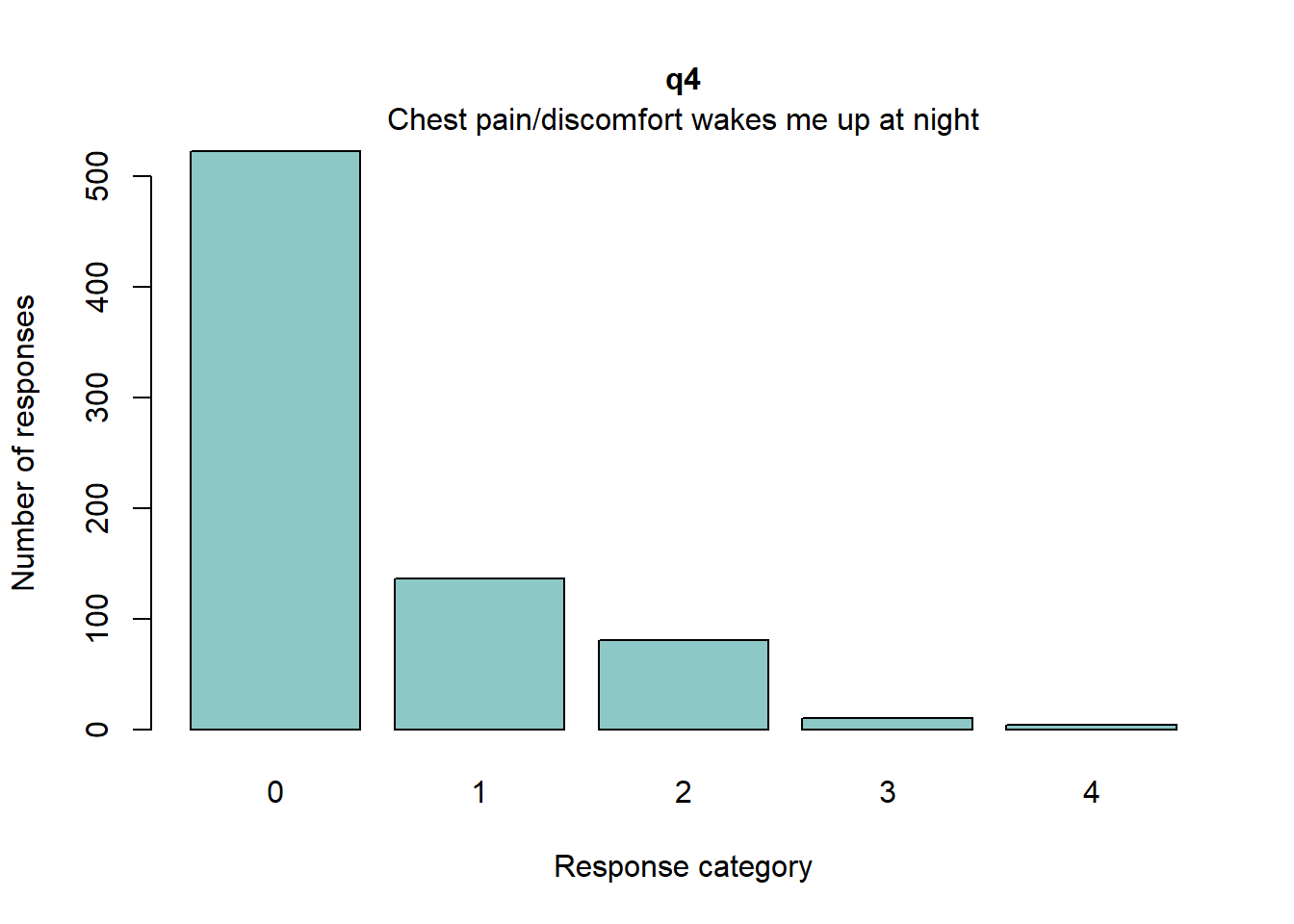

Supplement: Supplementary file 1 — Supplementary Material 1 [file 41598_2025_28073_MOESM1_ESM.zip › Supplementary/analysis_att_files/figure-html/unnamed-chunk-12-4.png]

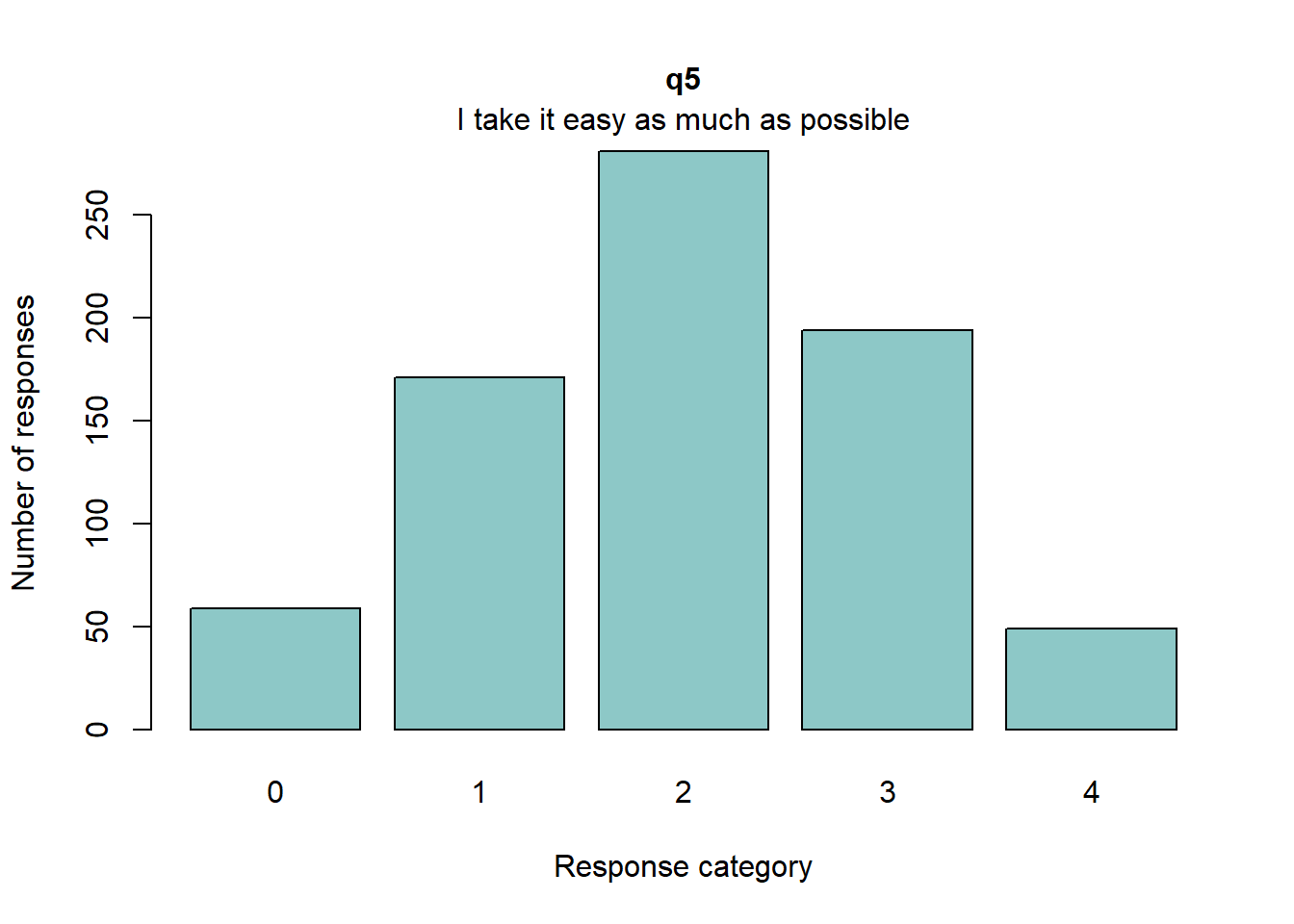

Supplement: Supplementary file 1 — Supplementary Material 1 [file 41598_2025_28073_MOESM1_ESM.zip › Supplementary/analysis_att_files/figure-html/unnamed-chunk-12-5.png]

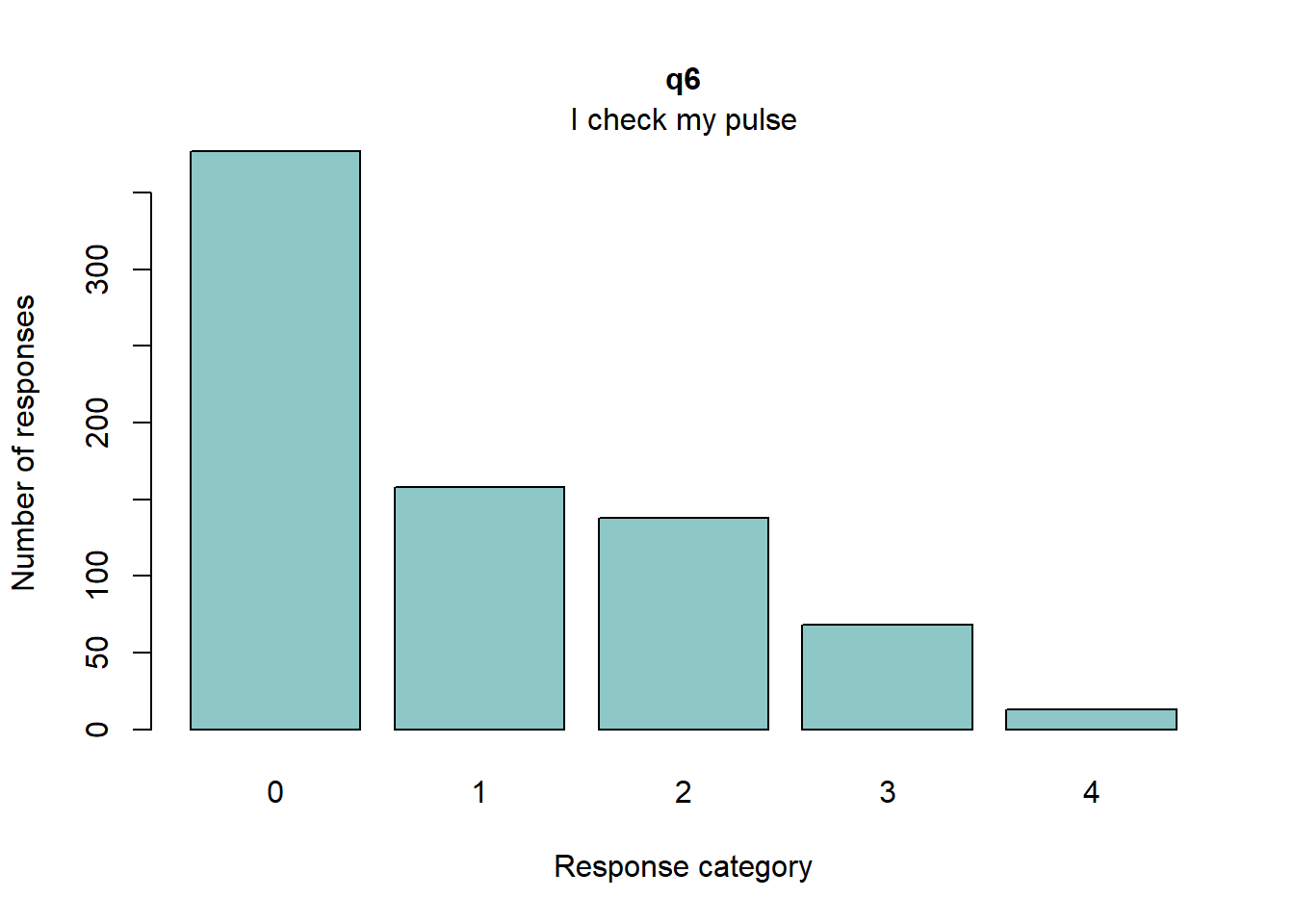

Supplement: Supplementary file 1 — Supplementary Material 1 [file 41598_2025_28073_MOESM1_ESM.zip › Supplementary/analysis_att_files/figure-html/unnamed-chunk-12-6.png]

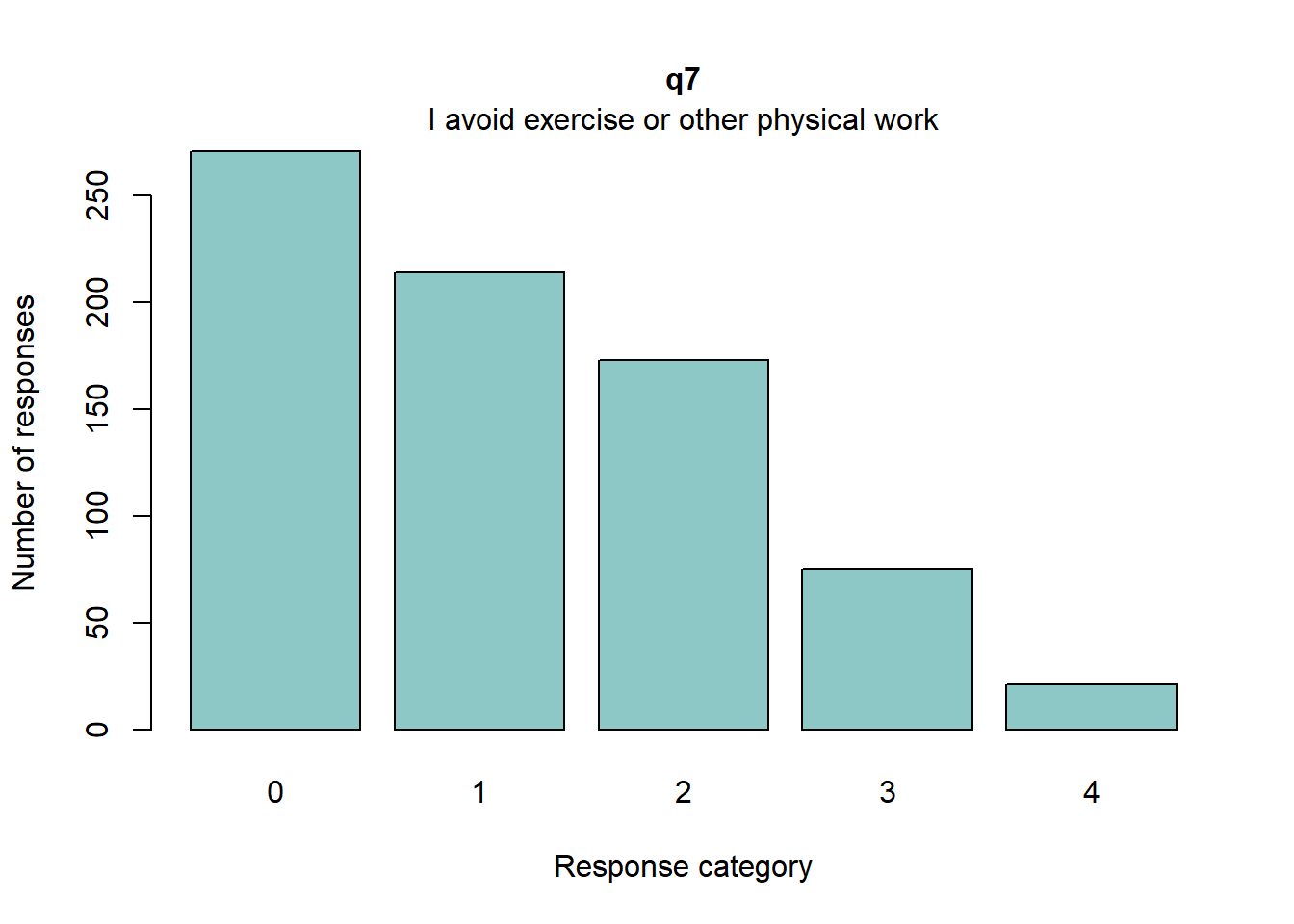

Supplement: Supplementary file 1 — Supplementary Material 1 [file 41598_2025_28073_MOESM1_ESM.zip › Supplementary/analysis_att_files/figure-html/unnamed-chunk-12-7.png]

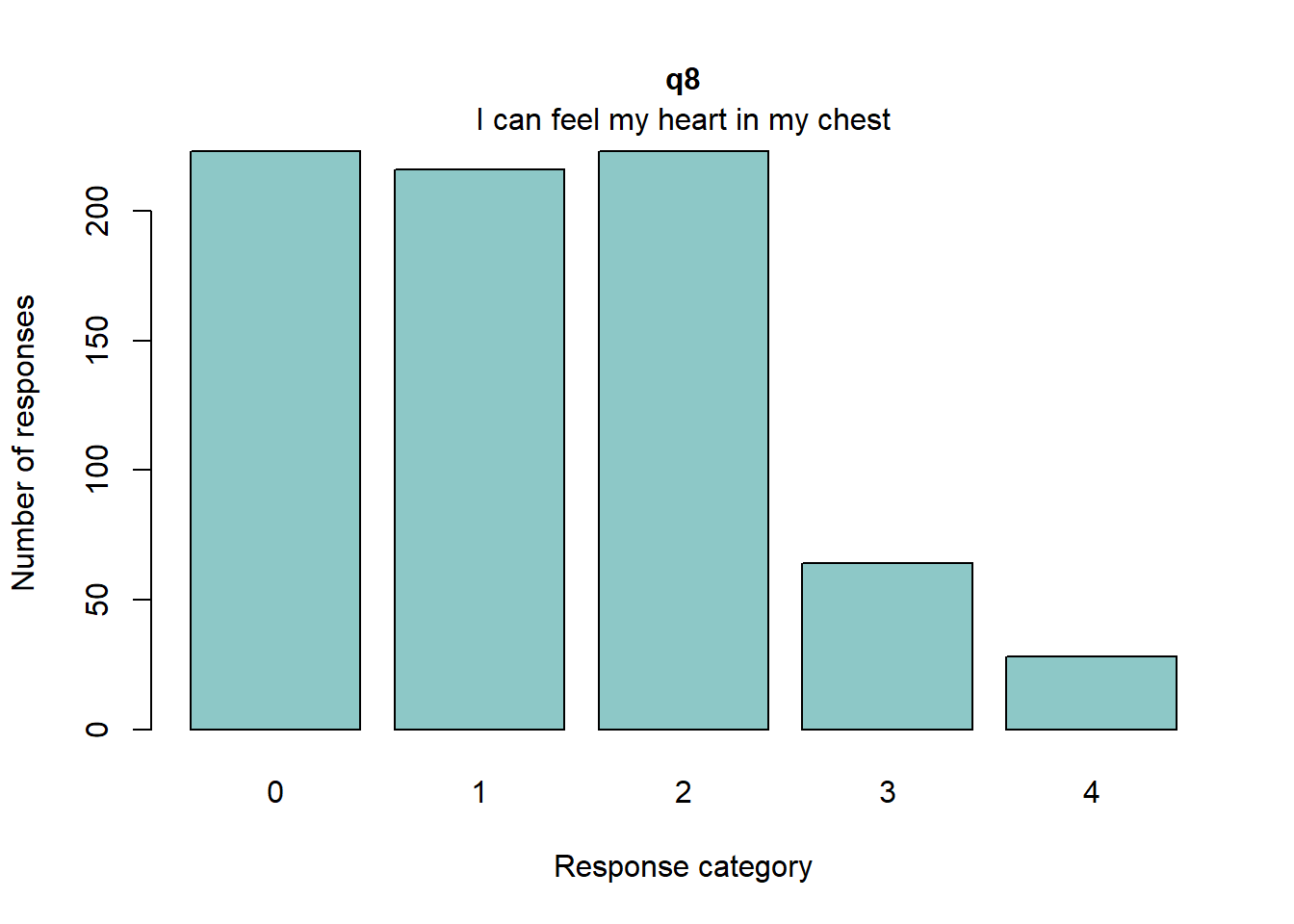

Supplement: Supplementary file 1 — Supplementary Material 1 [file 41598_2025_28073_MOESM1_ESM.zip › Supplementary/analysis_att_files/figure-html/unnamed-chunk-12-8.png]

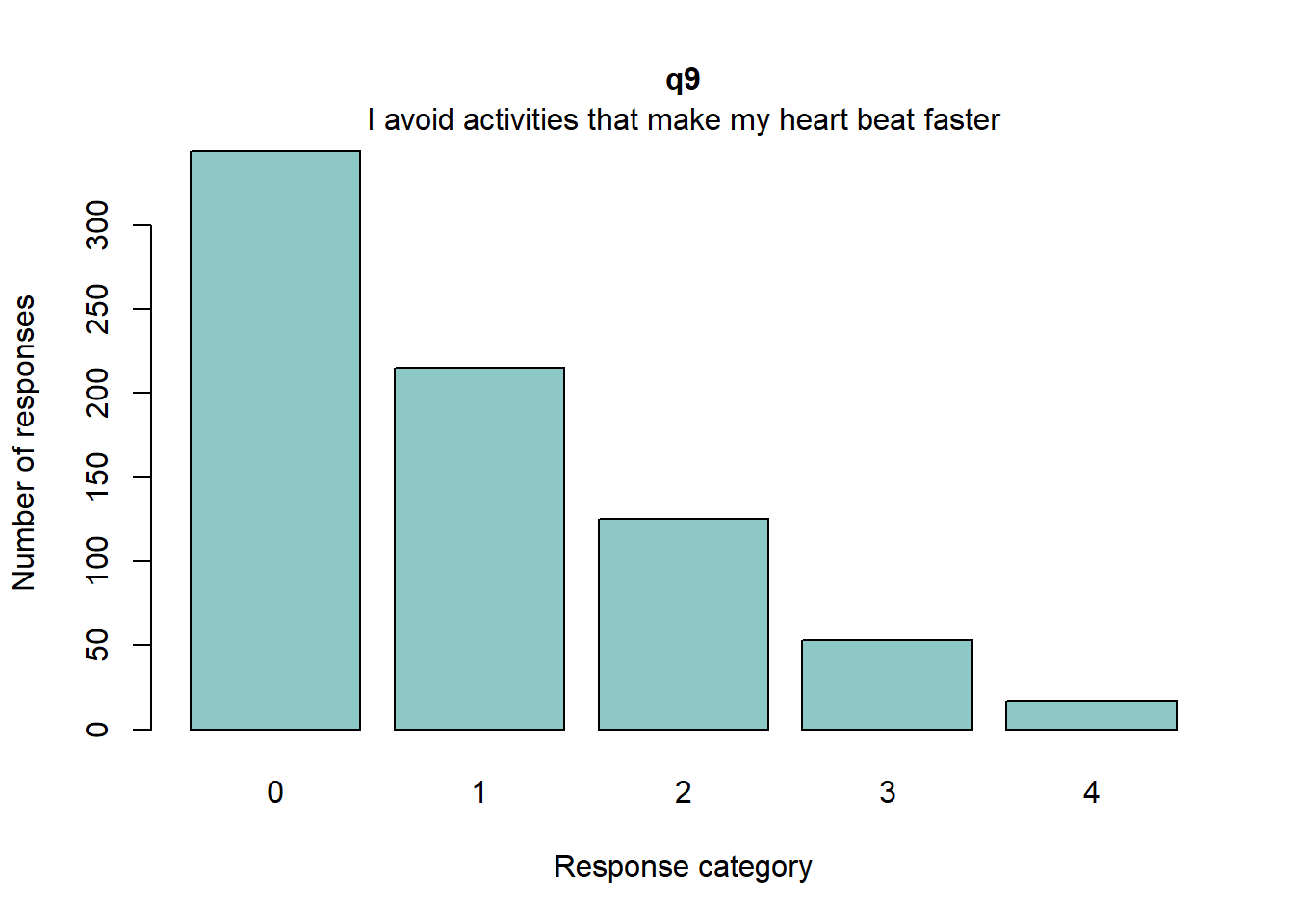

Supplement: Supplementary file 1 — Supplementary Material 1 [file 41598_2025_28073_MOESM1_ESM.zip › Supplementary/analysis_att_files/figure-html/unnamed-chunk-12-9.png]

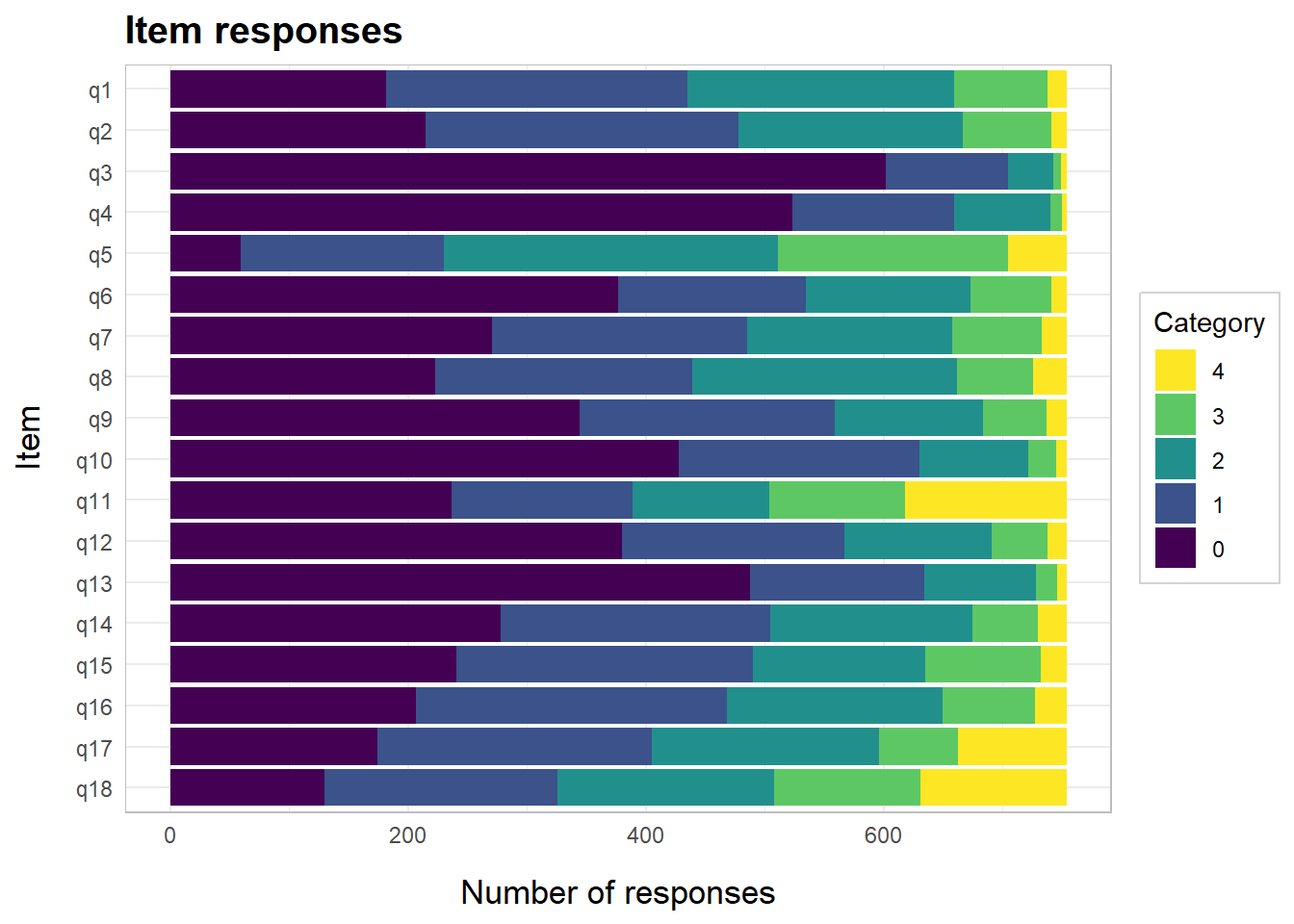

Supplement: Supplementary file 1 — Supplementary Material 1 [file 41598_2025_28073_MOESM1_ESM.zip › Supplementary/analysis_att_files/figure-html/unnamed-chunk-13-1.png]

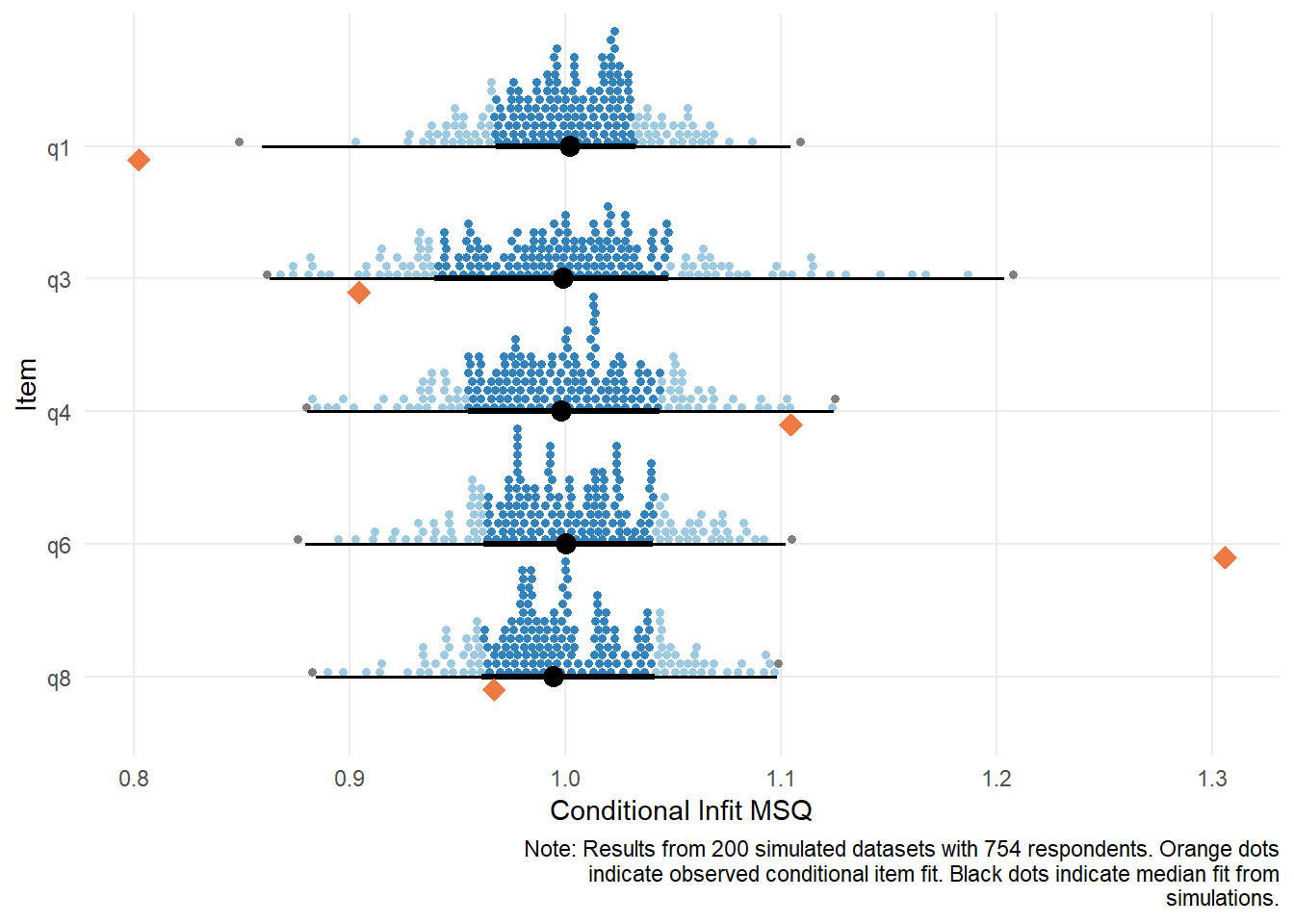

Supplement: Supplementary file 1 — Supplementary Material 1 [file 41598_2025_28073_MOESM1_ESM.zip › Supplementary/analysis_att_files/figure-html/unnamed-chunk-17-1.png]

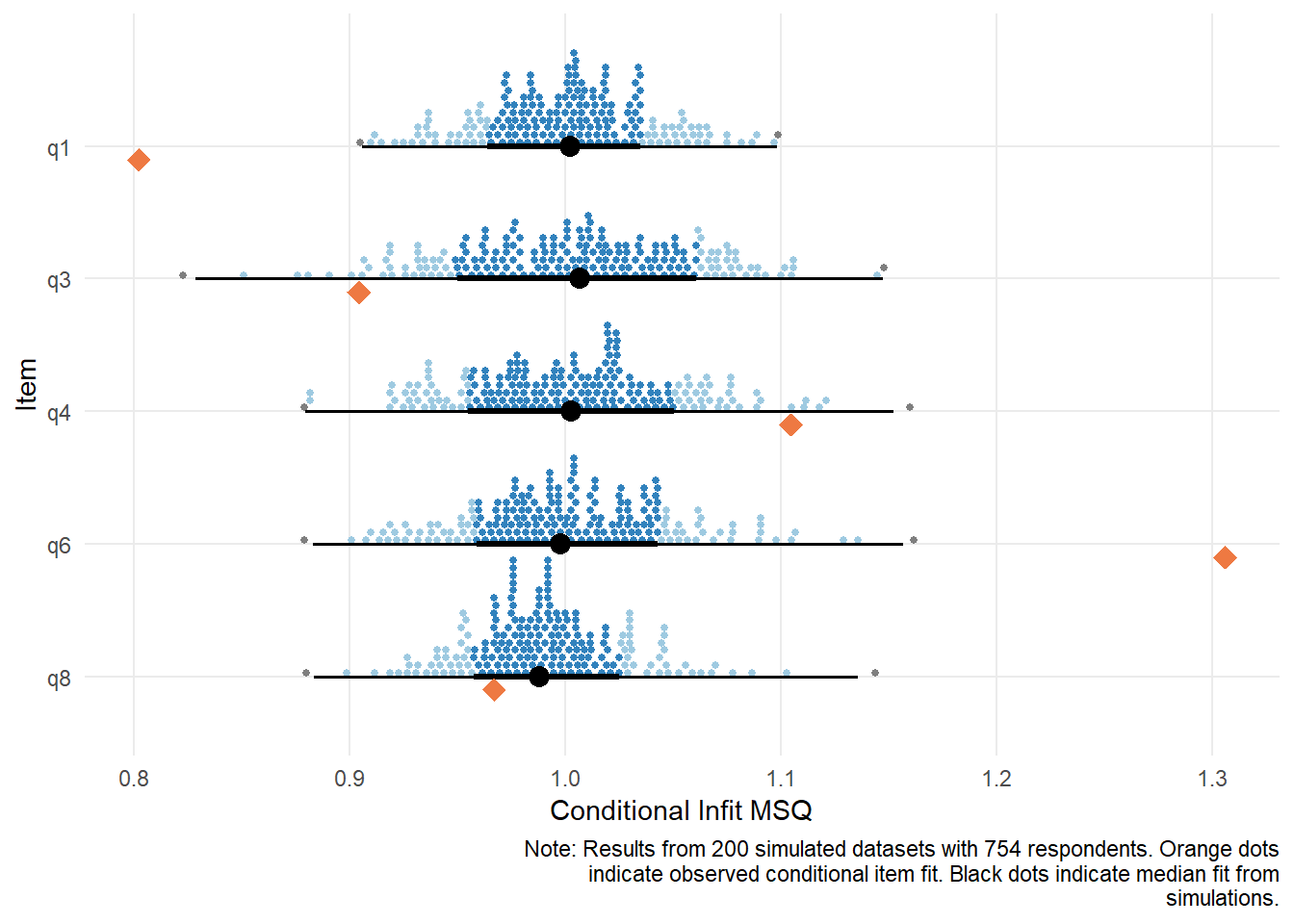

Supplement: Supplementary file 1 — Supplementary Material 1 [file 41598_2025_28073_MOESM1_ESM.zip › Supplementary/analysis_att_files/figure-html/unnamed-chunk-18-1.png]

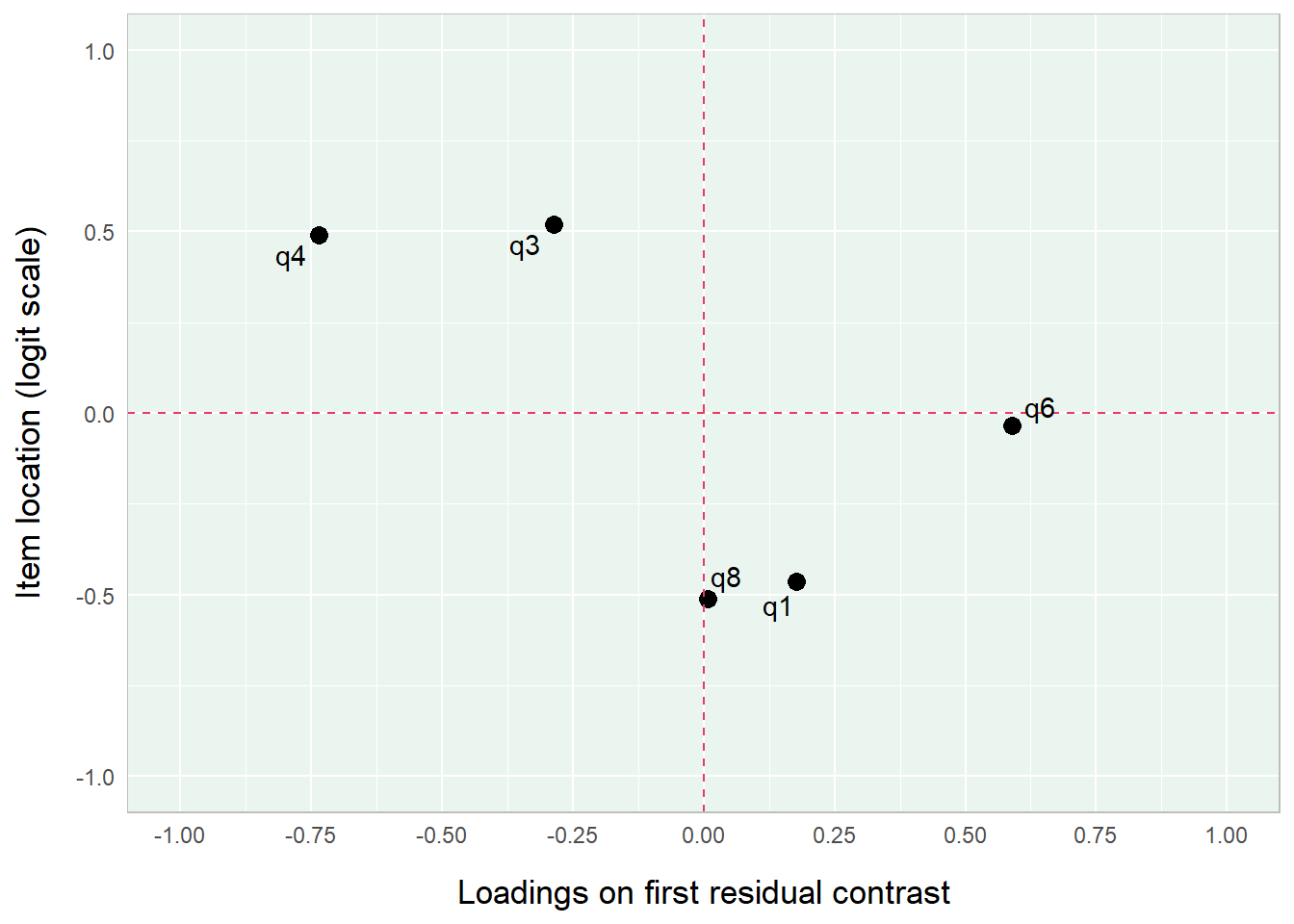

Supplement: Supplementary file 1 — Supplementary Material 1 [file 41598_2025_28073_MOESM1_ESM.zip › Supplementary/analysis_att_files/figure-html/unnamed-chunk-22-1.png]

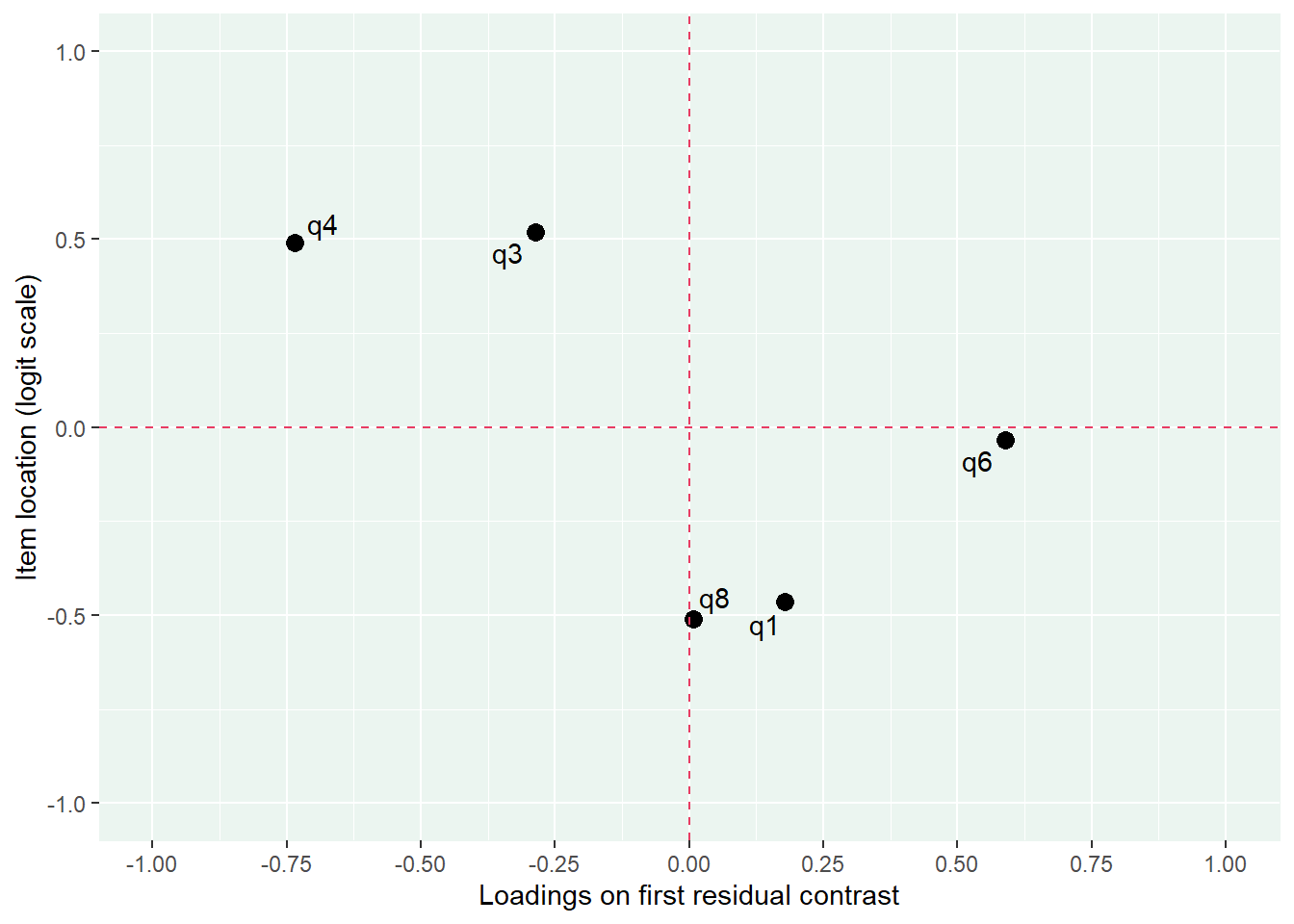

Supplement: Supplementary file 1 — Supplementary Material 1 [file 41598_2025_28073_MOESM1_ESM.zip › Supplementary/analysis_att_files/figure-html/unnamed-chunk-23-1.png]

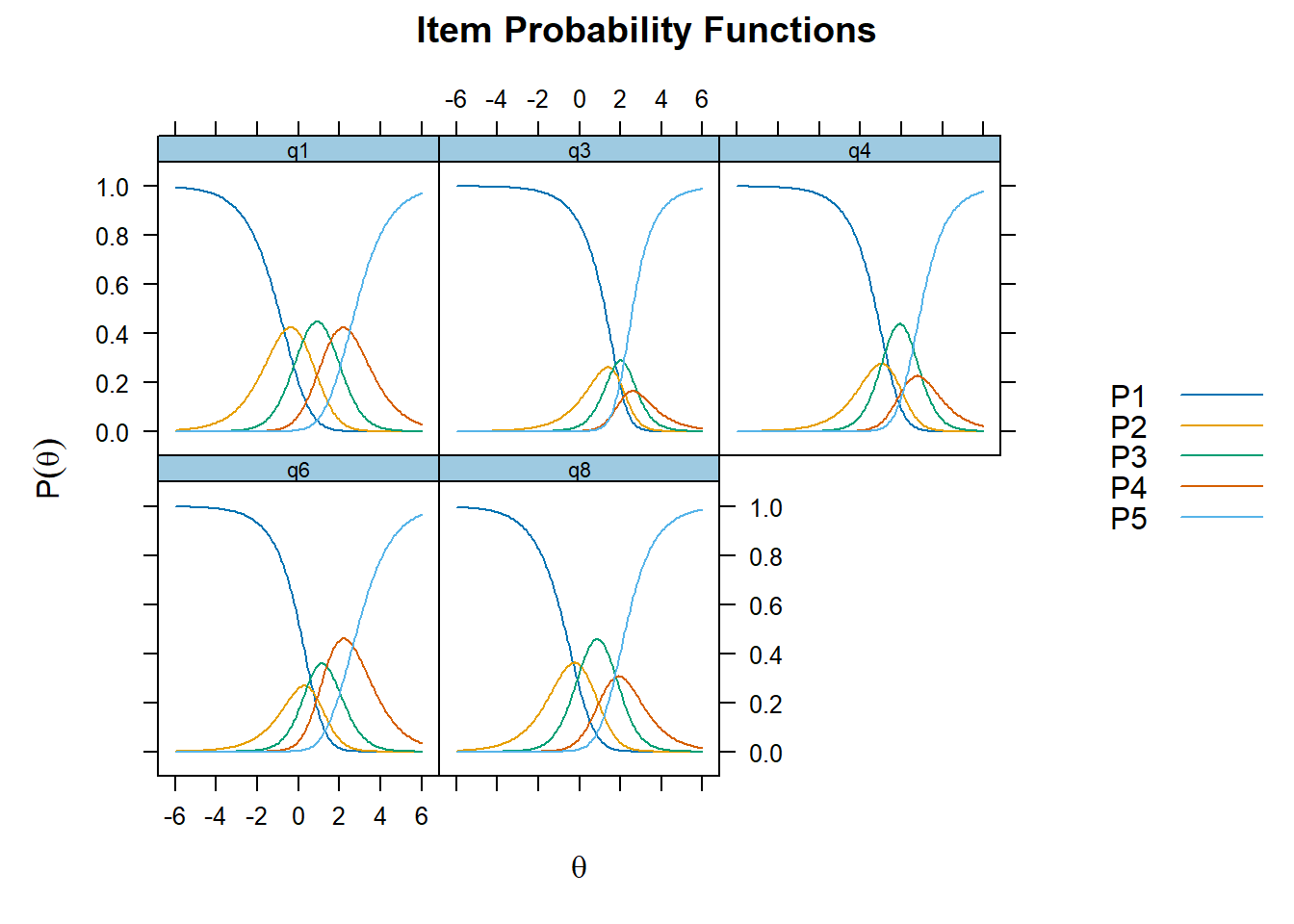

Supplement: Supplementary file 1 — Supplementary Material 1 [file 41598_2025_28073_MOESM1_ESM.zip › Supplementary/analysis_att_files/figure-html/unnamed-chunk-24-1.png]

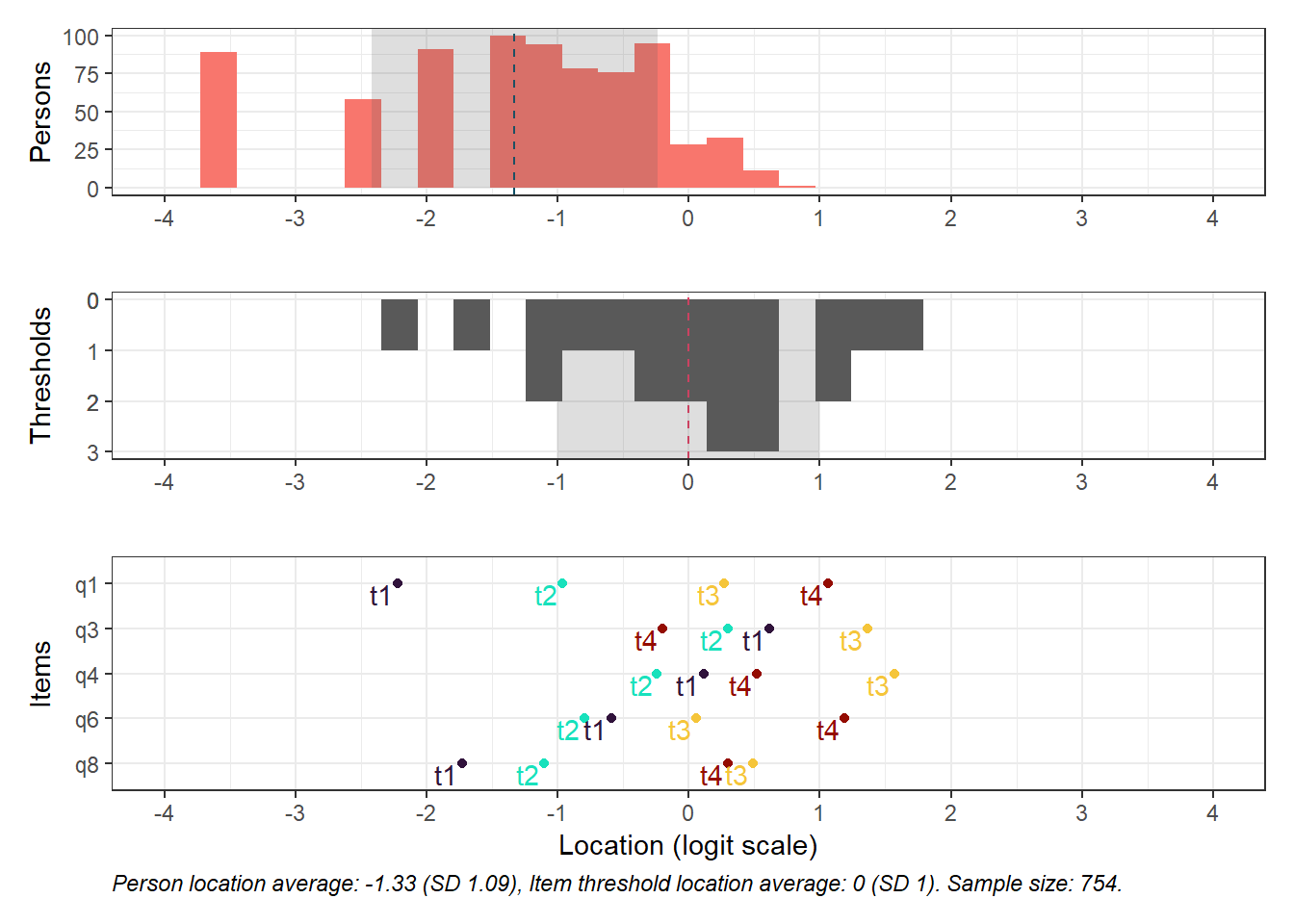

Supplement: Supplementary file 1 — Supplementary Material 1 [file 41598_2025_28073_MOESM1_ESM.zip › Supplementary/analysis_att_files/figure-html/unnamed-chunk-25-1.png]

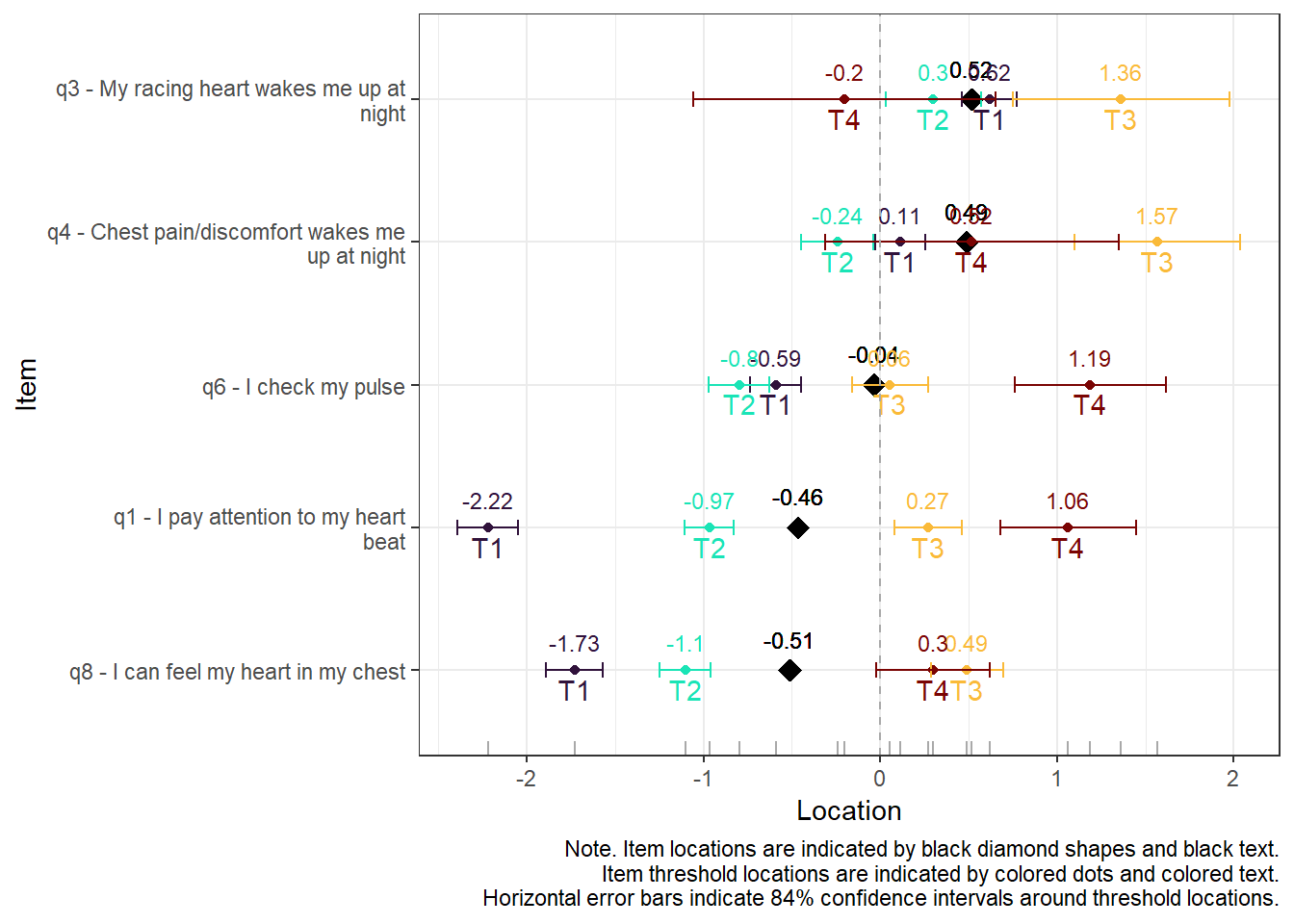

Supplement: Supplementary file 1 — Supplementary Material 1 [file 41598_2025_28073_MOESM1_ESM.zip › Supplementary/analysis_att_files/figure-html/unnamed-chunk-26-1.png]

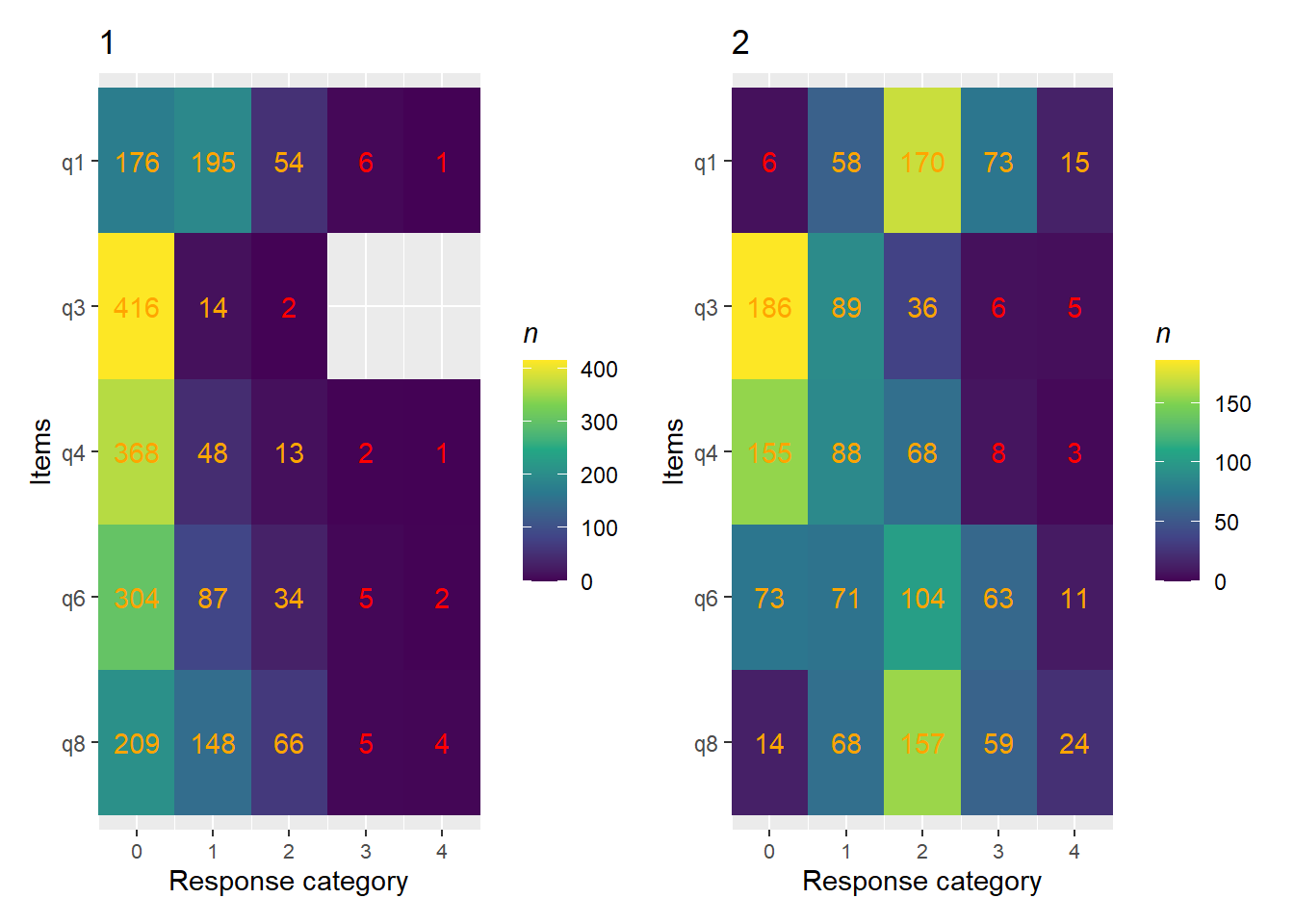

Supplement: Supplementary file 1 — Supplementary Material 1 [file 41598_2025_28073_MOESM1_ESM.zip › Supplementary/analysis_att_files/figure-html/unnamed-chunk-27-1.png]

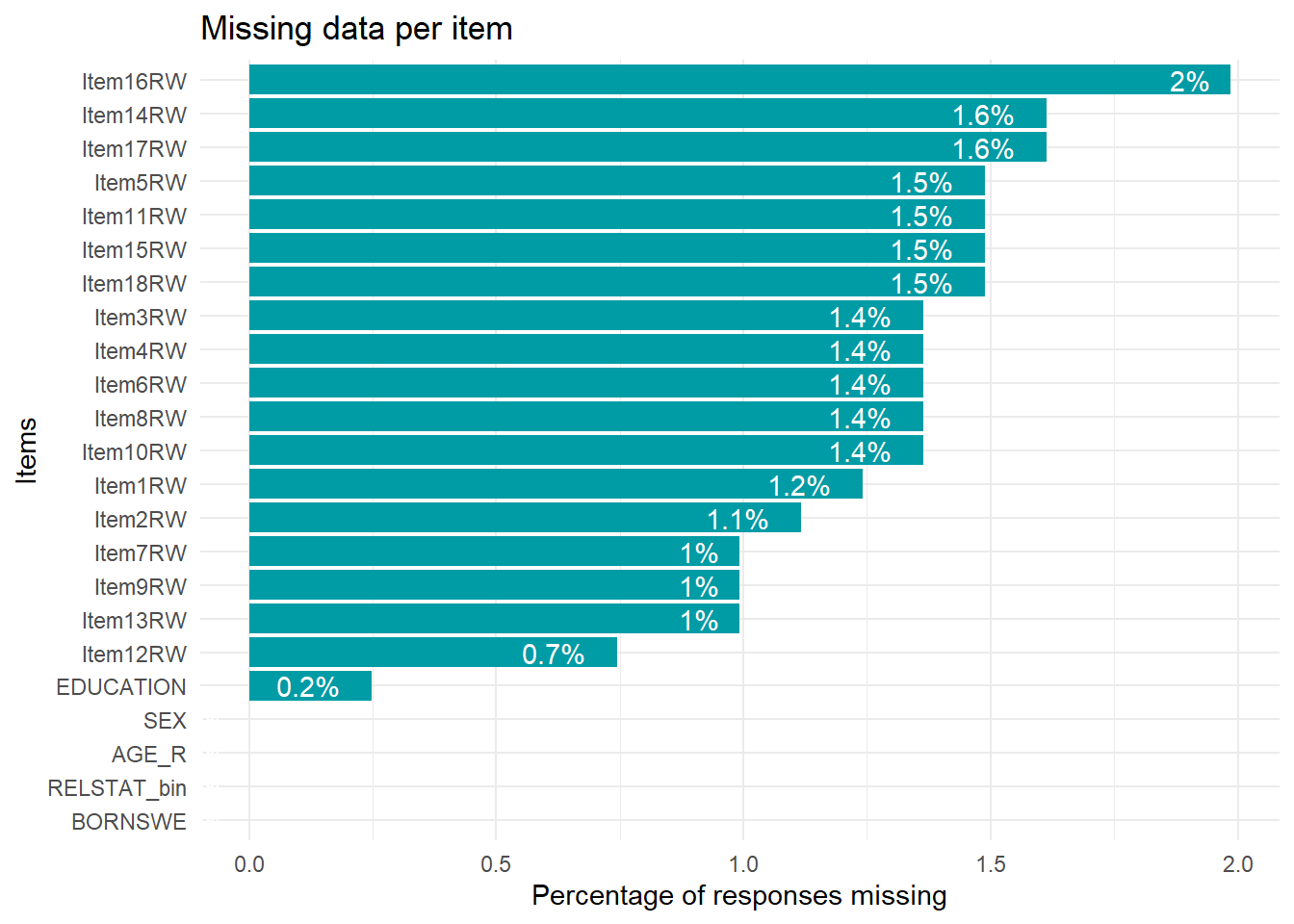

Supplement: Supplementary file 1 — Supplementary Material 1 [file 41598_2025_28073_MOESM1_ESM.zip › Supplementary/analysis_att_files/figure-html/unnamed-chunk-3-1.png]

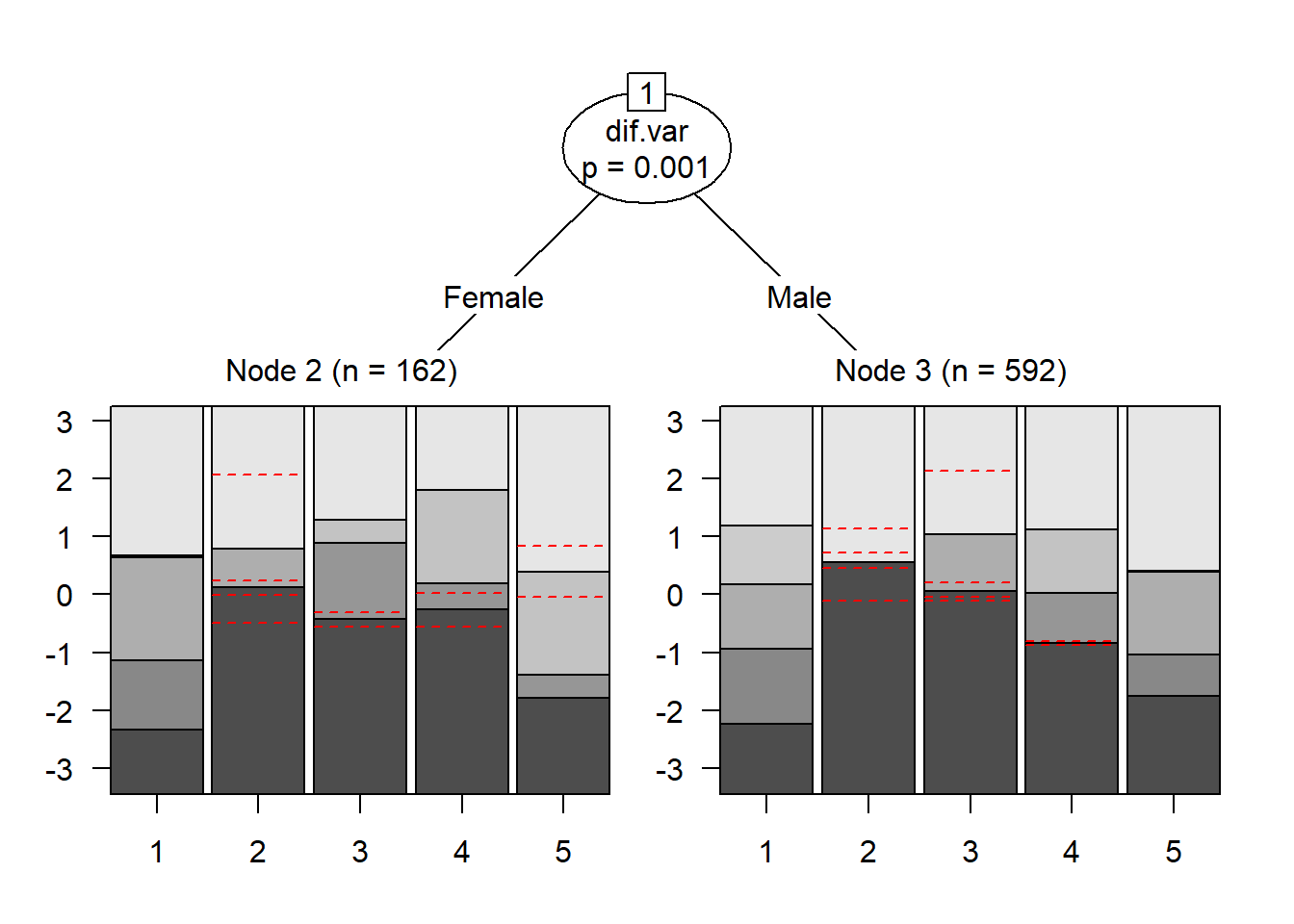

Supplement: Supplementary file 1 — Supplementary Material 1 [file 41598_2025_28073_MOESM1_ESM.zip › Supplementary/analysis_att_files/figure-html/unnamed-chunk-30-1.png]

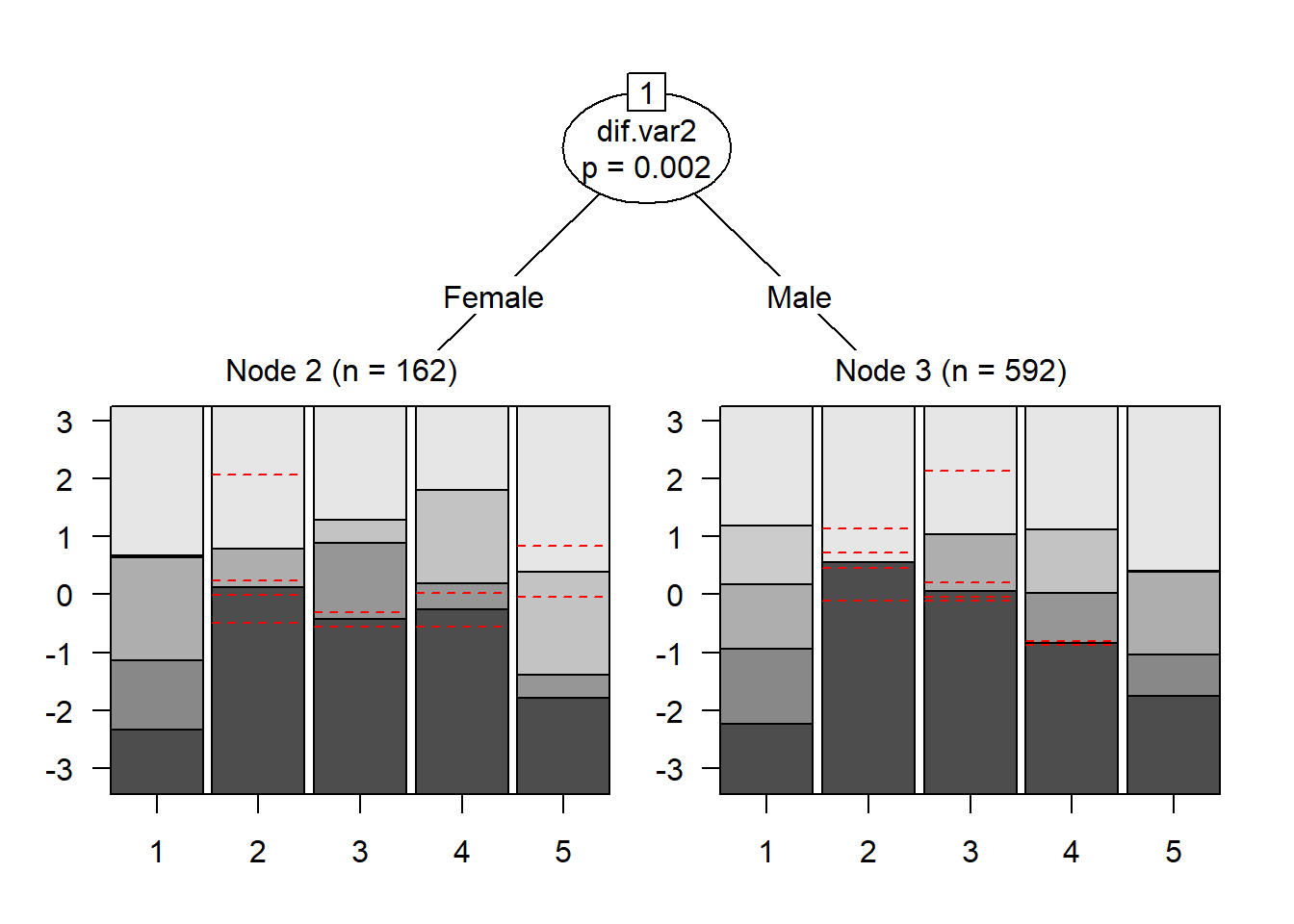

Supplement: Supplementary file 1 — Supplementary Material 1 [file 41598_2025_28073_MOESM1_ESM.zip › Supplementary/analysis_att_files/figure-html/unnamed-chunk-32-1.png]

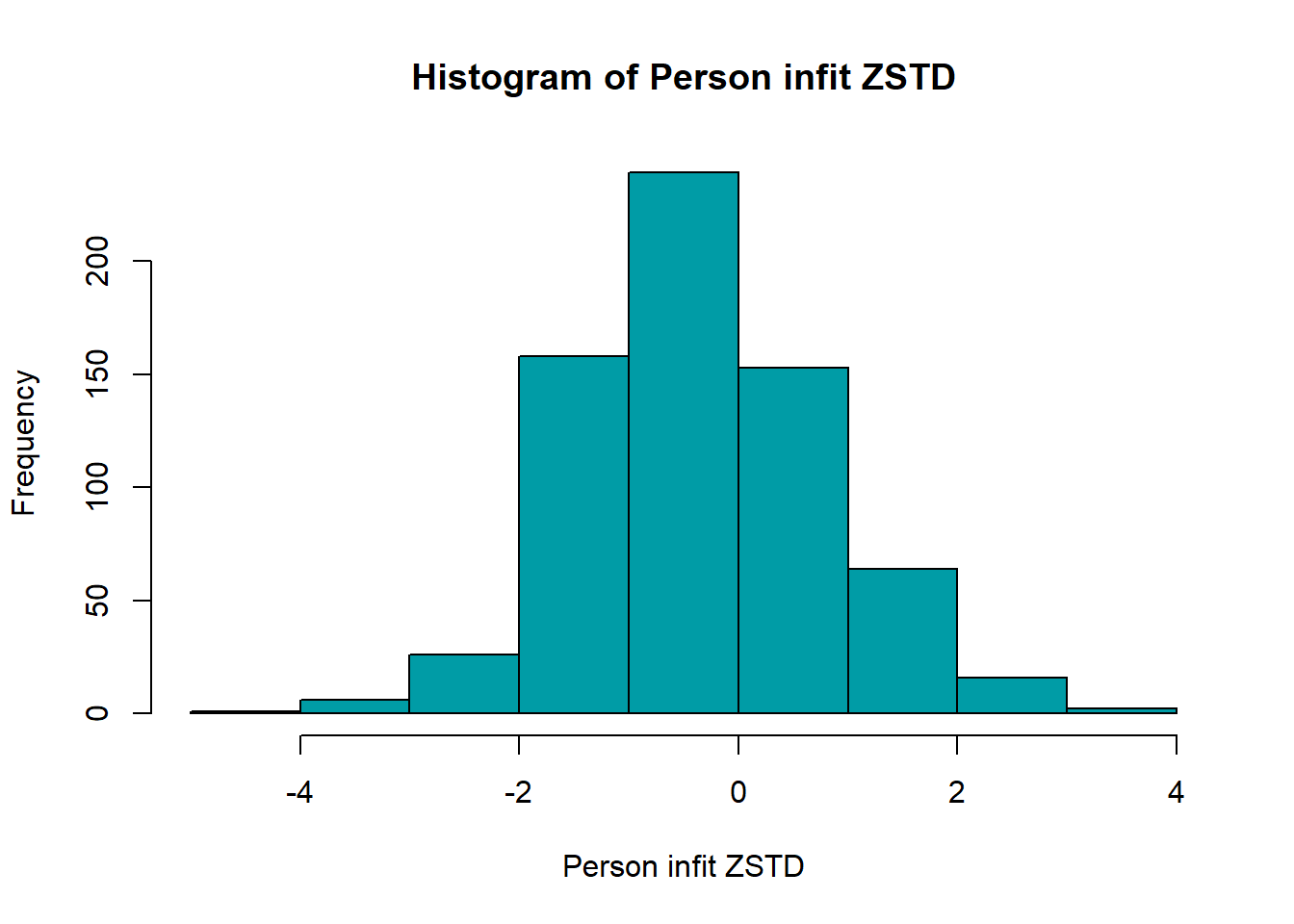

Supplement: Supplementary file 1 — Supplementary Material 1 [file 41598_2025_28073_MOESM1_ESM.zip › Supplementary/analysis_att_files/figure-html/unnamed-chunk-33-1.png]

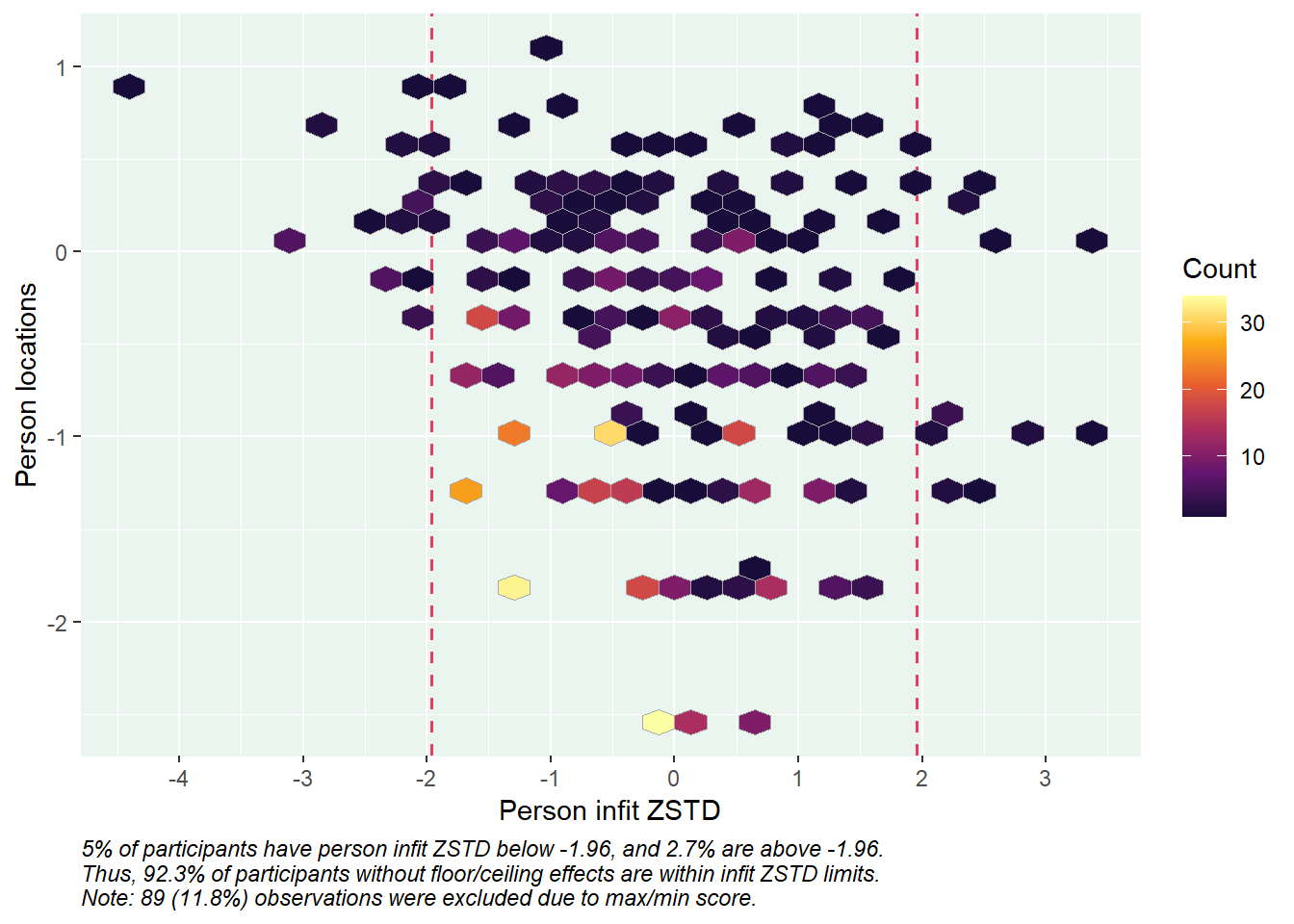

Supplement: Supplementary file 1 — Supplementary Material 1 [file 41598_2025_28073_MOESM1_ESM.zip › Supplementary/analysis_att_files/figure-html/unnamed-chunk-33-2.png]

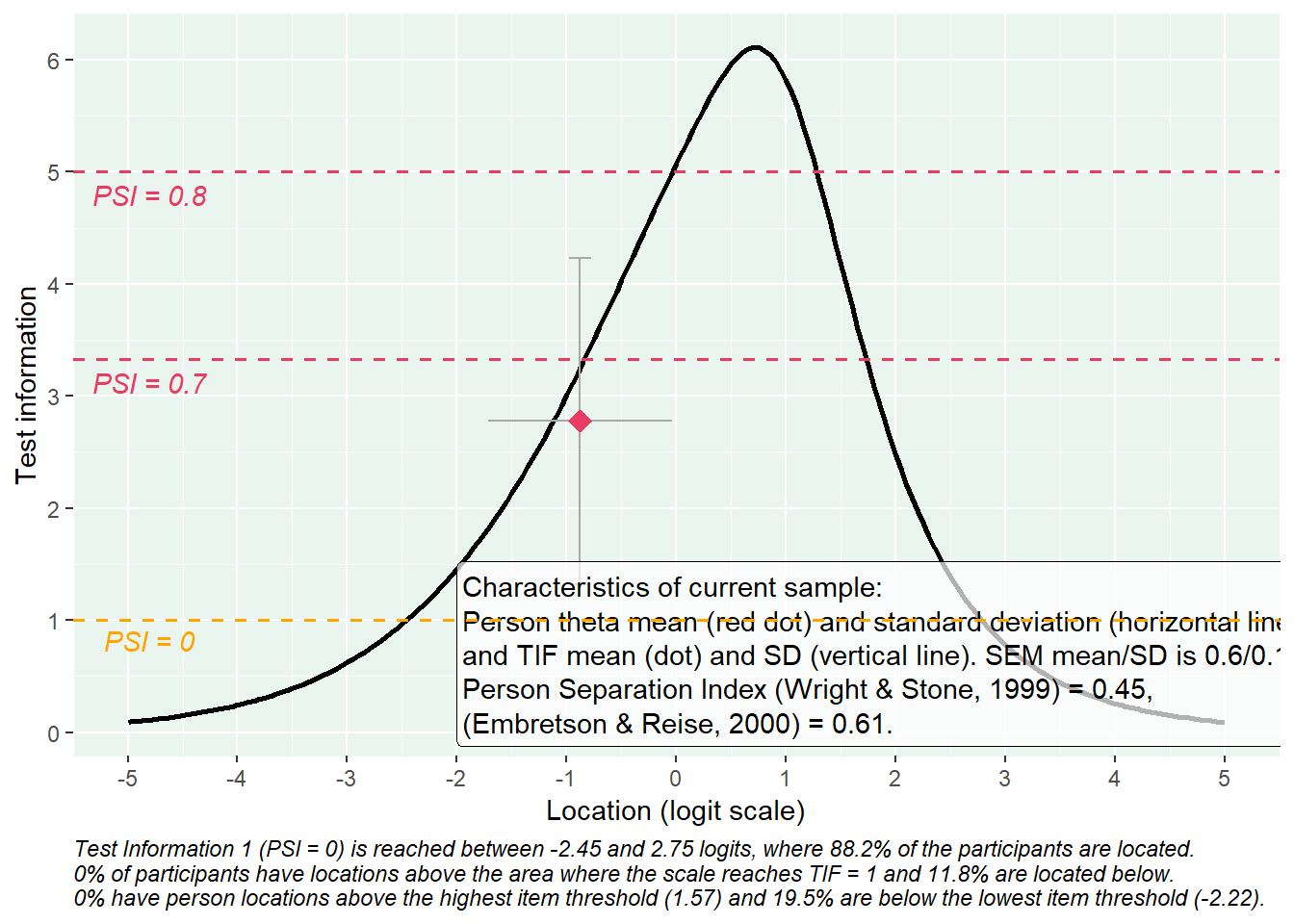

Supplement: Supplementary file 1 — Supplementary Material 1 [file 41598_2025_28073_MOESM1_ESM.zip › Supplementary/analysis_att_files/figure-html/unnamed-chunk-34-1.png]

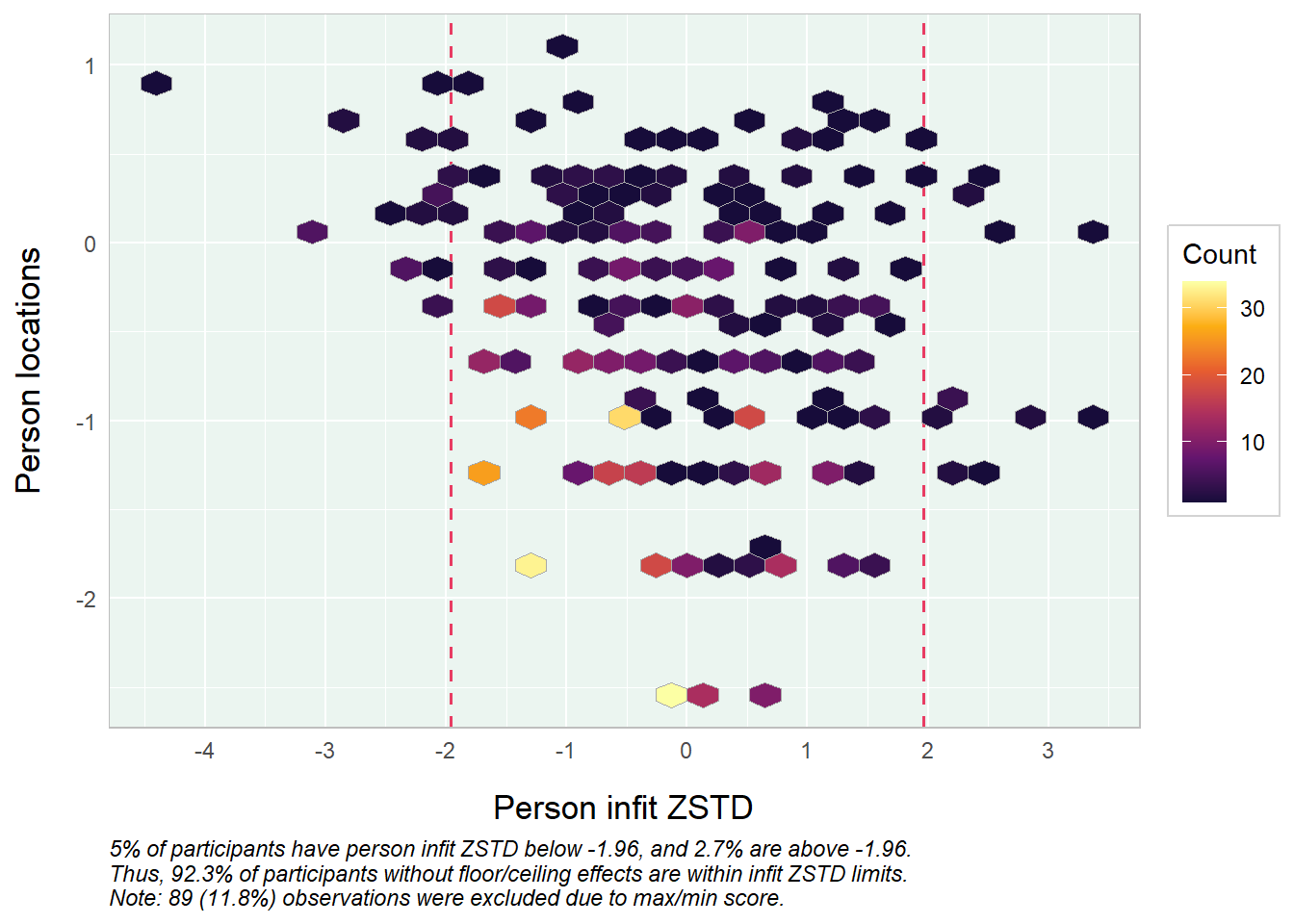

Supplement: Supplementary file 1 — Supplementary Material 1 [file 41598_2025_28073_MOESM1_ESM.zip › Supplementary/analysis_att_files/figure-html/unnamed-chunk-35-2.png]

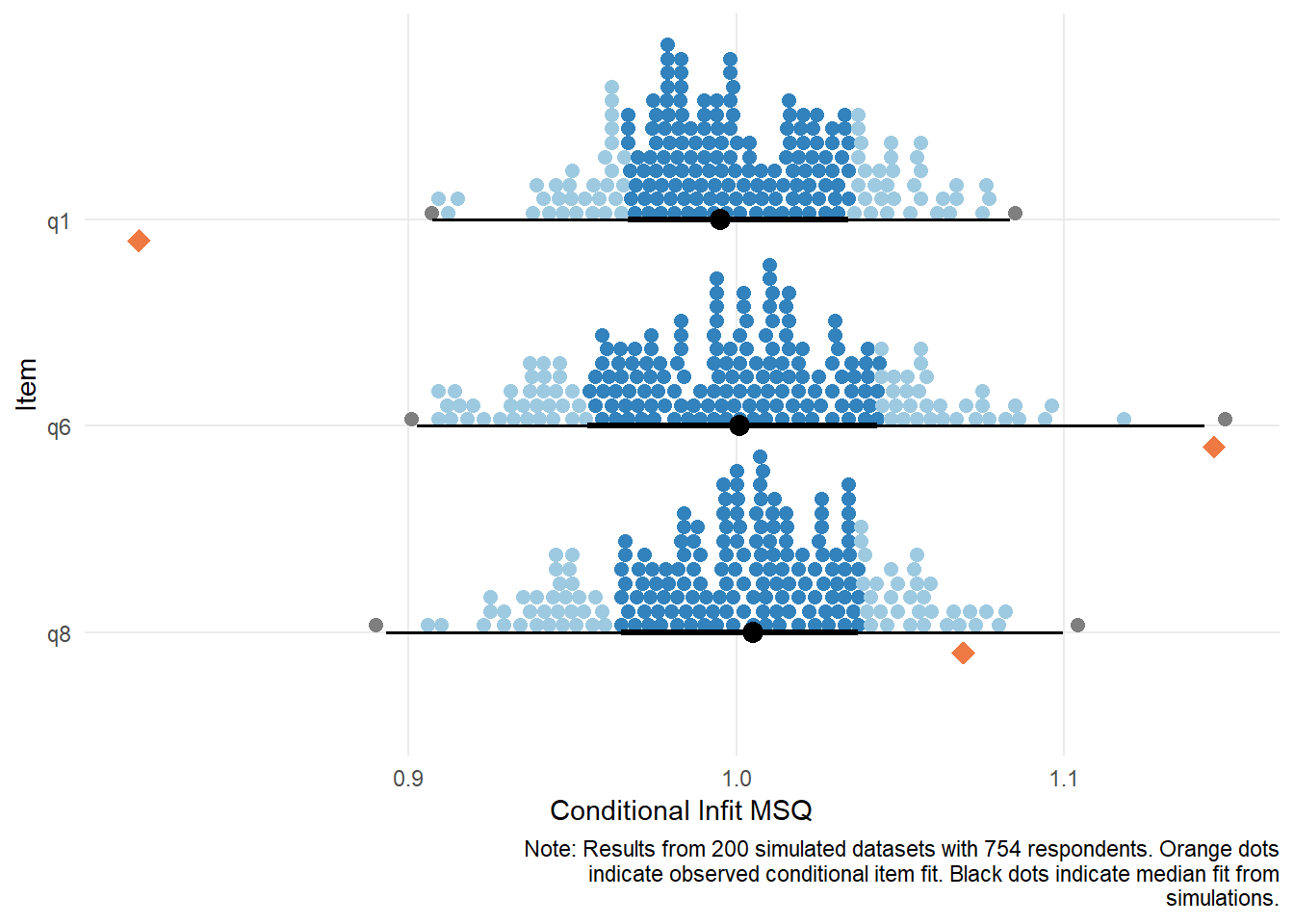

Supplement: Supplementary file 1 — Supplementary Material 1 [file 41598_2025_28073_MOESM1_ESM.zip › Supplementary/analysis_att_files/figure-html/unnamed-chunk-36-1.png]

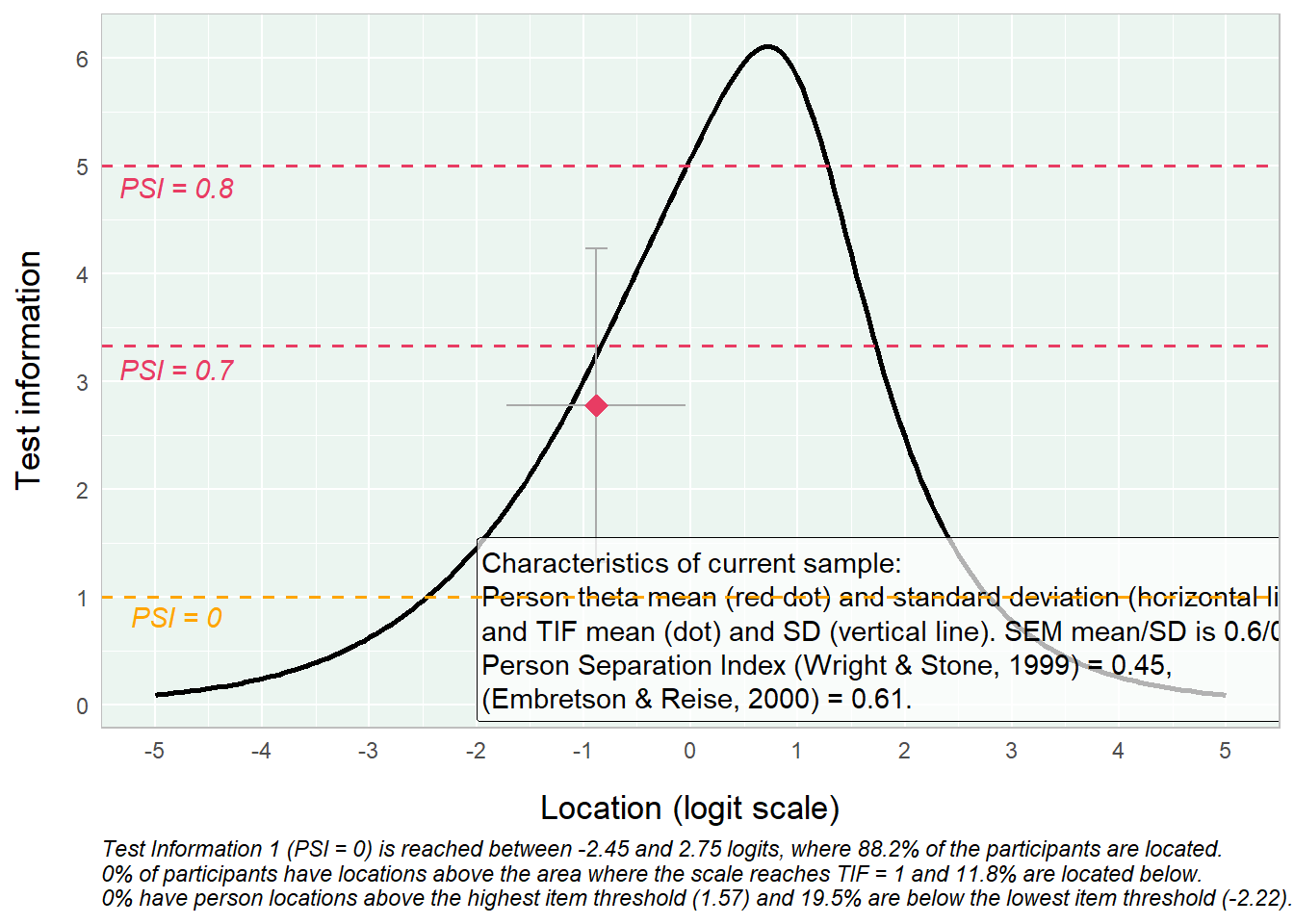

Supplement: Supplementary file 1 — Supplementary Material 1 [file 41598_2025_28073_MOESM1_ESM.zip › Supplementary/analysis_att_files/figure-html/unnamed-chunk-37-1.png]

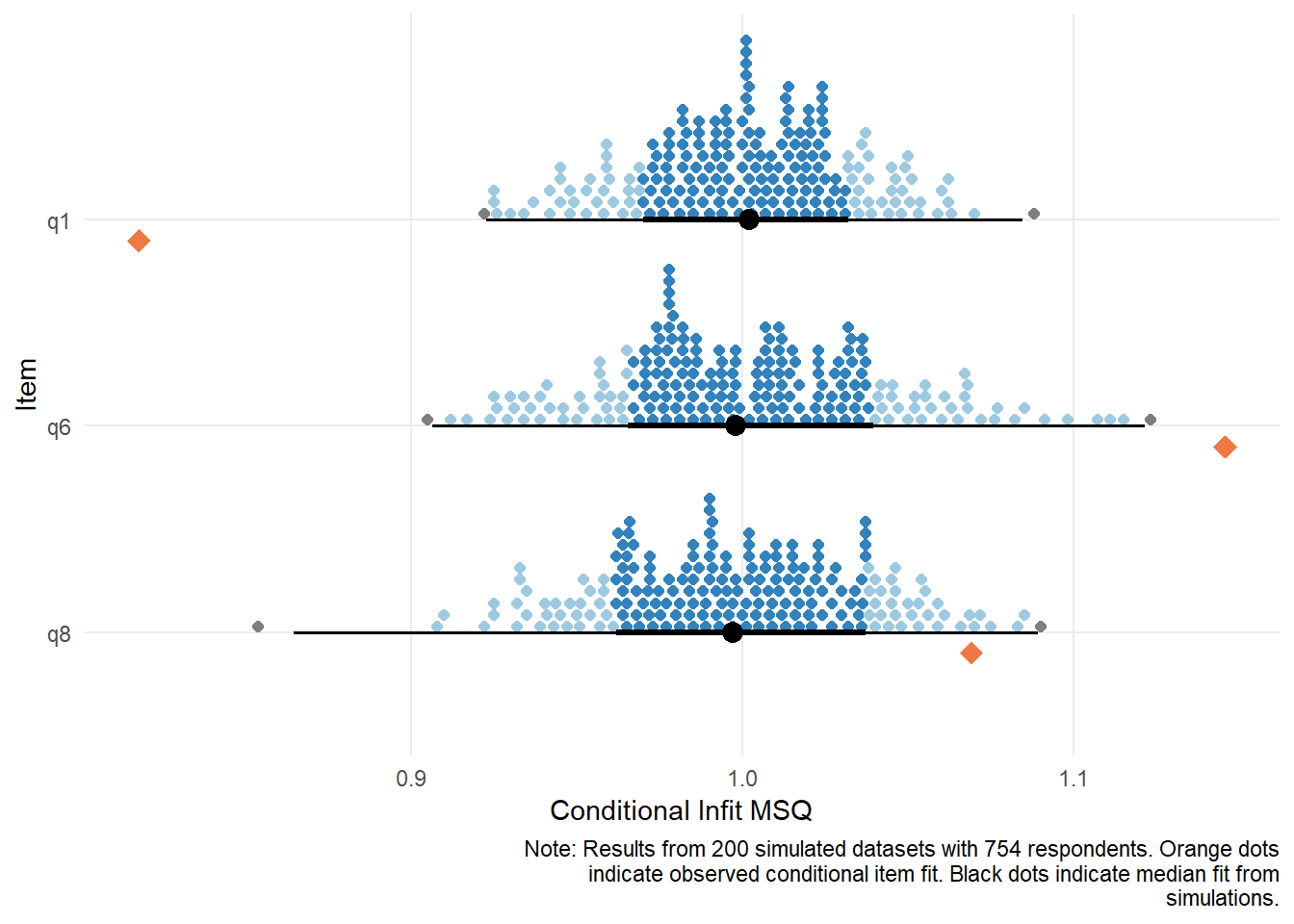

Supplement: Supplementary file 1 — Supplementary Material 1 [file 41598_2025_28073_MOESM1_ESM.zip › Supplementary/analysis_att_files/figure-html/unnamed-chunk-39-1.png]

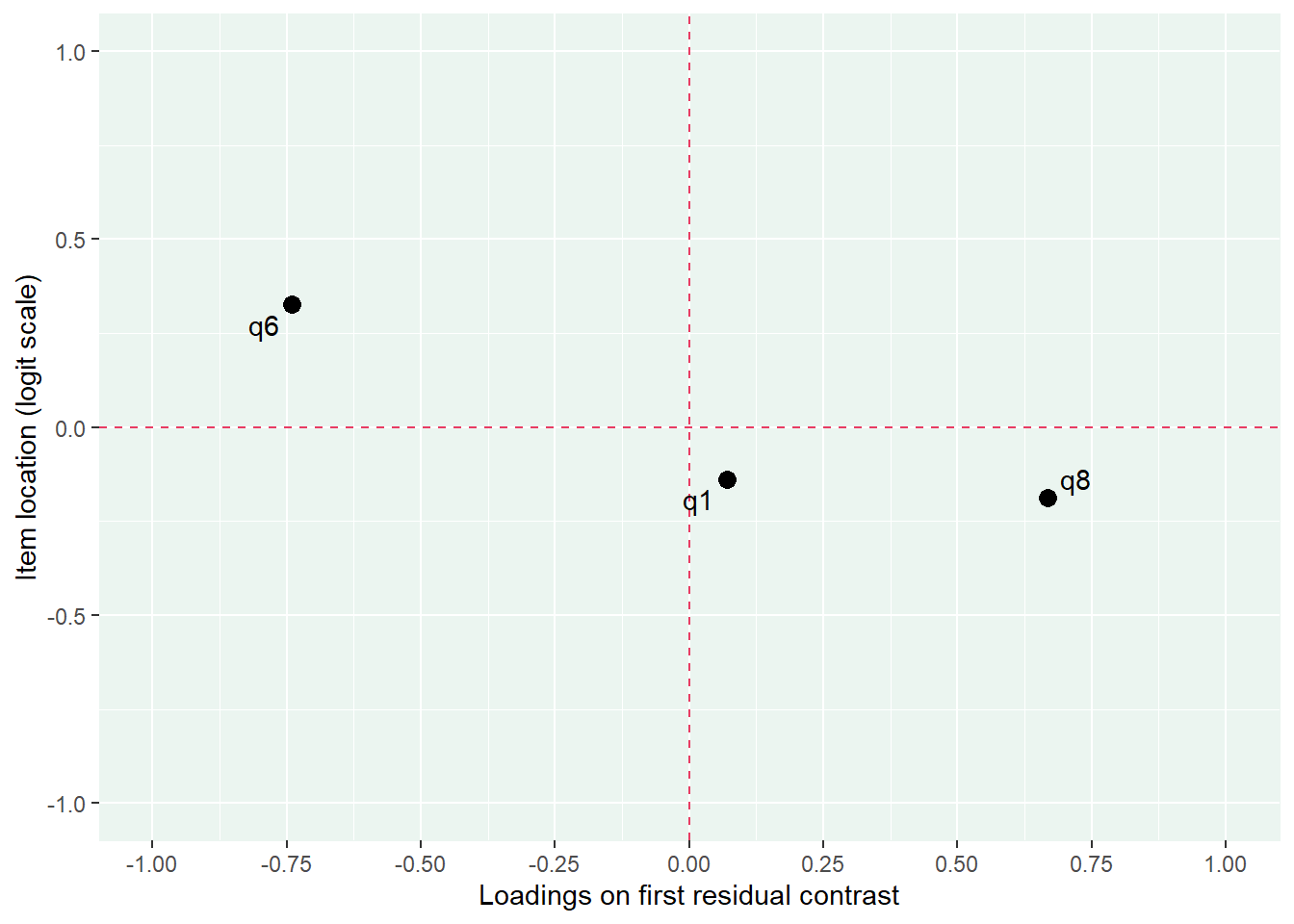

Supplement: Supplementary file 1 — Supplementary Material 1 [file 41598_2025_28073_MOESM1_ESM.zip › Supplementary/analysis_att_files/figure-html/unnamed-chunk-40-1.png]

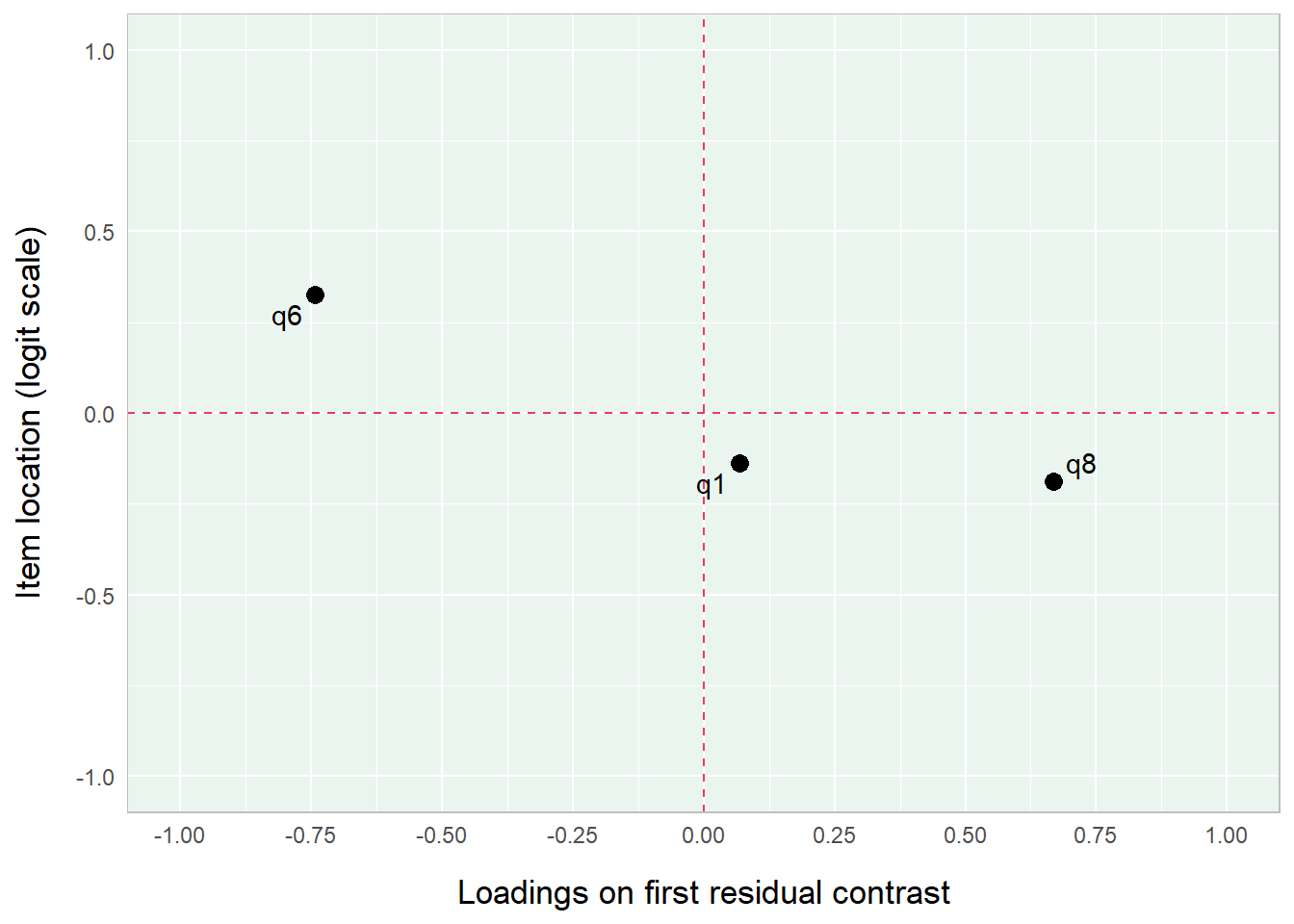

Supplement: Supplementary file 1 — Supplementary Material 1 [file 41598_2025_28073_MOESM1_ESM.zip › Supplementary/analysis_att_files/figure-html/unnamed-chunk-41-1.png]

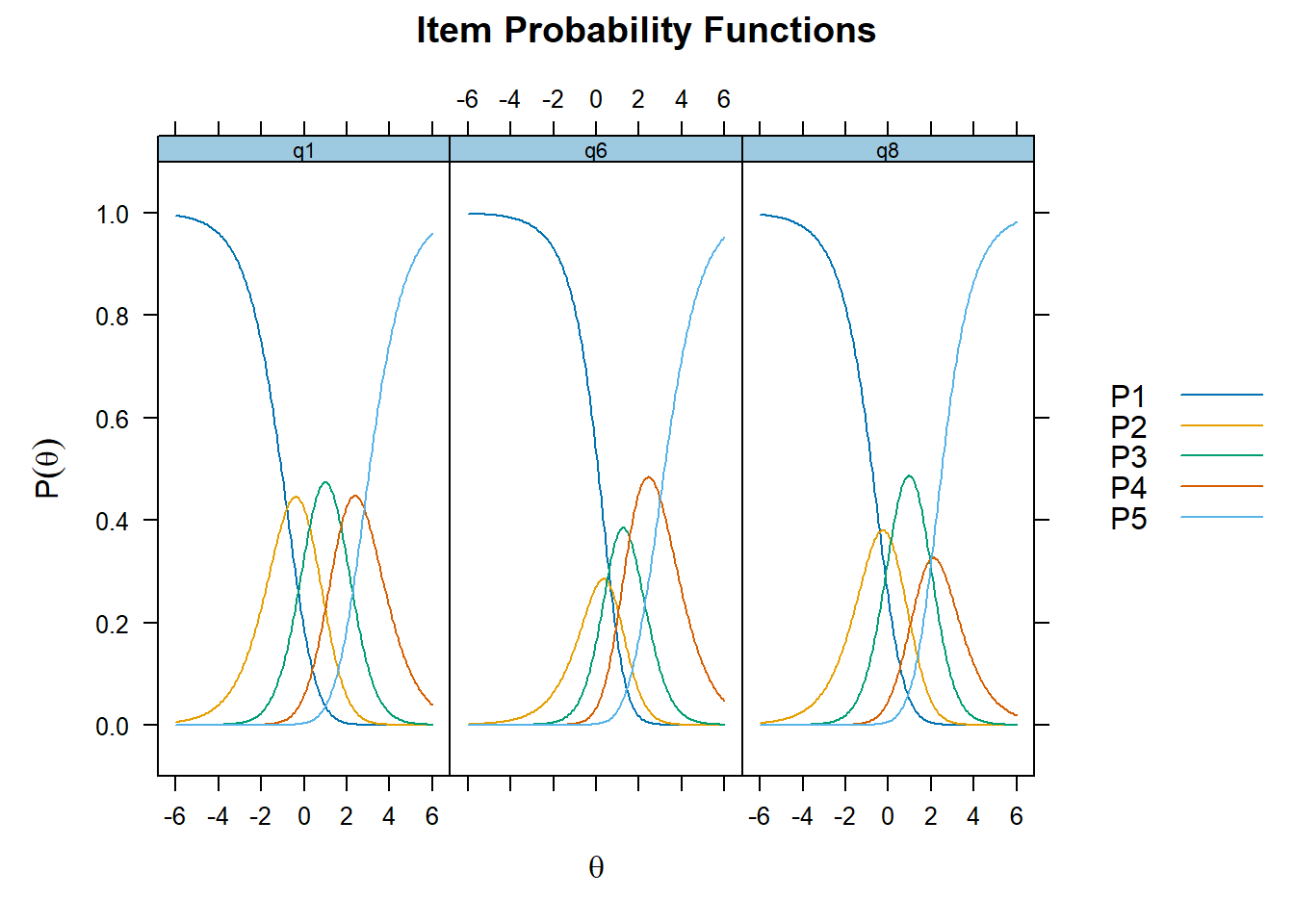

Supplement: Supplementary file 1 — Supplementary Material 1 [file 41598_2025_28073_MOESM1_ESM.zip › Supplementary/analysis_att_files/figure-html/unnamed-chunk-43-1.png]

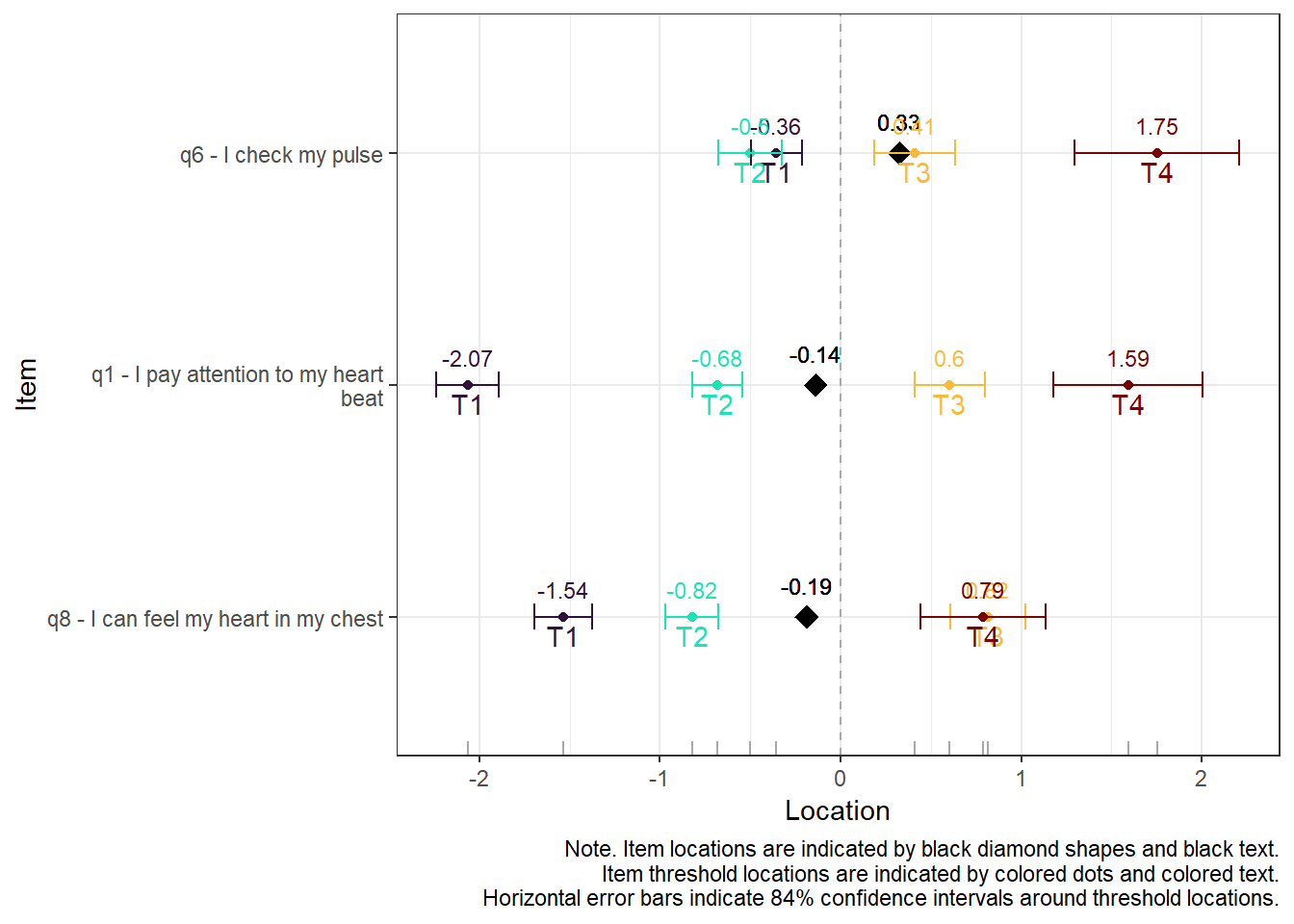

Supplement: Supplementary file 1 — Supplementary Material 1 [file 41598_2025_28073_MOESM1_ESM.zip › Supplementary/analysis_att_files/figure-html/unnamed-chunk-43-2.png]

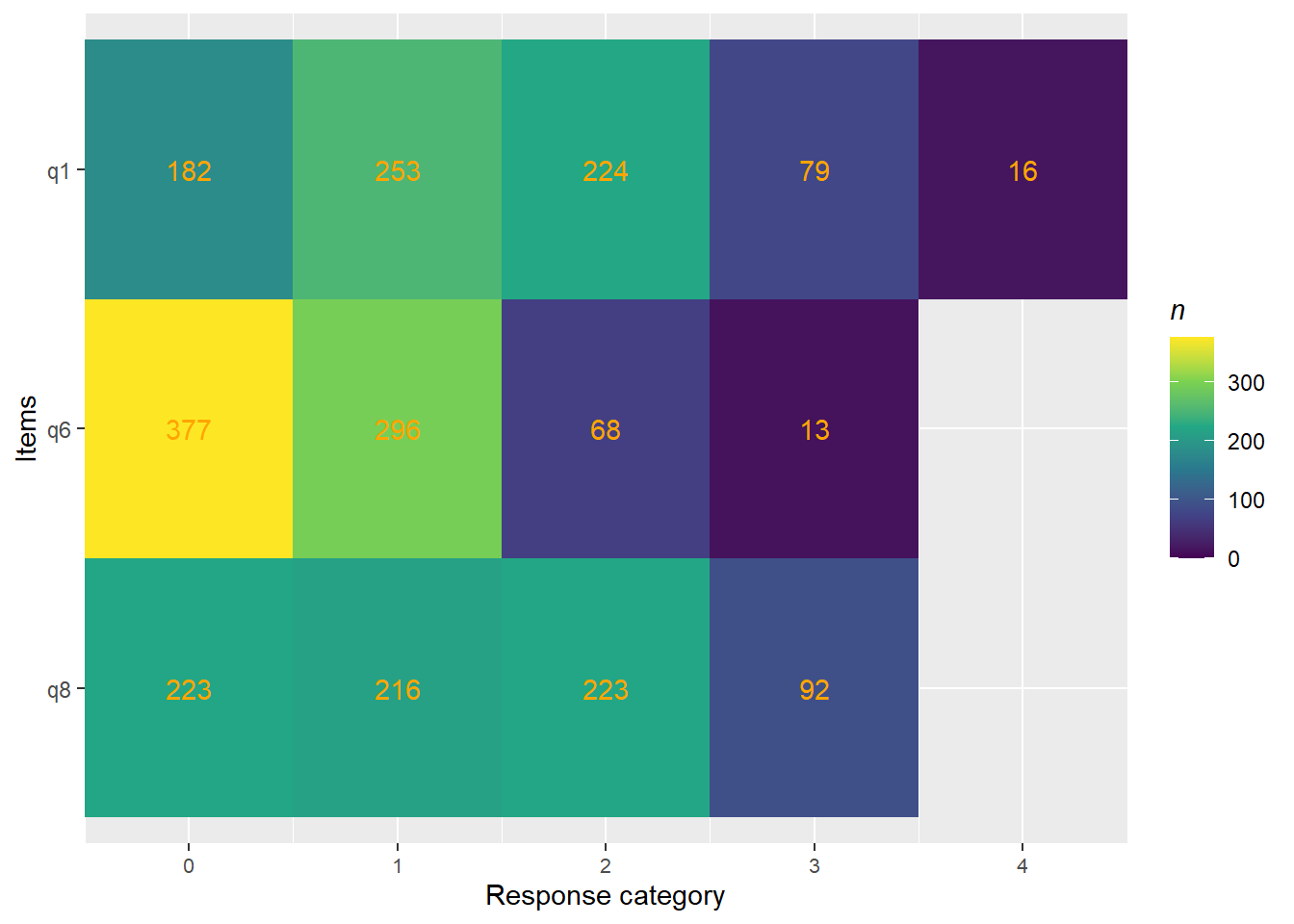

Supplement: Supplementary file 1 — Supplementary Material 1 [file 41598_2025_28073_MOESM1_ESM.zip › Supplementary/analysis_att_files/figure-html/unnamed-chunk-44-1.png]

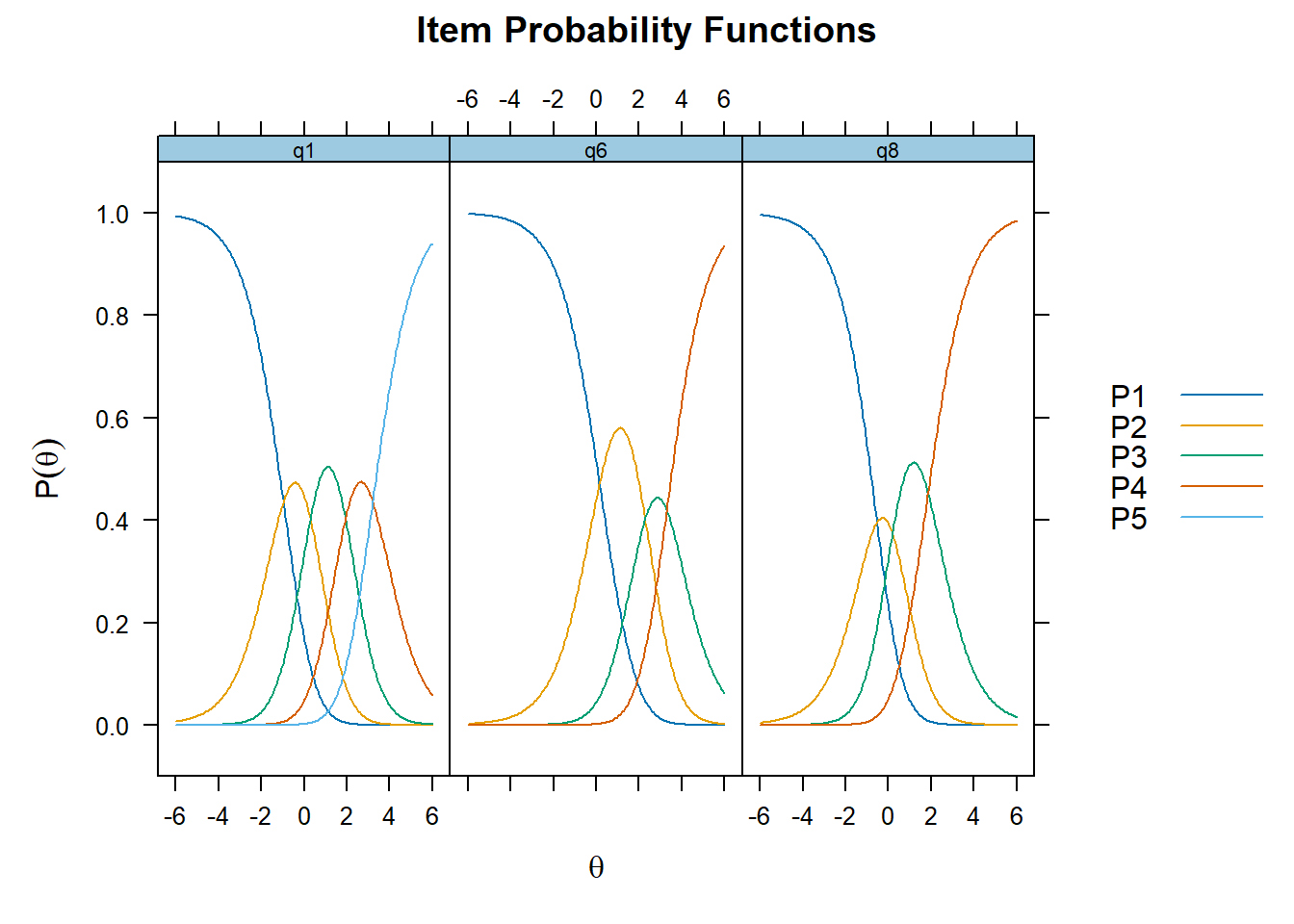

Supplement: Supplementary file 1 — Supplementary Material 1 [file 41598_2025_28073_MOESM1_ESM.zip › Supplementary/analysis_att_files/figure-html/unnamed-chunk-44-2.png]

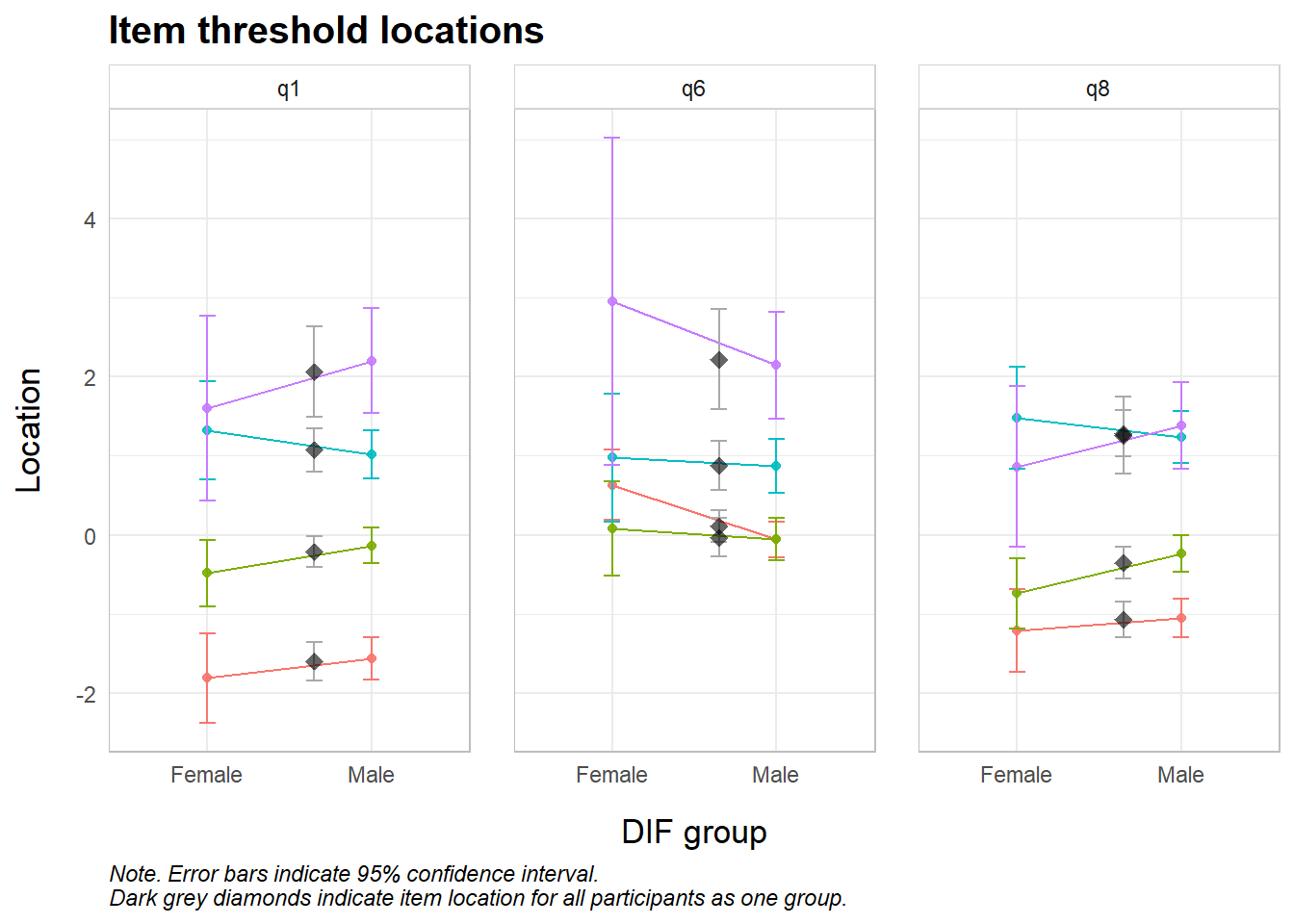

Supplement: Supplementary file 1 — Supplementary Material 1 [file 41598_2025_28073_MOESM1_ESM.zip › Supplementary/analysis_att_files/figure-html/unnamed-chunk-45-1.png]

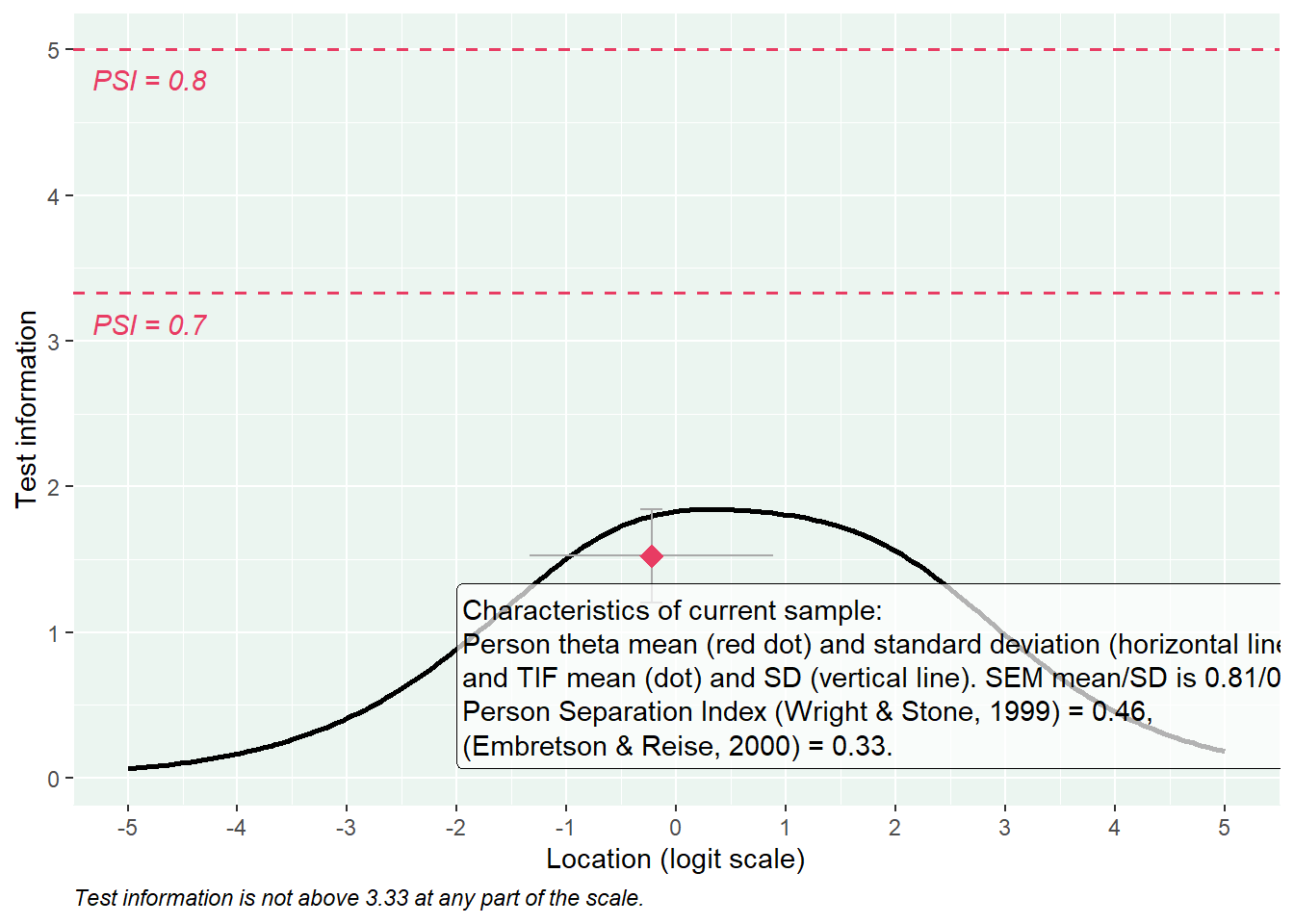

Supplement: Supplementary file 1 — Supplementary Material 1 [file 41598_2025_28073_MOESM1_ESM.zip › Supplementary/analysis_att_files/figure-html/unnamed-chunk-46-1.png]

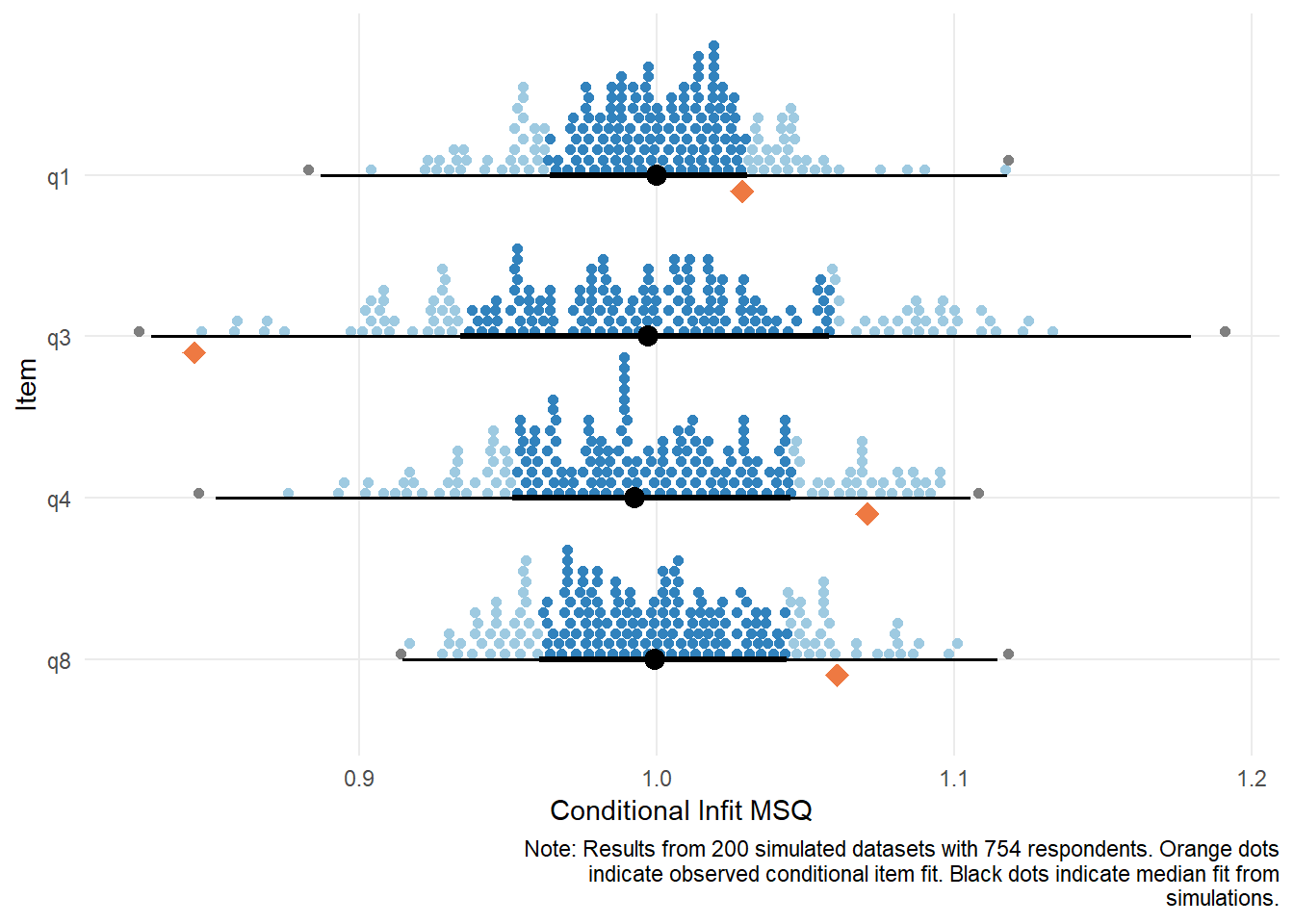

Supplement: Supplementary file 1 — Supplementary Material 1 [file 41598_2025_28073_MOESM1_ESM.zip › Supplementary/analysis_att_files/figure-html/unnamed-chunk-48-1.png]

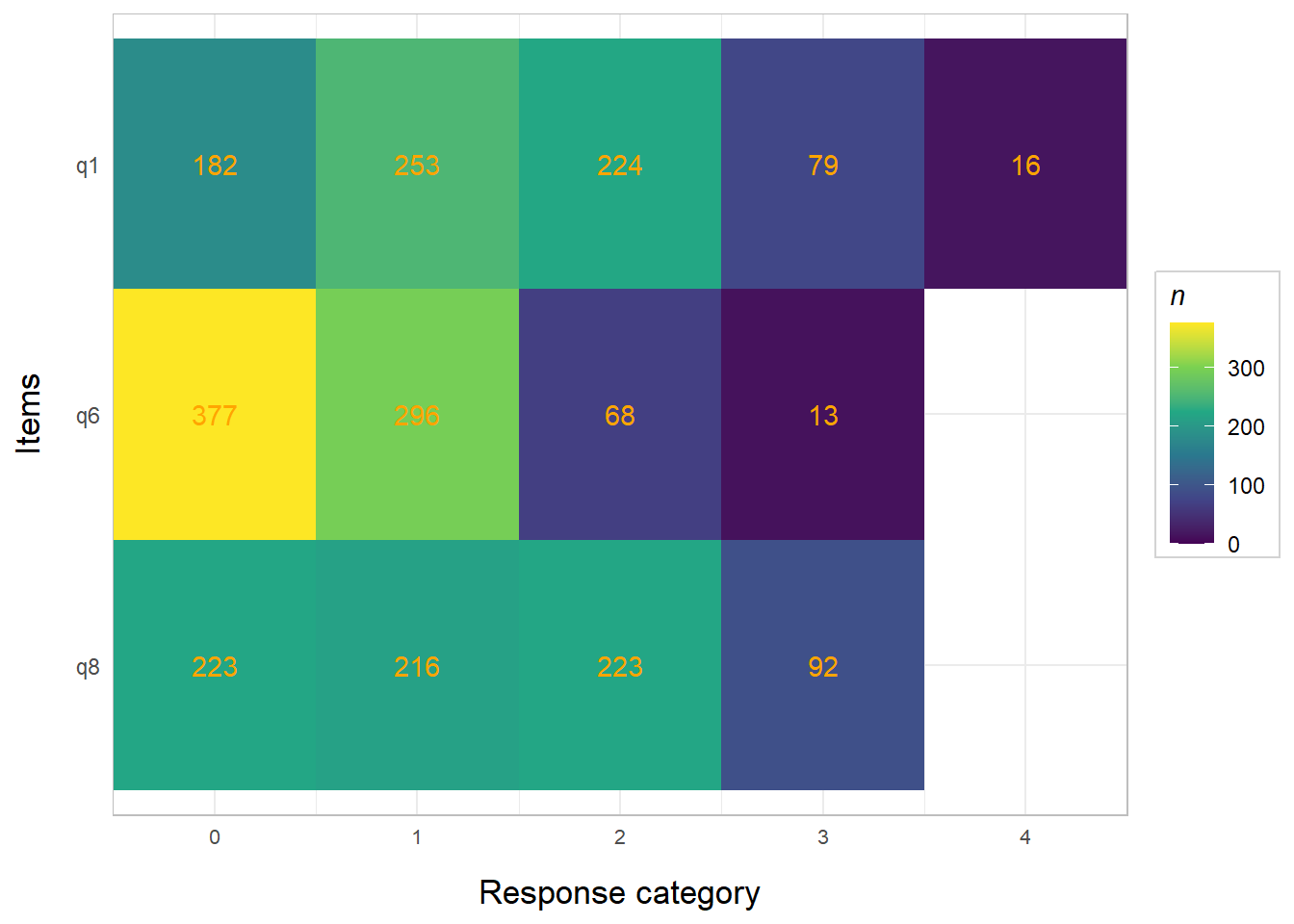

Supplement: Supplementary file 1 — Supplementary Material 1 [file 41598_2025_28073_MOESM1_ESM.zip › Supplementary/analysis_att_files/figure-html/unnamed-chunk-49-1.png]

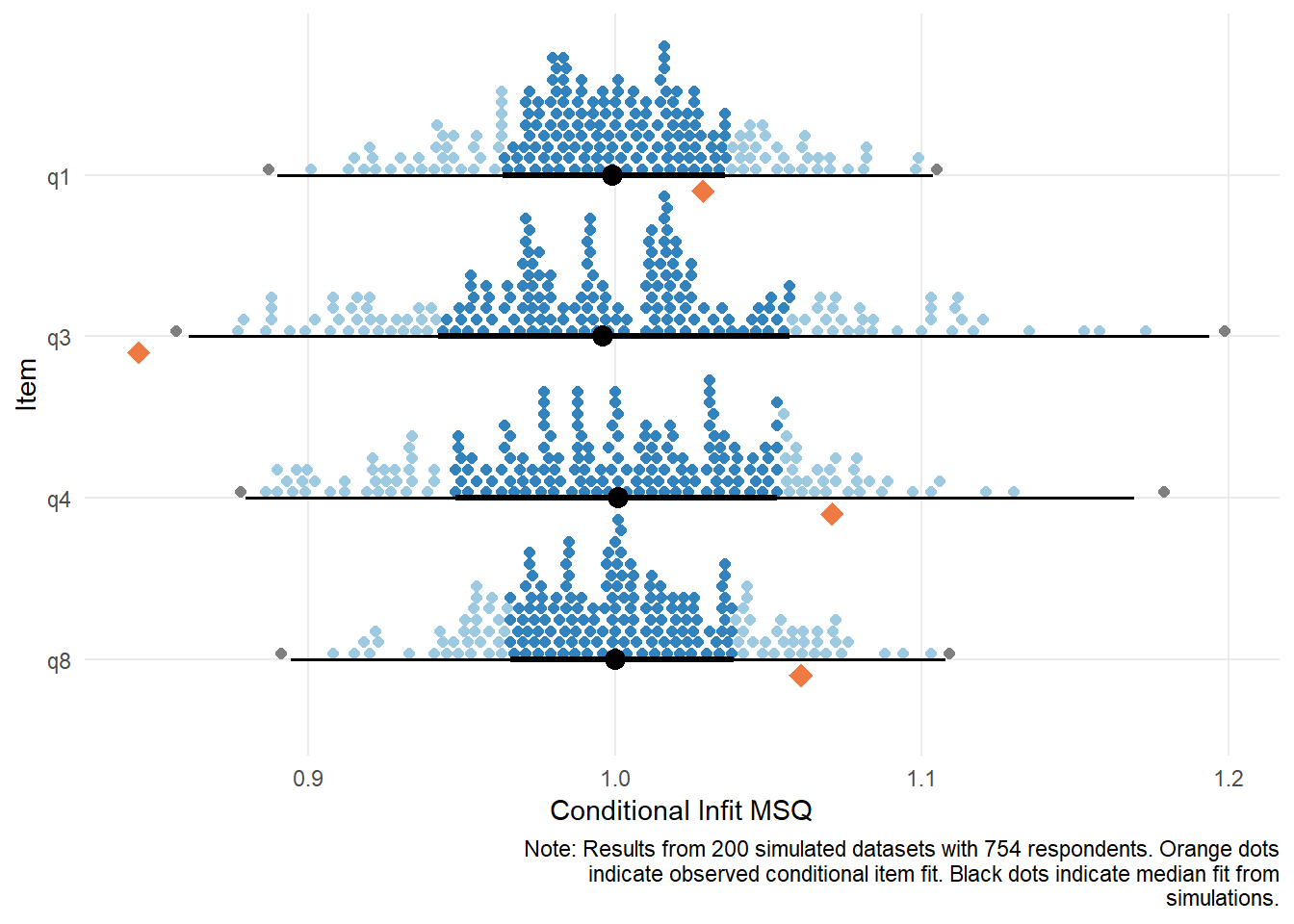

Supplement: Supplementary file 1 — Supplementary Material 1 [file 41598_2025_28073_MOESM1_ESM.zip › Supplementary/analysis_att_files/figure-html/unnamed-chunk-51-1.png]

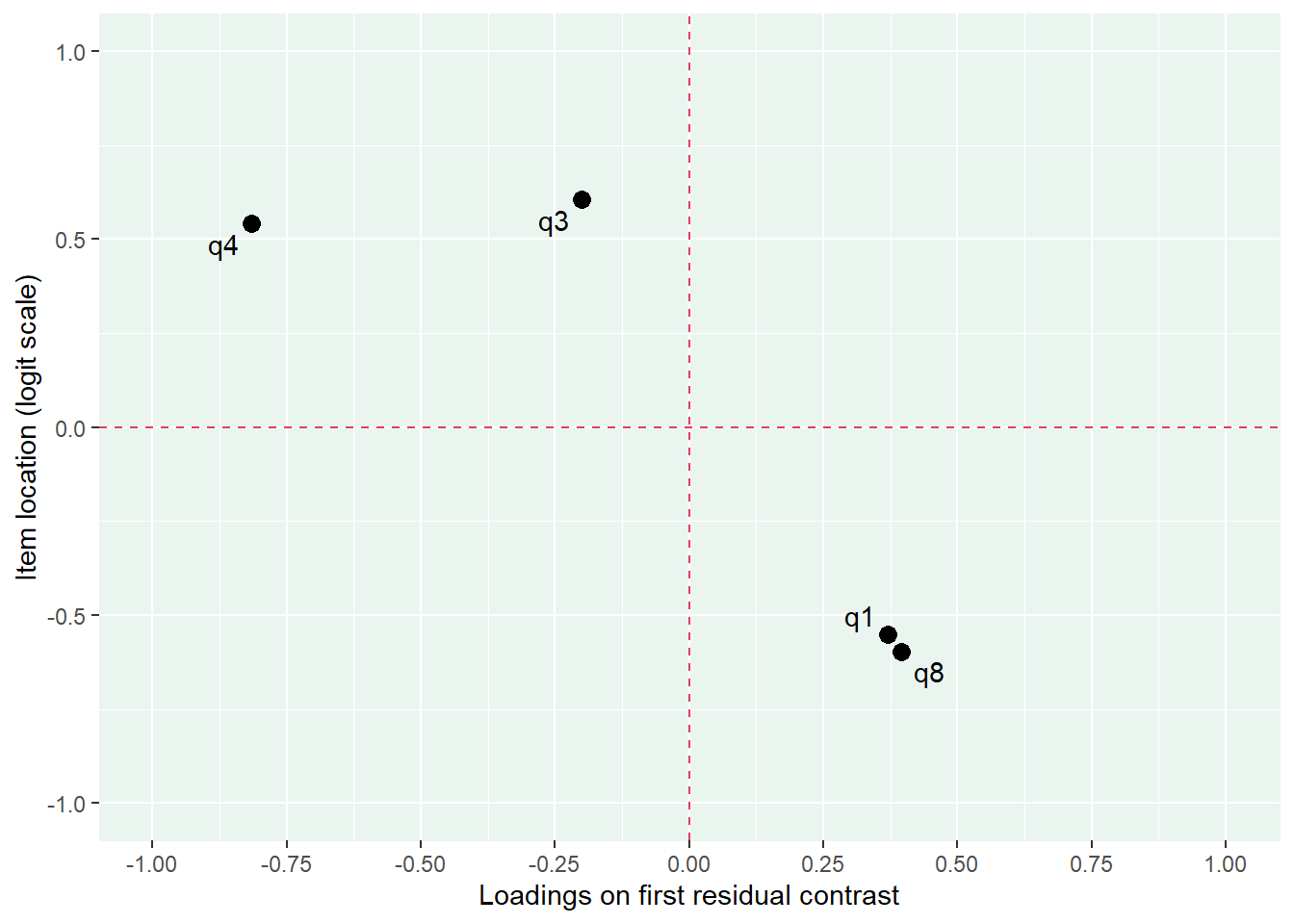

Supplement: Supplementary file 1 — Supplementary Material 1 [file 41598_2025_28073_MOESM1_ESM.zip › Supplementary/analysis_att_files/figure-html/unnamed-chunk-52-1.png]

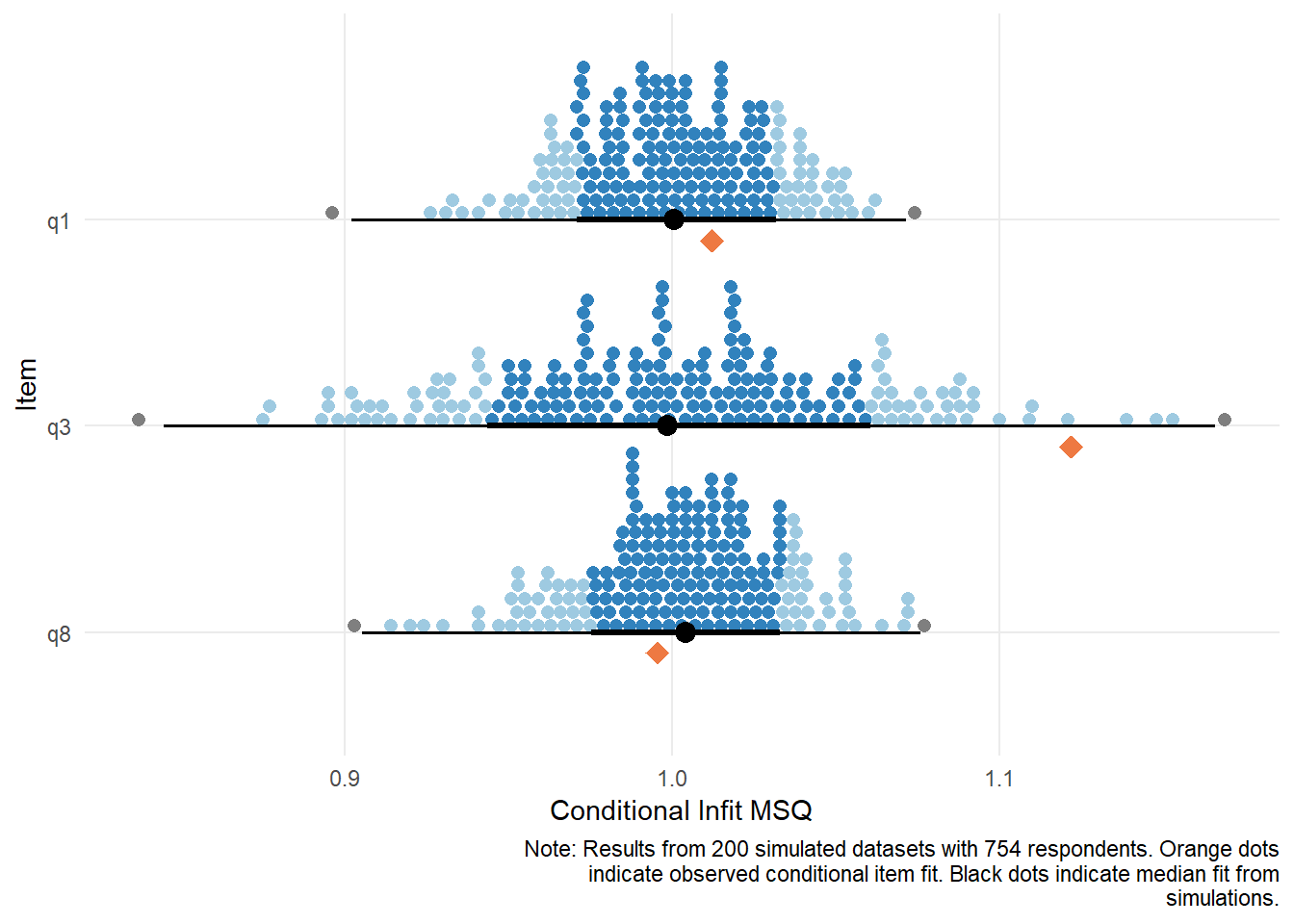

Supplement: Supplementary file 1 — Supplementary Material 1 [file 41598_2025_28073_MOESM1_ESM.zip › Supplementary/analysis_att_files/figure-html/unnamed-chunk-55-1.png]

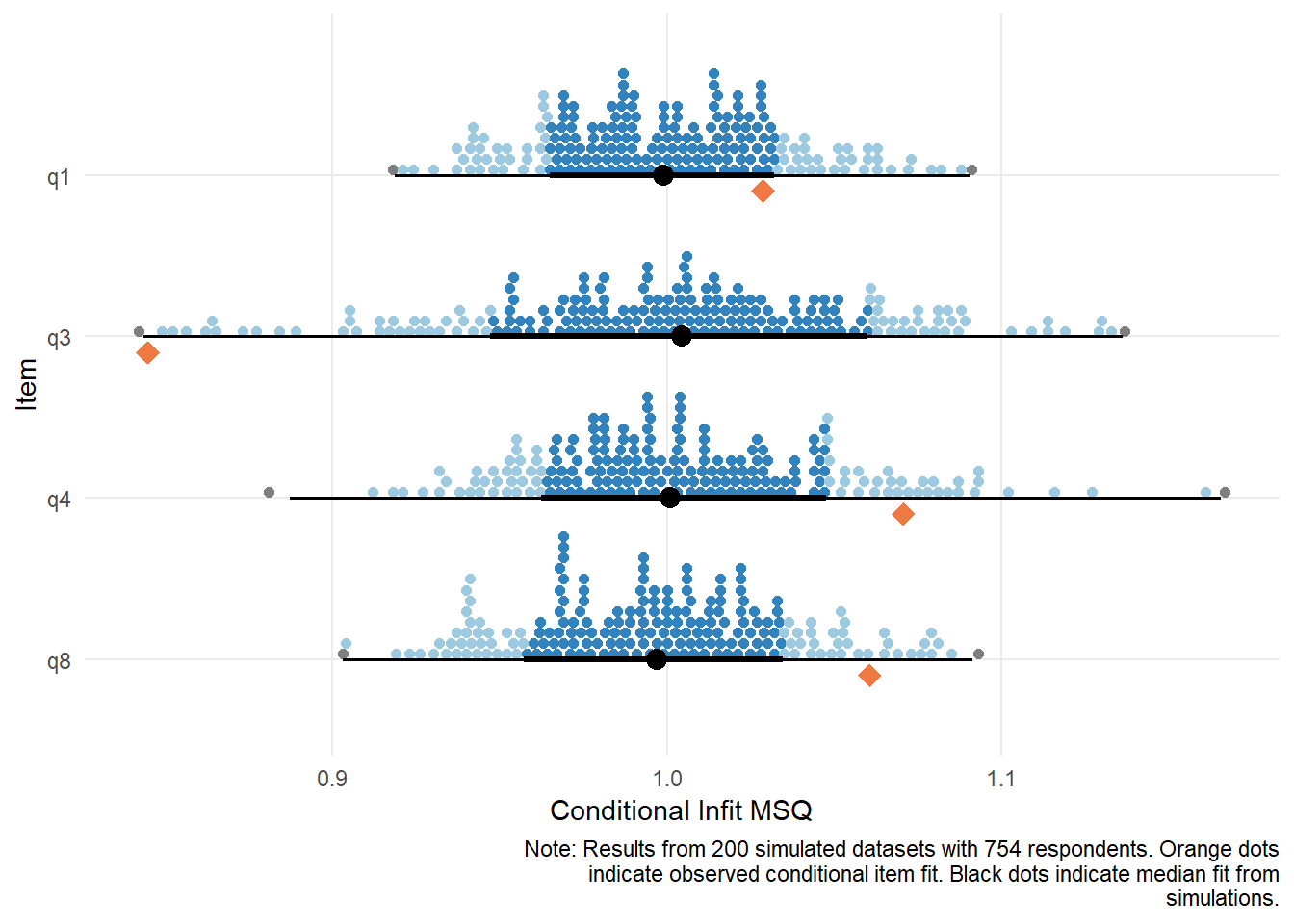

Supplement: Supplementary file 1 — Supplementary Material 1 [file 41598_2025_28073_MOESM1_ESM.zip › Supplementary/analysis_att_files/figure-html/unnamed-chunk-57-1.png]

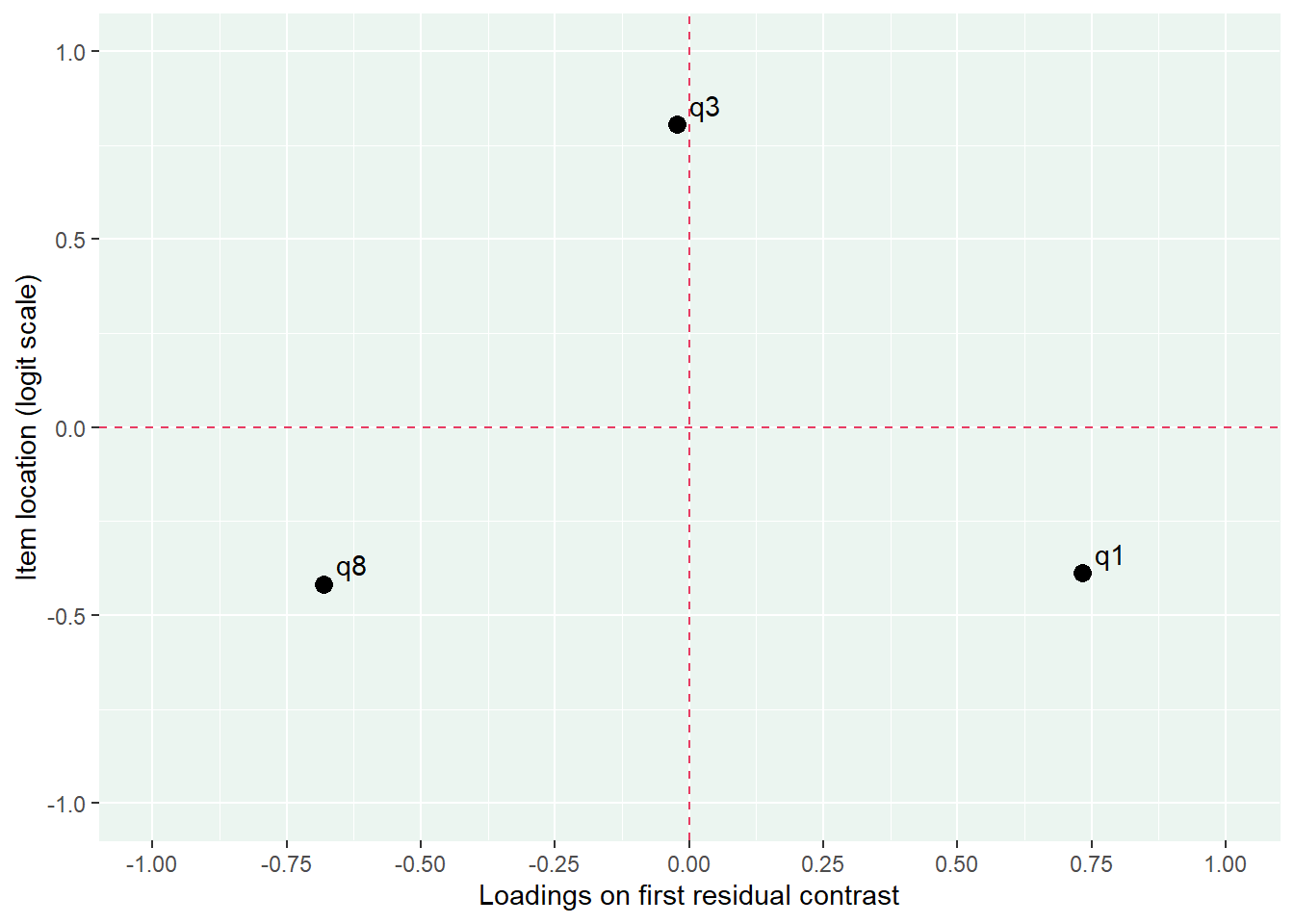

Supplement: Supplementary file 1 — Supplementary Material 1 [file 41598_2025_28073_MOESM1_ESM.zip › Supplementary/analysis_att_files/figure-html/unnamed-chunk-59-1.png]

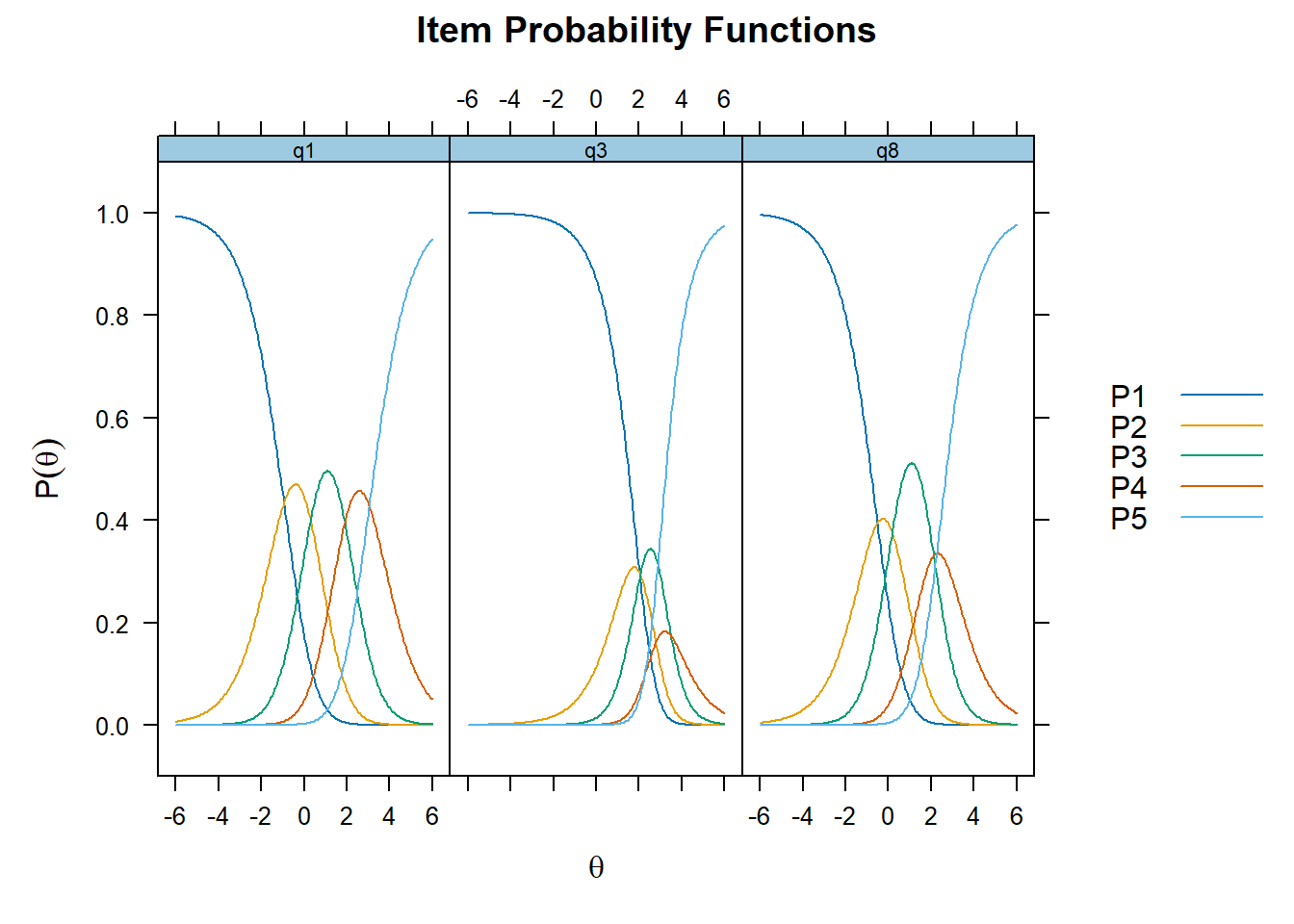

Supplement: Supplementary file 1 — Supplementary Material 1 [file 41598_2025_28073_MOESM1_ESM.zip › Supplementary/analysis_att_files/figure-html/unnamed-chunk-61-1.png]

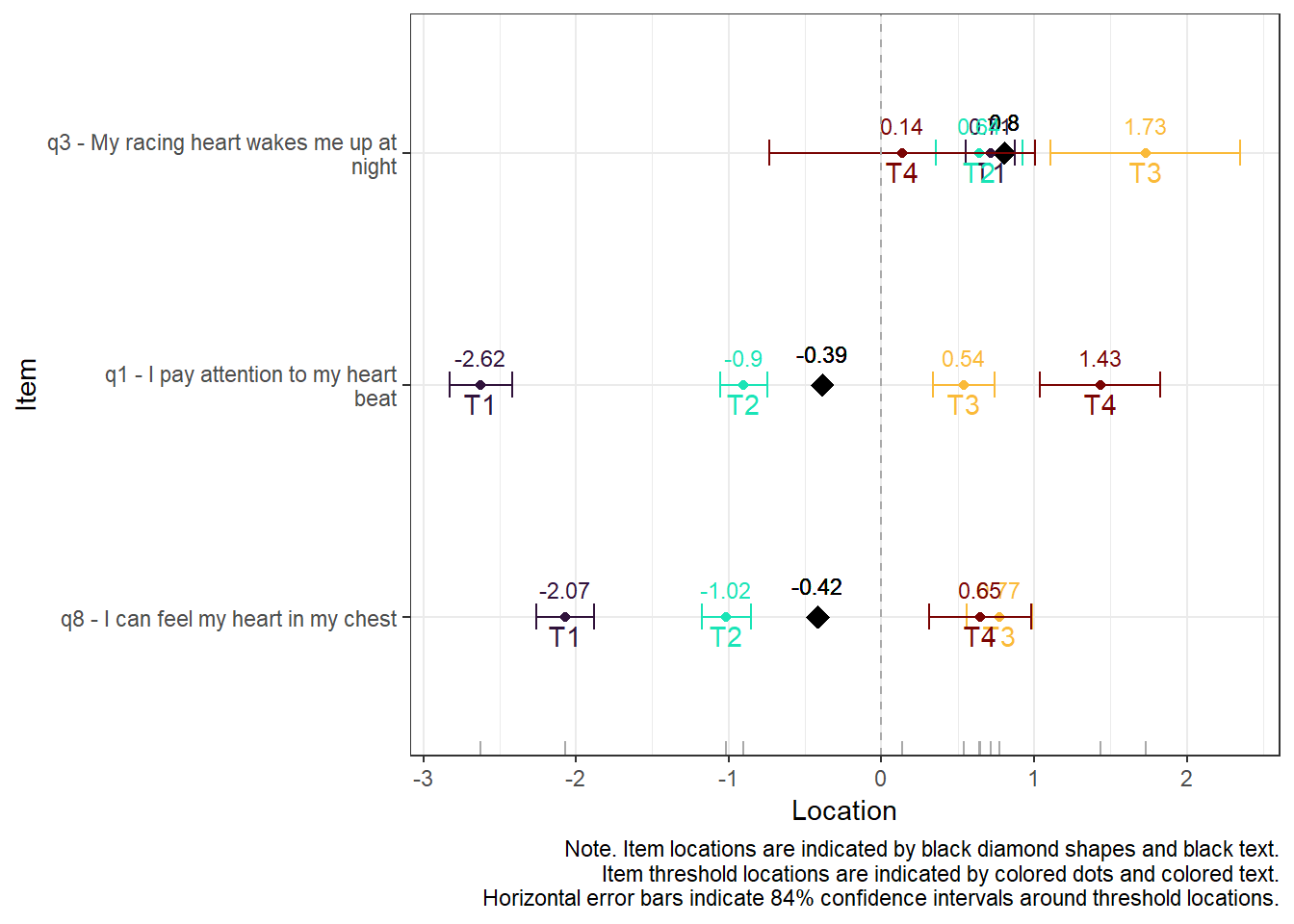

Supplement: Supplementary file 1 — Supplementary Material 1 [file 41598_2025_28073_MOESM1_ESM.zip › Supplementary/analysis_att_files/figure-html/unnamed-chunk-61-2.png]

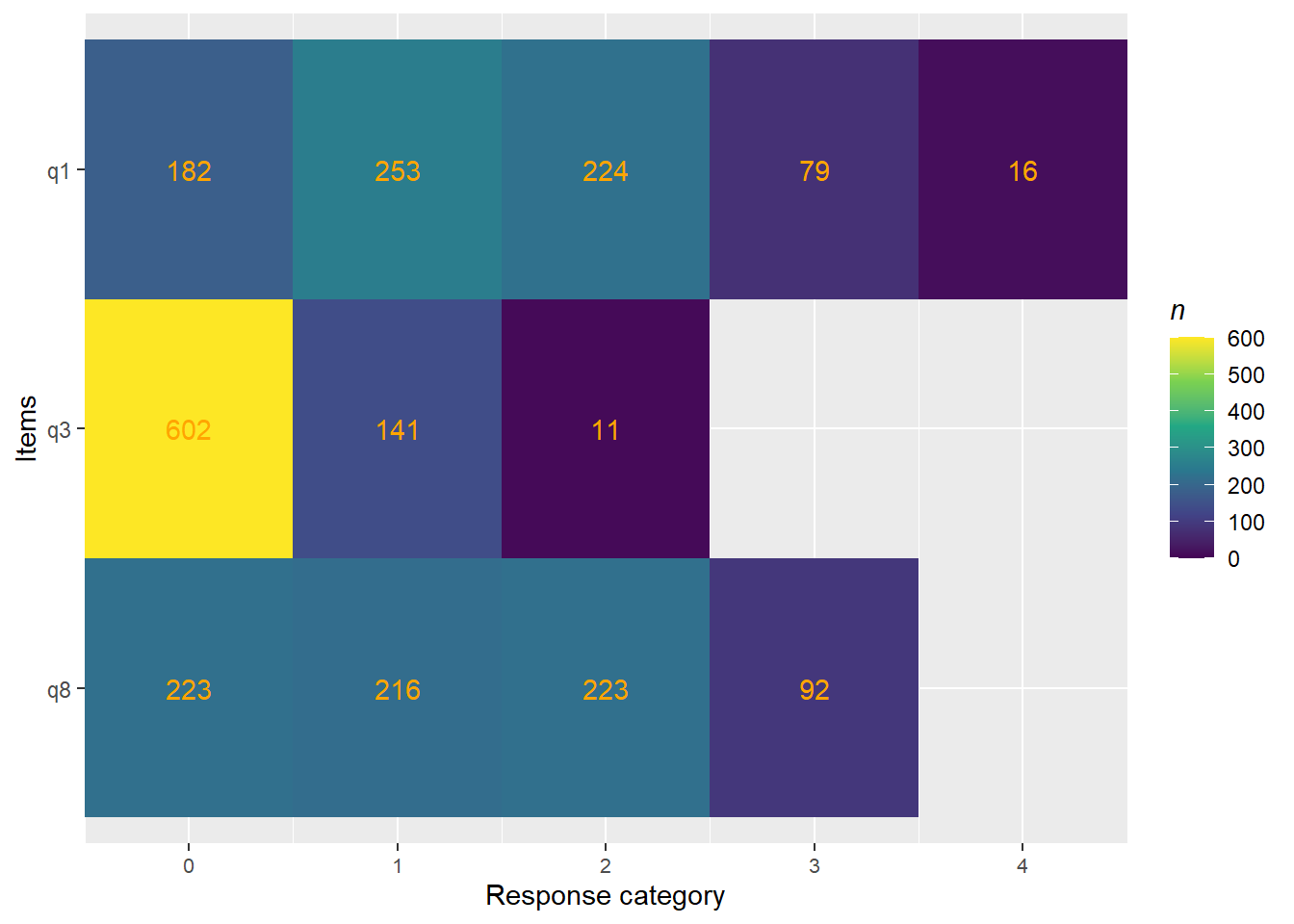

Supplement: Supplementary file 1 — Supplementary Material 1 [file 41598_2025_28073_MOESM1_ESM.zip › Supplementary/analysis_att_files/figure-html/unnamed-chunk-62-1.png]

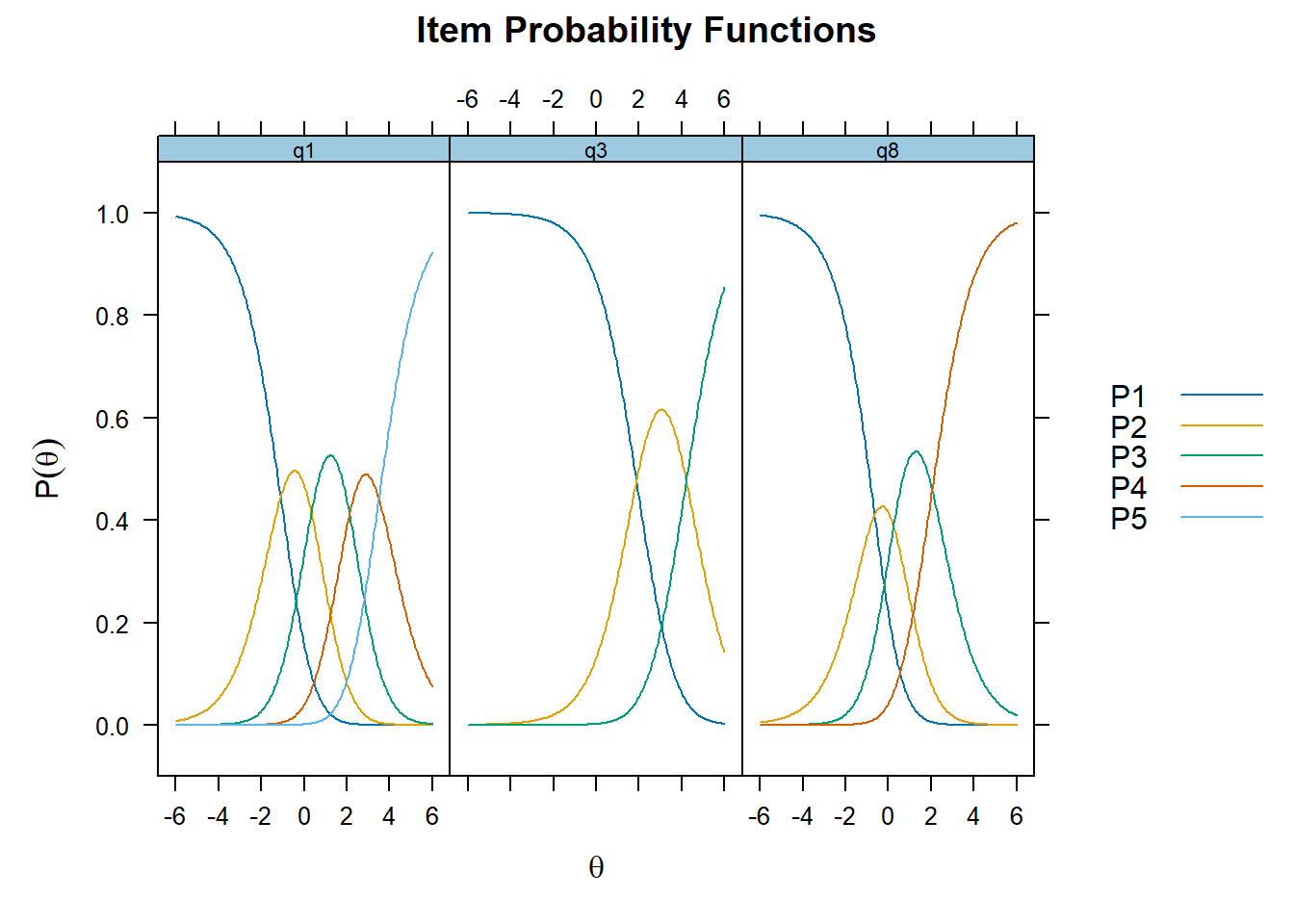

Supplement: Supplementary file 1 — Supplementary Material 1 [file 41598_2025_28073_MOESM1_ESM.zip › Supplementary/analysis_att_files/figure-html/unnamed-chunk-63-1.png]

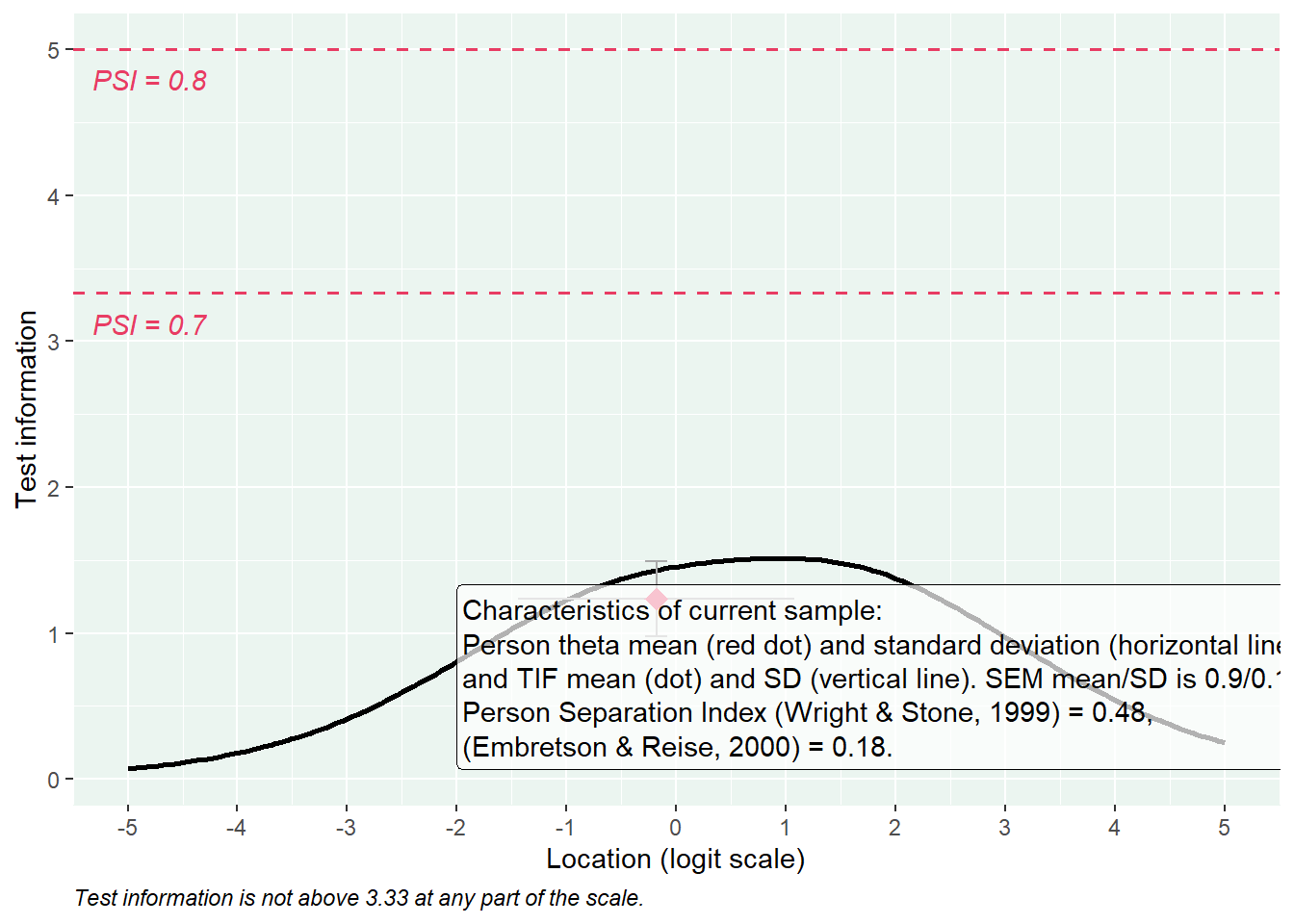

Supplement: Supplementary file 1 — Supplementary Material 1 [file 41598_2025_28073_MOESM1_ESM.zip › Supplementary/analysis_att_files/figure-html/unnamed-chunk-65-1.png]

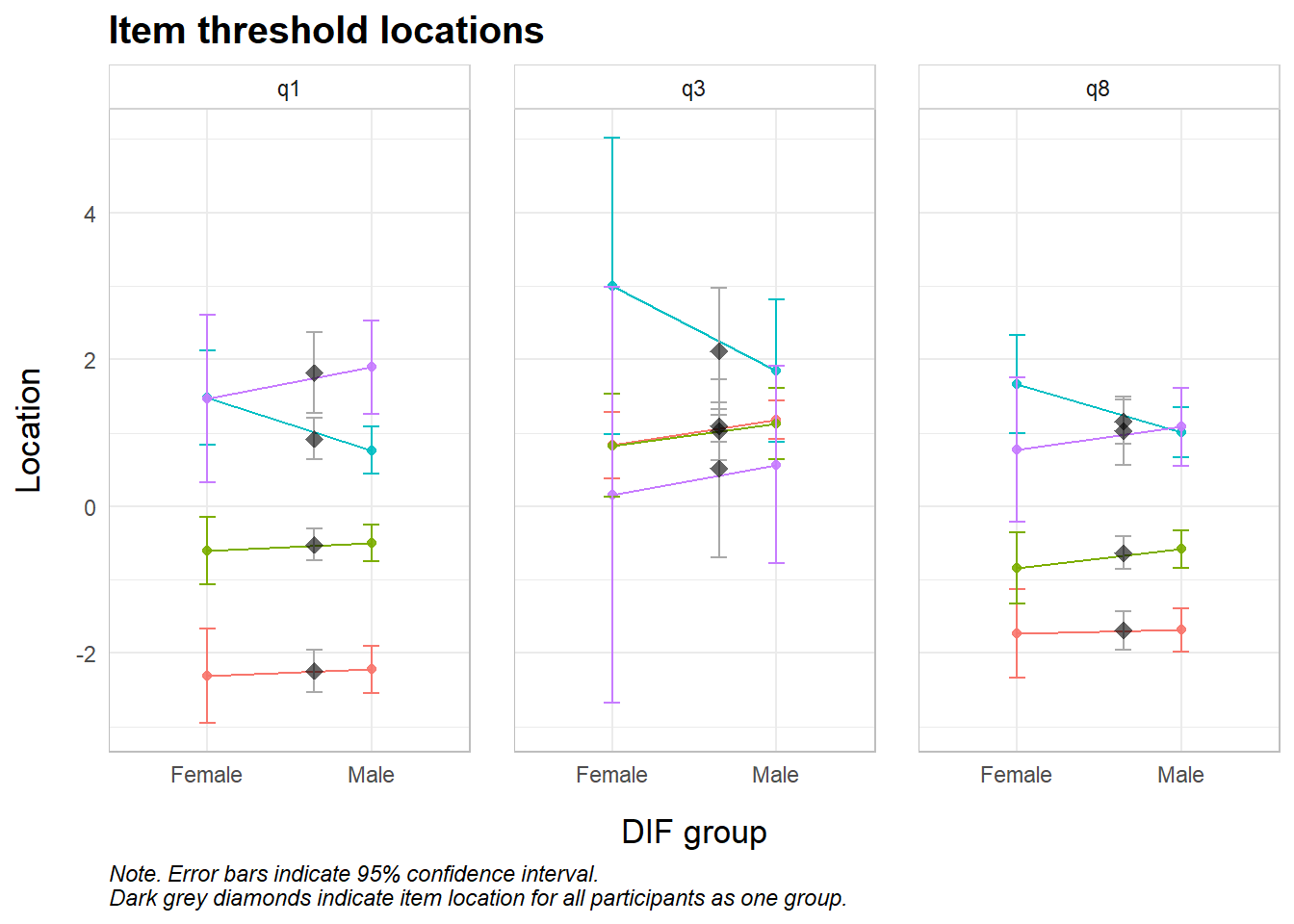

Supplement: Supplementary file 1 — Supplementary Material 1 [file 41598_2025_28073_MOESM1_ESM.zip › Supplementary/analysis_att_files/figure-html/unnamed-chunk-67-1.png]

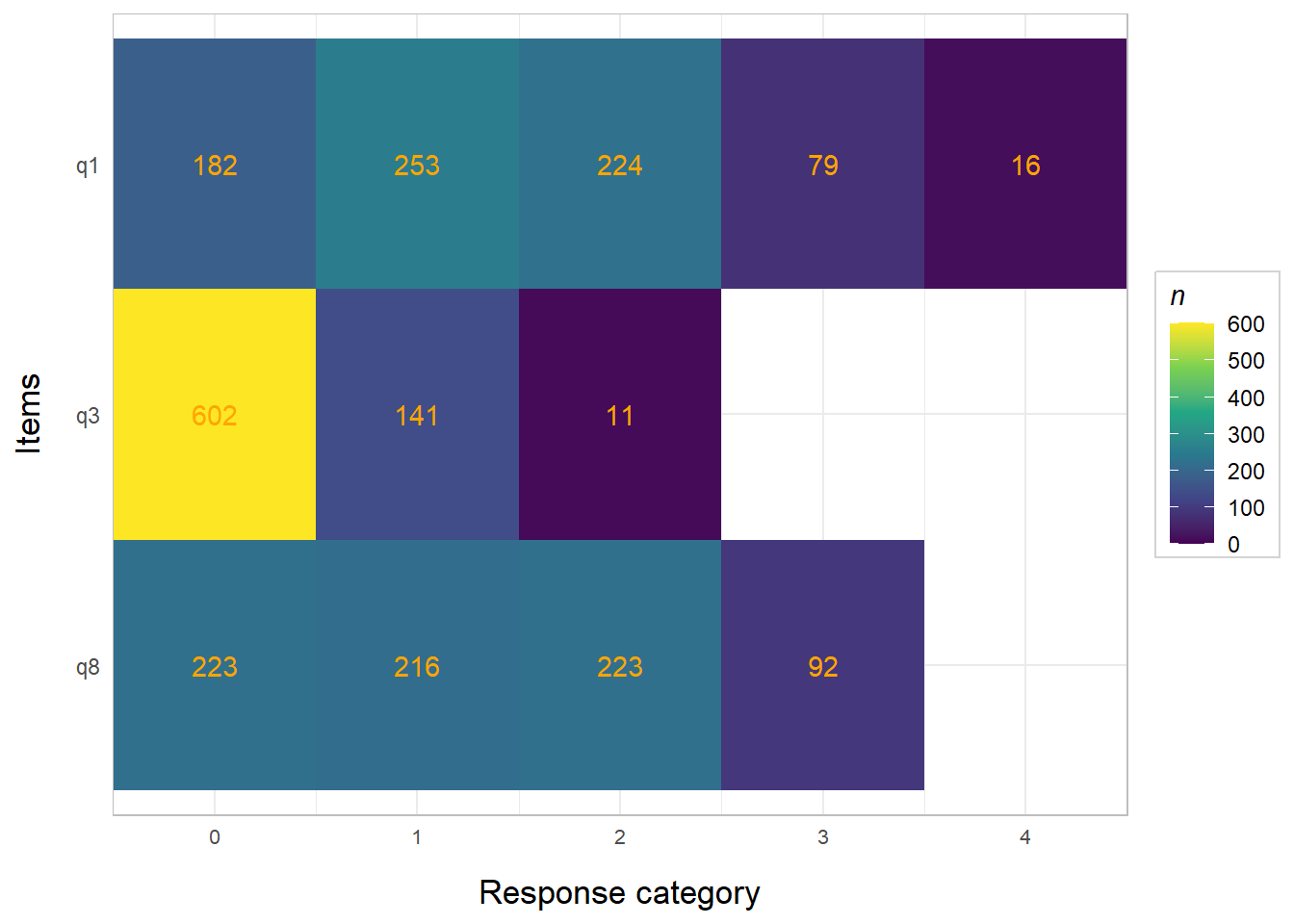

Supplement: Supplementary file 1 — Supplementary Material 1 [file 41598_2025_28073_MOESM1_ESM.zip › Supplementary/analysis_att_files/figure-html/unnamed-chunk-68-1.png]

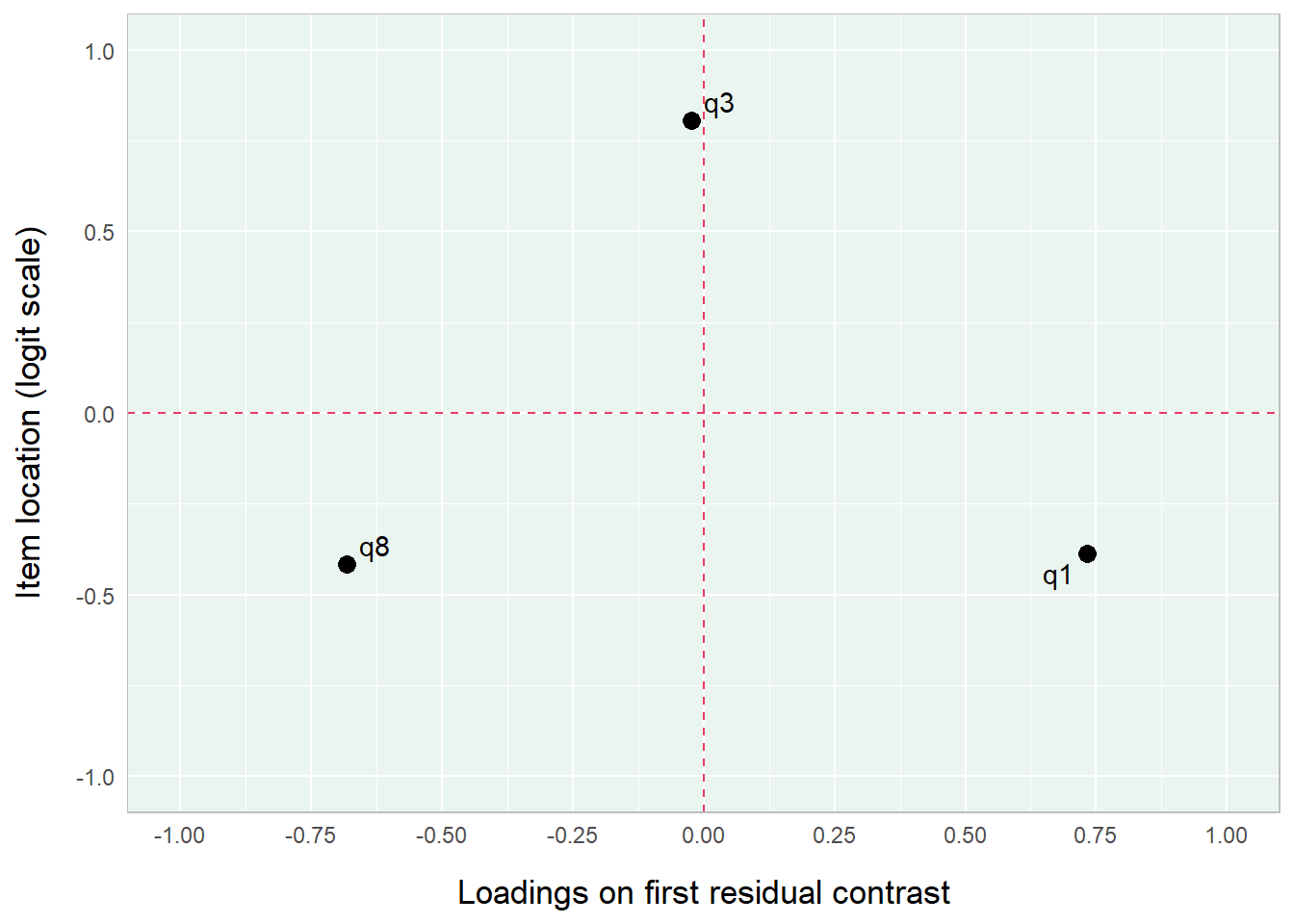

Supplement: Supplementary file 1 — Supplementary Material 1 [file 41598_2025_28073_MOESM1_ESM.zip › Supplementary/analysis_att_files/figure-html/unnamed-chunk-69-1.png]

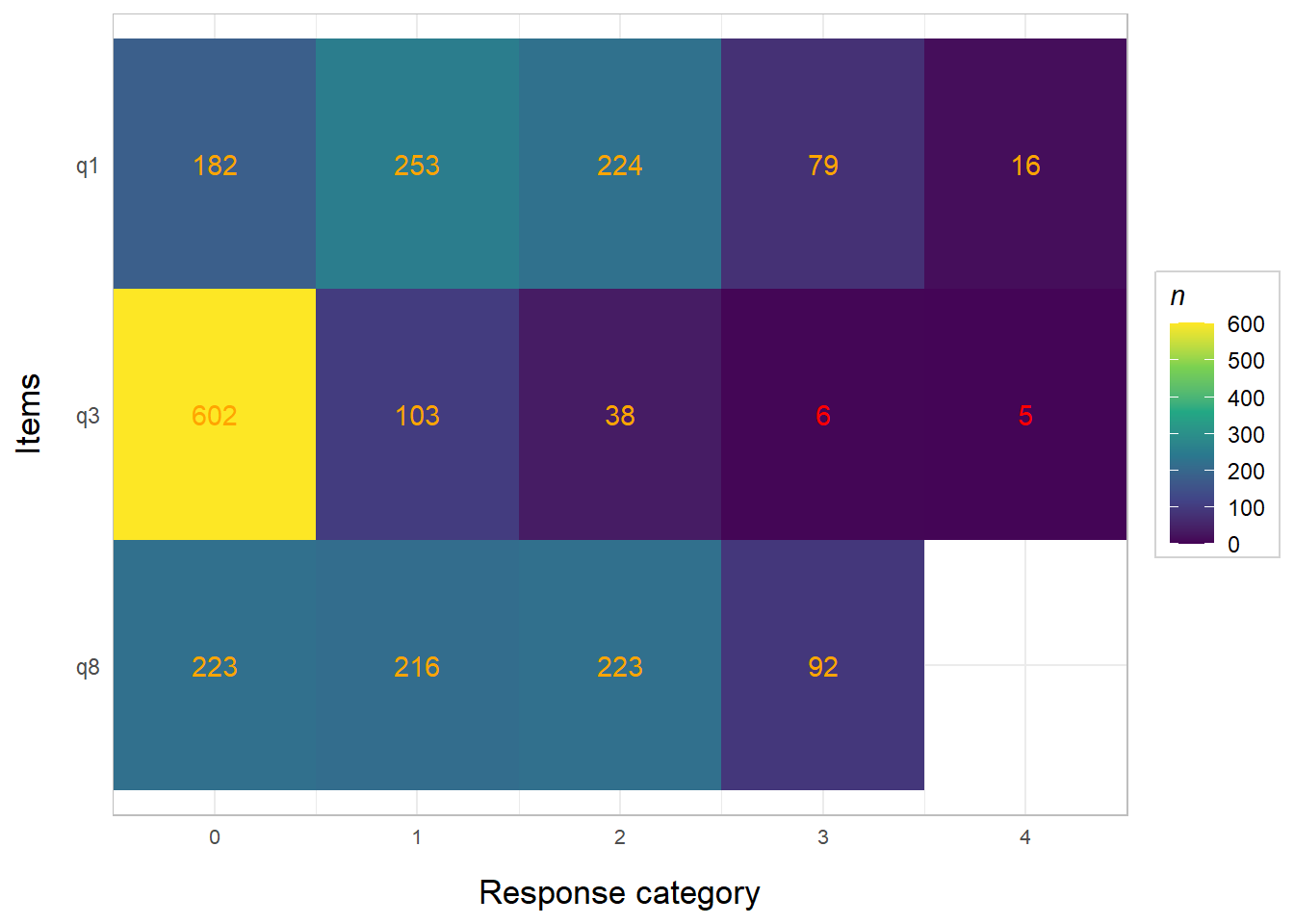

Supplement: Supplementary file 1 — Supplementary Material 1 [file 41598_2025_28073_MOESM1_ESM.zip › Supplementary/analysis_att_files/figure-html/unnamed-chunk-74-1.png]

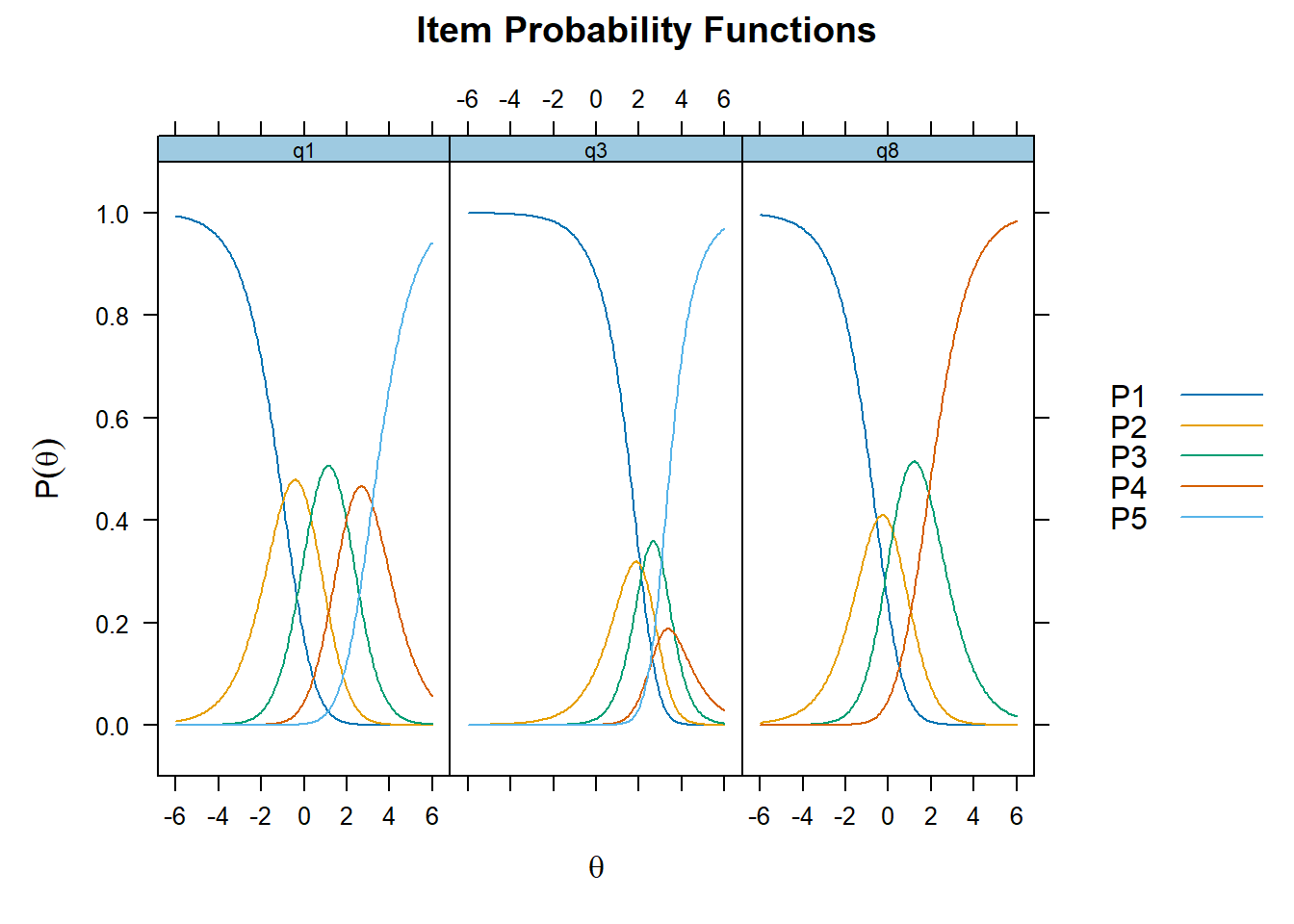

Supplement: Supplementary file 1 — Supplementary Material 1 [file 41598_2025_28073_MOESM1_ESM.zip › Supplementary/analysis_att_files/figure-html/unnamed-chunk-75-1.png]

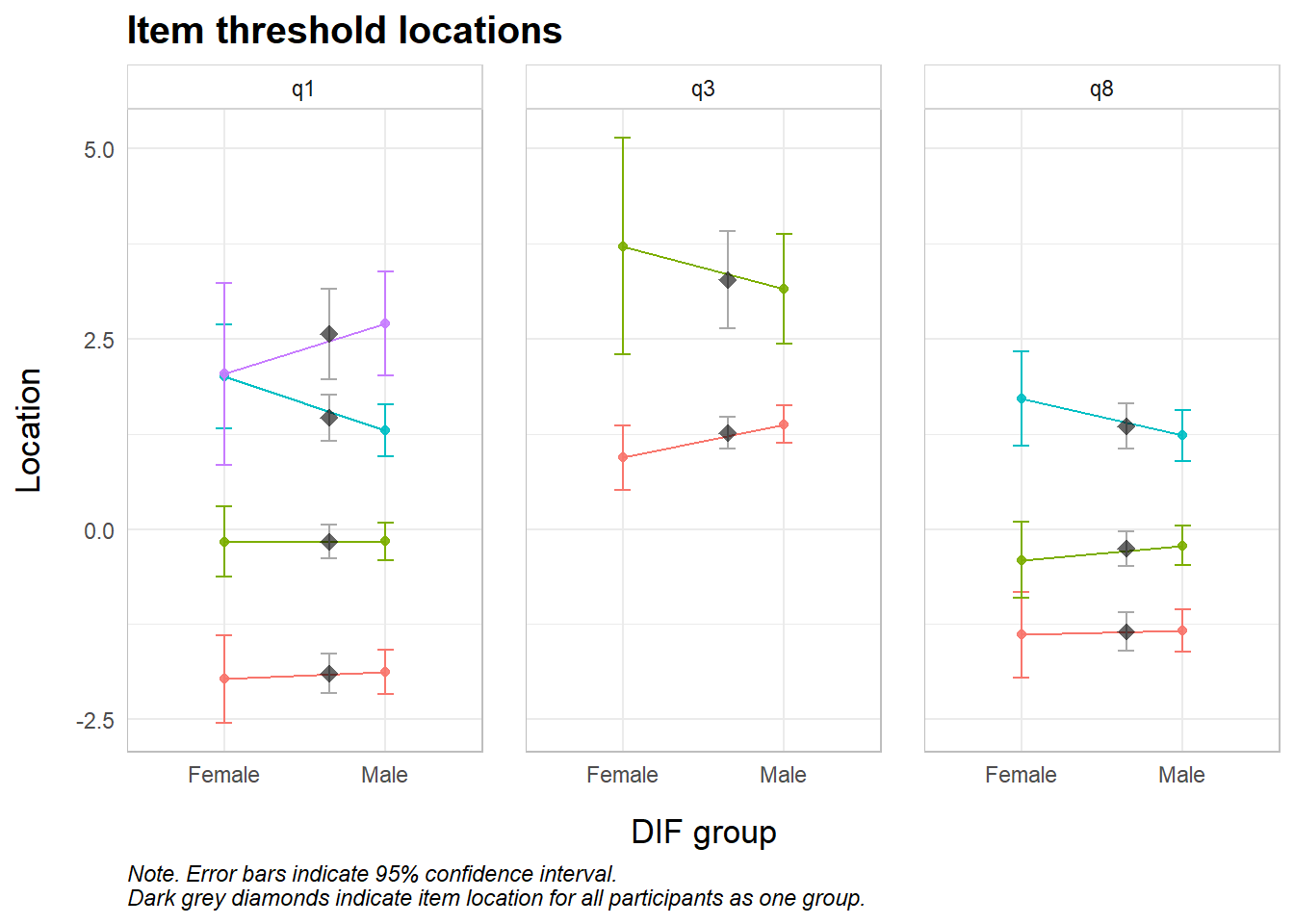

Supplement: Supplementary file 1 — Supplementary Material 1 [file 41598_2025_28073_MOESM1_ESM.zip › Supplementary/analysis_att_files/figure-html/unnamed-chunk-78-1.png]

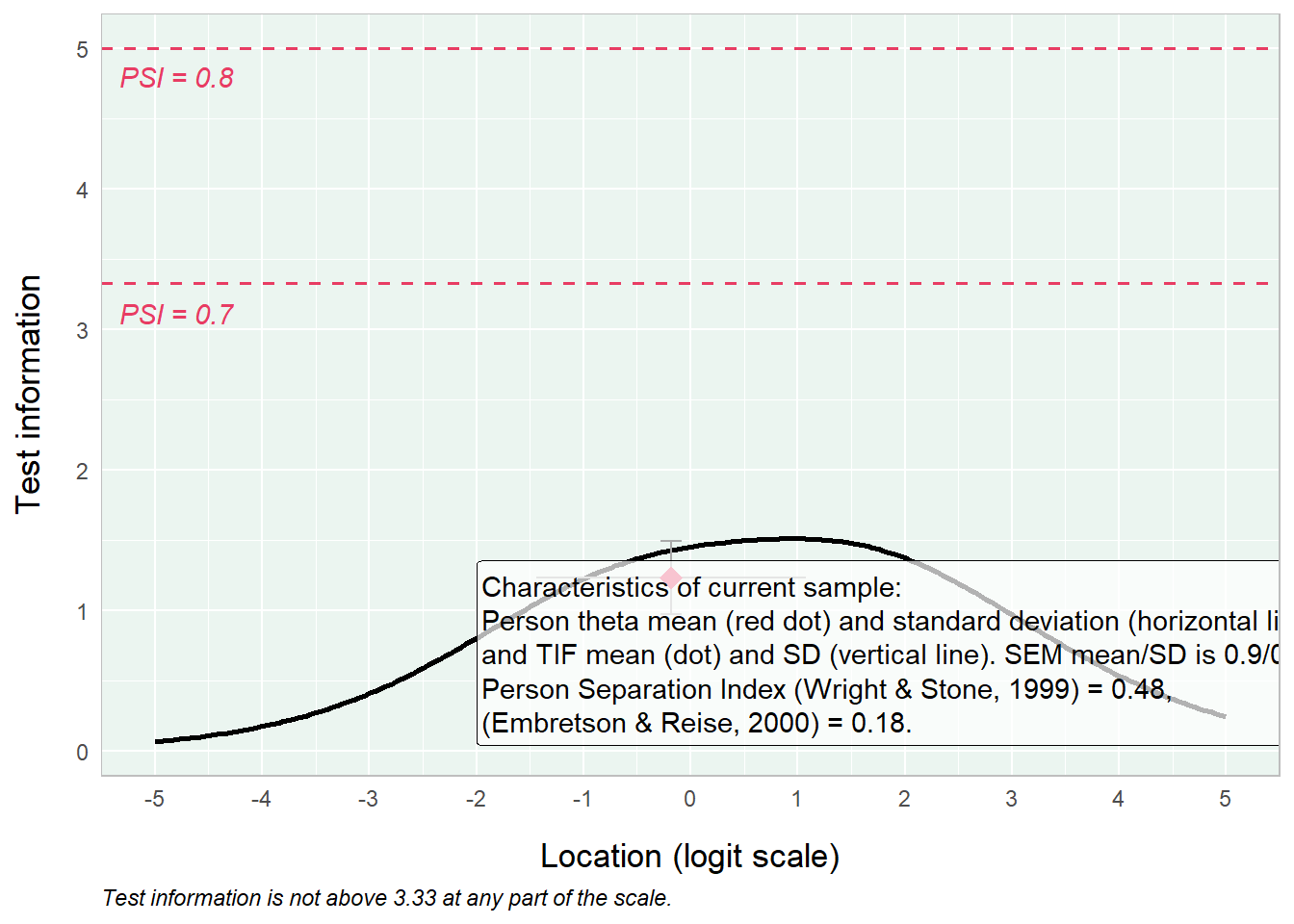

Supplement: Supplementary file 1 — Supplementary Material 1 [file 41598_2025_28073_MOESM1_ESM.zip › Supplementary/analysis_att_files/figure-html/unnamed-chunk-80-1.png]

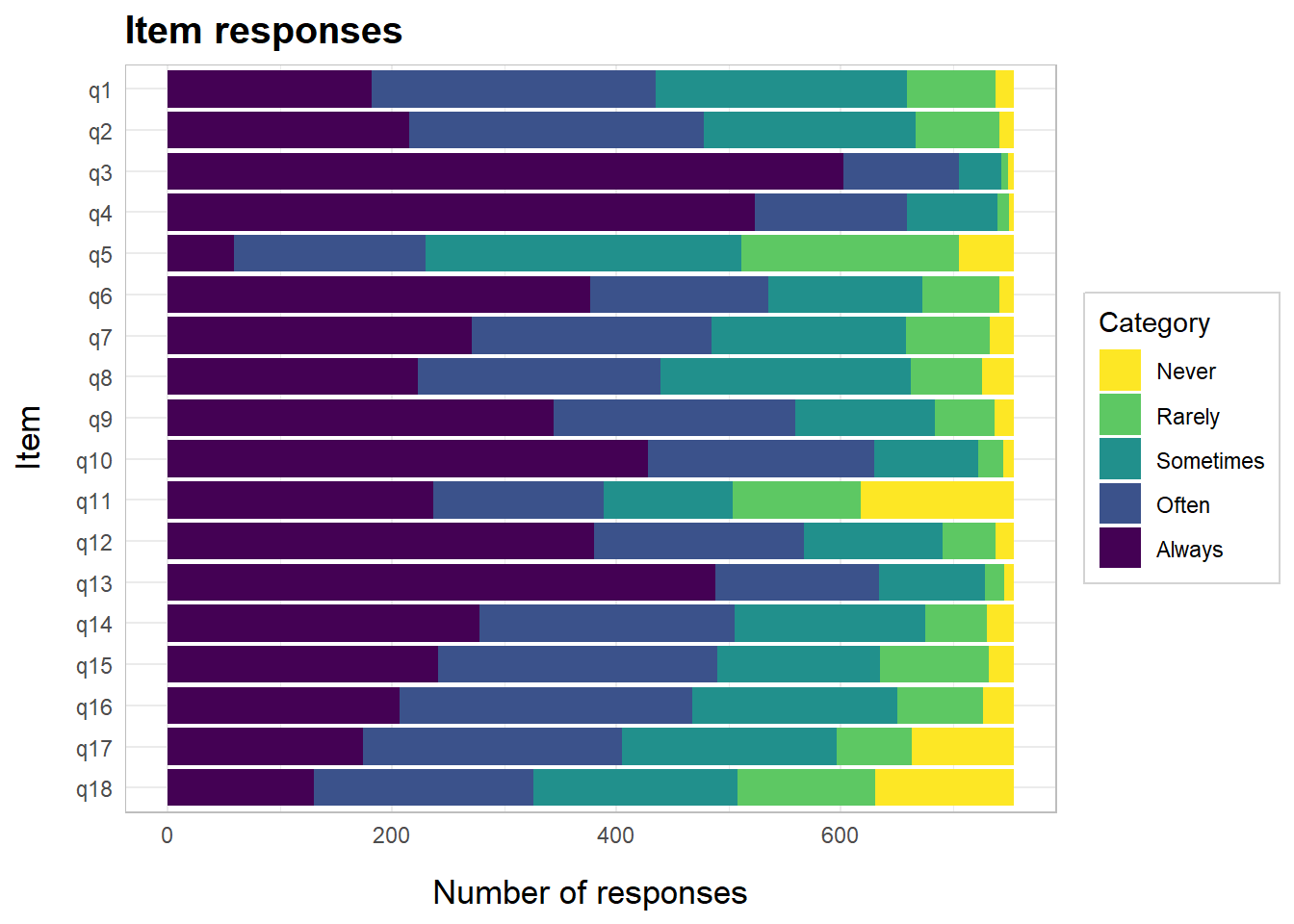

Supplement: Supplementary file 1 — Supplementary Material 1 [file 41598_2025_28073_MOESM1_ESM.zip › Supplementary/analysis_att_files/figure-html/unnamed-chunk-9-1.png]

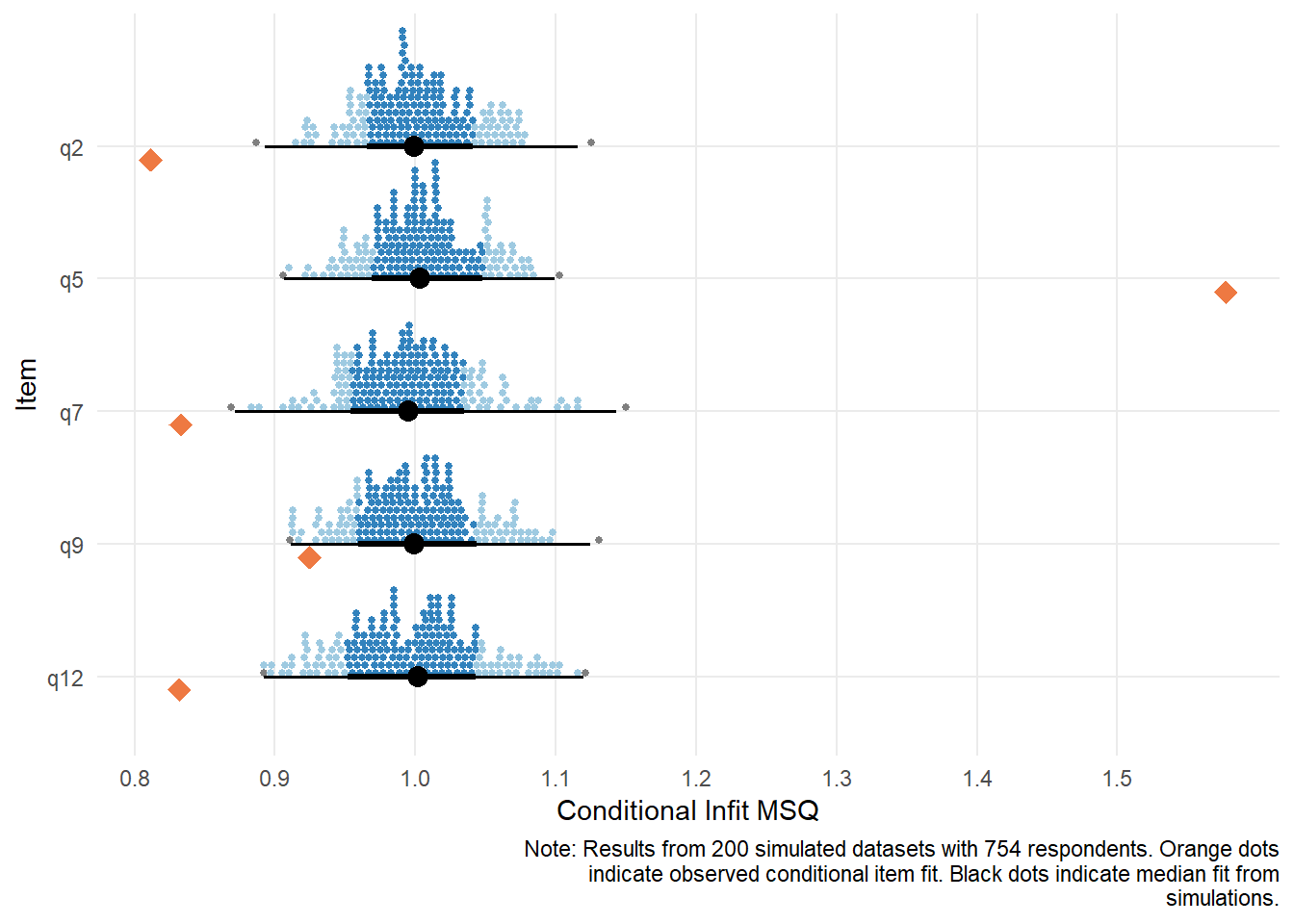

Supplement: Supplementary file 1 — Supplementary Material 1 [file 41598_2025_28073_MOESM1_ESM.zip › Supplementary/analysis_av_files/figure-html/unnamed-chunk-18-1.png]

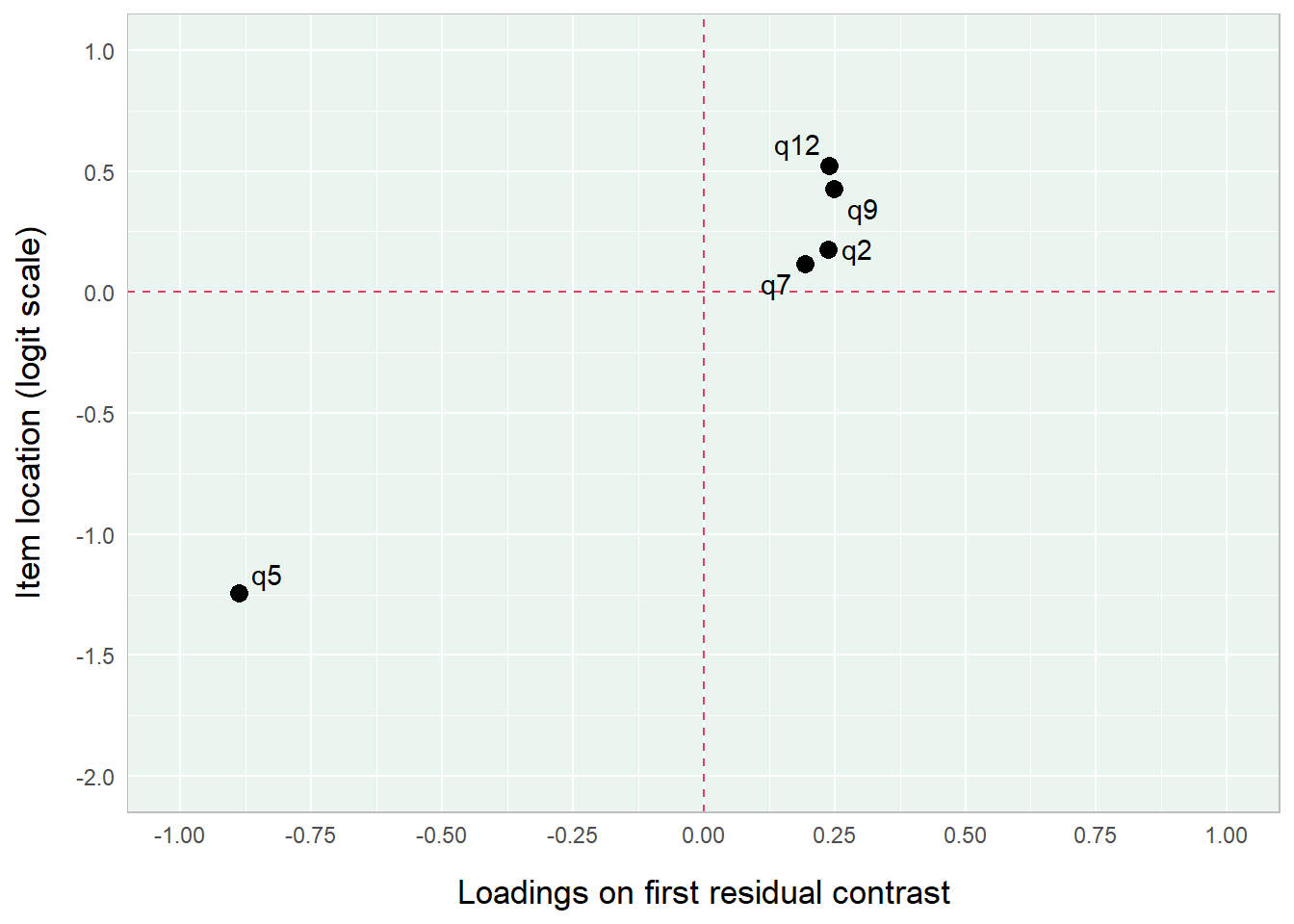

Supplement: Supplementary file 1 — Supplementary Material 1 [file 41598_2025_28073_MOESM1_ESM.zip › Supplementary/analysis_av_files/figure-html/unnamed-chunk-23-1.png]

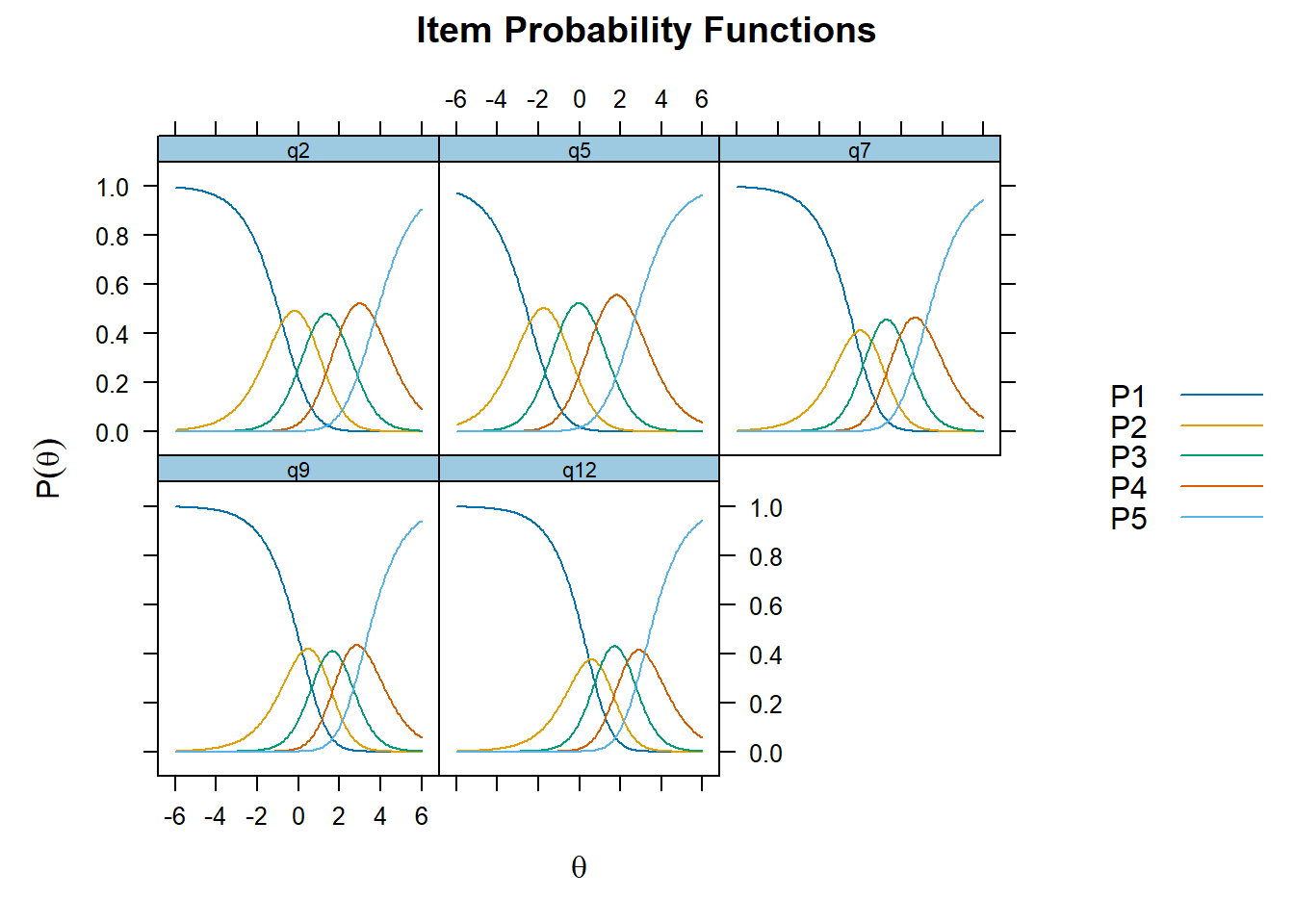

Supplement: Supplementary file 1 — Supplementary Material 1 [file 41598_2025_28073_MOESM1_ESM.zip › Supplementary/analysis_av_files/figure-html/unnamed-chunk-24-1.png]

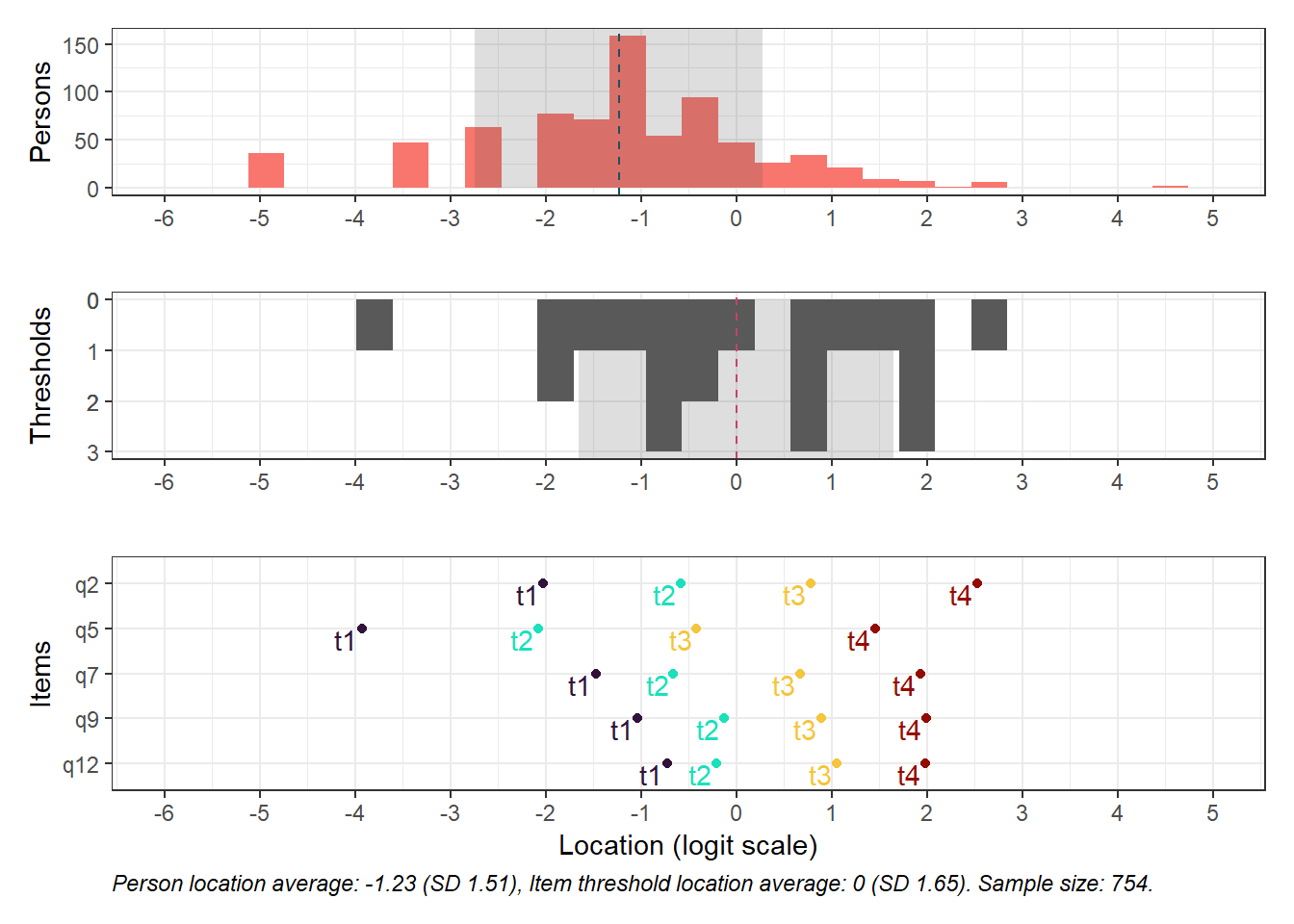

Supplement: Supplementary file 1 — Supplementary Material 1 [file 41598_2025_28073_MOESM1_ESM.zip › Supplementary/analysis_av_files/figure-html/unnamed-chunk-25-1.png]

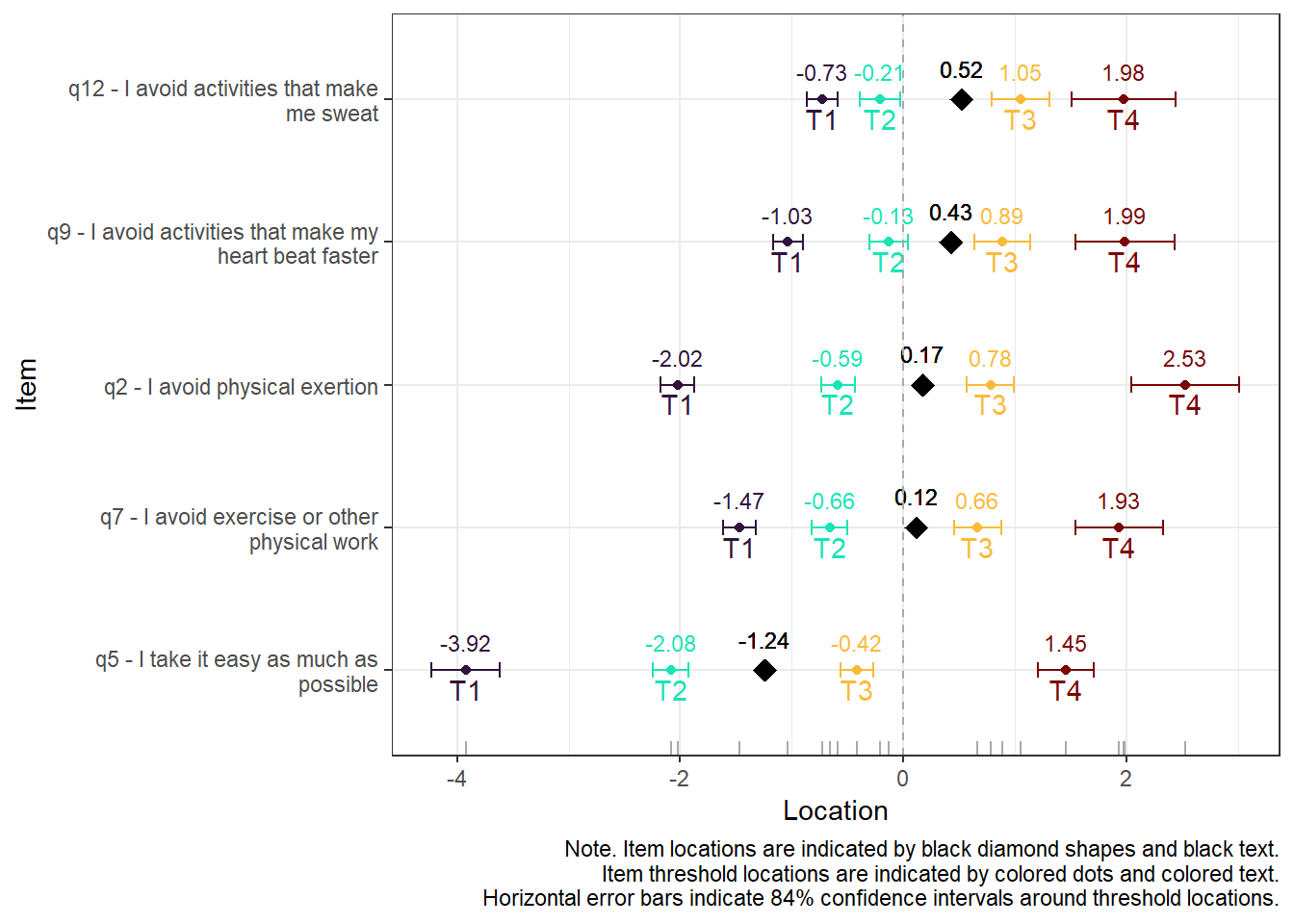

Supplement: Supplementary file 1 — Supplementary Material 1 [file 41598_2025_28073_MOESM1_ESM.zip › Supplementary/analysis_av_files/figure-html/unnamed-chunk-26-1.png]

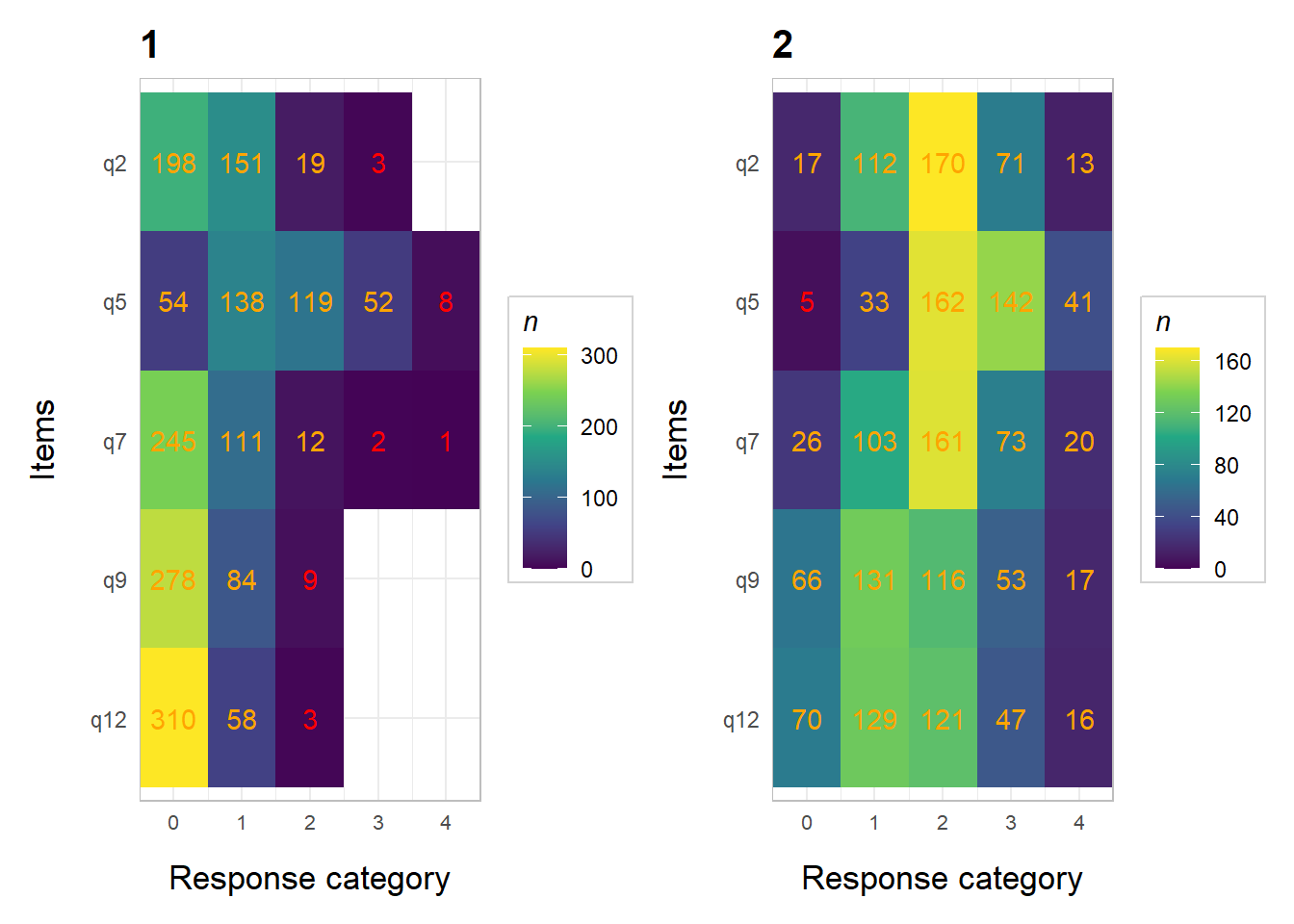

Supplement: Supplementary file 1 — Supplementary Material 1 [file 41598_2025_28073_MOESM1_ESM.zip › Supplementary/analysis_av_files/figure-html/unnamed-chunk-27-1.png]

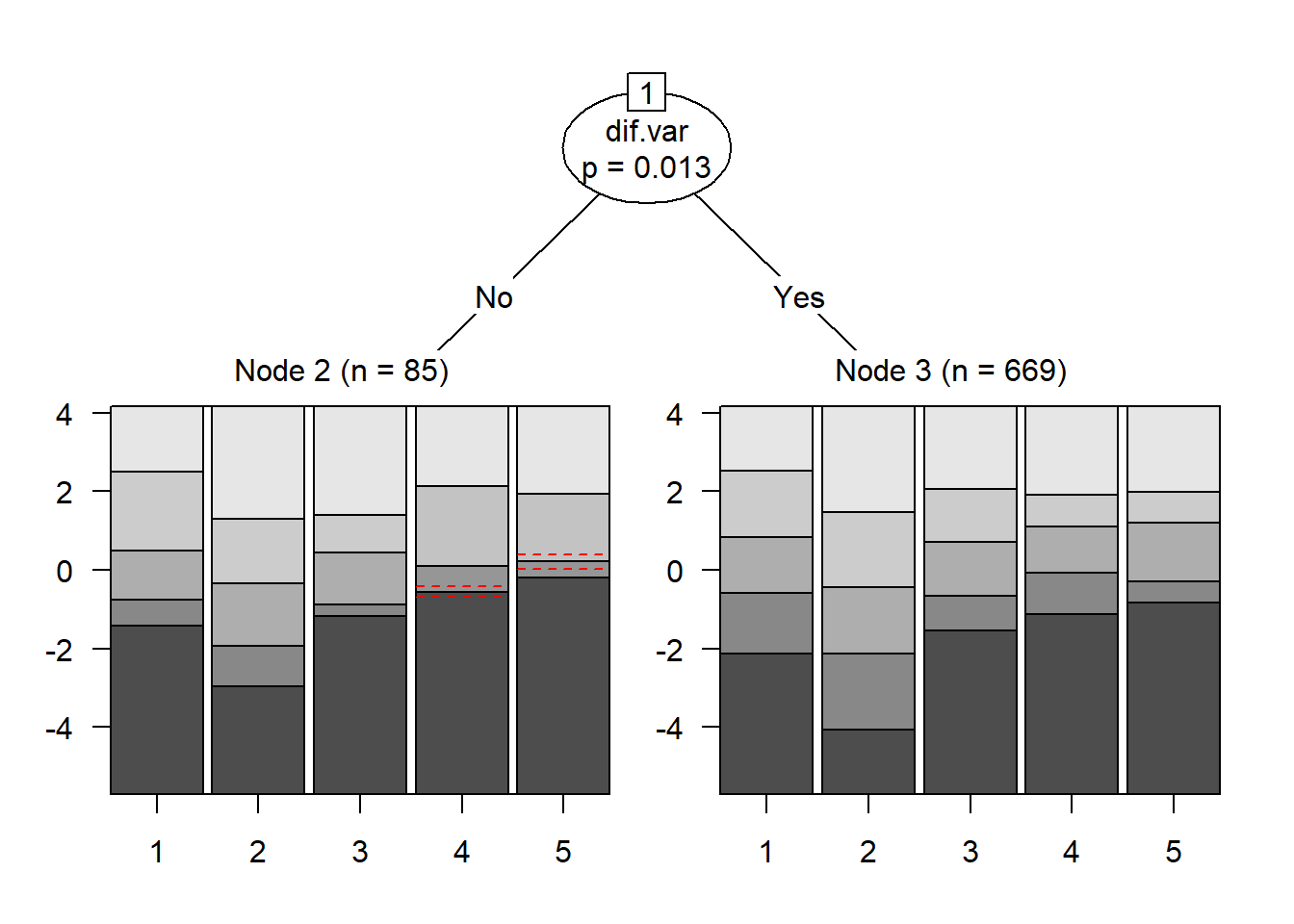

Supplement: Supplementary file 1 — Supplementary Material 1 [file 41598_2025_28073_MOESM1_ESM.zip › Supplementary/analysis_av_files/figure-html/unnamed-chunk-29-1.png]

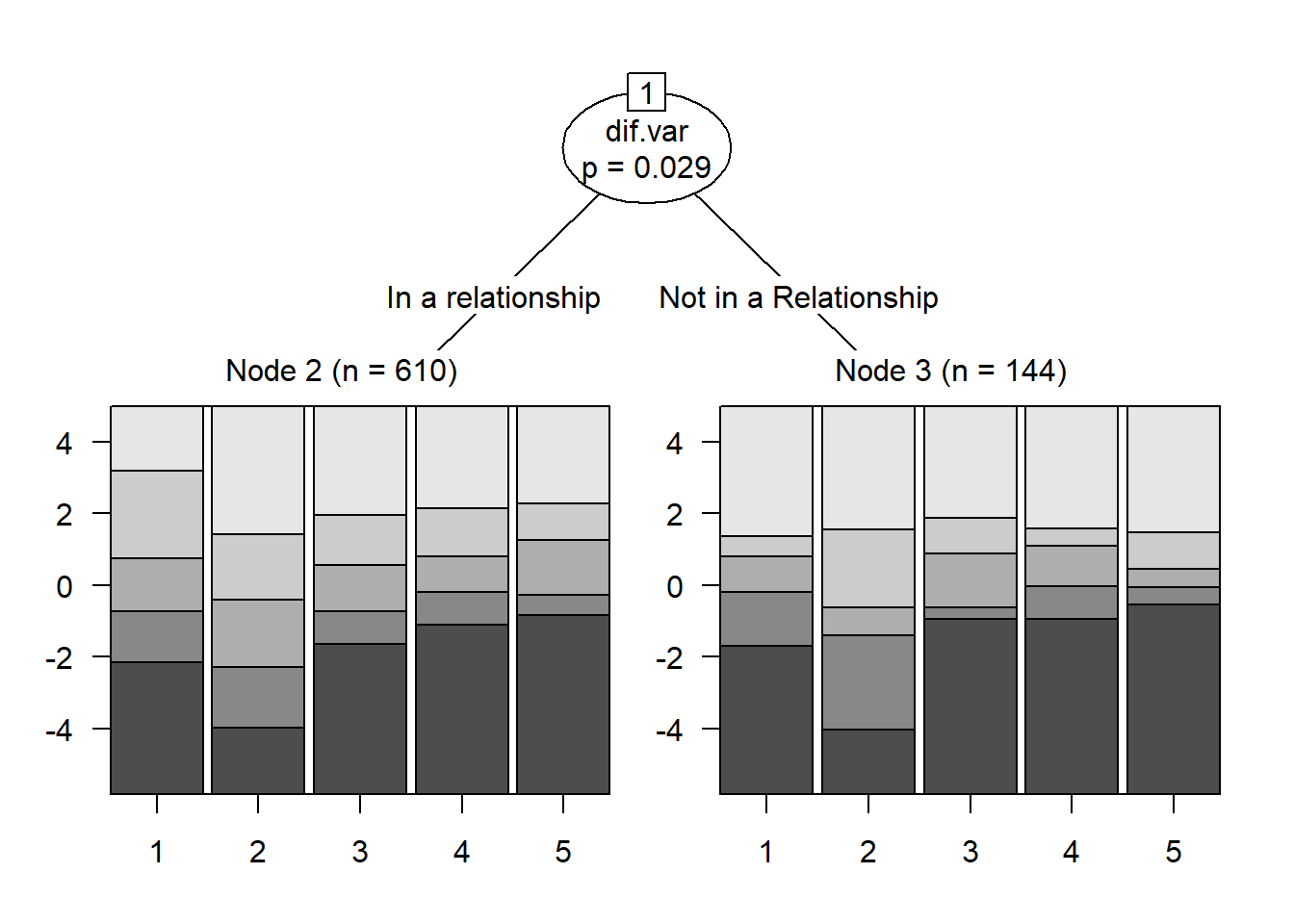

Supplement: Supplementary file 1 — Supplementary Material 1 [file 41598_2025_28073_MOESM1_ESM.zip › Supplementary/analysis_av_files/figure-html/unnamed-chunk-31-1.png]

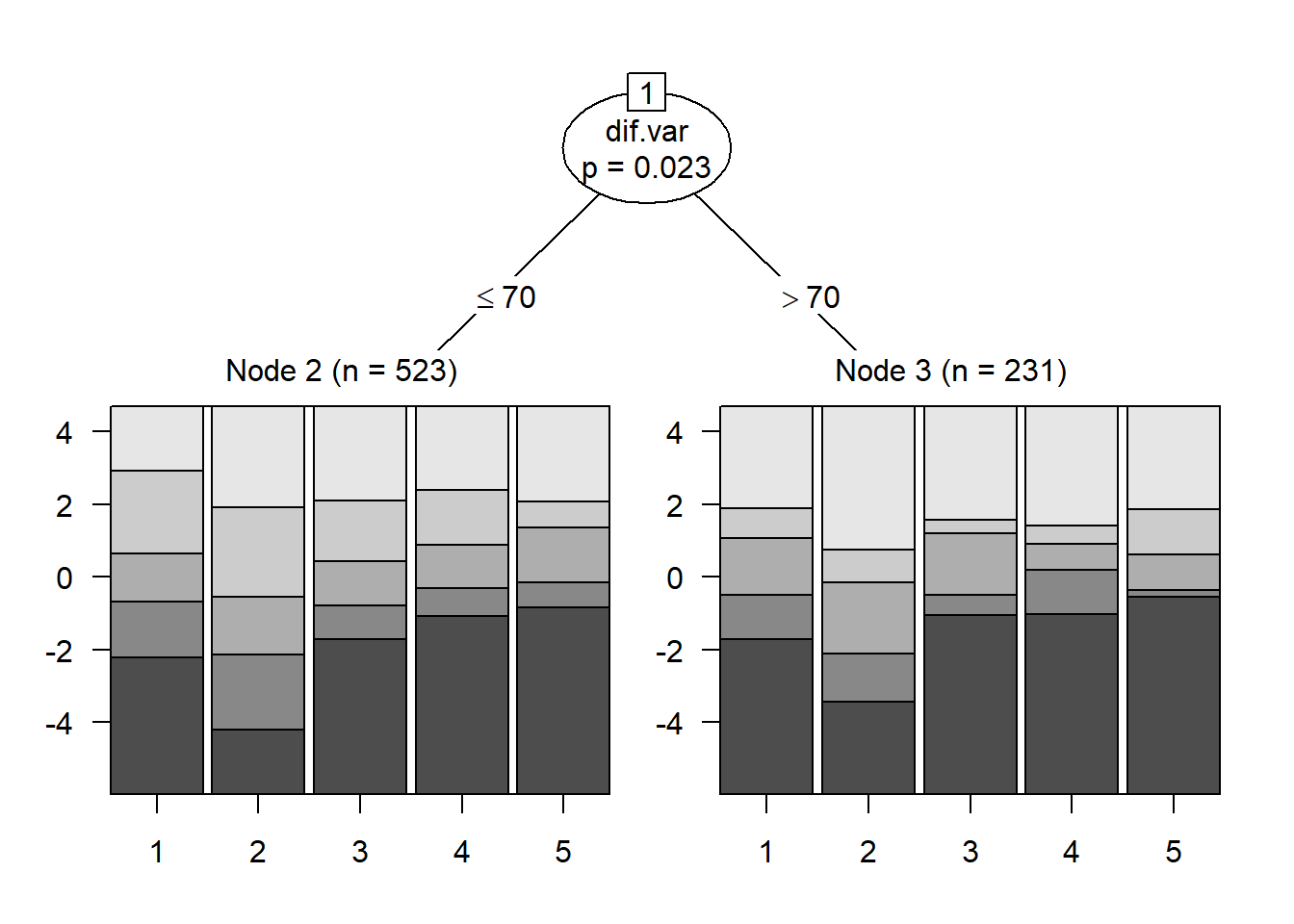

Supplement: Supplementary file 1 — Supplementary Material 1 [file 41598_2025_28073_MOESM1_ESM.zip › Supplementary/analysis_av_files/figure-html/unnamed-chunk-32-1.png]

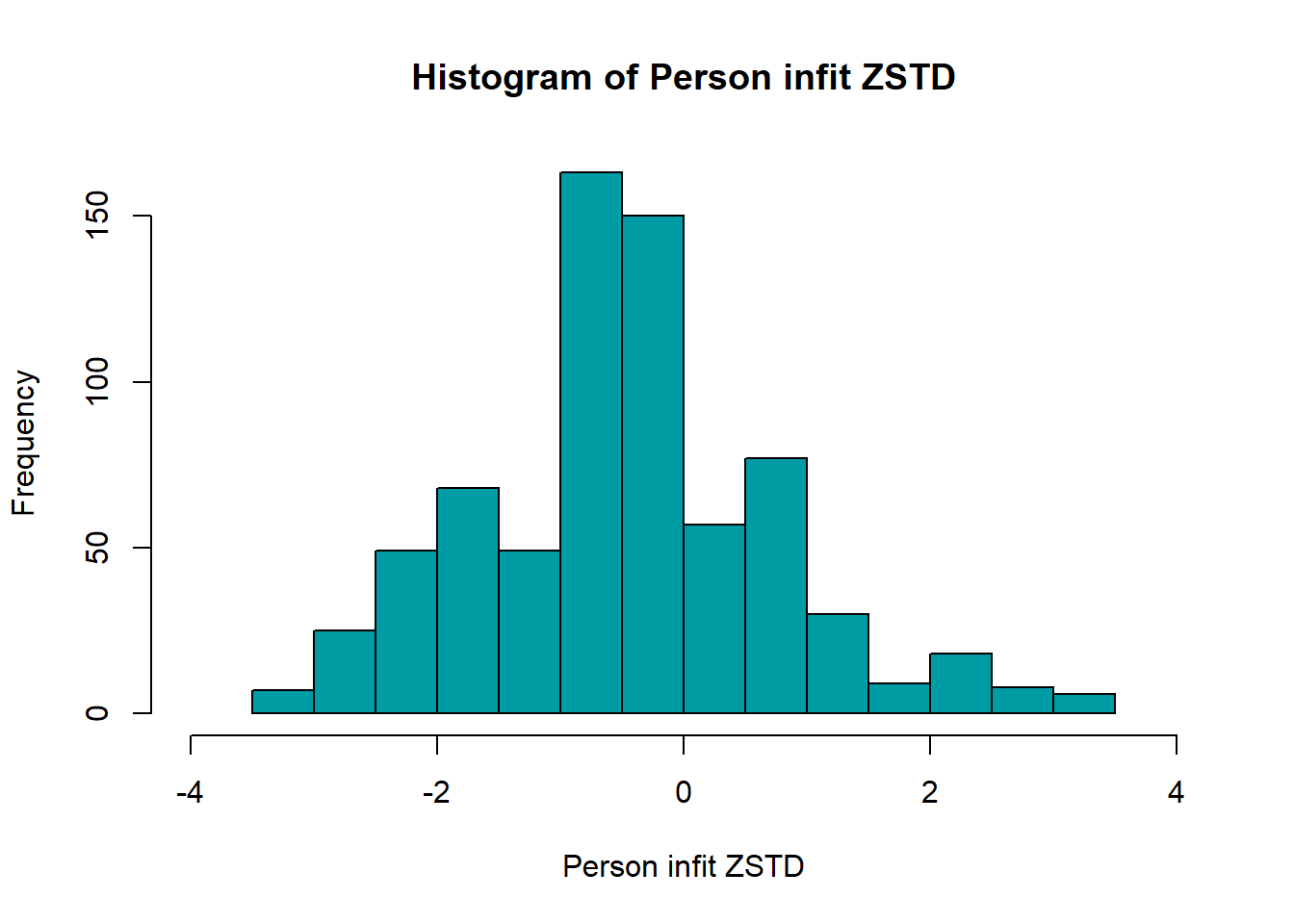

Supplement: Supplementary file 1 — Supplementary Material 1 [file 41598_2025_28073_MOESM1_ESM.zip › Supplementary/analysis_av_files/figure-html/unnamed-chunk-34-1.png]

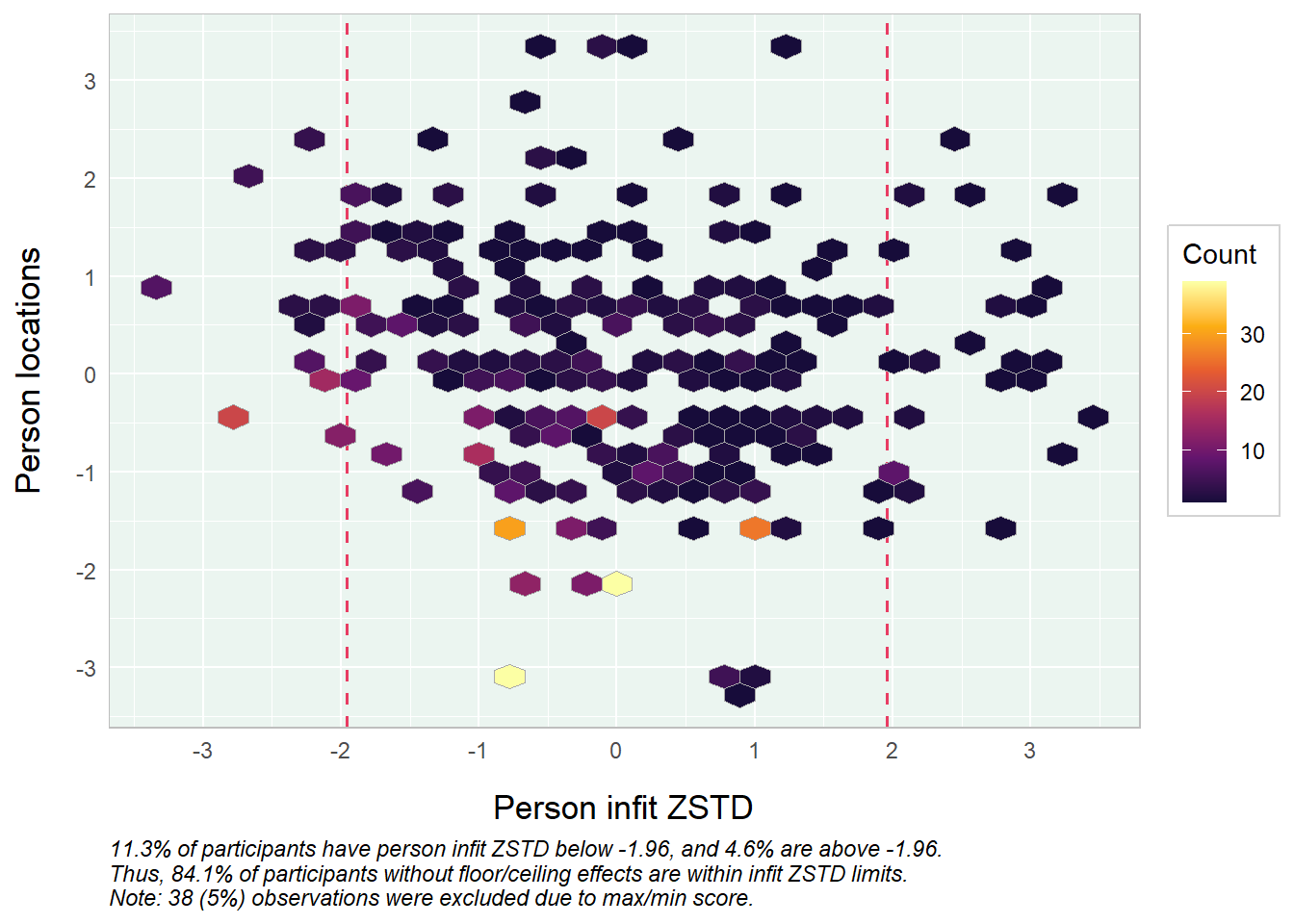

Supplement: Supplementary file 1 — Supplementary Material 1 [file 41598_2025_28073_MOESM1_ESM.zip › Supplementary/analysis_av_files/figure-html/unnamed-chunk-34-2.png]

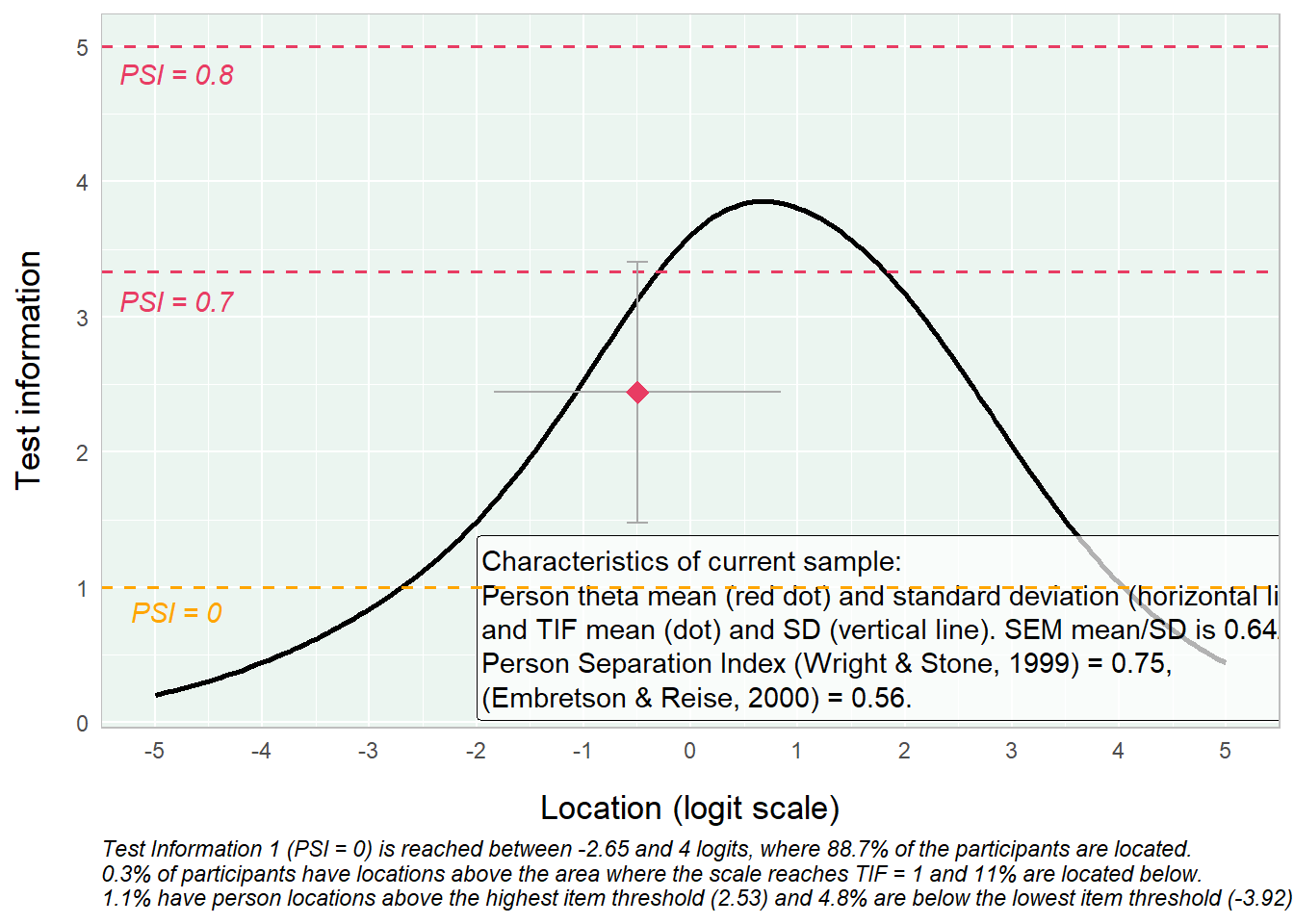

Supplement: Supplementary file 1 — Supplementary Material 1 [file 41598_2025_28073_MOESM1_ESM.zip › Supplementary/analysis_av_files/figure-html/unnamed-chunk-35-1.png]

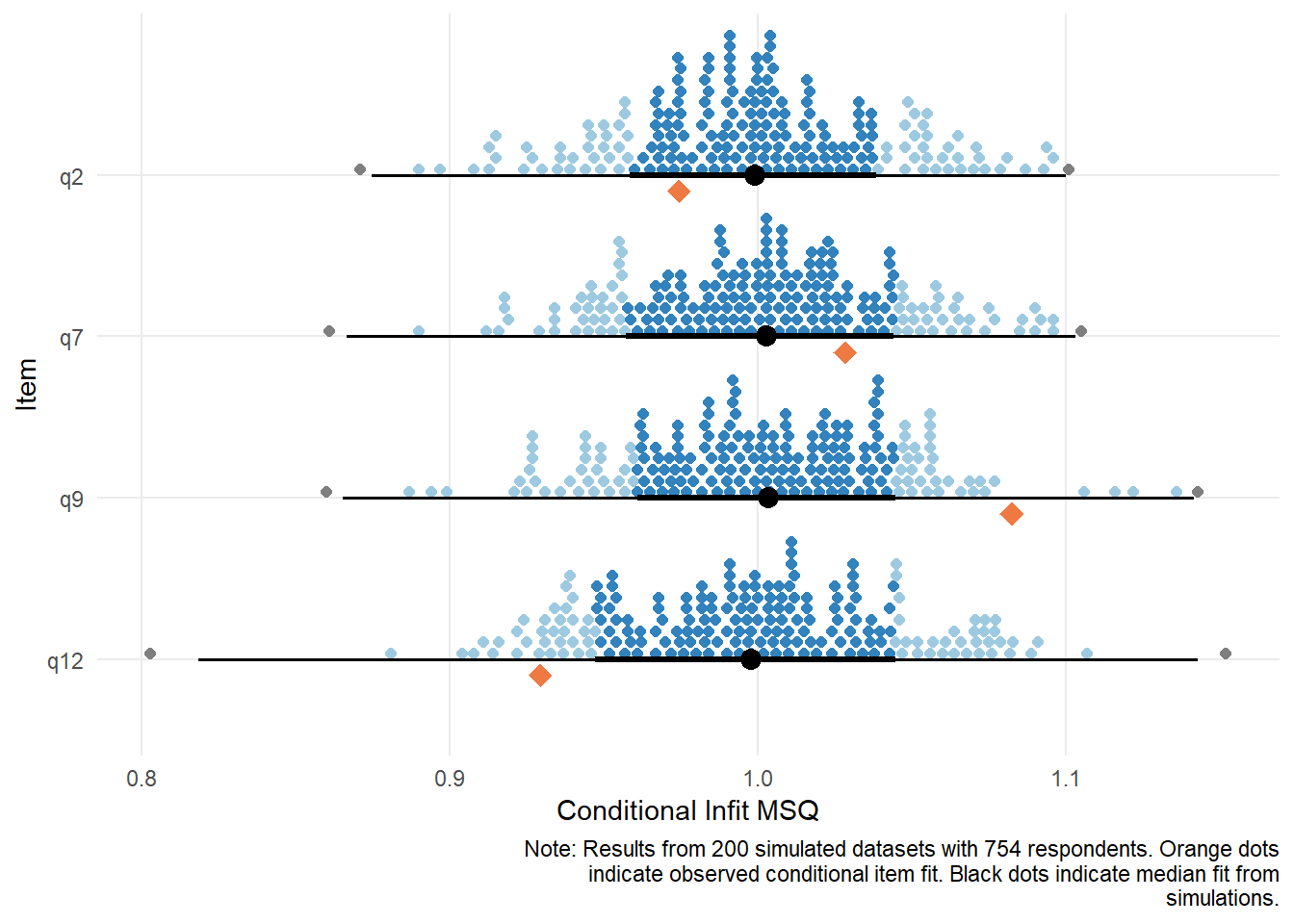

Supplement: Supplementary file 1 — Supplementary Material 1 [file 41598_2025_28073_MOESM1_ESM.zip › Supplementary/analysis_av_files/figure-html/unnamed-chunk-37-1.png]

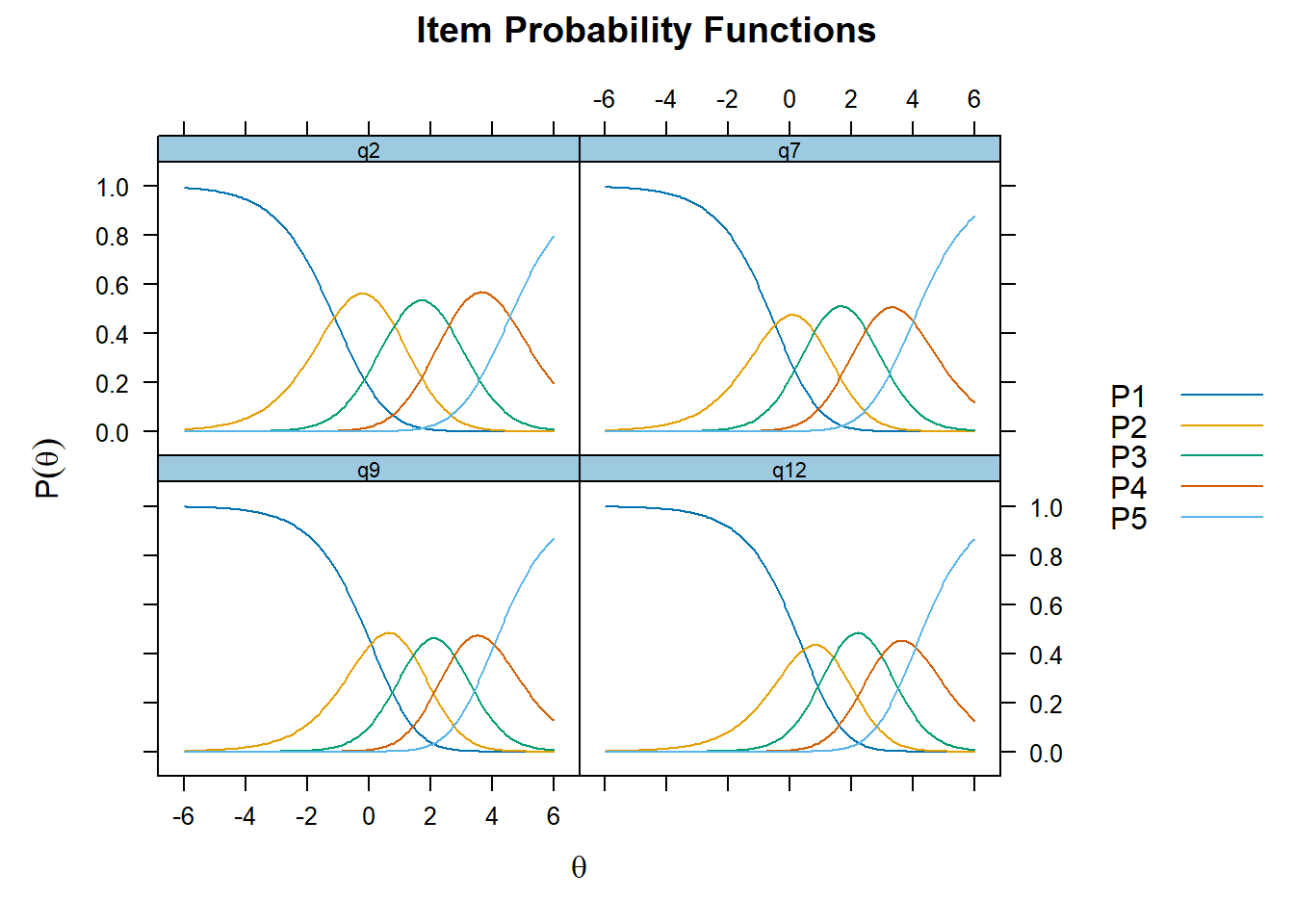

Supplement: Supplementary file 1 — Supplementary Material 1 [file 41598_2025_28073_MOESM1_ESM.zip › Supplementary/analysis_av_files/figure-html/unnamed-chunk-40-1.png]

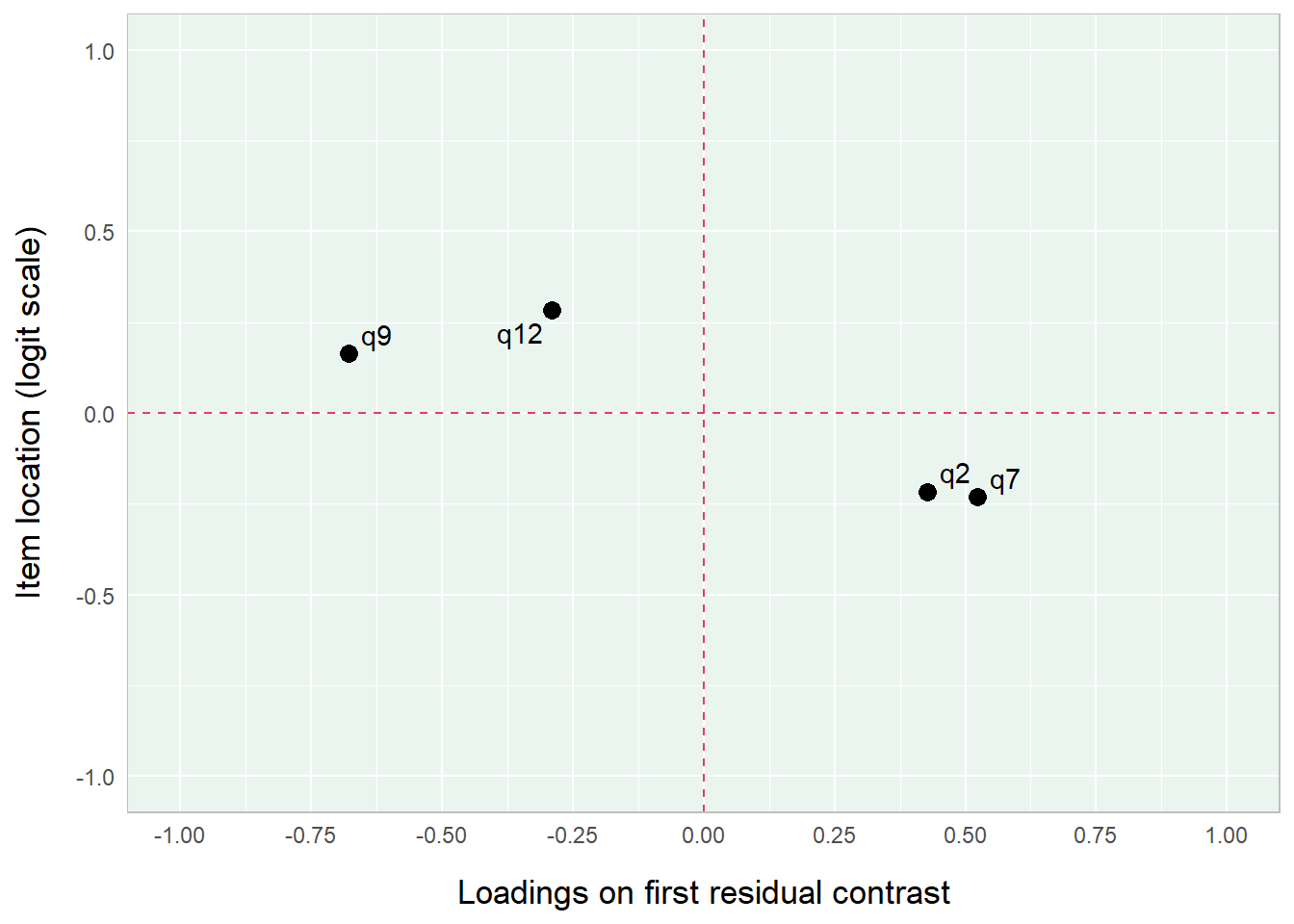

Supplement: Supplementary file 1 — Supplementary Material 1 [file 41598_2025_28073_MOESM1_ESM.zip › Supplementary/analysis_av_files/figure-html/unnamed-chunk-42-1.png]

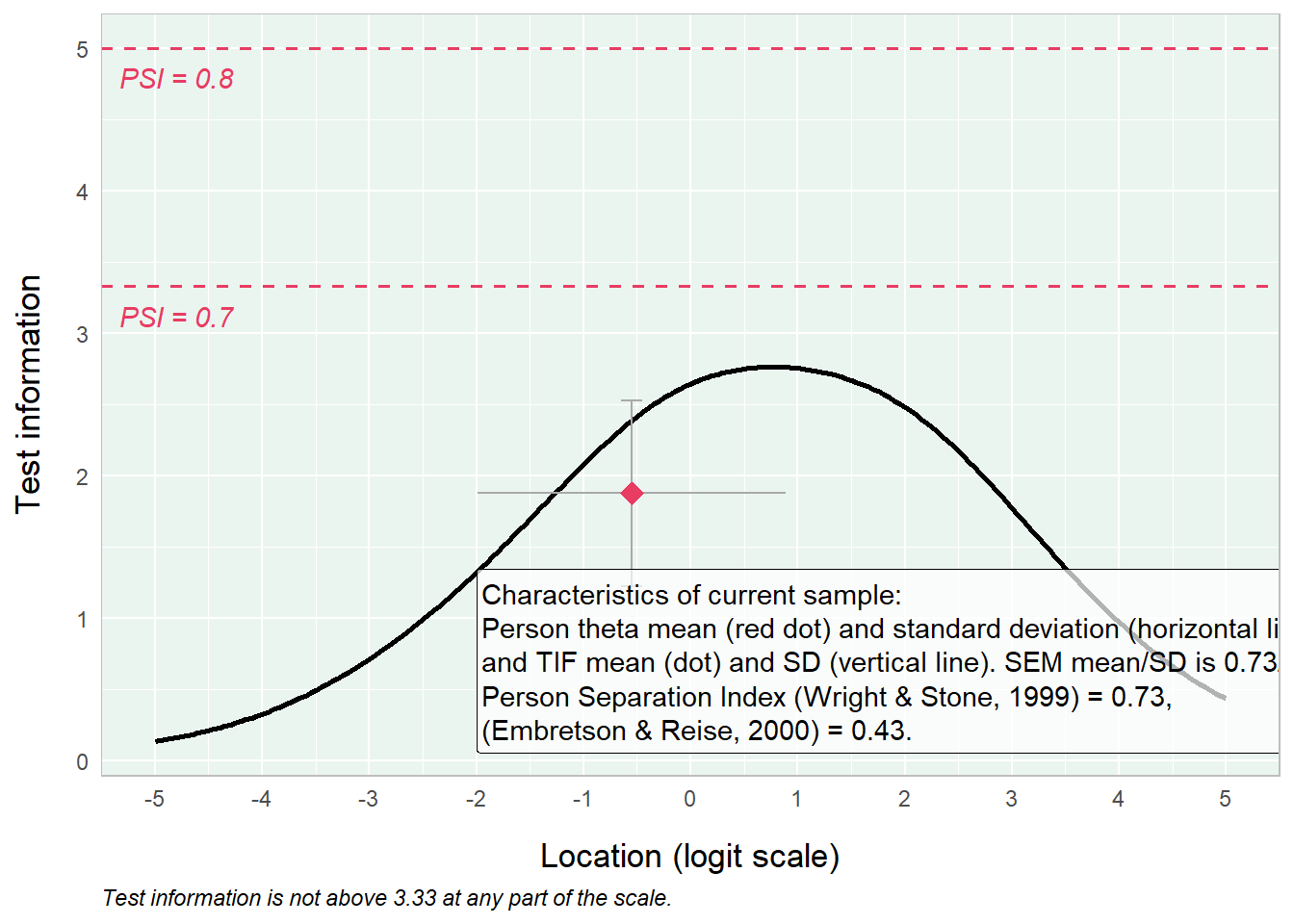

Supplement: Supplementary file 1 — Supplementary Material 1 [file 41598_2025_28073_MOESM1_ESM.zip › Supplementary/analysis_av_files/figure-html/unnamed-chunk-44-1.png]

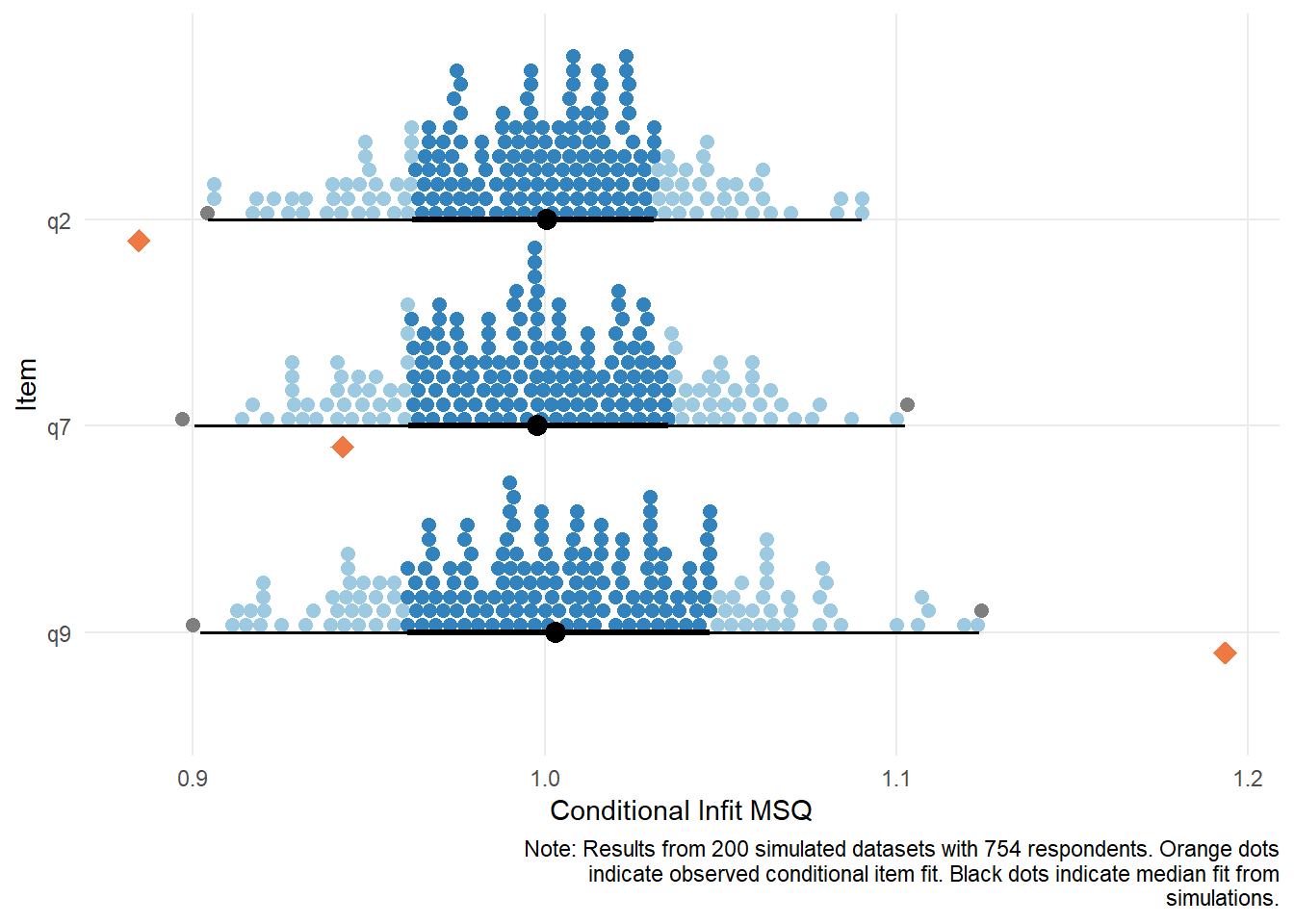

Supplement: Supplementary file 1 — Supplementary Material 1 [file 41598_2025_28073_MOESM1_ESM.zip › Supplementary/analysis_av_files/figure-html/unnamed-chunk-46-1.png]

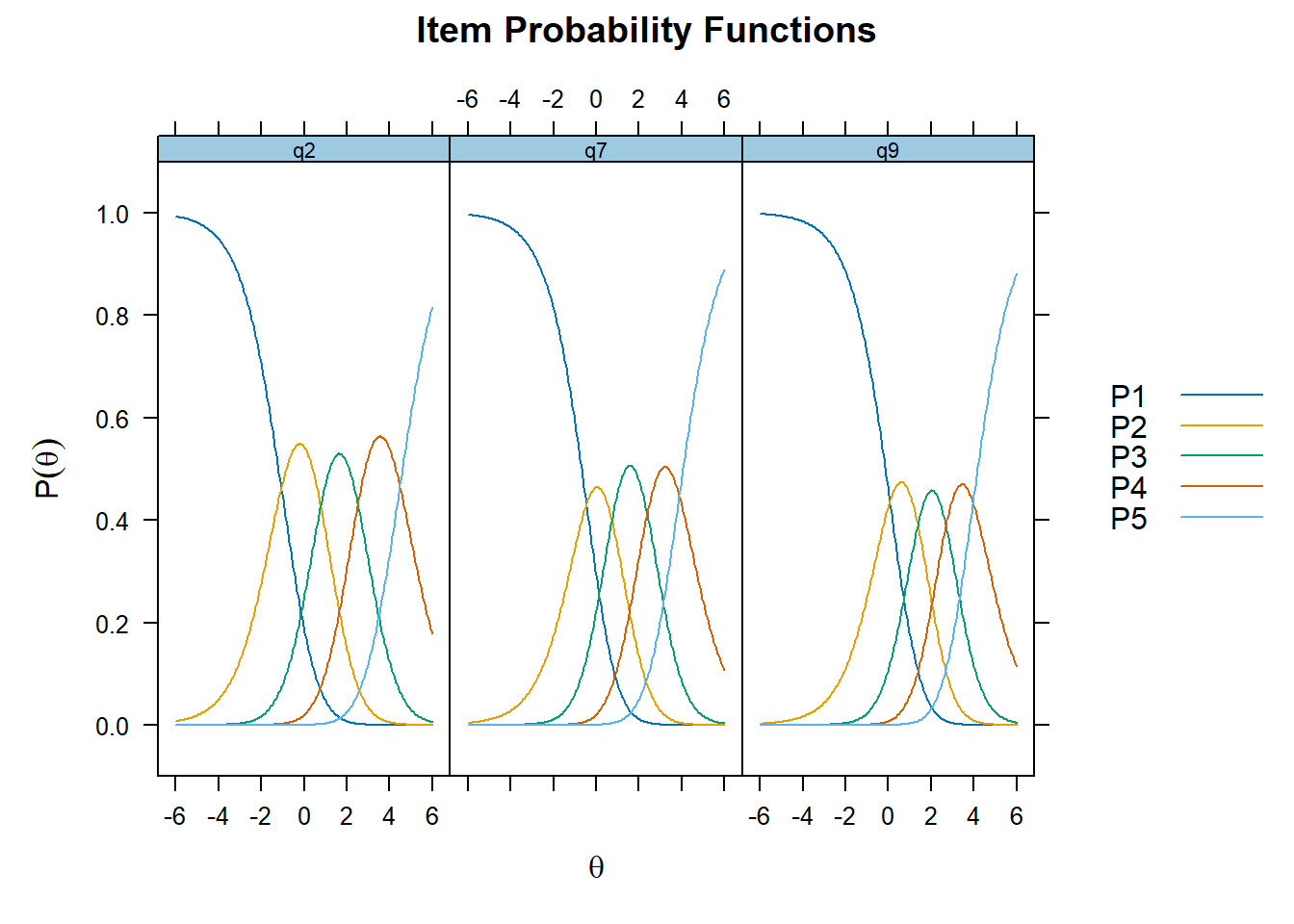

Supplement: Supplementary file 1 — Supplementary Material 1 [file 41598_2025_28073_MOESM1_ESM.zip › Supplementary/analysis_av_files/figure-html/unnamed-chunk-48-1.png]

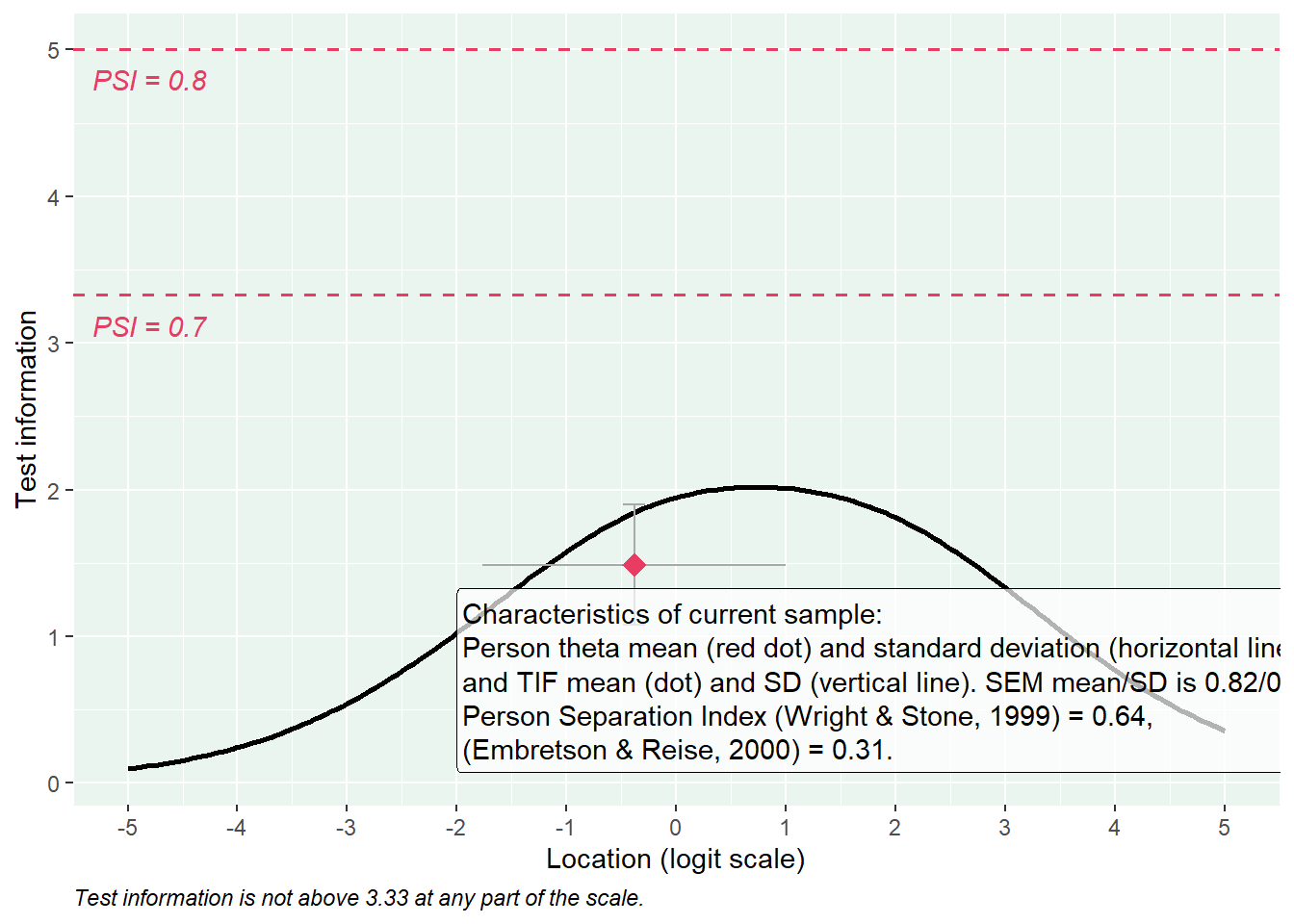

Supplement: Supplementary file 1 — Supplementary Material 1 [file 41598_2025_28073_MOESM1_ESM.zip › Supplementary/analysis_av_files/figure-html/unnamed-chunk-52-1.png]

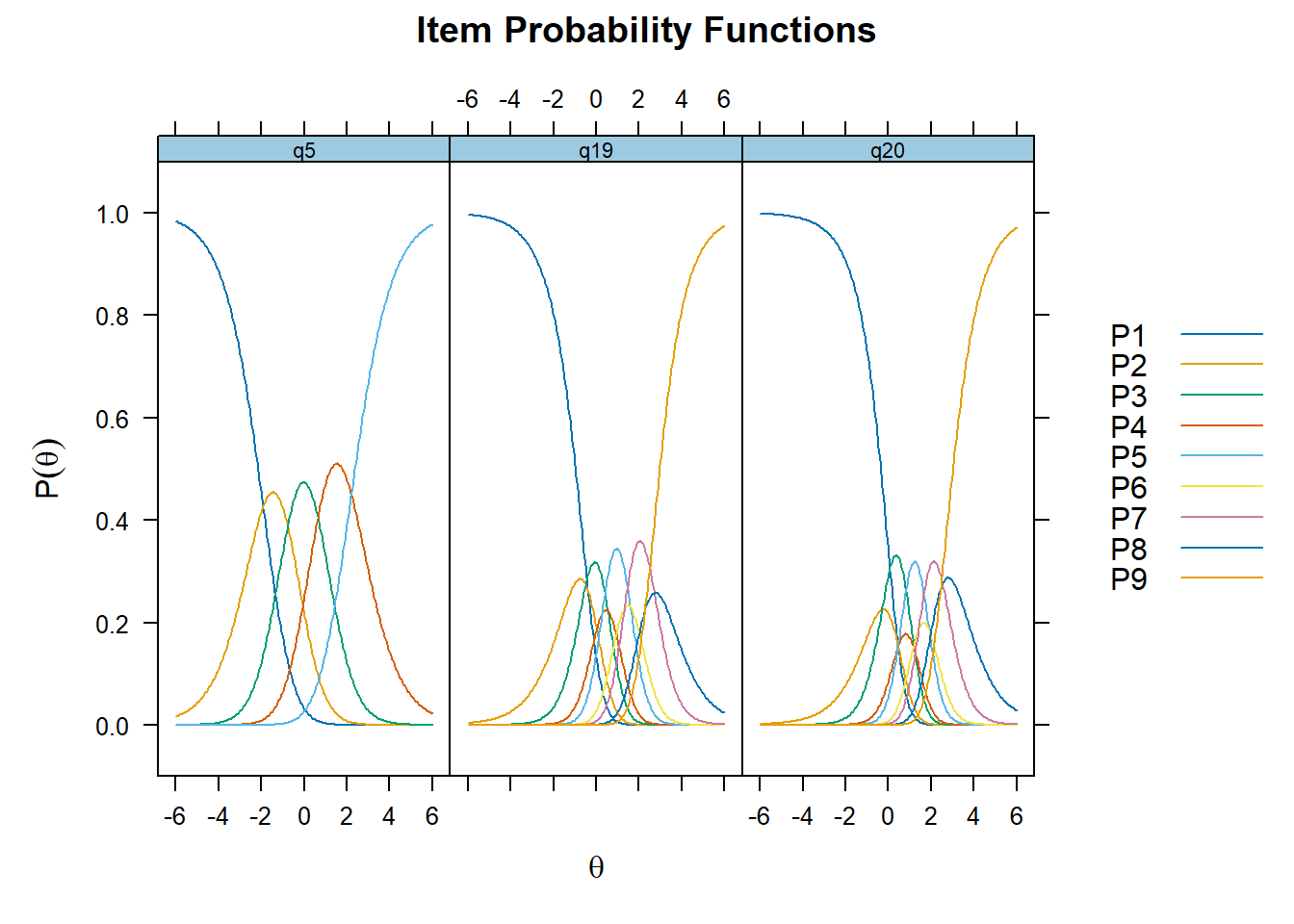

Supplement: Supplementary file 1 — Supplementary Material 1 [file 41598_2025_28073_MOESM1_ESM.zip › Supplementary/analysis_av_files/figure-html/unnamed-chunk-55-1.png]

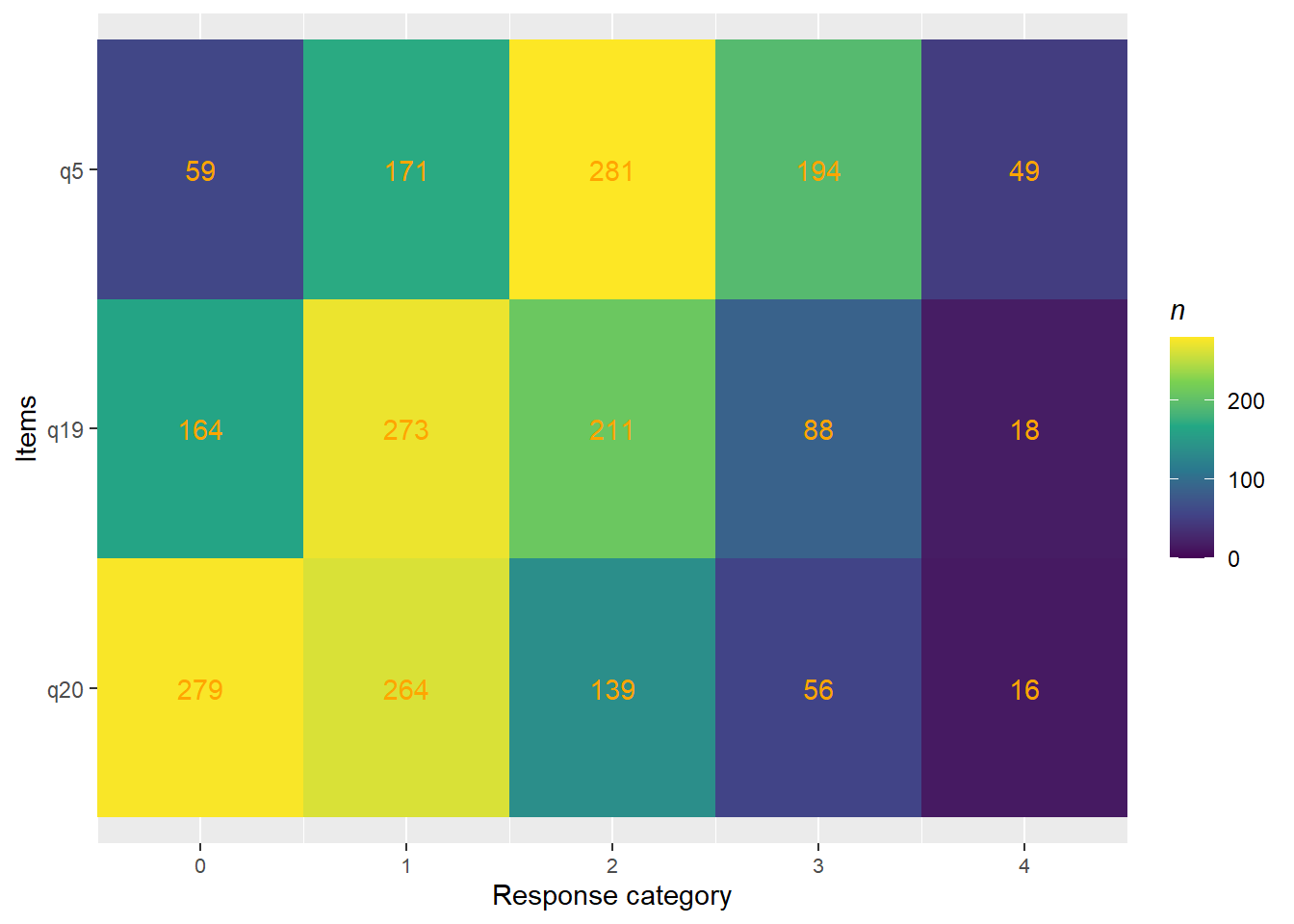

Supplement: Supplementary file 1 — Supplementary Material 1 [file 41598_2025_28073_MOESM1_ESM.zip › Supplementary/analysis_av_files/figure-html/unnamed-chunk-56-1.png]

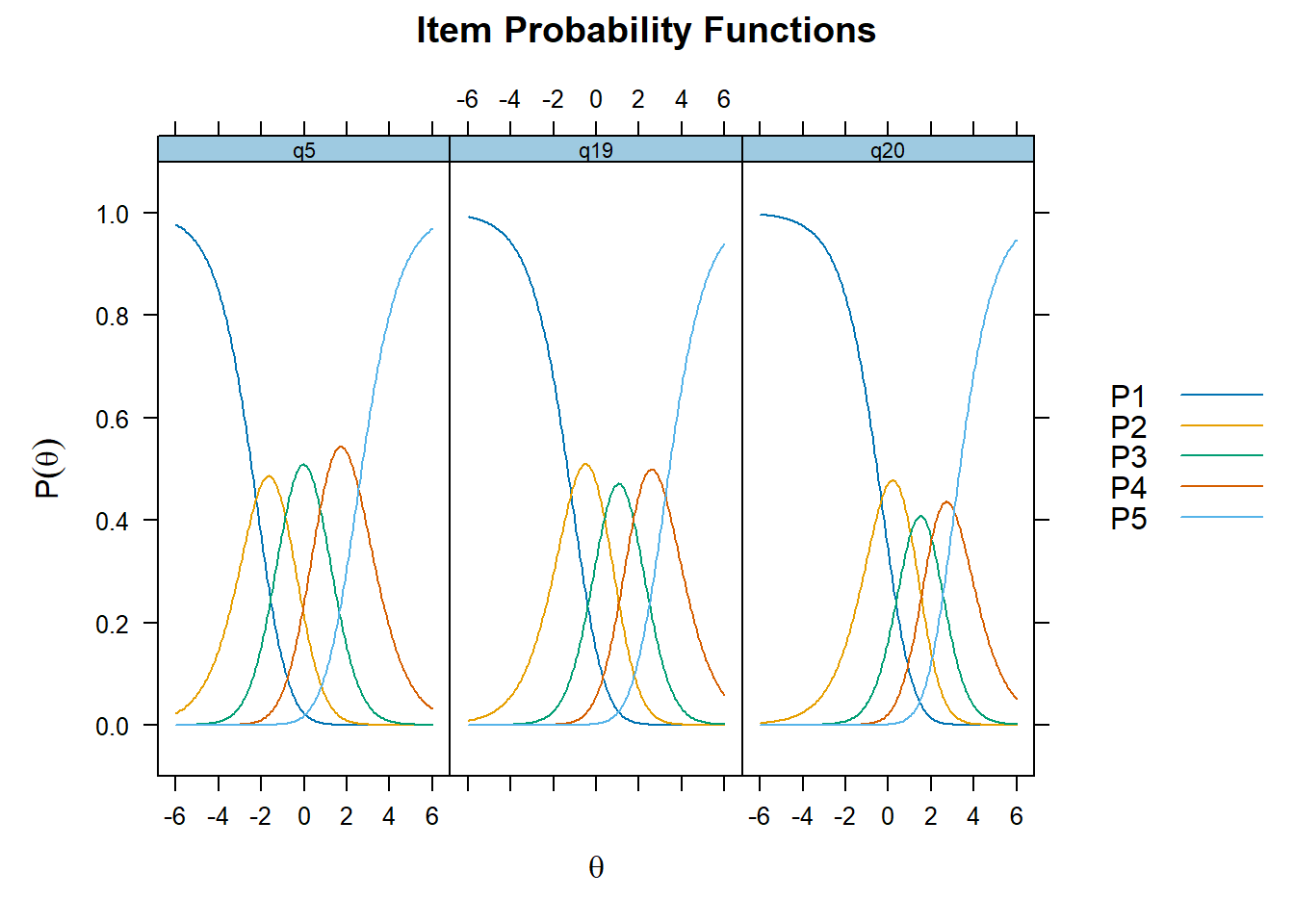

Supplement: Supplementary file 1 — Supplementary Material 1 [file 41598_2025_28073_MOESM1_ESM.zip › Supplementary/analysis_av_files/figure-html/unnamed-chunk-56-2.png]

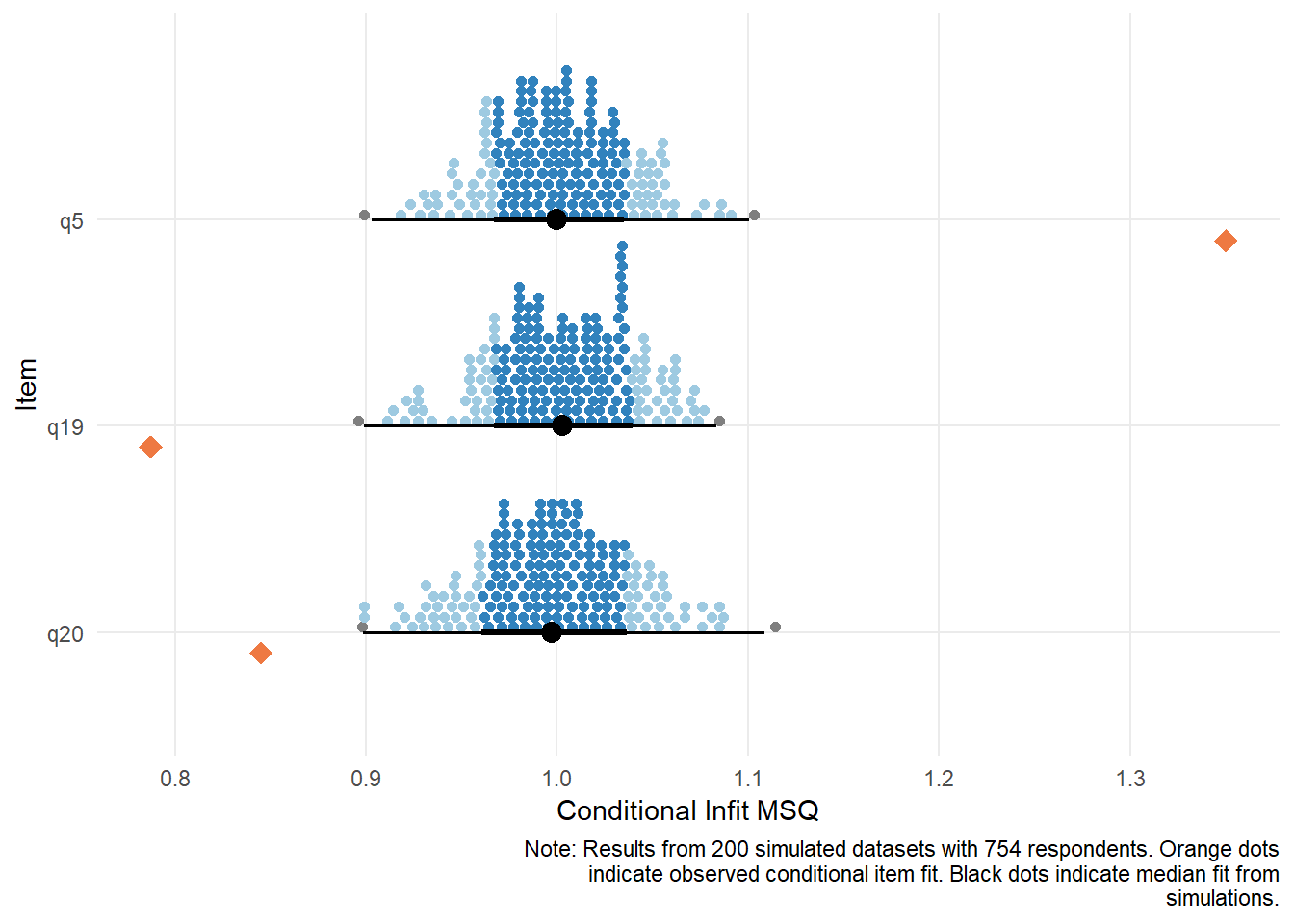

Supplement: Supplementary file 1 — Supplementary Material 1 [file 41598_2025_28073_MOESM1_ESM.zip › Supplementary/analysis_av_files/figure-html/unnamed-chunk-58-1.png]

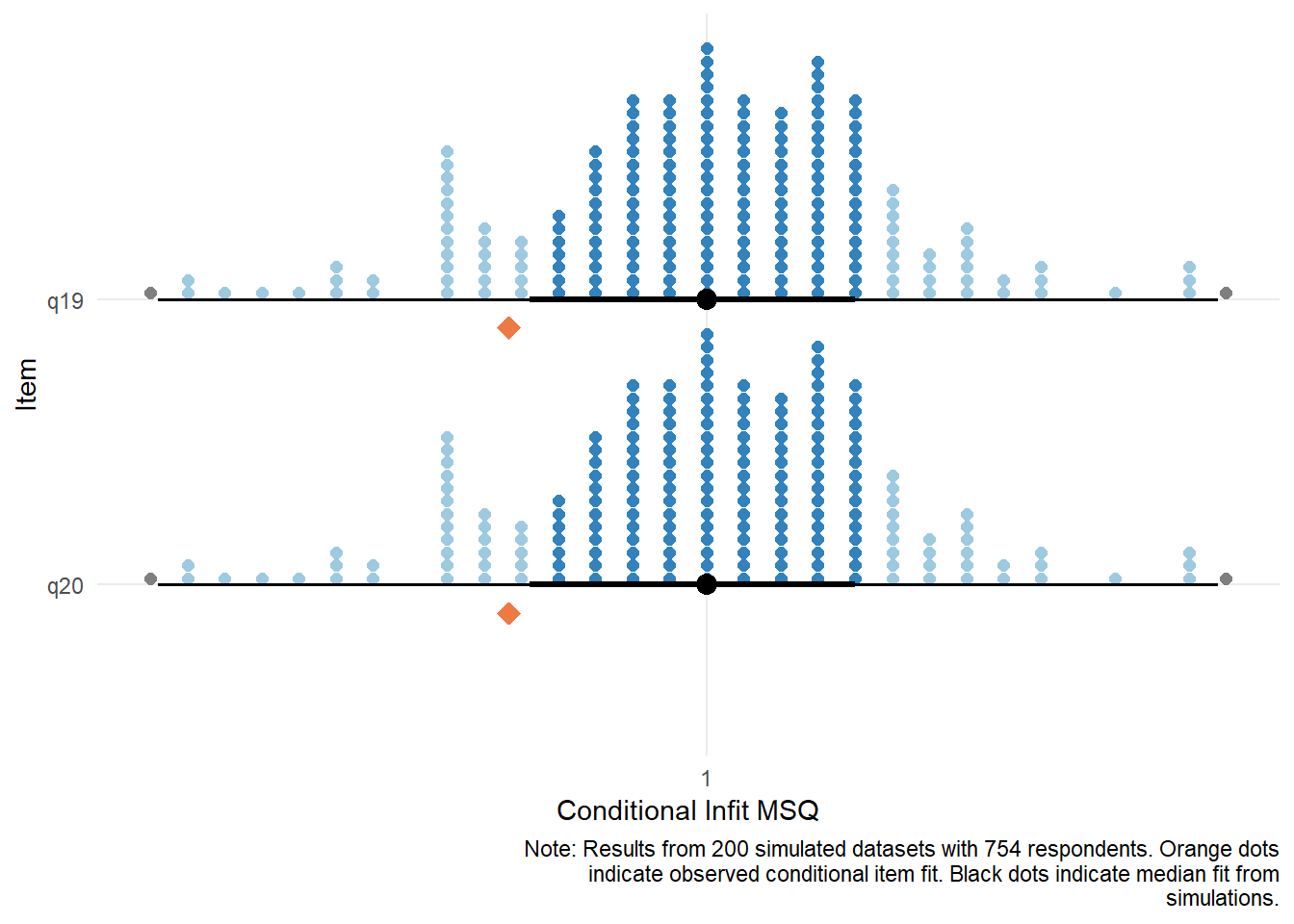

Supplement: Supplementary file 1 — Supplementary Material 1 [file 41598_2025_28073_MOESM1_ESM.zip › Supplementary/analysis_av_files/figure-html/unnamed-chunk-60-1.png]

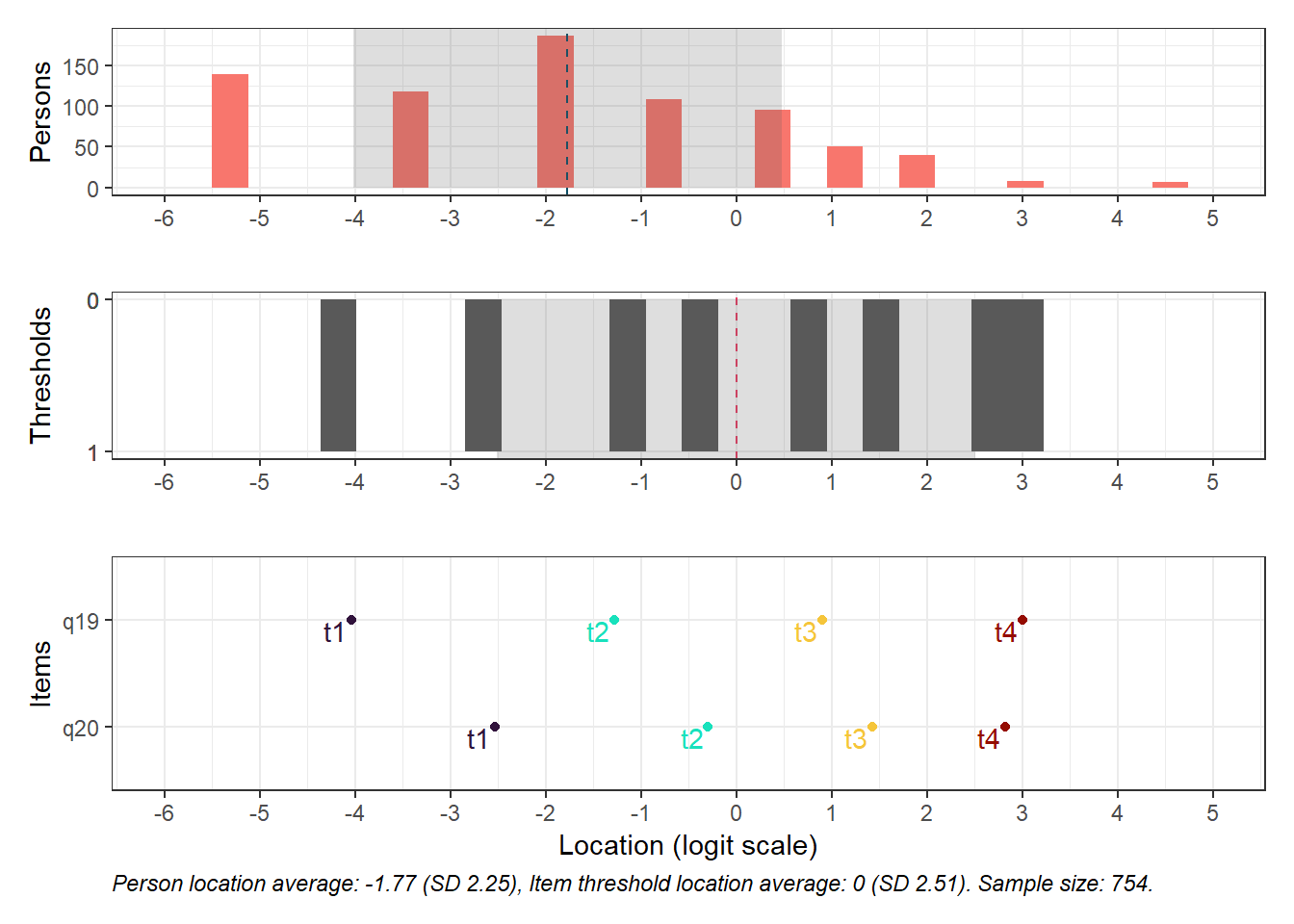

Supplement: Supplementary file 1 — Supplementary Material 1 [file 41598_2025_28073_MOESM1_ESM.zip › Supplementary/analysis_av_files/figure-html/unnamed-chunk-61-1.png]

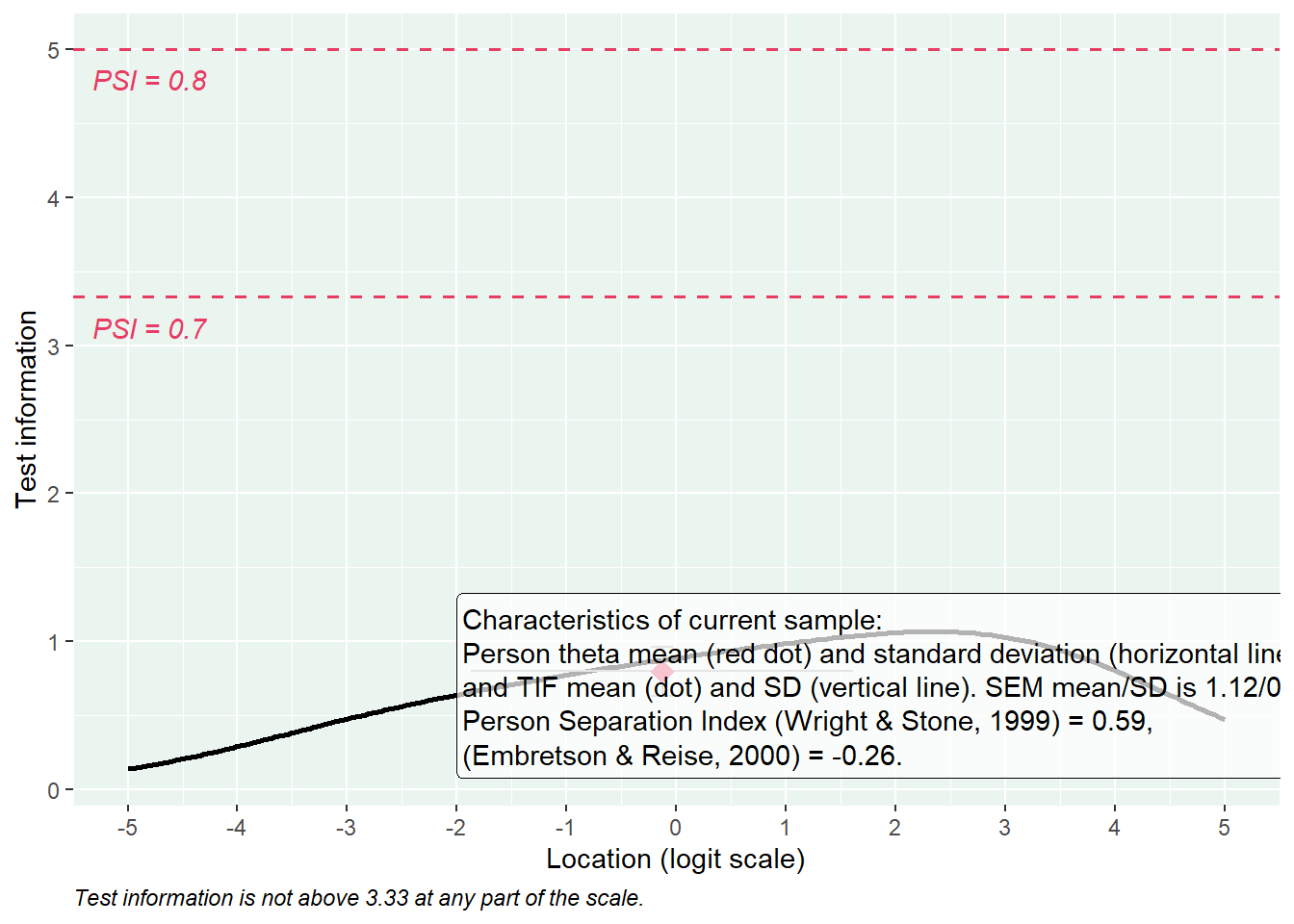

Supplement: Supplementary file 1 — Supplementary Material 1 [file 41598_2025_28073_MOESM1_ESM.zip › Supplementary/analysis_av_files/figure-html/unnamed-chunk-62-1.png]

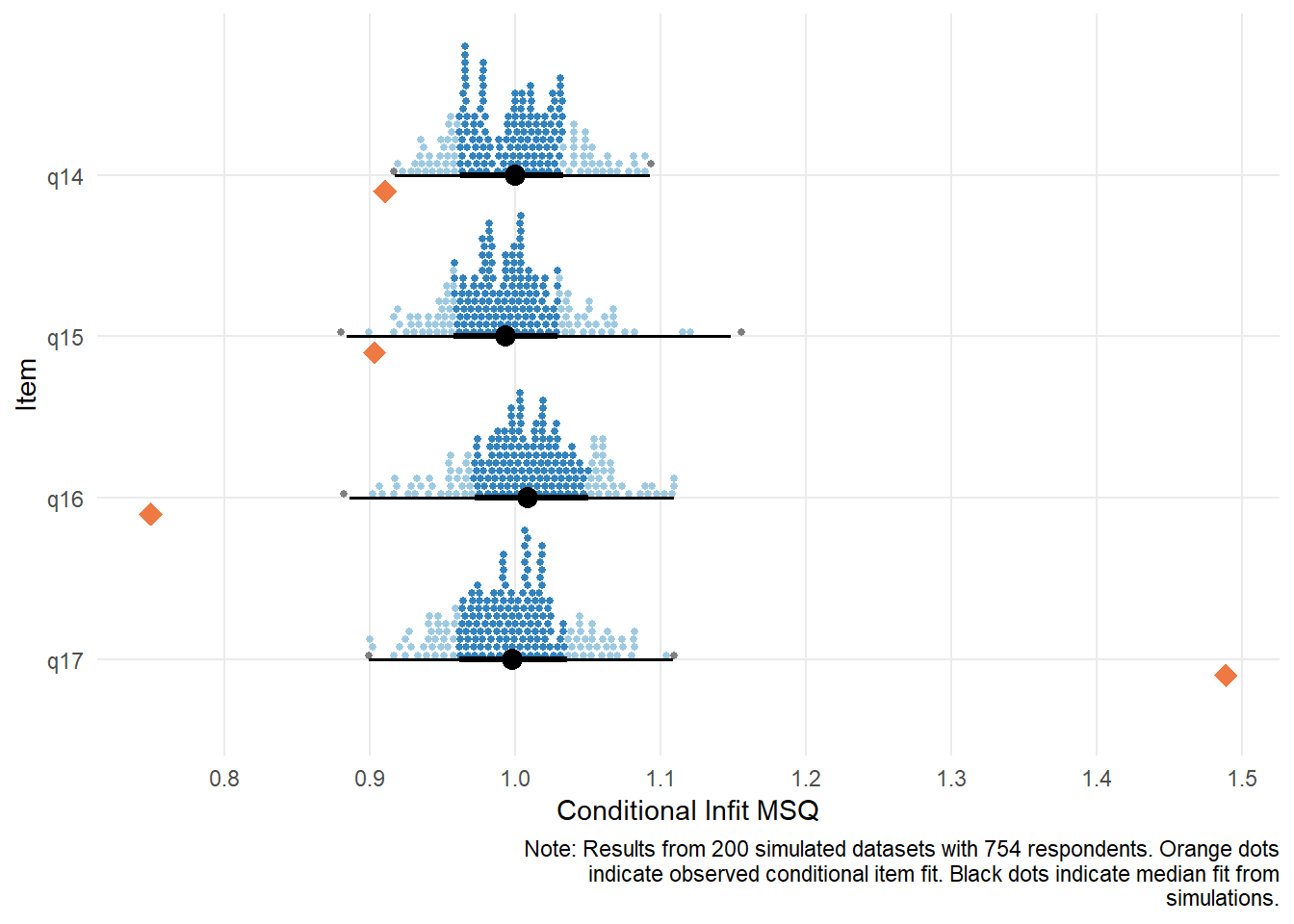

Supplement: Supplementary file 1 — Supplementary Material 1 [file 41598_2025_28073_MOESM1_ESM.zip › Supplementary/analysis_drag_files/figure-html/unnamed-chunk-15-1.png]

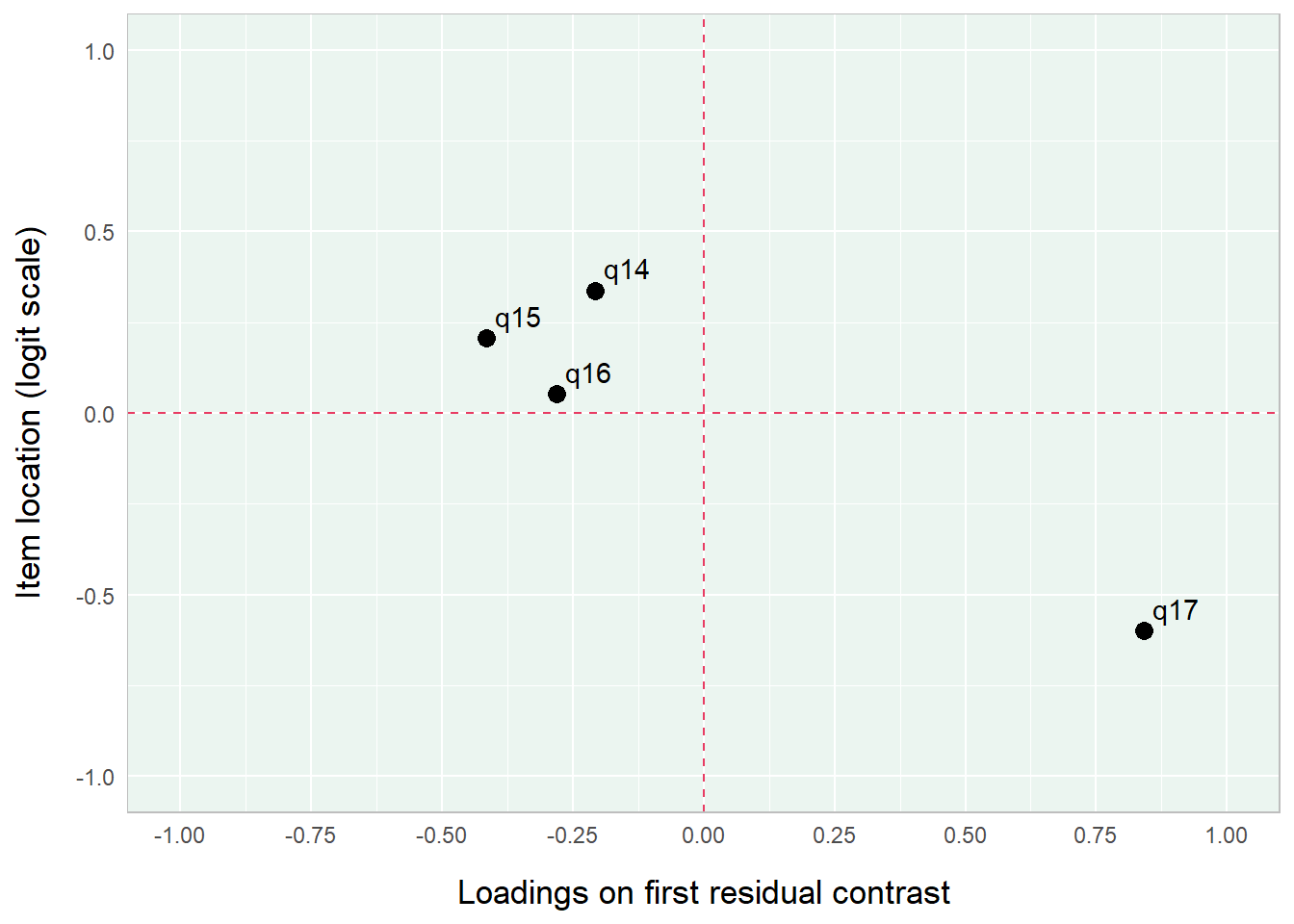

Supplement: Supplementary file 1 — Supplementary Material 1 [file 41598_2025_28073_MOESM1_ESM.zip › Supplementary/analysis_drag_files/figure-html/unnamed-chunk-20-1.png]

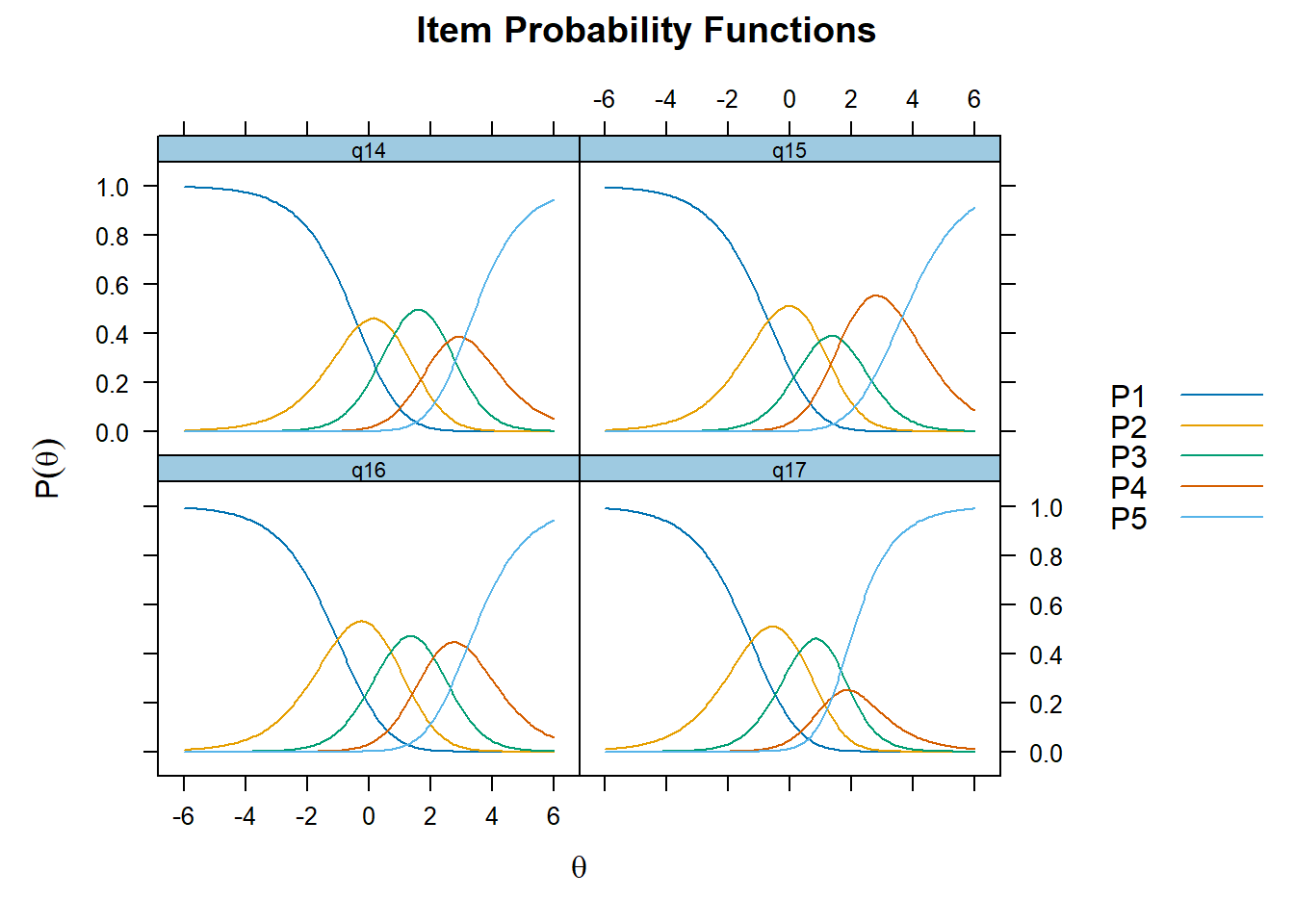

Supplement: Supplementary file 1 — Supplementary Material 1 [file 41598_2025_28073_MOESM1_ESM.zip › Supplementary/analysis_drag_files/figure-html/unnamed-chunk-21-1.png]

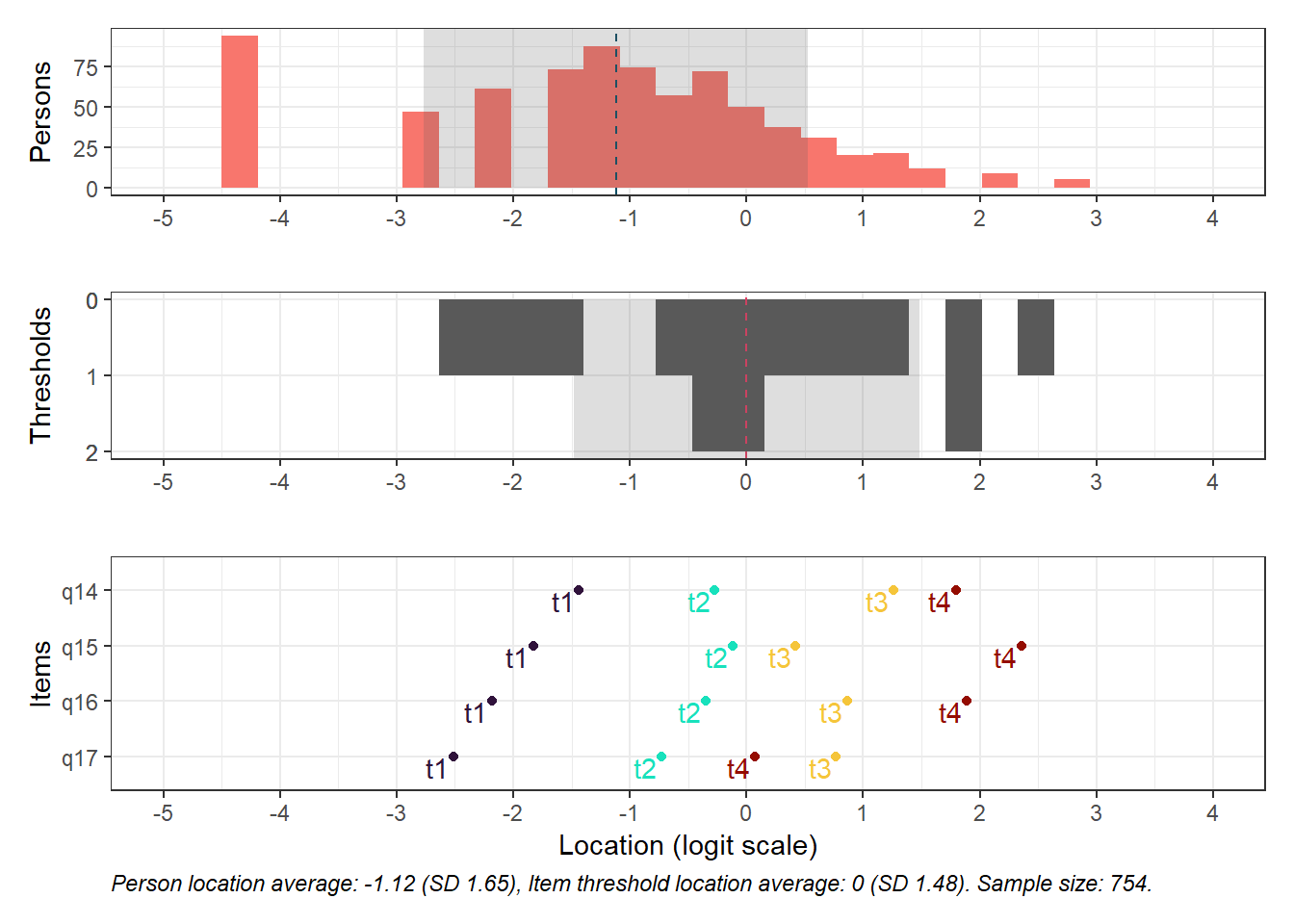

Supplement: Supplementary file 1 — Supplementary Material 1 [file 41598_2025_28073_MOESM1_ESM.zip › Supplementary/analysis_drag_files/figure-html/unnamed-chunk-22-1.png]

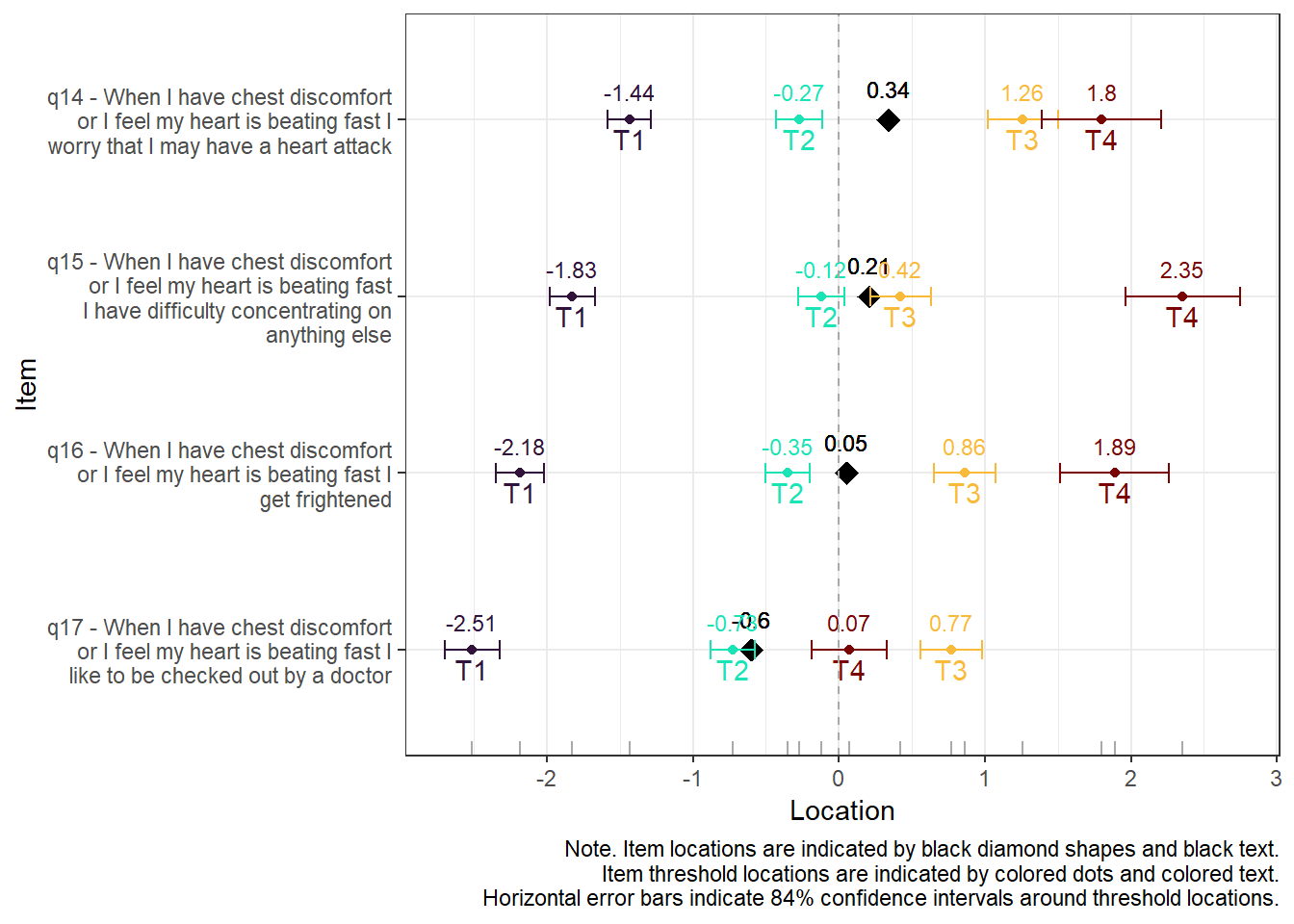

Supplement: Supplementary file 1 — Supplementary Material 1 [file 41598_2025_28073_MOESM1_ESM.zip › Supplementary/analysis_drag_files/figure-html/unnamed-chunk-23-1.png]

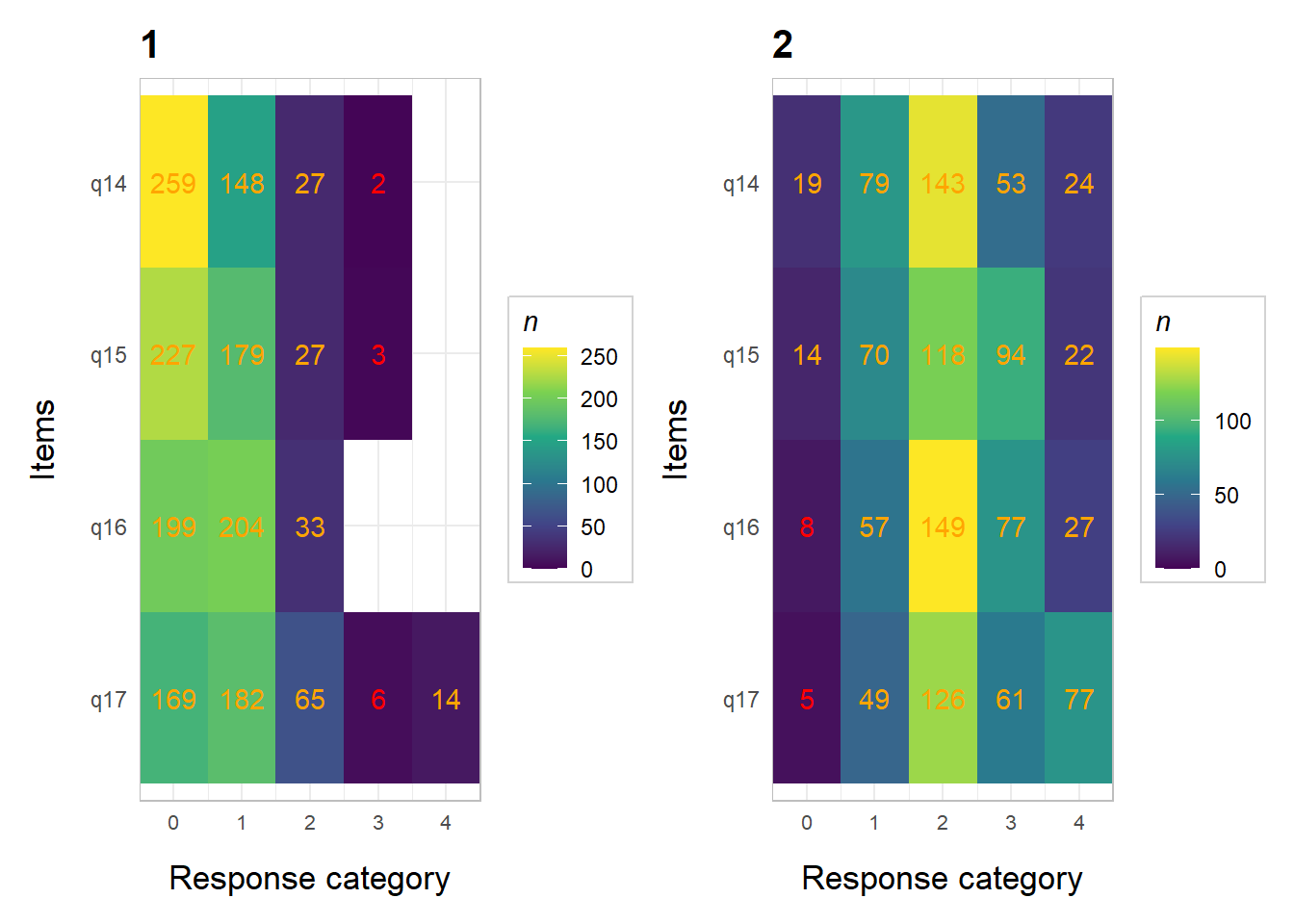

Supplement: Supplementary file 1 — Supplementary Material 1 [file 41598_2025_28073_MOESM1_ESM.zip › Supplementary/analysis_drag_files/figure-html/unnamed-chunk-24-1.png]

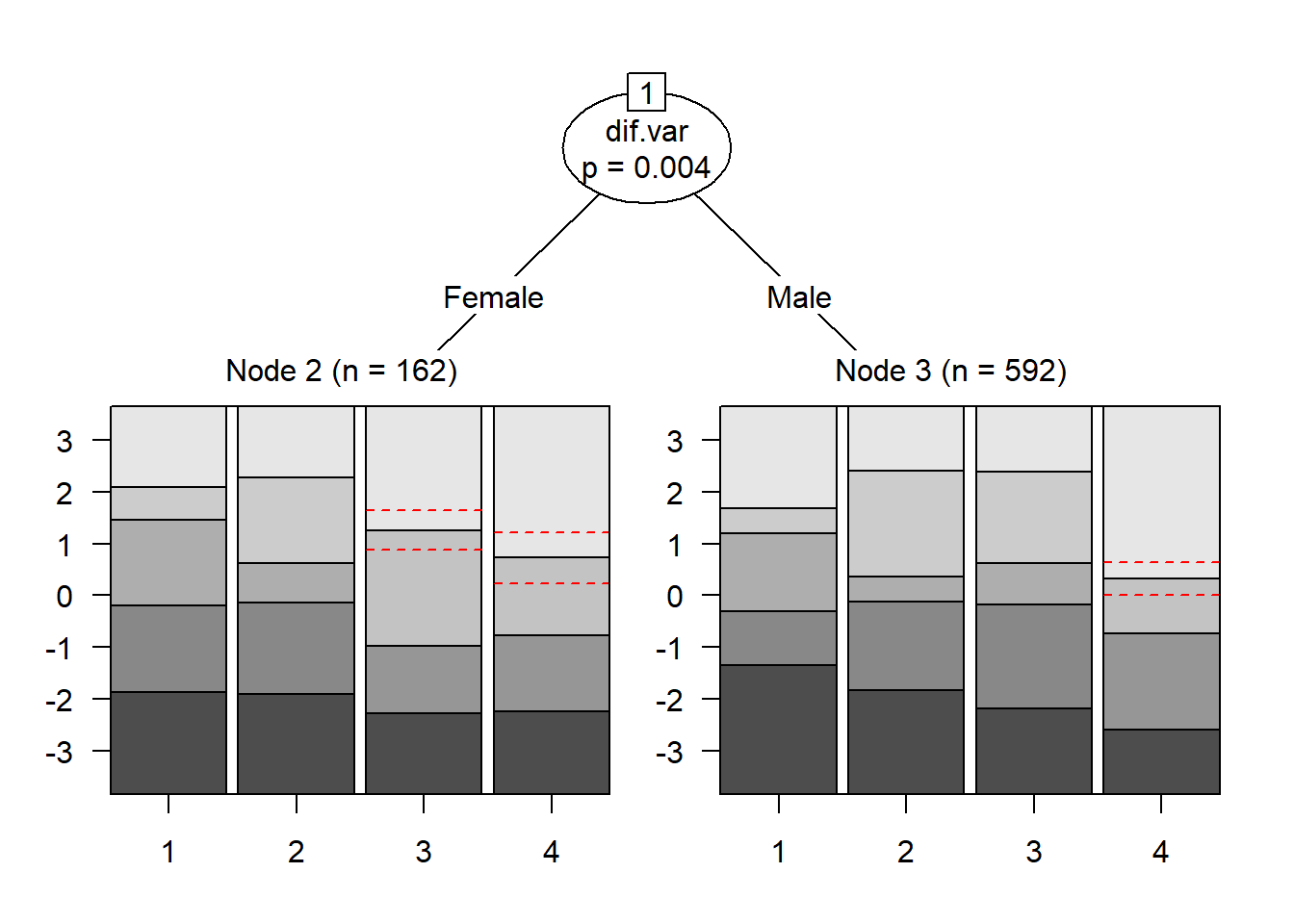

Supplement: Supplementary file 1 — Supplementary Material 1 [file 41598_2025_28073_MOESM1_ESM.zip › Supplementary/analysis_drag_files/figure-html/unnamed-chunk-26-1.png]
