# Supplementary material for: Psychometric properties of the Swedish cardiac anxiety questionnaire: a Rasch analysis
Source: Sci Rep. 2025 Nov 24;15:41834. doi: 10.1038/s41598-025-28073-8 (PMC12647126; doi:10.1038/s41598-025-28073-8)
Supplement: Supplementary file 1 — Supplementary Material 1 [file 41598_2025_28073_MOESM1_ESM.zip › Supplementary/analysis_f.html]

CAQ psychometric analysis, fear subscale


# CAQ psychometric analysis, fear subscale

 Code

- Show All Code
- Hide All Code
- ---
- View Source

Using Rasch Measurement Theory

Author

Affiliation

Magnus Johansson; Philip Leissner

RISE Research Institutes of Sweden; Department of women’s and children’s health, Uppsala University

## Table of contents

- 1 All items in the analysis
- 2 Descriptives - item level
- 3 Fear subscale
  - 3.1 Sumscore
  - 3.2 Rasch analysis
  - 3.3 Fear without q11 och q18
  - 3.4 Fear without q11, q17 och q18
  - 3.5 Fear without q11, q13, q17 och q18
  - 3.6 Fear without q10, q11, q13, q17 och q18
  - 3.7 Test Information (Reliability)

## Other Formats

- PDF

Code

```
# one package below requires that you use devtools to install them manually:
# first install devtools by
# install.packages('devtools')

library(easyRasch) # devtools::install_github("pgmj/easyRasch")
library(grateful)
library(ggrepel)
library(car)
library(kableExtra)
library(readxl)
library(tidyverse)
library(eRm)
library(iarm)
library(mirt)
library(psych)
library(psychotree)
library(matrixStats)
library(reshape)
library(knitr)
library(patchwork)
library(formattable) 
library(glue)
library(readxl) # for reading excel files

### optional libraries
#library(TAM)
#library(skimr)
#library(janitor)

### some commands exist in multiple packages, here we define preferred ones that are frequently used
select <- dplyr::select
count <- dplyr::count
recode <- car::recode
rename <- dplyr::rename
```

Code

```
### import data - this is just sample code, the files do not exist
df <- read_excel("data/CAQ_Rasch.xlsx") # replace with your datafile as needed

#library(haven) # for SPSS and other formats
#library(labelled) # for getting labels and metadata from SPSS files

### Load item information
# make sure that variable names in df match with itemlabels$itemnr
iteminfo <- read_excel("data/iteminfo.xlsx")

itemlabels <- iteminfo[,1:2]

### Make a backup of the dataframe, in case you need to revert changes at some point
d <- df
```

Code

```
##### Optionally: filter participants based on missing data

##### Before filtering out participants, you should check the missing data structure using RImissing() and RImissingP()

RImissing(d)
```

RImissingP() needs to be addressed, so we’ll do a manual check:

Code

```
d[,6:23] %>% 
  mutate(missing = rowSums(is.na(.))) %>% 
  count(missing)
```

```
# A tibble: 10 × 2
   missing     n
     <dbl> <int>
 1       0   756
 2       1    28
 3       2     8
 4       3     1
 5       4     3
 6       5     2
 7       9     1
 8      10     1
 9      16     1
10      18     5
```

We lose 50 respondents if we remove everyone with a missing value on any item. 756 is a good sample size, so we’ll go with that.

Code

```
d <- na.omit(d)
```

2 who were missing on some demographic variable were also removed.

Code

```
#---- Create DIF variables----
  
# DIF variables into vectors, recoded as factors since DIF functions need this
# these could also be stored in its own dataframe (not a tibble) instead of as vectors

d_dif <- d %>% 
  mutate(sex = factor(SEX),
         age = AGE_R,
         rel_status = factor(RELSTAT_bin),
         born_swe = factor(BORNSWE),
         edu = factor(EDUCATION)) %>% 
  select(sex,age,rel_status,born_swe,edu)

# remove DIF variables from item data
d <- d %>% 
  select(starts_with("Item")) %>% 
  set_names(itemlabels$itemnr)


### label sex variable as factor
# dif.sex <- factor(dif.sex,
#                       levels = c(1,2,3),
#                       labels = c("Female", "Male", "Other/missing response"))

# optionally, load RISE ggplot theme and color palettes and set the theme as default.
# just comment out the row below if you desire different theming
source("RISE_theme.R")
```

## 1 All items in the analysis

Code

```
RIlistitems(d)
```

| itemnr | item |
| --- | --- |
| q1 | I pay attention to my heart beat |
| q2 | I avoid physical exertion |
| q3 | My racing heart wakes me up at night |
| q4 | Chest pain/discomfort wakes me up at night |
| q5 | I take it easy as much as possible |
| q6 | I check my pulse |
| q7 | I avoid exercise or other physical work |
| q8 | I can feel my heart in my chest |
| q9 | I avoid activities that make my heart beat faster |
| q10 | If tests come out normal, I still worry about my heart |
| q11 | I feel safe being around a hospital, physician, or other medical facility |
| q12 | I avoid activities that make me sweat |
| q13 | I worry that doctors do not believe my chest pain/discomfort is real |
| q14 | When I have chest discomfort or I feel my heart is beating fast I worry that I may have a heart attack |
| q15 | When I have chest discomfort or I feel my heart is beating fast I have difficulty concentrating on anything else |
| q16 | When I have chest discomfort or I feel my heart is beating fast I get frightened |
| q17 | When I have chest discomfort or I feel my heart is beating fast I like to be checked out by a doctor |
| q18 | When I have chest discomfort or I feel my heart is beating fast I tell my family or friends |

Response distribution for all items are summarized below.

Code

```
RIallresp(d)
```

| Response category | Number of responses | Percent |
| --- | --- | --- |
| 0 | 5359 | 39.5 |
| 1 | 3580 | 26.4 |
| 2 | 2767 | 20.4 |
| 3 | 1244 | 9.2 |
| 4 | 622 | 4.6 |

## 2 Descriptives - item level

Code

```
RIlistItemsMargin(d, fontsize = 12)
```

| itemnr | item |
| --- | --- |
| q1 | I pay attention to my heart beat |
| q2 | I avoid physical exertion |
| q3 | My racing heart wakes me up at night |
| q4 | Chest pain/discomfort wakes me up at night |
| q5 | I take it easy as much as possible |
| q6 | I check my pulse |
| q7 | I avoid exercise or other physical work |
| q8 | I can feel my heart in my chest |
| q9 | I avoid activities that make my heart beat faster |
| q10 | If tests come out normal, I still worry about my heart |
| q11 | I feel safe being around a hospital, physician, or other medical facility |
| q12 | I avoid activities that make me sweat |
| q13 | I worry that doctors do not believe my chest pain/discomfort is real |
| q14 | When I have chest discomfort or I feel my heart is beating fast I worry that I may have a heart attack |
| q15 | When I have chest discomfort or I feel my heart is beating fast I have difficulty concentrating on anything else |
| q16 | When I have chest discomfort or I feel my heart is beating fast I get frightened |
| q17 | When I have chest discomfort or I feel my heart is beating fast I like to be checked out by a doctor |
| q18 | When I have chest discomfort or I feel my heart is beating fast I tell my family or friends |

- Tile plot
- Stacked bars
- Barplots

Code

```
RItileplot(d)
```

Code

```
RIbarstack(d) + scale_fill_viridis_d(labels = c("Always","Often","Sometimes","Rarely","Never"), direction = -1)
```

Code

```
RIbarplot(d)
```

Very few individuals endorse the highest categories of items q3, q4, q10, and q13. Over half of the participants endorse the lowest category for items q3, q4, and q13.

## 3 Fear subscale

Code

```
d_all <- d

items_f <- iteminfo %>% 
  filter(factor == "fear") %>% 
  pull(itemnr)

d <- d_all %>% 
  select(all_of(items_f))
```

### 3.1 Sumscore

Code

```
d_sum <- d %>%
  mutate(total = rowSums(select(., starts_with("q")), na.rm = TRUE))
d_sum <- d_sum["total"]
```

Code

```
total_freq <- as.data.frame(table(d_sum$total))
colnames(total_freq) <- c("Total", "N")
total_freq$Percent <- round(100 * total_freq$N / sum(total_freq$N), 1)
```

Code

```
RIallresp(d_sum)
```

| Response category | Number of responses | Percent |
| --- | --- | --- |
| 0 | 34 | 4.5 |
| 1 | 25 | 3.3 |
| 2 | 26 | 3.4 |
| 3 | 33 | 4.4 |
| 4 | 40 | 5.3 |
| 5 | 43 | 5.7 |
| 6 | 39 | 5.2 |
| 7 | 39 | 5.2 |
| 8 | 54 | 7.2 |
| 9 | 42 | 5.6 |
| 10 | 43 | 5.7 |
| 11 | 46 | 6.1 |
| 12 | 44 | 5.8 |
| 13 | 40 | 5.3 |
| 14 | 41 | 5.4 |
| 15 | 39 | 5.2 |
| 16 | 20 | 2.7 |
| 17 | 20 | 2.7 |
| 18 | 18 | 2.4 |
| 19 | 12 | 1.6 |
| 20 | 19 | 2.5 |
| 21 | 5 | 0.7 |
| 22 | 5 | 0.7 |
| 23 | 9 | 1.2 |
| 24 | 5 | 0.7 |
| 25 | 6 | 0.8 |
| 26 | 2 | 0.3 |
| 27 | 3 | 0.4 |
| 28 | 1 | 0.1 |
| 32 | 1 | 0.1 |

### 3.2 Rasch analysis

The eRm package, which uses Conditional Maximum Likelihood (CML) estimation, will be used primarily. For this analysis, the Partial Credit Model will be used.

| itemnr | item |
| --- | --- |
| q10 | If tests come out normal, I still worry about my heart |
| q11 | I feel safe being around a hospital, physician, or other medical facility |
| q13 | I worry that doctors do not believe my chest pain/discomfort is real |
| q14 | When I have chest discomfort or I feel my heart is beating fast I worry that I may have a heart attack |
| q15 | When I have chest discomfort or I feel my heart is beating fast I have difficulty concentrating on anything else |
| q16 | When I have chest discomfort or I feel my heart is beating fast I get frightened |
| q17 | When I have chest discomfort or I feel my heart is beating fast I like to be checked out by a doctor |
| q18 | When I have chest discomfort or I feel my heart is beating fast I tell my family or friends |

- Conditional item fit
- Item-restscore
- Conditional LRT
- Local dependency
- Residual correlations
- 1st contrast loadings
- Response categories
- Targeting
- Item hierarchy
- Score groups LR-test
- Score groups obs-exp
- Rasch-tree DIF immigration status
- Rasch-tree DIF sex
- Rasch-tree DIF rel
- Rasch-tree DIF age
- Rasch-tree DIF rel\*age
- Person fit
- Floor and ceiling effects

Code

```
#RIitemfit(d, cutoff = "Smith98")

simfit1 <- RIgetfit(d, iterations = 200, cpu = 8) 

RIitemfit(d, simfit1)
```

| Item | InfitMSQ | Infit thresholds | OutfitMSQ | Outfit thresholds | Infit diff | Outfit diff | Relative location |
| --- | --- | --- | --- | --- | --- | --- | --- |
| q10 | 0.951 | [0.911, 1.121] | 0.995 | [0.866, 1.261] | no misfit | no misfit | 1.62 |
| q11 | 1.386 | [0.828, 1.126] | 1.675 | [0.833, 1.165] | 0.26 | 0.51 | 0.33 |
| q13 | 1.211 | [0.86, 1.121] | 1.33 | [0.817, 1.219] | 0.09 | 0.111 | 1.73 |
| q14 | 0.754 | [0.904, 1.119] | 0.72 | [0.886, 1.171] | 0.15 | 0.166 | 1.04 |
| q15 | 0.832 | [0.893, 1.105] | 0.818 | [0.868, 1.12] | 0.061 | 0.05 | 0.98 |
| q16 | 0.722 | [0.914, 1.097] | 0.719 | [0.887, 1.113] | 0.192 | 0.168 | 0.86 |
| q17 | 0.806 | [0.886, 1.127] | 0.797 | [0.891, 1.199] | 0.08 | 0.094 | 0.38 |
| q18 | 1.418 | [0.923, 1.104] | 1.593 | [0.907, 1.117] | 0.314 | 0.476 | 0.09 |
|  |  |  |  |  |  |  |  |
| --- | --- | --- | --- | --- | --- | --- | --- |
| Note: |  |  |  |  |  |  |  |
| MSQ values based on conditional calculations (n = 754 complete cases).  Simulation based thresholds from 200 simulated datasets. |  |  |  |  |  |  |  |

Code

```
RIgetfitPlot(simfit1, d)
```

Code

```
RIrestscore(d)
```

| Item | Observed value | Model expected value | Absolute difference | Adjusted p-value (BH) | Statistical significance level | Location | Relative location |
| --- | --- | --- | --- | --- | --- | --- | --- |
| q10 | 0.50 | 0.47 | 0.03 | 0.301 |  | 0.74 | 1.62 |
| q11 | 0.40 | 0.54 | 0.14 | 0.000 | \*\*\* | -0.54 | 0.33 |
| q13 | 0.36 | 0.48 | 0.12 | 0.002 | \*\* | 0.85 | 1.73 |
| q14 | 0.64 | 0.49 | 0.15 | 0.000 | \*\*\* | 0.16 | 1.04 |
| q15 | 0.59 | 0.50 | 0.09 | 0.000 | \*\*\* | 0.10 | 0.98 |
| q16 | 0.66 | 0.49 | 0.17 | 0.000 | \*\*\* | -0.02 | 0.86 |
| q17 | 0.64 | 0.52 | 0.12 | 0.000 | \*\*\* | -0.50 | 0.38 |
| q18 | 0.36 | 0.52 | 0.16 | 0.000 | \*\*\* | -0.79 | 0.09 |

Code

```
clr_tests(d, model = "PCM")
```

```
Conditional Likelihood Ratio Tests:
```

```
        clr df pvalue sig 
overall 162 31 0       ***
```

Code

```
RIbootLRT(d, iterations = 1000, samplesize = 400, cpu = 8)
```

| Result | n | Percent |
| --- | --- | --- |
| Statistically significant | 1000 | 100 |

Code

```
# using partial gamma LD from library(iarm)
RIpartgamLD(d)
```

| Item 1 | Item 2 | Partial gamma | SE | Lower CI | Upper CI | Adjusted p-value (BH) |
| --- | --- | --- | --- | --- | --- | --- |
| q16 | q15 | 0.62 | 0.044 | 0.533 | 0.707 | 0.000 |
| q15 | q16 | 0.578 | 0.048 | 0.485 | 0.672 | 0.000 |
| q17 | q18 | 0.505 | 0.042 | 0.421 | 0.588 | 0.000 |
| q14 | q15 | 0.43 | 0.054 | 0.324 | 0.536 | 0.000 |
| q16 | q14 | 0.418 | 0.056 | 0.308 | 0.528 | 0.000 |
| q15 | q14 | 0.404 | 0.056 | 0.294 | 0.514 | 0.000 |
| q14 | q16 | 0.383 | 0.057 | 0.270 | 0.495 | 0.000 |
| q18 | q17 | 0.32 | 0.048 | 0.225 | 0.414 | 0.000 |
| q10 | q13 | 0.298 | 0.066 | 0.168 | 0.428 | 0.000 |
| q16 | q10 | 0.279 | 0.065 | 0.152 | 0.406 | 0.001 |
| q14 | q10 | 0.261 | 0.063 | 0.138 | 0.384 | 0.002 |
| q13 | q10 | 0.245 | 0.067 | 0.115 | 0.376 | 0.013 |
| q17 | q11 | 0.196 | 0.054 | 0.090 | 0.301 | 0.015 |

Code

```
simcor1 <- RIgetResidCor(d, iterations = 250, cpu = 8)
RIresidcorr(d, cutoff = simcor1$p99)
```

|  | q10 | q11 | q13 | q14 | q15 | q16 | q17 | q18 |
| --- | --- | --- | --- | --- | --- | --- | --- | --- |
| q10 |  |  |  |  |  |  |  |  |
| q11 | -0.15 |  |  |  |  |  |  |  |
| q13 | 0.08 | -0.18 |  |  |  |  |  |  |
| q14 | 0.03 | -0.27 | -0.02 |  |  |  |  |  |
| q15 | -0.04 | -0.34 | -0.04 | 0.19 |  |  |  |  |
| q16 | -0.01 | -0.33 | -0.1 | 0.21 | 0.38 |  |  |  |
| q17 | -0.26 | -0.09 | -0.2 | -0.15 | -0.21 | -0.1 |  |  |
| q18 | -0.23 | -0.13 | -0.3 | -0.31 | -0.33 | -0.3 | 0.18 |  |
|  |  |  |  |  |  |  |  |  |
| --- | --- | --- | --- | --- | --- | --- | --- | --- |
| Note: |  |  |  |  |  |  |  |  |
| Relative cut-off value is 0.031, which is 0.14 above the average correlation (-0.108).  Correlations above the cut-off are highlighted in red text. |  |  |  |  |  |  |  |  |

Code

```
RIloadLoc(d)
```

Code

```
mirt(d, model=1, itemtype='Rasch', verbose = FALSE) %>% 
  plot(type="trace", as.table = TRUE, 
       theta_lim = c(-6,6))
```

Code

```
# for fewer items or a more magnified figure, use:
#RIitemCats(d)
```

Code

```
# increase fig-height above as needed, if you have many items
RItargeting(d)
```

Code

```
RIitemHierarchy(d)
```

Code

```
iarm::score_groups(as.data.frame(d)) %>% 
  as.data.frame(nm = "score_group") %>% 
  dplyr::count(score_group)
```

```
  score_group   n
1           1 375
2           2 379
```

Code

```
dif_plots <- d %>% 
  add_column(dif = iarm::score_groups(.)) %>% 
  split(.$dif) %>% # split the data using the DIF variable
  map(~ RItileplot(.x %>% dplyr::select(!dif)) + labs(title = .x$dif))
dif_plots[[1]] + dif_plots[[2]]
```

Code

```
clr_tests(d, model = "PCM")
```

```
Conditional Likelihood Ratio Tests:
```

```
        clr df pvalue sig 
overall 162 31 0       ***
```

Code

```
item_obsexp(PCM(d))
```

```
Score group 1: 
    mean obs mean exp std.res sig
q10  0.264    0.285   -0.768     
q11  0.944    0.777    3.510  ++ 
q13  0.255    0.213    1.627     
q14  0.496    0.591   -2.579  -  
q15  0.642    0.685   -1.104     
q16  0.666    0.779   -2.946  -  
q17  0.795    0.928   -3.168  -- 
q18  1.416    1.200    4.678  ++ 

Score group 2: 
    mean obs mean exp std.res sig
q10  1.050    1.024    0.585     
q11  2.492    2.643   -2.592  -  
q13  0.870    0.908   -0.834     
q14  1.733    1.642    1.845     
q15  1.839    1.800    0.776     
q16  1.939    1.831    2.268  +  
q17  2.389    2.269    2.227  +  
q18  2.471    2.667   -3.755  --
```

Code

```
RIdifTable(d, d_dif$born_swe)
```

| Item | 2 | 3 | Mean location | StDev | MaxDiff |
| --- | --- | --- | --- | --- | --- |
| q10 | 0.514 | 0.785 | 0.650 | 0.192 | 0.272 |
| q11 | -0.736 | -0.537 | -0.636 | 0.140 | 0.198 |
| q13 | 0.804 | 0.849 | 0.826 | 0.032 | 0.045 |
| q14 | 0.021 | 0.208 | 0.114 | 0.132 | 0.187 |
| q15 | 0.377 | 0.054 | 0.216 | 0.228 | 0.323 |
| q16 | 0.030 | -0.032 | -0.001 | 0.044 | 0.062 |
| q17 | -0.449 | -0.505 | -0.477 | 0.040 | 0.056 |
| q18 | -0.561 | -0.822 | -0.691 | 0.185 | 0.261 |

Code

```
RIdifTable(d, d_dif$sex)
```

| Item | 2 | 3 | Mean location | StDev | MaxDiff |
| --- | --- | --- | --- | --- | --- |
| q10 | 0.880 | 0.687 | 0.783 | 0.136 | 0.193 |
| q11 | -0.452 | -0.593 | -0.522 | 0.099 | 0.140 |
| q13 | 0.720 | 0.900 | 0.810 | 0.127 | 0.180 |
| q14 | 0.059 | 0.184 | 0.121 | 0.088 | 0.125 |
| q15 | -0.015 | 0.141 | 0.063 | 0.110 | 0.156 |
| q16 | -0.286 | 0.116 | -0.085 | 0.284 | 0.402 |
| q17 | -0.414 | -0.544 | -0.479 | 0.092 | 0.131 |
| q18 | -0.492 | -0.890 | -0.691 | 0.282 | 0.398 |

Code

```
RIdifTable(d, d_dif$rel_status)
```

| Item | 2 | 3 | Mean location | StDev | MaxDiff |
| --- | --- | --- | --- | --- | --- |
| q10 | 0.756 | 0.684 | 0.720 | 0.051 | 0.073 |
| q11 | -0.535 | -0.608 | -0.571 | 0.052 | 0.073 |
| q13 | 0.945 | 0.586 | 0.765 | 0.254 | 0.359 |
| q14 | 0.187 | 0.055 | 0.121 | 0.094 | 0.132 |
| q15 | 0.105 | 0.053 | 0.079 | 0.037 | 0.053 |
| q16 | -0.010 | -0.081 | -0.045 | 0.050 | 0.071 |
| q17 | -0.570 | -0.231 | -0.401 | 0.240 | 0.339 |
| q18 | -0.878 | -0.457 | -0.668 | 0.298 | 0.421 |

Code

```
RIdifTable(d, d_dif$age)
```

| Item | 2 | 3 | Mean location | StDev | MaxDiff |
| --- | --- | --- | --- | --- | --- |
| q10 | 0.692 | 0.847 | 0.769 | 0.110 | 0.155 |
| q11 | -0.552 | -0.556 | -0.554 | 0.003 | 0.005 |
| q13 | 0.842 | 0.883 | 0.862 | 0.029 | 0.041 |
| q14 | 0.166 | 0.152 | 0.159 | 0.010 | 0.014 |
| q15 | 0.011 | 0.277 | 0.144 | 0.188 | 0.266 |
| q16 | -0.094 | 0.114 | 0.010 | 0.147 | 0.208 |
| q17 | -0.390 | -0.701 | -0.546 | 0.219 | 0.310 |
| q18 | -0.675 | -1.015 | -0.845 | 0.240 | 0.340 |

Code

```
RIdifTable2(d, d_dif$rel_status, d_dif$sex)
```

| Item | 2 | 4 | 5 | Mean location | StDev | MaxDiff |
| --- | --- | --- | --- | --- | --- | --- |
| q10 | 0.880 | 0.687 | 0.629 | 0.732 | 0.131 | 0.251 |
| q11 | -0.452 | -0.588 | -0.714 | -0.584 | 0.131 | 0.261 |
| q13 | 0.720 | 1.025 | 0.549 | 0.765 | 0.241 | 0.477 |
| q14 | 0.059 | 0.233 | -0.123 | 0.056 | 0.178 | 0.356 |
| q15 | -0.015 | 0.123 | 0.228 | 0.112 | 0.122 | 0.243 |
| q16 | -0.286 | 0.097 | 0.170 | -0.006 | 0.245 | 0.456 |
| q17 | -0.414 | -0.599 | -0.312 | -0.442 | 0.145 | 0.287 |
| q18 | -0.492 | -0.978 | -0.427 | -0.632 | 0.301 | 0.551 |

Code

```
RIpfit(d)
```

Code

```
RItif(d, samplePSI = T, cutoff = 1)
```

There are quite a few issues with item fit here. Particularly items q11, q13, and q18, which are underfit, while items q14 and q16 appear to be heavily overfit.

There are residual correlations between items q10 and q13, q14 and q15, q14 and q15, q15 and q16, and q17 and q18.On the 1st contrast loadings items can bee seen to cluster into distinct groups.

All items except q15 and q16 have disordered response thresholds, with item q11 being the biggest problem.

Items q18 shows significant DIF for men that are in a relationship compared to men who are not in a relationship. Interesting finding that may be related to sex and gender based relationship tendencies.

The problems with item misfit and residual correlations indicate that the Fear subscale is not unidimensional. Several previous studies have divided this scale into two (Fear and Reassurance Seeking). The reassurance seeking scale traditionally consists of item q11, q17 and q18.

In a previous study, using exploratory factor analysis, items q10 and q13 were removed due to cross-loading. We may need to remove many items or split the scale, but as a starting point, we begin with items q11 and q18, as they have the most severe dimensionality issues.

### 3.3 Fear without q11 och q18

Code

```
d$q11 <- NULL
d$q18 <- NULL
```

| itemnr | item |
| --- | --- |
| q10 | If tests come out normal, I still worry about my heart |
| q13 | I worry that doctors do not believe my chest pain/discomfort is real |
| q14 | When I have chest discomfort or I feel my heart is beating fast I worry that I may have a heart attack |
| q15 | When I have chest discomfort or I feel my heart is beating fast I have difficulty concentrating on anything else |
| q16 | When I have chest discomfort or I feel my heart is beating fast I get frightened |
| q17 | When I have chest discomfort or I feel my heart is beating fast I like to be checked out by a doctor |

- Conditional item fit
- Item-restscore
- Residual correlations
- Local dependency
- 1st contrast loadings
- Score groups obs-exp
- Rasch-tree DIF relationship and sex

Code

```
simfit1 <- RIgetfit(d, iterations = 200, cpu = 8) 

RIitemfit(d, simfit1)
```

| Item | InfitMSQ | Infit thresholds | OutfitMSQ | Outfit thresholds | Infit diff | Outfit diff | Relative location |
| --- | --- | --- | --- | --- | --- | --- | --- |
| q10 | 1.083 | [0.88, 1.131] | 1.316 | [0.816, 1.188] | no misfit | 0.128 | 1.97 |
| q13 | 1.331 | [0.891, 1.127] | 1.519 | [0.775, 1.249] | 0.204 | 0.270 | 2.10 |
| q14 | 0.776 | [0.871, 1.107] | 0.769 | [0.859, 1.146] | 0.095 | 0.090 | 1.26 |
| q15 | 0.827 | [0.869, 1.094] | 0.828 | [0.861, 1.087] | 0.042 | 0.033 | 1.16 |
| q16 | 0.715 | [0.897, 1.106] | 0.728 | [0.896, 1.113] | 0.182 | 0.168 | 1.02 |
| q17 | 1.307 | [0.93, 1.126] | 1.394 | [0.925, 1.127] | 0.181 | 0.267 | 0.47 |
|  |  |  |  |  |  |  |  |
| --- | --- | --- | --- | --- | --- | --- | --- |
| Note: |  |  |  |  |  |  |  |
| MSQ values based on conditional calculations (n = 754 complete cases).  Simulation based thresholds from 200 simulated datasets. |  |  |  |  |  |  |  |

Code

```
RIgetfitPlot(simfit1, d)
```

Code

```
RIrestscore(d)
```

| Item | Observed value | Model expected value | Absolute difference | Adjusted p-value (BH) | Statistical significance level | Location | Relative location |
| --- | --- | --- | --- | --- | --- | --- | --- |
| q10 | 0.54 | 0.58 | 0.04 | 0.219 |  | 0.64 | 1.97 |
| q13 | 0.43 | 0.58 | 0.15 | 0.000 | \*\*\* | 0.77 | 2.10 |
| q14 | 0.71 | 0.60 | 0.11 | 0.000 | \*\*\* | -0.07 | 1.26 |
| q15 | 0.68 | 0.60 | 0.08 | 0.002 | \*\* | -0.17 | 1.16 |
| q16 | 0.74 | 0.60 | 0.14 | 0.000 | \*\*\* | -0.31 | 1.02 |
| q17 | 0.53 | 0.62 | 0.09 | 0.002 | \*\* | -0.86 | 0.47 |

Code

```
simcor1 <- RIgetResidCor(d, iterations = 250, cpu = 8)
RIresidcorr(d, cutoff = simcor1$p99)
```

|  | q10 | q13 | q14 | q15 | q16 | q17 |
| --- | --- | --- | --- | --- | --- | --- |
| q10 |  |  |  |  |  |  |
| q13 | -0.03 |  |  |  |  |  |
| q14 | -0.13 | -0.2 |  |  |  |  |
| q15 | -0.23 | -0.24 | -0.05 |  |  |  |
| q16 | -0.19 | -0.31 | -0.03 | 0.18 |  |  |
| q17 | -0.27 | -0.24 | -0.22 | -0.32 | -0.19 |  |
|  |  |  |  |  |  |  |
| --- | --- | --- | --- | --- | --- | --- |
| Note: |  |  |  |  |  |  |
| Relative cut-off value is -0.014, which is 0.151 above the average correlation (-0.165).  Correlations above the cut-off are highlighted in red text. |  |  |  |  |  |  |

Code

```
# using partial gamma LD from library(iarm)
RIpartgamLD(d)
```

| Item 1 | Item 2 | Partial gamma | SE | Lower CI | Upper CI | Adjusted p-value (BH) |
| --- | --- | --- | --- | --- | --- | --- |
| q16 | q15 | 0.532 | 0.054 | 0.426 | 0.638 | 0.000 |
| q15 | q16 | 0.44 | 0.059 | 0.325 | 0.556 | 0.000 |
| q14 | q15 | 0.284 | 0.065 | 0.156 | 0.412 | 0.000 |
| q16 | q14 | 0.239 | 0.067 | 0.108 | 0.369 | 0.010 |
| q15 | q14 | 0.237 | 0.066 | 0.108 | 0.367 | 0.010 |
| q10 | q13 | 0.223 | 0.070 | 0.085 | 0.361 | 0.045 |

Code

```
RIloadLoc(d)
```

Code

```
item_obsexp(PCM(d))
```

```
Score group 1: 
    mean obs mean exp std.res sig
q10  0.300    0.274    0.970     
q13  0.265    0.187    3.306  ++ 
q14  0.529    0.612   -2.402  -  
q15  0.688    0.714   -0.730     
q16  0.744    0.832   -2.521  -  
q17  1.094    0.997    2.625  +  

Score group 2: 
    mean obs mean exp std.res sig
q10  1.142    1.168   -0.567     
q13  0.967    1.046   -1.706     
q14  1.911    1.827    1.795     
q15  2.018    1.992    0.543     
q16  2.098    2.007    1.991     
q17  2.380    2.478   -1.915
```

Code

```
RIdifTable2(d, d_dif$rel_status, d_dif$sex)
```

| Item | 2 | 4 | 5 | Mean location | StDev | MaxDiff |
| --- | --- | --- | --- | --- | --- | --- |
| q10 | 0.972 | 0.502 | 0.563 | 0.679 | 0.255 | 0.470 |
| q13 | 0.735 | 0.863 | 0.508 | 0.702 | 0.180 | 0.355 |
| q14 | -0.138 | -0.021 | -0.369 | -0.176 | 0.177 | 0.348 |
| q15 | -0.246 | -0.164 | -0.002 | -0.137 | 0.124 | 0.244 |
| q16 | -0.584 | -0.201 | -0.091 | -0.292 | 0.259 | 0.493 |
| q17 | -0.739 | -0.979 | -0.610 | -0.776 | 0.187 | 0.369 |

Item q13 is still problematic in terms of dimensionality, but now is also item q17 underfit.

We’ll proceed by removing item q17 as it shows the most problems.

### 3.4 Fear without q11, q17 och q18

Code

```
d$q17 <- NULL
```

- Conditional item fit
- Item-restscore
- Residual correlations
- Local dependency
- Score groups obs-exp

Code

```
simfit1 <- RIgetfit(d, iterations = 200, cpu = 8) 

RIitemfit(d, simfit1)
```

| Item | InfitMSQ | Infit thresholds | OutfitMSQ | Outfit thresholds | Infit diff | Outfit diff | Relative location |
| --- | --- | --- | --- | --- | --- | --- | --- |
| q10 | 1.13 | [0.864, 1.145] | 1.271 | [0.821, 1.2] | no misfit | 0.071 | 2.10 |
| q13 | 1.399 | [0.86, 1.154] | 1.723 | [0.794, 1.317] | 0.245 | 0.406 | 2.24 |
| q14 | 0.847 | [0.879, 1.116] | 0.854 | [0.887, 1.127] | 0.032 | 0.033 | 1.34 |
| q15 | 0.848 | [0.887, 1.114] | 0.85 | [0.888, 1.131] | 0.039 | 0.038 | 1.22 |
| q16 | 0.787 | [0.895, 1.142] | 0.806 | [0.898, 1.137] | 0.108 | 0.092 | 1.07 |
|  |  |  |  |  |  |  |  |
| --- | --- | --- | --- | --- | --- | --- | --- |
| Note: |  |  |  |  |  |  |  |
| MSQ values based on conditional calculations (n = 754 complete cases).  Simulation based thresholds from 200 simulated datasets. |  |  |  |  |  |  |  |

Code

```
RIgetfitPlot(simfit1, d)
```

Code

```
RIrestscore(d)
```

| Item | Observed value | Model expected value | Absolute difference | Adjusted p-value (BH) | Statistical significance level | Location | Relative location |
| --- | --- | --- | --- | --- | --- | --- | --- |
| q10 | 0.56 | 0.61 | 0.05 | 0.075 | . | 0.51 | 2.10 |
| q13 | 0.43 | 0.62 | 0.19 | 0.000 | \*\*\* | 0.65 | 2.24 |
| q14 | 0.71 | 0.64 | 0.07 | 0.005 | \*\* | -0.26 | 1.34 |
| q15 | 0.70 | 0.64 | 0.06 | 0.006 | \*\* | -0.37 | 1.22 |
| q16 | 0.74 | 0.64 | 0.10 | 0.000 | \*\*\* | -0.53 | 1.07 |

Code

```
simcor1 <- RIgetResidCor(d, iterations = 250, cpu = 8)
RIresidcorr(d, cutoff = simcor1$p99)
```

|  | q10 | q13 | q14 | q15 | q16 |
| --- | --- | --- | --- | --- | --- |
| q10 |  |  |  |  |  |
| q13 | -0.11 |  |  |  |  |
| q14 | -0.22 | -0.28 |  |  |  |
| q15 | -0.35 | -0.34 | -0.15 |  |  |
| q16 | -0.27 | -0.38 | -0.09 | 0.1 |  |
|  |  |  |  |  |  |
| --- | --- | --- | --- | --- | --- |
| Note: |  |  |  |  |  |
| Relative cut-off value is -0.078, which is 0.131 above the average correlation (-0.209).  Correlations above the cut-off are highlighted in red text. |  |  |  |  |  |

Code

```
# using partial gamma LD from library(iarm)
RIpartgamLD(d)
```

| Item 1 | Item 2 | Partial gamma | SE | Lower CI | Upper CI | Adjusted p-value (BH) |
| --- | --- | --- | --- | --- | --- | --- |
| q16 | q15 | 0.461 | 0.063 | 0.338 | 0.583 | 0 |
| q15 | q16 | 0.412 | 0.063 | 0.288 | 0.535 | 0 |

Code

```
item_obsexp(PCM(d))
```

```
Score group 1: 
    mean obs mean exp std.res sig
q10  0.353    0.316    1.403     
q13  0.311    0.214    4.009  ++ 
q14  0.704    0.718   -0.435     
q15  0.789    0.830   -1.202     
q16  0.886    0.961   -2.241  -  

Score group 2: 
    mean obs mean exp std.res sig
q10  1.247    1.293   -0.951     
q13  1.055    1.171   -2.400  -  
q14  1.983    1.965    0.366     
q15  2.189    2.138    1.048     
q16  2.230    2.137    1.975
```

Item q13 is still underfit.

Residual correlations remain between q15 and q16.

We proceed by removing item q13 as well.

### 3.5 Fear without q11, q13, q17 och q18

Code

```
d$q13 <- NULL
```

- Conditional item fit
- Item-restscore
- Residual correlations
- Local dependency
- Score groups obs-exp

Code

```
simfit1 <- RIgetfit(d, iterations = 200, cpu = 8) 

RIitemfit(d, simfit1)
```

| Item | InfitMSQ | Infit thresholds | OutfitMSQ | Outfit thresholds | Infit diff | Outfit diff | Relative location |
| --- | --- | --- | --- | --- | --- | --- | --- |
| q10 | 1.372 | [0.844, 1.12] | 1.585 | [0.795, 1.2] | 0.252 | 0.385 | 2.38 |
| q14 | 0.936 | [0.904, 1.109] | 0.942 | [0.903, 1.117] | no misfit | no misfit | 1.51 |
| q15 | 0.899 | [0.878, 1.188] | 0.888 | [0.879, 1.164] | no misfit | no misfit | 1.36 |
| q16 | 0.799 | [0.891, 1.107] | 0.81 | [0.88, 1.093] | 0.092 | 0.07 | 1.20 |
|  |  |  |  |  |  |  |  |
| --- | --- | --- | --- | --- | --- | --- | --- |
| Note: |  |  |  |  |  |  |  |
| MSQ values based on conditional calculations (n = 754 complete cases).  Simulation based thresholds from 200 simulated datasets. |  |  |  |  |  |  |  |

Code

```
RIgetfitPlot(simfit1, d)
```

Code

```
RIrestscore(d)
```

| Item | Observed value | Model expected value | Absolute difference | Adjusted p-value (BH) | Statistical significance level | Location | Relative location |
| --- | --- | --- | --- | --- | --- | --- | --- |
| q10 | 0.54 | 0.67 | 0.13 | 0.000 | \*\*\* | 0.77 | 2.38 |
| q14 | 0.72 | 0.68 | 0.04 | 0.184 |  | -0.10 | 1.51 |
| q15 | 0.73 | 0.69 | 0.04 | 0.087 | . | -0.25 | 1.36 |
| q16 | 0.77 | 0.69 | 0.08 | 0.000 | \*\*\* | -0.42 | 1.20 |

Code

```
simcor1 <- RIgetResidCor(d, iterations = 250, cpu = 8)
RIresidcorr(d, cutoff = simcor1$p99)
```

|  | q10 | q14 | q15 | q16 |
| --- | --- | --- | --- | --- |
| q10 |  |  |  |  |
| q14 | -0.28 |  |  |  |
| q15 | -0.43 | -0.3 |  |  |
| q16 | -0.37 | -0.26 | -0.05 |  |
|  |  |  |  |  |
| --- | --- | --- | --- | --- |
| Note: |  |  |  |  |
| Relative cut-off value is -0.162, which is 0.12 above the average correlation (-0.282).  Correlations above the cut-off are highlighted in red text. |  |  |  |  |

Code

```
# using partial gamma LD from library(iarm)
RIpartgamLD(d)
```

| Item 1 | Item 2 | Partial gamma | SE | Lower CI | Upper CI | Adjusted p-value (BH) |
| --- | --- | --- | --- | --- | --- | --- |
| q16 | q15 | 0.4 | 0.070 | 0.263 | 0.537 | 0 |
| q15 | q16 | 0.321 | 0.069 | 0.185 | 0.457 | 0 |

Code

```
item_obsexp(PCM(d))
```

```
Score group 1: 
    mean obs mean exp std.res sig
q10  0.342    0.248    3.739  ++ 
q14  0.625    0.633   -0.222     
q15  0.726    0.750   -0.714     
q16  0.831    0.894   -1.933     

Score group 2: 
    mean obs mean exp std.res sig
q10  1.175    1.264   -2.204  -  
q14  1.945    1.938    0.169     
q15  2.126    2.104    0.536     
q16  2.169    2.109    1.493
```

Now, item q10 shows problem with being underfit.

We remove item q10 and proceed.

### 3.6 Fear without q10, q11, q13, q17 och q18

Code

```
d$q10 <- NULL
```

- Conditional item fit
- Item-restscore
- Residual correlations
- Local dependency
- Score groups obs-exp

Code

```
simfit1 <- RIgetfit(d, iterations = 200, cpu = 8) 

RIitemfit(d, simfit1)
```

| Item | InfitMSQ | Infit thresholds | OutfitMSQ | Outfit thresholds | Infit diff | Outfit diff | Relative location |
| --- | --- | --- | --- | --- | --- | --- | --- |
| q14 | 1.158 | [0.914, 1.098] | 1.143 | [0.918, 1.098] | 0.06 | 0.045 | 1.80 |
| q15 | 0.964 | [0.909, 1.102] | 0.947 | [0.924, 1.095] | no misfit | no misfit | 1.60 |
| q16 | 0.885 | [0.904, 1.101] | 0.899 | [0.899, 1.093] | 0.019 | 0 | 1.41 |
|  |  |  |  |  |  |  |  |
| --- | --- | --- | --- | --- | --- | --- | --- |
| Note: |  |  |  |  |  |  |  |
| MSQ values based on conditional calculations (n = 754 complete cases).  Simulation based thresholds from 200 simulated datasets. |  |  |  |  |  |  |  |

Code

```
RIgetfitPlot(simfit1, d)
```

Code

```
RIrestscore(d)
```

| Item | Observed value | Model expected value | Absolute difference | Adjusted p-value (BH) | Statistical significance level | Location | Relative location |
| --- | --- | --- | --- | --- | --- | --- | --- |
| q14 | 0.72 | 0.74 | 0.02 | 0.298 |  | 0.20 | 1.80 |
| q15 | 0.77 | 0.75 | 0.02 | 0.298 |  | -0.01 | 1.60 |
| q16 | 0.79 | 0.75 | 0.04 | 0.090 | . | -0.19 | 1.41 |

Code

```
simcor1 <- RIgetResidCor(d, iterations = 250, cpu = 8)
RIresidcorr(d, cutoff = simcor1$p99)
```

|  | q14 | q15 | q16 |
| --- | --- | --- | --- |
| q14 |  |  |  |
| q15 | -0.53 |  |  |
| q16 | -0.45 | -0.29 |  |
|  |  |  |  |
| --- | --- | --- | --- |
| Note: |  |  |  |
| Relative cut-off value is -0.327, which is 0.093 above the average correlation (-0.419).  Correlations above the cut-off are highlighted in red text. |  |  |  |

Code

```
# using partial gamma LD from library(iarm)
RIpartgamLD(d)
```

| Item 1 | Item 2 | Partial gamma | SE | Lower CI | Upper CI | Adjusted p-value (BH) |
| --- | --- | --- | --- | --- | --- | --- |
| q16 | q15 | 0.275 | 0.086 | 0.106 | 0.444 | 0.008 |
| q15 | q16 | 0.254 | 0.083 | 0.092 | 0.416 | 0.013 |

Code

```
item_obsexp(PCM(d))
```

```
Score group 1: 
    mean obs mean exp std.res sig
q14  0.586    0.550    1.161     
q15  0.663    0.685   -0.719     
q16  0.835    0.845   -0.319     

Score group 2: 
    mean obs mean exp std.res sig
q14  1.871    1.898   -0.789     
q15  2.071    2.052    0.557     
q16  2.074    2.067    0.226
```

This leaves us with only three items

Now there are minor problems with dimensionality. Item q14 being slightly underfit and item q16 being slightly overfit.

The issue of residual correlations between items q15 and q16 remain. It is no longer possible to remove items and retain a stable scale so we cannot adress this issue further.

Let’s review the item thresholds.

#### 3.6.1 Response categories

Code

```
mirt(d, model=1, itemtype='Rasch', verbose = FALSE) %>% 
  plot(type="trace", as.table = TRUE, 
       theta_lim = c(-6,6))
```

Code

```
RIitemHierarchy(d)
```

All three items have nice category thresholds.

Let’s inspect the test information of this adapted version of the scale.

### 3.7 Test Information (Reliability)

Code

```
RItif(d, samplePSI = T)
```

The scale shows the highest amount of information at around 2 of the latent scale, but test information is not above 3.33 at any part of the scale, thus suggesting low reliability.

We are left with a Fear scale that consists of three items that only show minor problems in terms of item misfit and has nice ordering of item thresholds but there are residual correlations left between items q15 and q16.

Previous literature has suggested dividing the fear scale into fear (items q10, q13, q14, q15 and q16) and reassurance seeking (items q11, q17 and q18). We may also test and see if these versions show better reliability.

## Reuse

CC BY 4.0

##### Source Code

```
---
title: "CAQ psychometric analysis, fear subscale"
subtitle: "Using Rasch Measurement Theory"
title-block-banner: "#009ca6"
title-block-banner-color: "#FFFFFF"
author: 
  name: Magnus Johansson; Philip Leissner
  affiliation: RISE Research Institutes of Sweden; Department of women's and children's health, Uppsala University
  affiliation-url: https://www.ri.se/en/kbm; https://www.uu.se/en/department/womens-and-childrens-health
  orcid: 0000-0003-1669-592X; 0000-0003-0787-9102
date-format: iso
always_allow_html: true
format: 
  html:
    toc: true
    toc-depth: 3
    toc-title: "Table of contents"
    embed-resources: true
    standalone: true
    page-layout: full
    mainfont: 'Lato'
    monofont: 'Roboto Mono'
    code-overflow: wrap
    code-fold: true
    code-tools: true
    code-link: true
    number-sections: true
    fig-dpi: 96
    layout-align: left
    linestretch: 1.6
    theme:
      - materia
      - custom.scss
    css: styles.css
    license: CC BY
  pdf:
    papersize: a4
    documentclass: report 
execute:
  echo: true
  warning: false
  message: false
  cache: true
editor_options: 
  markdown: 
    wrap: 72
  chunk_output_type: console
---

```{r}
#| label: setup

# one package below requires that you use devtools to install them manually:
# first install devtools by
# install.packages('devtools')

library(easyRasch) # devtools::install_github("pgmj/easyRasch")
library(grateful)
library(ggrepel)
library(car)
library(kableExtra)
library(readxl)
library(tidyverse)
library(eRm)
library(iarm)
library(mirt)
library(psych)
library(psychotree)
library(matrixStats)
library(reshape)
library(knitr)
library(patchwork)
library(formattable) 
library(glue)
library(readxl) # for reading excel files

### optional libraries
#library(TAM)
#library(skimr)
#library(janitor)

### some commands exist in multiple packages, here we define preferred ones that are frequently used
select <- dplyr::select
count <- dplyr::count
recode <- car::recode
rename <- dplyr::rename
```


```{r}
### import data - this is just sample code, the files do not exist
df <- read_excel("data/CAQ_Rasch.xlsx") # replace with your datafile as needed

#library(haven) # for SPSS and other formats
#library(labelled) # for getting labels and metadata from SPSS files

### Load item information
# make sure that variable names in df match with itemlabels$itemnr
iteminfo <- read_excel("data/iteminfo.xlsx")

itemlabels <- iteminfo[,1:2]

### Make a backup of the dataframe, in case you need to revert changes at some point
d <- df
```

```{r}
##### Optionally: filter participants based on missing data

##### Before filtering out participants, you should check the missing data structure using RImissing() and RImissingP()

RImissing(d)
```

RImissingP() needs to be addressed, so we'll do a manual check:

```{r}
d[,6:23] %>% 
  mutate(missing = rowSums(is.na(.))) %>% 
  count(missing)
```

We lose 50 respondents if we remove everyone with a missing value on any item. 756 is a good sample size, so we'll go with that.

```{r}
d <- na.omit(d)
```

2 who were missing on some demographic variable were also removed.

```{r}
#---- Create DIF variables----
  
# DIF variables into vectors, recoded as factors since DIF functions need this
# these could also be stored in its own dataframe (not a tibble) instead of as vectors

d_dif <- d %>% 
  mutate(sex = factor(SEX),
         age = AGE_R,
         rel_status = factor(RELSTAT_bin),
         born_swe = factor(BORNSWE),
         edu = factor(EDUCATION)) %>% 
  select(sex,age,rel_status,born_swe,edu)

# remove DIF variables from item data
d <- d %>% 
  select(starts_with("Item")) %>% 
  set_names(itemlabels$itemnr)


### label sex variable as factor
# dif.sex <- factor(dif.sex,
#                       levels = c(1,2,3),
#                       labels = c("Female", "Male", "Other/missing response"))

# optionally, load RISE ggplot theme and color palettes and set the theme as default.
# just comment out the row below if you desire different theming
source("RISE_theme.R")
```

## All items in the analysis
```{r}
RIlistitems(d)
```

Response distribution for all items are summarized below.

```{r}
#| tbl-cap: "Total number of responses for all items"
RIallresp(d)
```

## Descriptives - item level

```{r}
#| column: margin
RIlistItemsMargin(d, fontsize = 12)
```

::: panel-tabset
### Tile plot
```{r}
RItileplot(d)
```
### Stacked bars
```{r}
RIbarstack(d) + scale_fill_viridis_d(labels = c("Always","Often","Sometimes","Rarely","Never"), direction = -1)
```
### Barplots
```{r}
#| layout-ncol: 2
RIbarplot(d)
```
:::

Very few individuals endorse the highest categories of items q3, q4, q10, and q13. Over half of the participants endorse the lowest category for items q3, q4, and q13.

## Fear subscale

```{r}
d_all <- d

items_f <- iteminfo %>% 
  filter(factor == "fear") %>% 
  pull(itemnr)

d <- d_all %>% 
  select(all_of(items_f))
  
```

### Sumscore
```{r}
d_sum <- d %>%
  mutate(total = rowSums(select(., starts_with("q")), na.rm = TRUE))
d_sum <- d_sum["total"]
```

```{r}
total_freq <- as.data.frame(table(d_sum$total))
colnames(total_freq) <- c("Total", "N")
total_freq$Percent <- round(100 * total_freq$N / sum(total_freq$N), 1)
```

```{r}
RIallresp(d_sum)
```

### Rasch analysis

The eRm package, which uses Conditional Maximum Likelihood (CML)
estimation, will be used primarily. For this analysis, the Partial
Credit Model will be used.

```{r}
#| column: margin
#| echo: false
RIlistItemsMargin(d, fontsize = 13)
```

::: panel-tabset
#### Conditional item fit
```{r}
#RIitemfit(d, cutoff = "Smith98")

simfit1 <- RIgetfit(d, iterations = 200, cpu = 8) 

RIitemfit(d, simfit1)
RIgetfitPlot(simfit1, d)
```
#### Item-restscore
```{r}
RIrestscore(d)
```
#### Conditional LRT
```{r}
clr_tests(d, model = "PCM")

RIbootLRT(d, iterations = 1000, samplesize = 400, cpu = 8)
```
#### Local dependency
```{r}
# using partial gamma LD from library(iarm)
RIpartgamLD(d)
```

#### Residual correlations
```{r}
simcor1 <- RIgetResidCor(d, iterations = 250, cpu = 8)
RIresidcorr(d, cutoff = simcor1$p99)
```
#### 1st contrast loadings
```{r}
RIloadLoc(d)
```
#### Response categories
```{r}
mirt(d, model=1, itemtype='Rasch', verbose = FALSE) %>% 
  plot(type="trace", as.table = TRUE, 
       theta_lim = c(-6,6))
# for fewer items or a more magnified figure, use:
#RIitemCats(d)
```
#### Targeting
```{r}
#| fig-height: 5
# increase fig-height above as needed, if you have many items
RItargeting(d)
```
#### Item hierarchy
```{r}
#| fig-height: 5
RIitemHierarchy(d)
```
#### Score groups LR-test
```{r}
iarm::score_groups(as.data.frame(d)) %>% 
  as.data.frame(nm = "score_group") %>% 
  dplyr::count(score_group)

dif_plots <- d %>% 
  add_column(dif = iarm::score_groups(.)) %>% 
  split(.$dif) %>% # split the data using the DIF variable
  map(~ RItileplot(.x %>% dplyr::select(!dif)) + labs(title = .x$dif))
dif_plots[[1]] + dif_plots[[2]]

clr_tests(d, model = "PCM")
```
#### Score groups obs-exp
```{r}
item_obsexp(PCM(d))
```
#### Rasch-tree DIF immigration status
```{r}
RIdifTable(d, d_dif$born_swe)
```
#### Rasch-tree DIF sex
```{r}
RIdifTable(d, d_dif$sex)
```
#### Rasch-tree DIF rel
```{r}
RIdifTable(d, d_dif$rel_status)
```
#### Rasch-tree DIF age
```{r}
RIdifTable(d, d_dif$age)
```
#### Rasch-tree DIF rel*age
```{r}
RIdifTable2(d, d_dif$rel_status, d_dif$sex)
```
#### Person fit
```{r}
RIpfit(d)
```
#### Floor and ceiling effects
```{r}
RItif(d, samplePSI = T, cutoff = 1)
```
:::

There are quite a few issues with item fit here. Particularly items q11, q13, and q18, which are underfit, while items q14 and q16 appear to be heavily overfit.

There are residual correlations between items q10 and q13, q14 and q15, q14 and q15, q15 and q16, and q17 and q18.On the 1st contrast loadings items can bee seen to cluster into distinct groups.

All items except q15 and q16 have disordered response thresholds, with item q11 being the biggest problem.

Items q18 shows significant DIF for men that are in a relationship compared to men who are not in a relationship. Interesting finding that may be related to sex and gender based relationship tendencies.

The problems with item misfit and residual correlations indicate that the Fear subscale is not unidimensional. Several previous studies have divided this scale into two (Fear and Reassurance Seeking). The reassurance seeking scale traditionally consists of item q11, q17 and q18. 

In a previous study, using exploratory factor analysis, items q10 and q13  were removed due to cross-loading. We may need to remove many items or split the scale, but as a starting point, we begin with items q11 and q18, as they have the most severe dimensionality issues.

### Fear without q11 och q18

```{r}
d$q11 <- NULL
d$q18 <- NULL
```

```{r}
#| column: margin
#| echo: false
RIlistItemsMargin(d, fontsize = 13)
```

::: panel-tabset
#### Conditional item fit
```{r}
simfit1 <- RIgetfit(d, iterations = 200, cpu = 8) 

RIitemfit(d, simfit1)
RIgetfitPlot(simfit1, d)
```
#### Item-restscore
```{r}
RIrestscore(d)
```
#### Residual correlations
```{r}
simcor1 <- RIgetResidCor(d, iterations = 250, cpu = 8)
RIresidcorr(d, cutoff = simcor1$p99)
```
#### Local dependency
```{r}
# using partial gamma LD from library(iarm)
RIpartgamLD(d)
```
#### 1st contrast loadings
```{r}
RIloadLoc(d)
```
#### Score groups obs-exp
```{r}
item_obsexp(PCM(d))
```
#### Rasch-tree DIF relationship and sex
```{r}
RIdifTable2(d, d_dif$rel_status, d_dif$sex)
```
:::

Item q13 is still problematic in terms of dimensionality, but now is also item q17 underfit.

We’ll proceed by removing item q17 as it shows the most problems.

### Fear without q11, q17 och q18

```{r}
d$q17 <- NULL
```

::: panel-tabset
#### Conditional item fit
```{r}
simfit1 <- RIgetfit(d, iterations = 200, cpu = 8) 

RIitemfit(d, simfit1)
RIgetfitPlot(simfit1, d)
```
#### Item-restscore
```{r}
RIrestscore(d)
```
#### Residual correlations
```{r}
simcor1 <- RIgetResidCor(d, iterations = 250, cpu = 8)
RIresidcorr(d, cutoff = simcor1$p99)
```
#### Local dependency
```{r}
# using partial gamma LD from library(iarm)
RIpartgamLD(d)
```
#### Score groups obs-exp
```{r}
item_obsexp(PCM(d))
```
:::

Item q13 is still underfit. 

Residual correlations remain between q15 and q16.

We proceed by removing item q13 as well.


### Fear without q11, q13, q17 och q18

```{r}
d$q13 <- NULL
```

::: panel-tabset
#### Conditional item fit
```{r}
simfit1 <- RIgetfit(d, iterations = 200, cpu = 8) 

RIitemfit(d, simfit1)
RIgetfitPlot(simfit1, d)
```
#### Item-restscore
```{r}
RIrestscore(d)
```
#### Residual correlations
```{r}
simcor1 <- RIgetResidCor(d, iterations = 250, cpu = 8)
RIresidcorr(d, cutoff = simcor1$p99)
```
#### Local dependency
```{r}
# using partial gamma LD from library(iarm)
RIpartgamLD(d)
```
#### Score groups obs-exp
```{r}
item_obsexp(PCM(d))
```
:::

Now, item q10 shows problem with being underfit. 

We remove item q10 and proceed.


### Fear without q10, q11, q13, q17 och q18

```{r}
d$q10 <- NULL
```

::: panel-tabset
#### Conditional item fit
```{r}
simfit1 <- RIgetfit(d, iterations = 200, cpu = 8) 

RIitemfit(d, simfit1)
RIgetfitPlot(simfit1, d)
```
#### Item-restscore
```{r}
RIrestscore(d)
```
#### Residual correlations
```{r}
simcor1 <- RIgetResidCor(d, iterations = 250, cpu = 8)
RIresidcorr(d, cutoff = simcor1$p99)
```
#### Local dependency
```{r}
# using partial gamma LD from library(iarm)
RIpartgamLD(d)
```
#### Score groups obs-exp
```{r}
item_obsexp(PCM(d))
```
:::

This leaves us with only three items

Now there are minor problems with dimensionality. Item q14 being slightly underfit and item q16 being slightly overfit.

The issue of residual correlations between items q15 and q16 remain. It is no longer possible to remove items and retain a stable scale so we cannot adress this issue further.

Let's review the item thresholds.

#### Response categories
```{r}
mirt(d, model=1, itemtype='Rasch', verbose = FALSE) %>% 
  plot(type="trace", as.table = TRUE, 
       theta_lim = c(-6,6))

RIitemHierarchy(d)
```

All three items have nice category thresholds.

Let's inspect the test information of this adapted version of the scale.

### Test Information (Reliability)
```{r}
RItif(d, samplePSI = T)
```

The scale shows the highest amount of information at around 2 of the latent scale, but test information is not above 3.33 at any part of the scale, thus suggesting low reliability.

We are left with a Fear scale that consists of three items that only show minor problems in terms of item misfit and has nice ordering of item thresholds but there are residual correlations left between items q15 and q16.

Previous literature has suggested dividing the fear scale into fear (items q10, q13, q14, q15 and q16) and reassurance seeking (items q11, q17 and q18). We may also test and see if these versions show better reliability.
```
