# Supplementary material for: Psychometric properties of the Swedish cardiac anxiety questionnaire: a Rasch analysis
Source: Sci Rep. 2025 Nov 24;15:41834. doi: 10.1038/s41598-025-28073-8 (PMC12647126; doi:10.1038/s41598-025-28073-8)
Supplement: Supplementary file 1 — Supplementary Material 1 [file 41598_2025_28073_MOESM1_ESM.zip › Supplementary/analysis_rs.html]

CAQ psychometric analysis, reassurance seeking subscale


# CAQ psychometric analysis, reassurance seeking subscale

 Code

- Show All Code
- Hide All Code
- ---
- View Source

Using Rasch Measurement Theory

Author

Affiliation

Magnus Johansson; Philip Leissner

RISE Research Institutes of Sweden; Department of women’s and children’s health, Uppsala University

Published

2025-08-28

## Table of contents

- 1 All items in the analysis
- 2 Descriptives - item level
- 3 Reassurance seeking subscale
  - 3.1 Sumscore
  - 3.2 Rasch analysis 1
  - 3.3 Test Information (Reliability)

## Other Formats

- PDF

Code

```
# one package below requires that you use devtools to install them manually:
# first install devtools by
# install.packages('devtools')

library(easyRasch) # devtools::install_github("pgmj/easyRasch")
library(grateful)
library(ggrepel)
library(car)
library(kableExtra)
library(readxl)
library(tidyverse)
library(eRm)
library(iarm)
library(mirt)
library(psych)
library(psychotree)
library(matrixStats)
library(reshape)
library(knitr)
library(patchwork)
library(formattable) 
library(glue)
library(readxl) # for reading excel files

### optional libraries
#library(TAM)
#library(skimr)
#library(janitor)

### some commands exist in multiple packages, here we define preferred ones that are frequently used
select <- dplyr::select
count <- dplyr::count
recode <- car::recode
rename <- dplyr::rename
```

Code

```
### import data - this is just sample code, the files do not exist
df <- read_excel("data/CAQ_Rasch.xlsx") # replace with your datafile as needed

#library(haven) # for SPSS and other formats
#library(labelled) # for getting labels and metadata from SPSS files

### Load item information
# make sure that variable names in df match with itemlabels$itemnr
iteminfo <- read_excel("data/iteminfo.xlsx")

itemlabels <- iteminfo[,1:2]

### Make a backup of the dataframe, in case you need to revert changes at some point
d <- df
```

Code

```
##### Optionally: filter participants based on missing data

##### Before filtering out participants, you should check the missing data structure using RImissing() and RImissingP()

RImissing(d)
```

RImissingP() needs to be addressed, so we’ll do a manual check:

Code

```
d[,6:23] %>% 
  mutate(missing = rowSums(is.na(.))) %>% 
  count(missing)
```

```
# A tibble: 10 × 2
   missing     n
     <dbl> <int>
 1       0   756
 2       1    28
 3       2     8
 4       3     1
 5       4     3
 6       5     2
 7       9     1
 8      10     1
 9      16     1
10      18     5
```

We lose 50 respondents if we remove everyone with a missing value on any item. 756 is a good sample size, so we’ll go with that.

Code

```
d <- na.omit(d)
```

2 who were missing on some demographic variable were also removed.

Code

```
#---- Create DIF variables----
  
# DIF variables into vectors, recoded as factors since DIF functions need this
# these could also be stored in its own dataframe (not a tibble) instead of as vectors

d_dif <- d %>% 
  mutate(sex = factor(SEX),
         age = AGE_R,
         rel_status = factor(RELSTAT_bin),
         born_swe = factor(BORNSWE),
         edu = factor(EDUCATION)) %>% 
  select(sex,age,rel_status,born_swe,edu)

# remove DIF variables from item data
d <- d %>% 
  select(starts_with("Item")) %>% 
  set_names(itemlabels$itemnr)


### label gender variable as factor
# dif.gender <- factor(dif.gender,
#                       levels = c(1,2,3),
#                       labels = c("Female", "Male", "Other/missing response"))

# optionally, load RISE ggplot theme and color palettes and set the theme as default.
# just comment out the row below if you desire different theming
source("RISE_theme.R")
```

## 1 All items in the analysis

Code

```
RIlistitems(d)
```

| itemnr | item |
| --- | --- |
| q1 | I pay attention to my heart beat |
| q2 | I avoid physical exertion |
| q3 | My racing heart wakes me up at night |
| q4 | Chest pain/discomfort wakes me up at night |
| q5 | I take it easy as much as possible |
| q6 | I check my pulse |
| q7 | I avoid exercise or other physical work |
| q8 | I can feel my heart in my chest |
| q9 | I avoid activities that make my heart beat faster |
| q10 | If tests come out normal, I still worry about my heart |
| q11 | I feel safe being around a hospital, physician, or other medical facility |
| q12 | I avoid activities that make me sweat |
| q13 | I worry that doctors do not believe my chest pain/discomfort is real |
| q14 | When I have chest discomfort or I feel my heart is beating fast I worry that I may have a heart attack |
| q15 | When I have chest discomfort or I feel my heart is beating fast I have difficulty concentrating on anything else |
| q16 | When I have chest discomfort or I feel my heart is beating fast I get frightened |
| q17 | When I have chest discomfort or I feel my heart is beating fast I like to be checked out by a doctor |
| q18 | When I have chest discomfort or I feel my heart is beating fast I tell my family or friends |

Response distribution for all items are summarized below.

Code

```
RIallresp(d)
```

| Response category | Number of responses | Percent |
| --- | --- | --- |
| 0 | 5359 | 39.5 |
| 1 | 3580 | 26.4 |
| 2 | 2767 | 20.4 |
| 3 | 1244 | 9.2 |
| 4 | 622 | 4.6 |

## 2 Descriptives - item level

Code

```
RIlistItemsMargin(d, fontsize = 12)
```

| itemnr | item |
| --- | --- |
| q1 | I pay attention to my heart beat |
| q2 | I avoid physical exertion |
| q3 | My racing heart wakes me up at night |
| q4 | Chest pain/discomfort wakes me up at night |
| q5 | I take it easy as much as possible |
| q6 | I check my pulse |
| q7 | I avoid exercise or other physical work |
| q8 | I can feel my heart in my chest |
| q9 | I avoid activities that make my heart beat faster |
| q10 | If tests come out normal, I still worry about my heart |
| q11 | I feel safe being around a hospital, physician, or other medical facility |
| q12 | I avoid activities that make me sweat |
| q13 | I worry that doctors do not believe my chest pain/discomfort is real |
| q14 | When I have chest discomfort or I feel my heart is beating fast I worry that I may have a heart attack |
| q15 | When I have chest discomfort or I feel my heart is beating fast I have difficulty concentrating on anything else |
| q16 | When I have chest discomfort or I feel my heart is beating fast I get frightened |
| q17 | When I have chest discomfort or I feel my heart is beating fast I like to be checked out by a doctor |
| q18 | When I have chest discomfort or I feel my heart is beating fast I tell my family or friends |

- Tile plot
- Stacked bars
- Barplots

Code

```
RItileplot(d)
```

Code

```
RIbarstack(d)
```

Code

```
RIbarplot(d)
```

## 3 Reassurance seeking subscale

Code

```
d_all <- d

items_rs <- iteminfo %>% 
  filter(rseeking == "rseeking") %>% 
  pull(itemnr)

d <- d_all %>% 
  select(all_of(items_rs))
```

### 3.1 Sumscore

Code

```
d_sum <- d %>%
  mutate(total = rowSums(select(., starts_with("q")), na.rm = TRUE))
d_sum <- d_sum["total"]
```

Code

```
total_freq <- as.data.frame(table(d_sum$total))
colnames(total_freq) <- c("Total", "N")
total_freq$Percent <- round(100 * total_freq$N / sum(total_freq$N), 1)
```

Code

```
RIallresp(d_sum)
```

| Response category | Number of responses | Percent |
| --- | --- | --- |
| 0 | 57 | 7.6 |
| 1 | 50 | 6.6 |
| 2 | 63 | 8.4 |
| 3 | 81 | 10.7 |
| 4 | 102 | 13.5 |
| 5 | 68 | 9.0 |
| 6 | 89 | 11.8 |
| 7 | 59 | 7.8 |
| 8 | 70 | 9.3 |
| 9 | 37 | 4.9 |
| 10 | 29 | 3.8 |
| 11 | 18 | 2.4 |
| 12 | 31 | 4.1 |

### 3.2 Rasch analysis 1

The eRm package, which uses Conditional Maximum Likelihood (CML) estimation, will be used primarily. For this analysis, the Partial Credit Model will be used.

| itemnr | item |
| --- | --- |
| q11 | I feel safe being around a hospital, physician, or other medical facility |
| q17 | When I have chest discomfort or I feel my heart is beating fast I like to be checked out by a doctor |
| q18 | When I have chest discomfort or I feel my heart is beating fast I tell my family or friends |

- Conditional item fit
- Item-restscore
- Conditional LRT
- Local dependency
- Residual correlations
- 1st contrast loadings
- Response categories
- Targeting
- Item hierarchy
- Score groups LR-test
- Score groups obs-exp
- Rasch-tree DIF immigration status
- Rasch-tree DIF sex
- Rasch-tree DIF age
- Rasch-tree DIF relationship and sex
- Person fit
- Floor and ceiling effects

Code

```
#RIitemfit(d, cutoff = "Smith98")

simfit1 <- RIgetfit(d, iterations = 200, cpu = 8) 

RIitemfit(d, simfit1)
```

| Item | InfitMSQ | Infit thresholds | OutfitMSQ | Outfit thresholds | Infit diff | Outfit diff | Relative location |
| --- | --- | --- | --- | --- | --- | --- | --- |
| q11 | 1.247 | [0.898, 1.115] | 1.281 | [0.871, 1.117] | 0.132 | 0.164 | 0.29 |
| q17 | 0.765 | [0.905, 1.136] | 0.783 | [0.916, 1.143] | 0.14 | 0.133 | 0.35 |
| q18 | 1.054 | [0.895, 1.091] | 1.043 | [0.9, 1.072] | no misfit | no misfit | 0.07 |
|  |  |  |  |  |  |  |  |
| --- | --- | --- | --- | --- | --- | --- | --- |
| Note: |  |  |  |  |  |  |  |
| MSQ values based on conditional calculations (n = 754 complete cases).  Simulation based thresholds from 200 simulated datasets. |  |  |  |  |  |  |  |

Code

```
RIgetfitPlot(simfit1, d)
```

Code

```
RIrestscore(d)
```

| Item | Observed value | Model expected value | Absolute difference | Adjusted p-value (BH) | Statistical significance level | Location | Relative location |
| --- | --- | --- | --- | --- | --- | --- | --- |
| q11 | 0.38 | 0.46 | 0.08 | 0.015 | \* | 0.06 | 0.29 |
| q17 | 0.60 | 0.46 | 0.14 | 0.000 | \*\*\* | 0.12 | 0.35 |
| q18 | 0.44 | 0.46 | 0.02 | 0.529 |  | -0.17 | 0.07 |

Code

```
clr_tests(d, model = "PCM")
```

```
Conditional Likelihood Ratio Tests:
```

```
        clr  df pvalue sig 
overall 43.1 11 1e-05   ***
```

Code

```
RIbootLRT(d, iterations = 1000, samplesize = 400, cpu = 8)
```

| Result | n | Percent |
| --- | --- | --- |
| Not statistically significant | 57 | 5.7 |
| Statistically significant | 943 | 94.3 |

Code

```
# using partial gamma LD from library(iarm)
RIpartgamLD(d)
```

| Item 1 | Item 2 | Partial gamma | SE | Lower CI | Upper CI | Adjusted p-value (BH) |
| --- | --- | --- | --- | --- | --- | --- |
| q17 | q18 | 0.44 | 0.048 | 0.346 | 0.535 | 0.000 |
| q17 | q11 | 0.288 | 0.055 | 0.180 | 0.397 | 0.000 |
| q18 | q17 | 0.195 | 0.054 | 0.089 | 0.302 | 0.002 |

Code

```
simcor1 <- RIgetResidCor(d, iterations = 250, cpu = 8)
RIresidcorr(d, cutoff = simcor1$p99)
```

|  | q11 | q17 | q18 |
| --- | --- | --- | --- |
| q11 |  |  |  |
| q17 | -0.35 |  |  |
| q18 | -0.6 | -0.08 |  |
|  |  |  |  |
| --- | --- | --- | --- |
| Note: |  |  |  |
| Relative cut-off value is -0.21, which is 0.132 above the average correlation (-0.341).  Correlations above the cut-off are highlighted in red text. |  |  |  |

Code

```
RIloadLoc(d)
```

Code

```
mirt(d, model=1, itemtype='Rasch', verbose = FALSE) %>% 
  plot(type="trace", as.table = TRUE, 
       theta_lim = c(-6,6))
```

Code

```
# for fewer items or a more magnified figure, use:
#RIitemCats(d)
```

Code

```
# increase fig-height above as needed, if you have many items
RItargeting(d)
```

Code

```
RIitemHierarchy(d)
```

Code

```
iarm::score_groups(as.data.frame(d)) %>% 
  as.data.frame(nm = "score_group") %>% 
  dplyr::count(score_group)
```

```
  score_group   n
1           1 421
2           2 333
```

Code

```
dif_plots <- d %>% 
  add_column(dif = iarm::score_groups(.)) %>% 
  split(.$dif) %>% # split the data using the DIF variable
  map(~ RItileplot(.x %>% dplyr::select(!dif)) + labs(title = .x$dif))
dif_plots[[1]] + dif_plots[[2]]
```

Code

```
clr_tests(d, model = "PCM")
```

```
Conditional Likelihood Ratio Tests:
```

```
        clr  df pvalue sig 
overall 43.1 11 1e-05   ***
```

Code

```
item_obsexp(PCM(d))
```

```
Score group 1: 
    mean obs mean exp std.res sig
q11  0.937    0.850    1.982     
q17  0.909    0.997   -2.262  -  
q18  1.360    1.328    0.736     

Score group 2: 
    mean obs mean exp std.res sig
q11  2.659    2.764   -1.773     
q17  2.394    2.250    2.549  +  
q18  2.656    2.694   -0.694
```

Code

```
RIdifTable(d, d_dif$born_swe)
```

| Item | 2 | 3 | Mean location | StDev | MaxDiff |
| --- | --- | --- | --- | --- | --- |
| q11 | -0.165 | 0.069 | -0.048 | 0.166 | 0.235 |
| q17 | 0.146 | 0.122 | 0.134 | 0.018 | 0.025 |
| q18 | 0.019 | -0.191 | -0.086 | 0.148 | 0.210 |

Code

```
RIdifTable(d, d_dif$sex)
```

```
[1] "No statistically significant DIF found."
```

Code

```
RIdifTable(d, d_dif$age)
```

```
[1] "No statistically significant DIF found."
```

Code

```
RIdifTable2(d, d_dif$rel_status, d_dif$sex)
```

| Item | 2 | 3 | Mean location | StDev | MaxDiff |
| --- | --- | --- | --- | --- | --- |
| q11 | 0.110 | -0.182 | -0.036 | 0.206 | 0.292 |
| q17 | 0.096 | 0.216 | 0.156 | 0.085 | 0.120 |
| q18 | -0.205 | -0.034 | -0.120 | 0.122 | 0.172 |

Code

```
RIpfit(d)
```

Code

```
RItif(d, samplePSI = T, cutoff = 1)
```

Item q11 does not quite fit in this scale as it is underfit. This could be expected as the item is not very specific to anxiety. Item q17 seem to be overfit and q17 and q18 have residual correlations. Not very strange given the semantic similarity between the two.

6.5% have person locations above the highest item threshold (1.13), and 14.2% are below the lowest item threshold (-1.33).

Unlike the analysis of the full fear scale, item q18 does not show significant DIF for sex and relationship status. No other DIF is observed.

Regarding response categories, item q11 barely has any distance between thresholds. Both items q17 and q18 has unclear ordering of the top thresholds.

Let’s begin with re-coding response categories.

Code

```
d %>% 
  mutate(q11 = car::recode(q11,"2=1;3=2;4=2"),
         q17 = car::recode(q17,"4=3"),
         q18 = car::recode(q18,"4=3; 3=2")) %>% 
  RItileplot()
```

Code

```
d %>% 
  mutate(q11 = car::recode(q11,"2=1;3=2;4=2"),
         q17 = car::recode(q17,"4=3"),
         q18 = car::recode(q18,"4=3; 3=2")) %>% 
  mirt(model=1, itemtype='Rasch', verbose = FALSE) %>% 
  plot(type="trace", as.table = TRUE, 
       theta_lim = c(-6,6))
```

Looks better

Code

```
d2 <- d %>% 
  mutate(q11 = car::recode(q11,"2=1;3=2;4=2"),
         q17 = car::recode(q17,"4=3"),
         q18 = car::recode(q18,"4=3; 3=2"))
```

Looks better

### 3.3 Test Information (Reliability)

Code

```
RItif(d2, samplePSI = T)
```

Low reliability

## Reuse

CC BY 4.0

##### Source Code

```
---
title: "CAQ psychometric analysis, reassurance seeking subscale"
subtitle: "Using Rasch Measurement Theory"
title-block-banner: "#009ca6"
title-block-banner-color: "#FFFFFF"
author: 
  name: Magnus Johansson; Philip Leissner
  affiliation: RISE Research Institutes of Sweden; Department of women's and children's health, Uppsala University
  affiliation-url: https://www.ri.se/en/kbm; https://www.uu.se/en/department/womens-and-childrens-health
  orcid: 0000-0003-1669-592X; 0000-0003-0787-9102
date: last-modified
date-format: iso
always_allow_html: true
format: 
  html:
    toc: true
    toc-depth: 3
    toc-title: "Table of contents"
    embed-resources: true
    standalone: true
    page-layout: full
    mainfont: 'Lato'
    monofont: 'Roboto Mono'
    code-overflow: wrap
    code-fold: true
    code-tools: true
    code-link: true
    number-sections: true
    fig-dpi: 96
    layout-align: left
    linestretch: 1.6
    theme:
      - materia
      - custom.scss
    css: styles.css
    license: CC BY
  pdf:
    papersize: a4
    documentclass: report 
execute:
  echo: true
  warning: false
  message: false
  cache: false
editor_options: 
  markdown: 
    wrap: 72
  chunk_output_type: console
---

```{r}
#| label: setup

# one package below requires that you use devtools to install them manually:
# first install devtools by
# install.packages('devtools')

library(easyRasch) # devtools::install_github("pgmj/easyRasch")
library(grateful)
library(ggrepel)
library(car)
library(kableExtra)
library(readxl)
library(tidyverse)
library(eRm)
library(iarm)
library(mirt)
library(psych)
library(psychotree)
library(matrixStats)
library(reshape)
library(knitr)
library(patchwork)
library(formattable) 
library(glue)
library(readxl) # for reading excel files

### optional libraries
#library(TAM)
#library(skimr)
#library(janitor)

### some commands exist in multiple packages, here we define preferred ones that are frequently used
select <- dplyr::select
count <- dplyr::count
recode <- car::recode
rename <- dplyr::rename
```


```{r}
### import data - this is just sample code, the files do not exist
df <- read_excel("data/CAQ_Rasch.xlsx") # replace with your datafile as needed

#library(haven) # for SPSS and other formats
#library(labelled) # for getting labels and metadata from SPSS files

### Load item information
# make sure that variable names in df match with itemlabels$itemnr
iteminfo <- read_excel("data/iteminfo.xlsx")

itemlabels <- iteminfo[,1:2]

### Make a backup of the dataframe, in case you need to revert changes at some point
d <- df
```

```{r}
##### Optionally: filter participants based on missing data

##### Before filtering out participants, you should check the missing data structure using RImissing() and RImissingP()

RImissing(d)
```

RImissingP() needs to be addressed, so we'll do a manual check:

```{r}
d[,6:23] %>% 
  mutate(missing = rowSums(is.na(.))) %>% 
  count(missing)
```

We lose 50 respondents if we remove everyone with a missing value on any item. 756 is a good sample size, so we’ll go with that.

```{r}
d <- na.omit(d)
```

2 who were missing on some demographic variable were also removed.

```{r}
#---- Create DIF variables----
  
# DIF variables into vectors, recoded as factors since DIF functions need this
# these could also be stored in its own dataframe (not a tibble) instead of as vectors

d_dif <- d %>% 
  mutate(sex = factor(SEX),
         age = AGE_R,
         rel_status = factor(RELSTAT_bin),
         born_swe = factor(BORNSWE),
         edu = factor(EDUCATION)) %>% 
  select(sex,age,rel_status,born_swe,edu)

# remove DIF variables from item data
d <- d %>% 
  select(starts_with("Item")) %>% 
  set_names(itemlabels$itemnr)


### label gender variable as factor
# dif.gender <- factor(dif.gender,
#                       levels = c(1,2,3),
#                       labels = c("Female", "Male", "Other/missing response"))

# optionally, load RISE ggplot theme and color palettes and set the theme as default.
# just comment out the row below if you desire different theming
source("RISE_theme.R")
```

## All items in the analysis
```{r}
RIlistitems(d)
```

Response distribution for all items are summarized below.

```{r}
#| tbl-cap: "Total number of responses for all items"
RIallresp(d)
```

## Descriptives - item level

```{r}
#| column: margin
RIlistItemsMargin(d, fontsize = 12)
```

::: panel-tabset
#### Tile plot
```{r}
RItileplot(d)
```
#### Stacked bars
```{r}
RIbarstack(d)
```
#### Barplots
```{r}
#| layout-ncol: 2
RIbarplot(d)
```
:::

## Reassurance seeking subscale

```{r}
d_all <- d

items_rs <- iteminfo %>% 
  filter(rseeking == "rseeking") %>% 
  pull(itemnr)

d <- d_all %>% 
  select(all_of(items_rs))
  
```

### Sumscore
```{r}
d_sum <- d %>%
  mutate(total = rowSums(select(., starts_with("q")), na.rm = TRUE))
d_sum <- d_sum["total"]
```

```{r}
total_freq <- as.data.frame(table(d_sum$total))
colnames(total_freq) <- c("Total", "N")
total_freq$Percent <- round(100 * total_freq$N / sum(total_freq$N), 1)
```

```{r}
RIallresp(d_sum)
```

### Rasch analysis 1

The eRm package, which uses Conditional Maximum Likelihood (CML)
estimation, will be used primarily. For this analysis, the Partial
Credit Model will be used.

```{r}
#| column: margin
#| echo: false
RIlistItemsMargin(d, fontsize = 13)
```

::: panel-tabset
#### Conditional item fit
```{r}
#RIitemfit(d, cutoff = "Smith98")

simfit1 <- RIgetfit(d, iterations = 200, cpu = 8) 

RIitemfit(d, simfit1)
RIgetfitPlot(simfit1, d)
```
#### Item-restscore
```{r}
RIrestscore(d)
```
#### Conditional LRT
```{r}
clr_tests(d, model = "PCM")

RIbootLRT(d, iterations = 1000, samplesize = 400, cpu = 8)
```
#### Local dependency
```{r}
# using partial gamma LD from library(iarm)
RIpartgamLD(d)
```

#### Residual correlations
```{r}
simcor1 <- RIgetResidCor(d, iterations = 250, cpu = 8)
RIresidcorr(d, cutoff = simcor1$p99)
```
#### 1st contrast loadings
```{r}
RIloadLoc(d)
```
#### Response categories
```{r}
mirt(d, model=1, itemtype='Rasch', verbose = FALSE) %>% 
  plot(type="trace", as.table = TRUE, 
       theta_lim = c(-6,6))
# for fewer items or a more magnified figure, use:
#RIitemCats(d)
```
#### Targeting
```{r}
#| fig-height: 5
# increase fig-height above as needed, if you have many items
RItargeting(d)
```
#### Item hierarchy
```{r}
#| fig-height: 5
RIitemHierarchy(d)
```
#### Score groups LR-test
```{r}
iarm::score_groups(as.data.frame(d)) %>% 
  as.data.frame(nm = "score_group") %>% 
  dplyr::count(score_group)

dif_plots <- d %>% 
  add_column(dif = iarm::score_groups(.)) %>% 
  split(.$dif) %>% # split the data using the DIF variable
  map(~ RItileplot(.x %>% dplyr::select(!dif)) + labs(title = .x$dif))
dif_plots[[1]] + dif_plots[[2]]

clr_tests(d, model = "PCM")
```
#### Score groups obs-exp
```{r}
item_obsexp(PCM(d))
```
#### Rasch-tree DIF immigration status
```{r}
RIdifTable(d, d_dif$born_swe)
```
#### Rasch-tree DIF sex
```{r}
RIdifTable(d, d_dif$sex)
```
#### Rasch-tree DIF age
```{r}
RIdifTable(d, d_dif$age)
```
#### Rasch-tree DIF relationship and sex
```{r}
RIdifTable2(d, d_dif$rel_status, d_dif$sex)
```
#### Person fit
```{r}
RIpfit(d)
```
#### Floor and ceiling effects
```{r}
RItif(d, samplePSI = T, cutoff = 1)
```
:::

Item q11 does not quite fit in this scale as it is underfit. This could be expected as the item is not very specific to anxiety. Item q17 seem to be overfit and q17 and q18 have residual correlations. Not very strange given the semantic similarity between the two.

6.5% have person locations above the highest item threshold (1.13), and 14.2% are below the lowest item threshold (-1.33).

Unlike the analysis of the full fear scale, item q18 does not show significant DIF for sex and relationship status. No other DIF is observed.

Regarding response categories, item q11 barely has any distance between thresholds. Both items q17 and q18 has unclear ordering of the top thresholds.

Let's begin with re-coding response categories.

```{r}
d %>% 
  mutate(q11 = car::recode(q11,"2=1;3=2;4=2"),
         q17 = car::recode(q17,"4=3"),
         q18 = car::recode(q18,"4=3; 3=2")) %>% 
  RItileplot()

d %>% 
  mutate(q11 = car::recode(q11,"2=1;3=2;4=2"),
         q17 = car::recode(q17,"4=3"),
         q18 = car::recode(q18,"4=3; 3=2")) %>% 
  mirt(model=1, itemtype='Rasch', verbose = FALSE) %>% 
  plot(type="trace", as.table = TRUE, 
       theta_lim = c(-6,6))
```

Looks better

```{r}
d2 <- d %>% 
  mutate(q11 = car::recode(q11,"2=1;3=2;4=2"),
         q17 = car::recode(q17,"4=3"),
         q18 = car::recode(q18,"4=3; 3=2")) 
```

Looks better

### Test Information (Reliability)
```{r}
RItif(d2, samplePSI = T)
```

Low reliability
```
